# An unexpected synthesis of azepinone derivatives through a metal-free photochemical cascade reaction

**DOI:** 10.1038/s41467-023-36190-z

**Published:** 2023-02-14

**Authors:** Lina Song, Xianhai Tian, Kaveh Farshadfar, Farshad Shiri, Frank Rominger, Alireza Ariafard, A. Stephen K. Hashmi

**Affiliations:** 1 Institut für Organische Chemie, Heidelberg University, Heidelberg, Germany; 2 Department of Chemistry, Islamic Azad University, Central Tehran Branch, Poonak, Tehran, Iran; 3 Research Group of Computational Chemistry, Department of Chemistry and Materials Science, Aalto University, Aalto, Finland; 4 School of Natural Sciences, University of Tasmania, Hobart, TAS Australia; 5 Chemistry Department, Faculty of Science, King Abdulaziz University, Jeddah, Saudi Arabia

**Keywords:** Synthetic chemistry methodology, Computational chemistry, Photochemistry

## Abstract

Azepinone derivatives are privileged in organic synthesis and pharmaceuticals. Synthetic approaches to these frameworks are limited to complex substrates, strong bases, high power UV light or noble metal catalysis. We herein report a mild synthesis of azepinone derivatives by a photochemical generation of 2-aryloxyaryl nitrene, [2 + 1] annulation, ring expansion/water addition cascade reaction without using any metal catalyst. Among the different nitrene precursors tested, 2-aryloxyaryl azides performed best under blue light irradiation and Brønsted acid catalysis. The reaction scope is broad and the obtained products underwent divergent transformations to afford other related compounds. A computational study suggests a pathway involving a step-wise aziridine formation, followed by a ring-expansion to the seven-membered heterocycle. Finally, water is added in a regio-selective manner, this is accelerated by the added TsOH.

## Abstract (web summary)

Photochemical nitrene transfer offers a green avenue for heterocyclic syntheses. Here, the authors developed a metal-free, visible light-mediated cascade reaction for the preparation of azepinone derivatives.

## Introduction

**Fig. 1:**
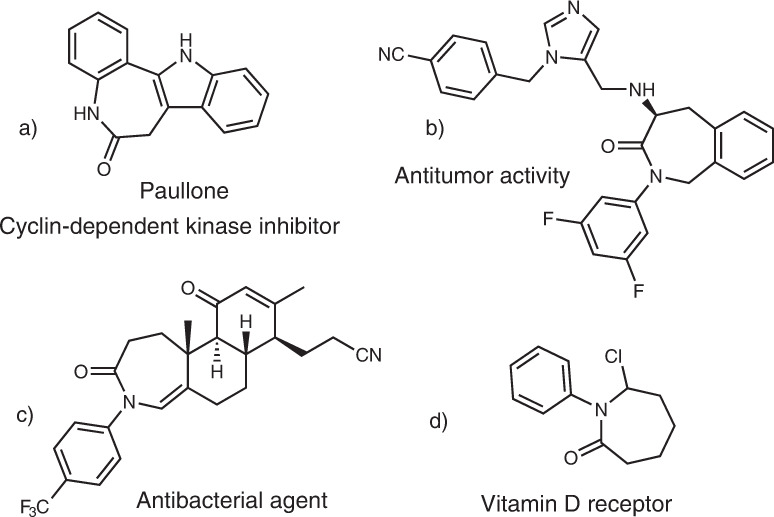
Biologically important azepinone derivatives. **a** Azepinone-based cyclin-dependent kinase inhibitor. **b** Azepinone-based antitumor compound. **c** Azepinone-based antibacterial agent. **d** Azepinone-based vitamin D receptor.

Azepinones and related seven-membered nitrogen-containing heterocycles are distinguished by their versatility in organic synthesis^1^ and ubiquity in various natural products as well as pharmaceutically important compounds (Fig. 1)^2–5^. Traditionally, these compounds were prepared by a strong base-mediated cyclization of complex diester substrates^2^. High-power (>300 W) UV-light promoted decompositions of aryl azides in the presence of nucleophiles also delivered azepinone derivatives^6–9^. Recently, Rh-catalyzed annulations of benzamides with *α*,*β*-unsaturated aldehydes and ketones provided another option for accessing these privileged heterocycles^10^. Despite these advances, a metal-free method using readily available substrates under mild conditions is still challenging and of high utility for synthesis of pharmaceuticals and natural products and the generation of libraries of derivatives.

**Fig. 2:**
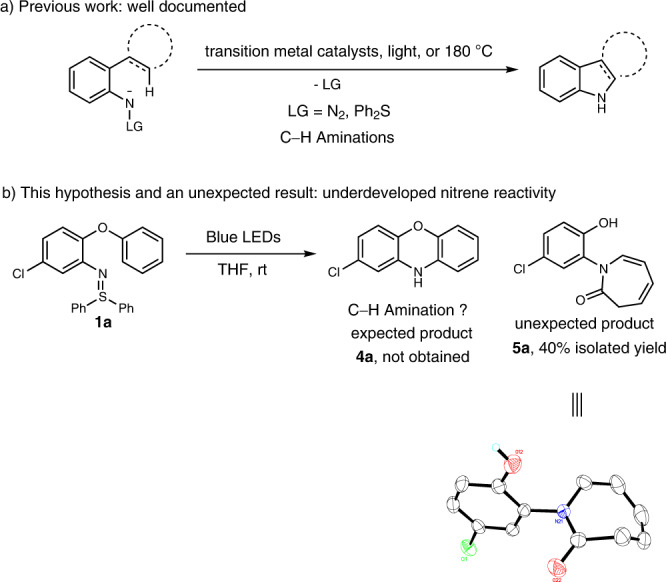
Aryl nitrene precursors for aza-heterocyclic syntheses. **a** Previous work: synthesis of carbazoles and related heterocycles through 2-alkyl, 2-alkenyl or 2-aryl arylnitrene intermediates. **b** This work: synthesis of azepinone derivatives through 2-aryloxyaryl nitrene intermediates.

There is a continuing demand for selective and efficient C − N bond formations for challenging organic synthesis. Organic reactions forging C − N bonds through nitrene intermediates have opened up new avenues for the synthesis of *N*-heterocycles^11–13^. The applicability of these transformations has advanced with the development of new nitrene precursors. In this context, a series of reagents, including azides, 2*H*-azirines, isoxazole derivatives, aza-pyridium ylides and sulfilimines, were elegantly designed^14^. Methods for preparing aza-heterocycles based upon transition metal-catalyzed nitrene transfer from these nitrene equivalents to functionalized alkynes have been frequently reported by Liu^15–17^, Ye^18–21^, Hashmi^22,23^, and others^14^. The direct generation of nitrene intermediates, generally promoted by photoirradiation, high temperatures or transition metal catalysts, allows to form C − N bonds, including C − H amination, C − H amidation and alkene aziridation^24–27^. Among these nitrene transfer reactions, those using visible light source under metal-free conditions are comparatively rare and attractive^26,28,29^. Ortho alkyl-, alkenyl- or aryl-substituted aryl nitrenes have garnered significant attention as such aryl nitrene intermediates can undergo C − H insertions to afford carbazoles or indoles (Fig. 2a)^28,30–32^. To date, the chemistry of 2-aryloxyaryl nitrenes has not yet been explored. Very recently, we disclosed a blue light-promoted synthesis of unprotected carbazoles and indoles by utilizing 2-substituted aryl sulfilimines as nitrene precursors^28^. Based on our ongoing interest in sulfilimine chemistry^22,23,28,33–36^, we envisaged a synthesis of 10*H*-phenoxazine **4a** from 2-phenoxyphenyl sulfilimine **1a** by a photochemical intramolecular C − H amination (Fig. 2b). The desired product **4a**, however, was not observed. An unexpected seven-membered ring product, 2*H*-azepinone **5a**, was formed instead and its structure was confirmed by single crystal X-ray structure analysis. Here, a mild synthesis of azepinone derivatives by leveraging the reactivity of 2-aryloxyaryl nitrene intermediates is presented.

## Results

### Reaction optimization

Optimization commenced with sulfilimine **1a** as substrate (Table 1). Blue light performed better than UVA (entries 1–2). The product yield decreased to 14% when the solvent was changed from THF to toluene and further decreased in DCE (entries 3-4). Adding 10 equivalents of water is beneficial for product formation (entry 5). Other nitrene precursors were also tested. While phosphanimine **2a** did not react under the standard reaction conditions, the product yield was raised to 65% by using azide substrate **3a** (entries 6–7). Adding more H_2_O afforded decrease in the product yield (entries 8–9). The addition of 50 mol% TsOH significantly improved the reaction (entry 10). Both 5 mol% and 100 mol% TsOH led to less efficient reactions (entries 11–12). Other protonic acids and a Lewis acid, Zn(OTf)_2_, delivered the desired product in no higher than 69% yield (entries 13–16). A control experiment in the dark demonstrated that light is essential to this transformation (entry 17).

**Table 1:** Optimization of the reaction conditions^a^


Entry	Substrate	Light source	Additive	Solvent	Yield of 5a (%)^b^
1	**1a**	UVA	—	THF	17
2	**1a**	blue LEDs	—	THF	40
3	**1a**	blue LEDs	—	toluene	14
4	**1a**	blue LEDs	—	DCE	9
5	**1a**	blue LEDs	10 eq. H_2_O	THF	46
6	**2a**	blue LEDs	10 eq. H_2_O	THF	n.d.
7	**3a**	blue LEDs	10 eq. H_2_O	THF	65
8	**3a**	blue LEDs	30 eq. H_2_O	THF	45
9	**3a**	blue LEDs	—	THF/H_2_O = 4/1	28
10	**3a**	blue LEDs	10 eq. H_2_O; 0.5 eq. TsOH	THF	83
11	**3a**	blue LEDs	10 eq. H_2_O; 1.0 eq. TsOH	THF	63
12	**3a**	blue LEDs	10 eq. H_2_O; 0.05 eq. TsOH	THF	69
13	**3a**	blue LEDs	10 eq. H_2_O; 0.5 eq. MsOH	THF	69
14	**3a**	blue LEDs	10 eq. H_2_O; 0.5 eq. AcOH	THF	59
15	**3a**	blue LEDs	10 eq. H_2_O; 0.5 eq. HCl	THF	69
16	**3a**	blue LEDs	10 eq. H_2_O; 0.5 eq. Zn(OTf)_2_	THF	37
17	**3a**	in the dark	10 eq. H_2_O; 0.5 eq. TsOH	THF	n.d.

Reaction conditions: **1a**, **2a** or **3a** (0.1 mmol), additive, solvent (1.0 mL, 0.1 M), RT. ^a^ RT: room temperature; THF: tetrahydrofuran; DCE: 1,2-dichloroethane; MsOH: methanesulfonic acid; TsOH: *p*-toluenesulfonic acid; n.d.: not detected. ^b 1^H NMR yield of **5a** was determined by using 1,3,5-trimethoxyl benzene as the internal standard.

### Substrate scope

**Fig. 3:**
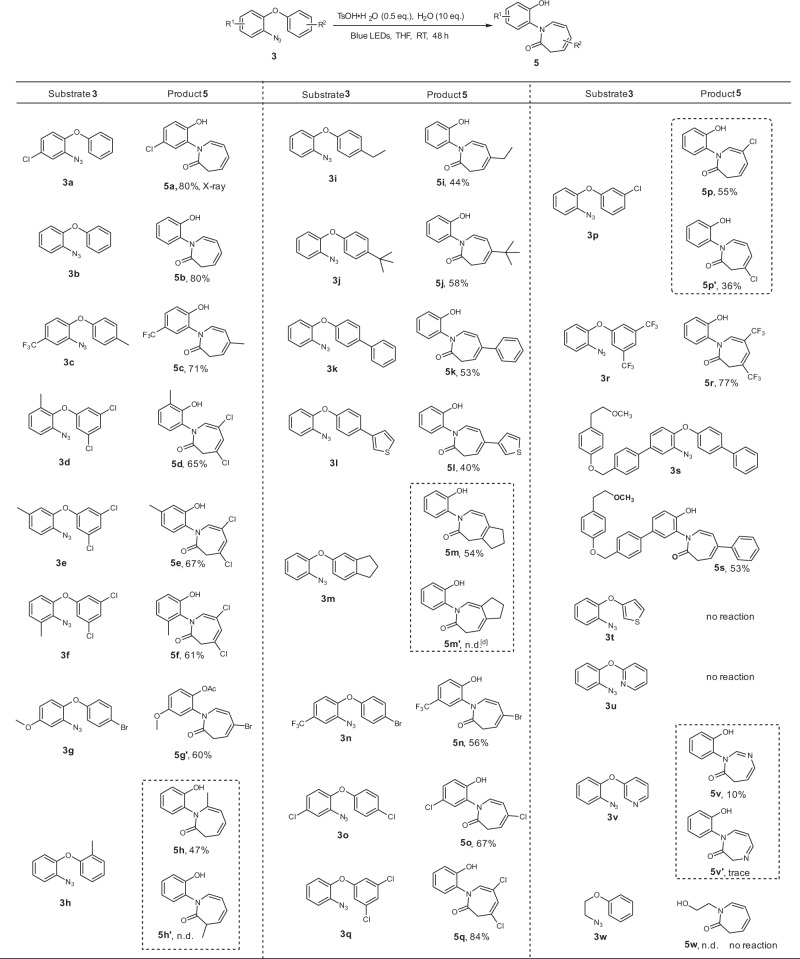
The reaction scope. Reaction conditions: **3** (0.2 mmol), H_2_O (2.0 mmol), TsOH•H_2_O (0.1 mmol), THF (2.0 mL, 0.1 M), blue LEDs, room temperature. Isolated yields of **5** are given. Compound **5g’** was obtained by a subsequent transformation: pyridine (1.5 eq.), acetic anhydride (1.2 eq.), CH_2_Cl_2_ (1.0 mL, 0.1 M), RT.

We then examined the reaction scope and limitations under the optimal reaction conditions (Table 1, entry 10). As shown in Fig. 3, different R^1^ groups at the azido arene moiety, including chloro, trifluoromethyl, 3-methyl, 4-methyl, 6-methyl as well as methoxy groups, were well tolerated, and the corresponding products **5a**-**5f** were obtained in moderate to good yield. Product **5g** was further transformed into acetyl derivative **5g’**. Next, R^2^ substituents were varied to investigate the functional group tolerance further. Azide **3h** bearing a methyl group at ortho position (R^2^ = 2′-methyl) was able to undergo the desired cascade reaction, selectively generating product **5h** in 47% yield. When R^2^ was varied from an alkyl group (ethyl or *tert*-butyl) to an aryl substituent (phenyl or thiophenyl) at the para positions, the reactions proceeded smoothly and product yield ranged from 40% to 67%. A dihydroindene-based substrate **3m** afforded a single isomer, **5m**, in good yield. The *para*-bromo and -chloro substrates **5n**-**5o** were very well tolerated and the obtained products are valuable starting materials for further cross coupling strategies. The *meta*-chloro substrate **3p** delivered two isomers **5p** and **5p**′ in 91% combined yield with low levels of regioselectivity. 3′,5′-Dichloro and 3′,5′-ditrifluoromethyl groups remained untouched, the products **5q** and **5r** were isolated in high yield. A complex substrate **3s** was also tested, enabling 53% yield of the desired product **5s**. Encouraged by the performance of aryloxyaryl azides, we became interested in evaluating heteroaryloxy and alkoxy type substrates under the standard conditions. Although the thiophene and 2-pyridinyl substrates (**3t** and **3u**) remained unreactive, 3-pyridinyl substrate **3** **v** regioselectively afforded 1,3-diazepinone **5v** in 10% yield with 20% conversion. An attempt to synthesize *N*-alkyl azepinone **5w** by engaging alkoxy substrate **3w** was unsuccessful.

**Fig. 4:**
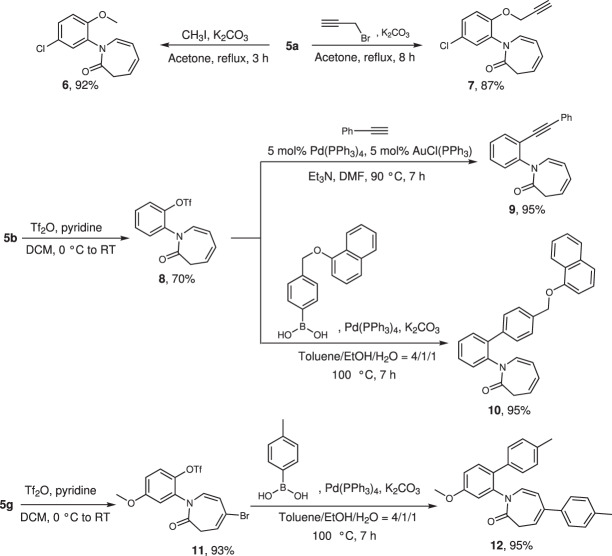
Important transformations based on the obtained products. All reactions are performed on a 0.2 mmol scale. Isolated yields are given.

Our attention then turned to the synthetic utilizations of the obtained products (Fig. 4). The unprotected phenol products **5** can be protected by different protecting groups, such as methyl, propargyl and trifluoromethanesulfonyl, resulting in products **6**, **7**, **8**, **11** in high yield. The generated triflate group can further be coupled with phenylacetylene or a complex arylboronic acid in excellent yield. Compound **11**, containing a triflate and a bromo substituent, was able to efficiently react with 2 equivalents of *para*-tolylboronic acid under palladium catalysis.

### Mechanistic studies

**Fig. 5:**
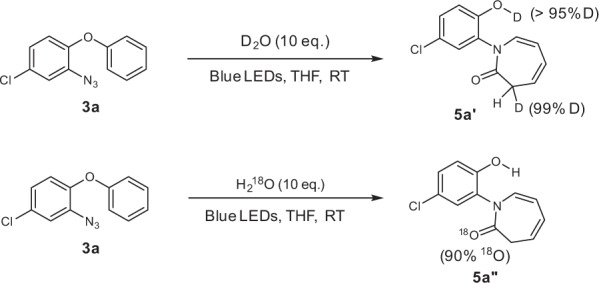
Deuterium and ^18^O labeling experiments. The H/D ratios were determined by ^1^H NMR spectroscopy with trimethoxybenzene as the internal standard. The incorporation of the ^18^O atom was detected by MS and a second carbonyl peak in the ^13^C NMR spectrum.

In an effort to elucidate the reaction mechanism, we conducted an isotope labeling experiment by adding D_2_O instead of H_2_O (Fig. 5). As a result, 50% D was found in the CH_2_ group and >95% D was found in the OH group of the product, which suggested that both the proton in OH group and one of the protons in the CH_2_ group of the product originated from H_2_O. We further conducted an ^18^O-labeling experiment by using ^18^O water (98% ^18^O). Mass spectrometry does not indicate any two-fold incorporation, 90% ^18^O were incorporated, but due to the fragmentation pathways, MS did not allow to assign the ^18^O to either the ketone or the hydroxyl group. Thus the obtained product **5a”** was analyzed by ^13^C NMR (150 MHz). The carbonyl carbon signal at 167.12 ppm could be assigned to the ^16^O carbonyl by comparison with the unlabeled product **5a**, the signal at 167.09 ppm thus belongs to the ^18^O labeled **5a”** (Supplementary Fig. 14). Thus the carbonyl oxygen of product **5a”** indeed is derived from the external water nucleophile.

**Fig. 6:**
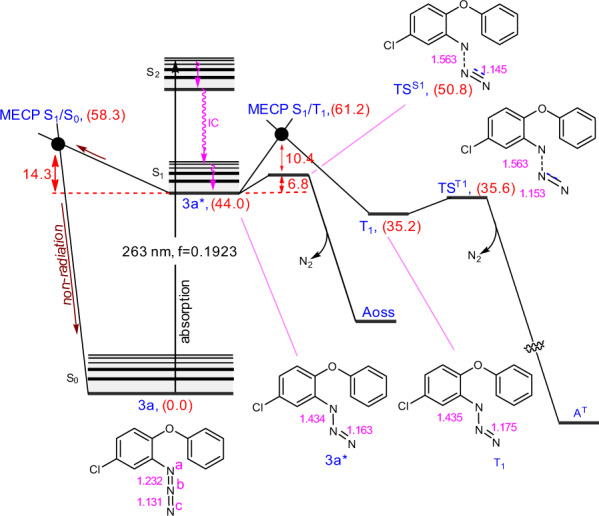
Calculated potential energy profile for light-driven decomposition of aryl azide **3a**. The relative potential energies are given in kcal/mol.

It is well established that light decomposes aryl azides into open-shell singlet nitrenes^37^. To confirm this, we utilized TD-DFT calculations at the CPCM/CAM-B3LYP/def2-TZVP//CPCM/CAM-B3LYP/6-31G(d) level of theory to investigate the mechanism of photoactivation (Fig. 6). In the calculations, we used substrate **3a** as a representative for the aryl azides. The first excitation was identified at 301 nm with a negligible oscillator strength (*f* = 0.0003), therefore it was not computationally investigated further. The second excitation at 263 nm has a significant oscillator strength with f = 0.1923. This excitation mainly corresponds to the transition of an electron from the HOMO to the LUMO of substrate **3a**. Attempts to optimize this excited state, however, always collapsed to the S_1_ state, implying that the internal conversion (IC) from S_2_ to S_1_ should be extremely fast. The optimization of this excited state results in a structure with a much weakened N^a^–N^b^ bond, as supported by the elongation of the corresponding bond from 1.232 Å in **3a** to 1.434 Å in 3a*. The resultant 3a* could then serve as a branching point for several pathways, three of which are depicted in Fig. 6. This structure can be involved in breaking the N^a^–N^b^ bond via transition structure **TS**^**S1**^ to form open-shell singlet structure **A**^**OSS**^, or it can give triplet structure **T**_**1**_ via **MECP S**_**1**_**/T**_**1**_, or it can relax non-radiatively to structure **3a** by crossing M**ECP S**_**1**_**/S**_**0**_. According to the results of the calculations, **TS**^**S1**^ has a significantly lower energy than both **MECP S**_**1**_**/T**_**1**_ and **MECP S**_**1**_**/S**_**0**_. This implies that open-shell singlet structure **A**^**OSS**^ should be formed significantly faster than other species, which is completely consistent with previous findings, indicating that light-driven decomposition of aryl azides produces directly open-shell singlet nitrenes^37^.

**Fig. 7:**
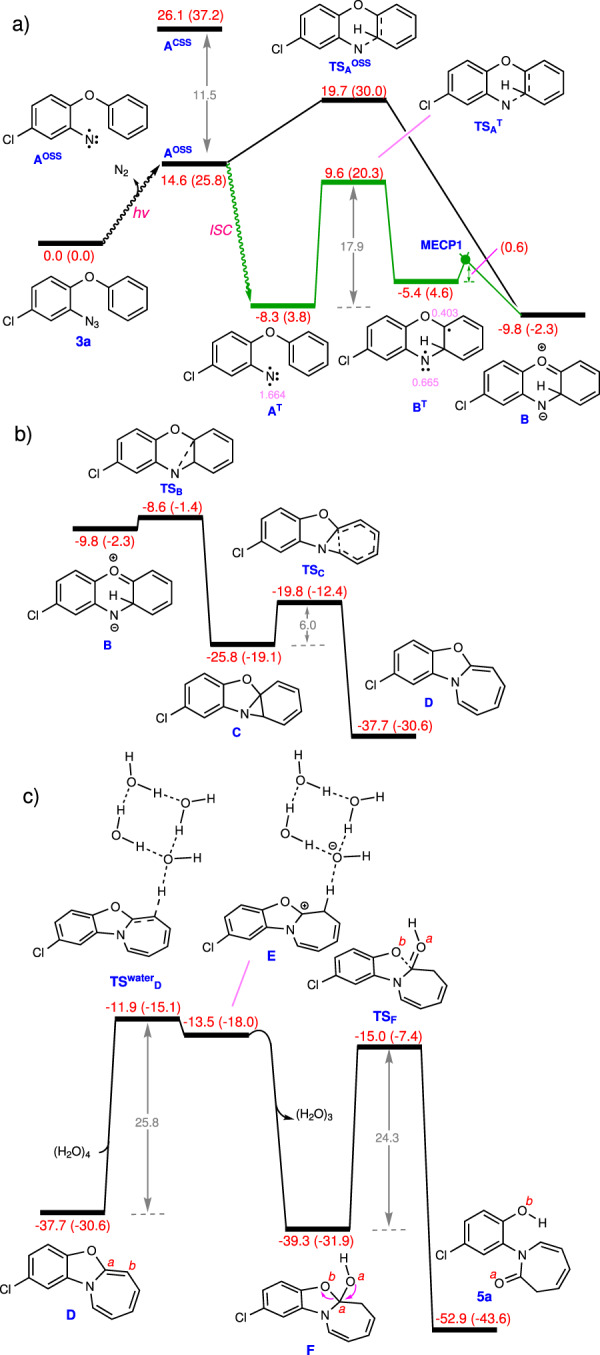
Calculated energy profiles. **a** The calculated energy profile of the aziridation step. **b** The calculated energy profile of the aziridation step for the ring-closing and first ring-opening steps. **c** The calculated energy profile of the hydrolysis and second ring-opening steps. Free energies (potential energies) calculated at the SMD/M06-2X/def2-TZVP//SMD/M06-2X/6-31G(d) level of theory in THF are given in kcal/mol. The triplet pathway is shown in green. The pink values are the Mulliken spin density on selective atoms in **A**^**T**^ and **B**^**T**^.

To understand how the title reaction proceeds, we investigated its mechanistic details with the aid of the density functional theory (DFT) by using the SMD/M06-2X/def2-TZVP//SMD/M06-2X/6-31G(d) calculations in THF (for details, see Supplementary Data 1–2). The unrestricted open-shell singlet **A**^**OSS**^ is computed to be 14.6 kcal/mol higher in energy than **3a** (Fig. 7a). We have also calculated the restricted closed-shell singlet for structure **A** and found this structure (**A**^**CSS**^) with a free energy of 26.1 kcal/mol is far less stable than **A**^**OSS**^ (Fig. 7a). The intersystem crossing (ISC) to the triplet state gives **A**^**T**^, which is 22.9 kcal/mol more stable than **A**^**OSS**^^38^. The intramolecular aziridation was then investigated from **A**^**OSS**^ and **A**^**T**^. Our DFT calculations demonstrate that both **A**^**OSS**^ and **A**^**T**^ intermediates are reactive toward the aziridation. The unrestricted open-shell intermediate directly gives Zwitterion intermediate **B** via transition structure **TS**_**A**_^**OSS**^, which is only 5.1 kcal/mol higher in energy than **A**^**OSS**^. **A**^**T**^ is connected to **B**^**T**^ on the triplet surface by surmounting an activation free energy of 17.9 kcal/mol via transition structure **TS**_**A**_^**T**^. The resultant triplet intermediate **B**^**T**^ then undergoes a spin flip transition via **MECP1** to form more stable Zwitterion intermediate **B** on the single surface; the **MECP1** is found to be only 0.6 kcal/mol above **B**^**T**^. The exergonicity for transformation **3a** → **A**^**T**^ + N_2_ is further supported by our additional calculations at the SMD/CCSD(T)/def2-TZVP//SMD/M06-2X/6-31 G(d) level of theory for N_2_ release from a related system (phenyl azide, Supplementary Fig. 15d); the single-point calculations using CCSD(T) yield an ΔE of +1.3 kcal/mol for the N_2_ release, suggesting that the reaction is exergonic with ΔG = −11.1 kcal/mol when the entropy contribution from the SMD/M06-2X/6-31 G(d) calculations is taken into account (Supplementary Fig. 15d). The Mulliken spin density analysis for structure **A**^**T**^ indicates that the two single electrons are mainly located on the nitrogen atom with a contribution of 1.664 (Fig. 7a). The rest of the electrons, due to the π conjugation effect are distributed on the carbon atoms of the six-membered ring bonded to the N atom with a very negligible contribution from the oxygen atom (0.045, for details, see Supplementary Fig. 16). A similar electron distribution was also obtained for **A**^**OSS**^, with the nitrogen atom contributing the most with a value of 0.529 (Supplementary Fig. 16).

Once **B** has formed, it is involved in a ring-closing process and furnishes **C** (Fig. 7b). This process is predicted to occur extremely fast with a calculated activation barrier as low as 1.2 kcal/mol. Intermediate **B** is also prone to participate in an aromatization process to give a phenoxazine. Our DFT calculations indicate that although the aromatization process from **B** is predicted to be fast with an activation barrier of ~11 kcal/mol (Supplementary Fig. 17), it should take place much slower than the ring-closing transformation **B** → **C**, resulting in no phenoxazine being formed during the light-promoted reaction. This is in contrast to the observations in our recent report^28^ where the nitrene produced from sulfilimines practically only affords carbazoles attributed to favourability of the aromatization process over the ring-closing (Supplementary Fig. 18). We also investigated the direct formation of **C** from **B**^**T**^ (Supplementary Fig. 20) and found that it is 1.8 kcal/mol less favorable than the stepwise pathway following the sequence **B**^**T**^ → **B** → **C**. Once **C** has formed, it undergoes ring expansion to give tricyclic intermediate **D** (Fig. 7b). This step is computed to be exergonic by about 11.9 kcal/mol and occurs with a small activation barrier of 6.0 kcal/mol. Next, product **5a** is generated by the hydrolysis of intermediate **D** followed by a second ring-opening process. The hydrolysis starts with the addition of a proton from water to the C^*b*^ atom of **D**. Similar to the previous studies, a water cluster of three water molecules was employed for this step (Fig. 7c)^39,40^. The presence of the π-donor atoms on the C^a^ atom, particularly the N atom, makes the C^*b*^ atom in **D** so basic that it deprotonates water with a moderate activation free energy of 25.8 kcal/mol to give the ion pair **E** (Supplementary Fig. 19). Subsequently, the in situ generated hydroxide ion is trapped by the carbocation stabilized by the oxygen and nitrogen atoms in **E** and produces intermediate **F**. Finally, the breaking of the C^a^–O^b^ bond (the second ring-opening process) via transition structure **TS**_**F**_ directly affords final product **5a**. This means that when the C^a^-O^b^ bond is completely broken, the proton transfer from O^a^ to O^b^ occurs spontaneously. Efforts to identify a transition structure in which the proton transfer occurs prior to the C^a^-O^b^ bond breaking were unsuccessful.

Two important points can emerge from these calculations: (i) the hydrolysis mechanism proposed by the computations explains well the isotope labeling experiments outlined in Fig. 5 and (ii) the rate-determining step is found to be the hydrolysis step with ΔG^‡^ = 25.8 kcal/mol. This relatively high activation barrier rationalizes why the yield is moderate when only water is used as the additive.

**Fig. 8:**
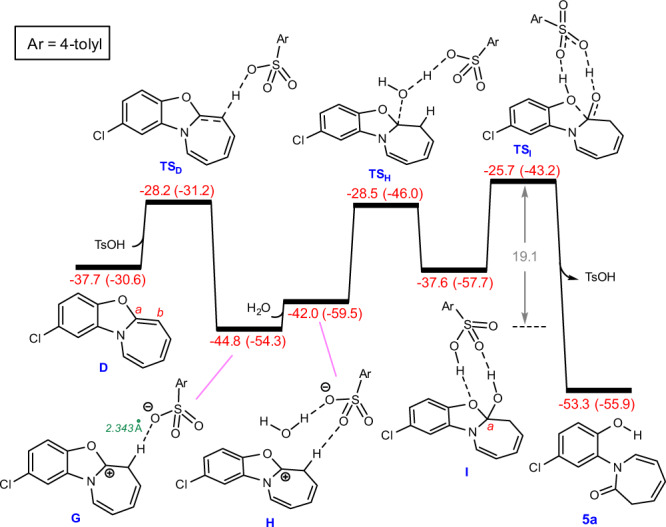
Calculated energy profile for TsOH-catalyzed hydrolysis and second ring-opening steps. Free energies (potential energies) calculated at the SMD/M06-2X/def2-TZVP//SMD/M06-2X/6-31G(d) level of theory in THF are given in kcal/mol.

Figure 8 shows the role of TsOH in catalyzing the hydrolysis and second ring-opening steps. The use of TsOH decreases the overall barrier to 19.1 kcal/mol, resulting in the reaction proceeding experimentally with a much higher yield. As shown in Fig. 8, the hydrolysis step is commenced by protonation of the C^*b*^ atom of **D** through transition structure **TS**_**D**_ with an activation free energy of 9.5 kcal/mol. The reaction is then followed by the formation of hydrogen-bonded adduct **H**, which subsequently gives intermediate **I** through trapping the carbocation by the TsO^-^-stabilized water. Finally, the second ring-opening process from **I** takes place by overcoming a barrier of 11.9 kcal/mol via **TS**_**I**_. The relatively low barrier calculated for this step confirms that TsOH plays a crucial role in its facilitation. In short, the hydrolysis process is determined to be the rate-determining step for the formation of product **5a**. The activation free energy of this step is calculated to be 25.8 kcal/mol (Fig. 7c), whereas TsOH facilitates the hydrolysis, allowing it to proceed with a lower activation free energy of 19.1 kcal/mol (Fig. 8).

**Fig. 9:**
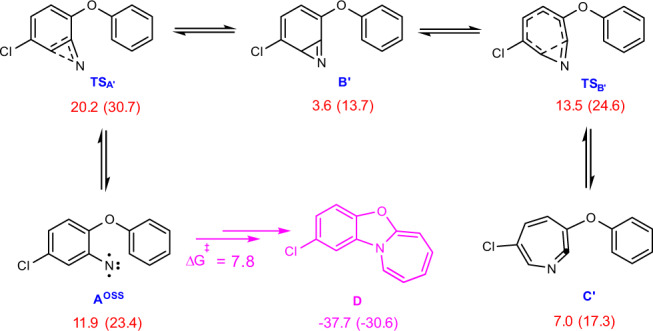
Calculated reaction mechanism for formation of normally produced Bʹ and Cʹ. Free energies (potential energies) calculated at the SMD/M06-2X/def2-TZVP//SMD/M06-2X/6-31G(d) level of theory in THF are given in kcal/mol.

For the sake of completeness, we also investigated computationally the possibility of formation of the normally produced **Bʹ** and **Cʹ** (Fig. 9)^6–9^. Although the formation of such intermediates is calculated to be kinetically feasible, those are thermodynamically highly unstable with respect to intermediate **D**. As a result, once formed, they can involve a reverse process with an activation energy of 16.6 kcal/mol (free energy difference between **TS**_**Aʹ**_ and **Bʹ**) to yield the thermodynamically stable intermediate **D** from which the final product **5a** is formed by the above discussed mechanism.

In summary, we have reported a green method for the synthesis of azepinone derivatives by using readily available 2-aryloxy aryl azides as aryl nitrene precursors under blue light irradiation. An isotope labeling experiment suggested that H_2_O is key to the formation of the final products. A detailed computational study rationalized the reaction pathway. The current reaction features a broad scope, mild conditions, and good efficiency, providing an alternative method for the synthesis of these seven-membered aza-heterocycles without using any transition metals. The isolated products are versatile substrates for further transformations.

## Methods

### Synthesis of substituted compound 5

Under nitrogen atmosphere, to a solution of substrate **3** (0.2 mmol) in 2.0 mL THF were added TsOH·H_2_O (19 mg, 0.1 mmol, 0.5 eq.) and H_2_O (36 mg, 2.0 mmol, 10 eq.). The resulting mixture was irradiated at room temperature with 29 W blue LEDs for 48 h. The solvent was removed under reduced pressure and the residue was purified by column chromatography on silica gel to provide the desired product **5**.

## Supplementary information

Supplementary Information

Description of Additional Supplementary Files

Dataset 1

Dataset 2

## Data Availability

Experimental procedures, mechanistic studies, characterization data, copies of NMR spectra and computational details are available in the Supplementary Information. Crystallographic parameters for compound **5a** are available free of charge from the Cambridge Crystallographic Data Centre under deposition number CCDC 2026384 (**5a**).
