# Supplementary material for: An unexpected synthesis of azepinone derivatives through a metal-free photochemical cascade reaction
Source: Nat Commun. 2023 Feb 14;14:831. doi: 10.1038/s41467-023-36190-z (PMC9929248; doi:10.1038/s41467-023-36190-z)
Supplement: Supplementary file 1 — Supplementary Information [file 41467_2023_36190_MOESM1_ESM.pdf]

Supplementary Information

An Unexpected Synthesis of Azepinone Derivatives through a Metal-Free Photochemical Cascade Reaction

Lina Song, Xianhai Tian*, Kaveh Farshadfar, Farshad Shiri, Frank Rominger, Alireza Ariafard* and A. Stephen K. Hashmi*

Correspondence to:
xhtian1013@outlook.com
Alireza.Ariafard@utas.edu.au
hashmi@hashmi.de

Contents

1. Supplementary Notes 1

2. Supplementary Methods 2

Procedure 1: Synthesis of substrates 3 2
Procedure 2: Photochemical synthesis of substituted compound 5 3
Procedure 3: Synthesis of compound 6 3
Procedure 4: Synthesis of compound 7 4
Procedure 5: Synthesis of compound 9 4
Procedure 6: Synthesis of compound 10 and 12 5
Characterization data 5

3. Supplementary Discussion 20

Mechanistic investigation 20
Additional calculation results and computational details 23

4. Supplementary Figures 29

X-ray structure 29
NMR spectra 30
UV-Vis spectra of substrates 3 82

5. Supplementary References 89

1. Supplementary Notes

Chemicals were purchased from commercial suppliers and used directly as delivered. Dry solvents were dispensed from the solvent purification system MB SPS-800. The emission spectrum of the blue LEDs (maximum 470 nm) is available in our previous publication.[1] Deuterated solvents were bought from Euriso-Top. NMR spectra were, if not mentioned otherwise, recorded at room temperature on the following spectrometers: Bruker Avance-III-300, Bruker Avance III 400, and Bruker Avance-III-500. Chemical shifts are given in ppm and coupling constants in Hz. The following abbreviations were used for ^{1}H NMR spectra to indicate the signal multiplicity: s (singlet), brs (broad singlet), d (doublet), t (triplet), q (quartet), quint (quintet), sext (sextet), hept (heptet) and m (multiplet) as well as combinations of them. When combinations of multiplicities are given the first character noted refers to the biggest coupling constant. All ^{13}C NMR spectra were measured with ^{1}H-decoupling. The multiplicities mentioned in these spectra [s (singlet, quaternary carbon), d (doublet, CH-group), t (triplet, CH_2-group), q (quartet, CH_3-group)] were determined by DEPT135 spectra. Mass spectra (HRMS) were determined at the chemistry department of the University of Heidelberg under the direction of Dr. J. Gross. For EI+-, ESI+- or DART+-spectra, a Bruker Apex-Qu FT-ICR-MS spectrometer was applied. Infrared Spectroscopy (IR) was processed on an FT-IR Bruker (IF528), IR Perkin Elmer (283) or FT-IR Bruker Vector 22. The solvent or matrix is denoted in brackets. For the most significant bands the wave number ν (cm^{-1}) is given. X-ray crystal structure analyses were measured at the chemistry department of the University of Heidelberg under the direction of Dr. F. Rominger on a Bruker Smart CCD or Bruker APEX-II CCD instrument using Mo-Kα-radiation. Diffraction intensities were corrected for Lorentz and polarization effects. An empirical absorption correction was applied using SADABS based on the Laue symmetry of reciprocal space. Heavy atom diffractions were solved by direct methods and refined against F2 with full matrix least square algorithm. Hydrogen atoms were either isotropically refined or calculated. The structures were solved and refined by Dr. F. Rominger using the SHELXTL software package. Melting Points were measured in open glass capillaries in a Büchi melting point apparatus (according to Dr. Tottoli) and were not calibrated. Flash Column Chromatography was accomplished using silica gel 60 (0.04-0.063 mm / 230-400 mesh ASTM) purchased from Aldrich. As eluents, mixtures of petroleum ether (PE), ethyl acetate (EA) were used. Analytical Thin Layer Chromatography (TLC) was carried out on precoated Macherey-Nagel POLYGRAM® SIL G/UV254 or POLYGRAM® ALOX N/UV254 plastic sheets. Detection was accomplished using UV-light (254 nm), or KMnO4 (in 1.5 M Na_2CO_3 (aq.)). IUPAC names of the compounds described in the experimental section were determined with the program ACDLabs 12.0®.

2. Supplementary Methods

Procedure 1: Synthesis of substrates 3[2,3]

Synthesis of substituted 1-(2-nitrophenoxy)benzenes:

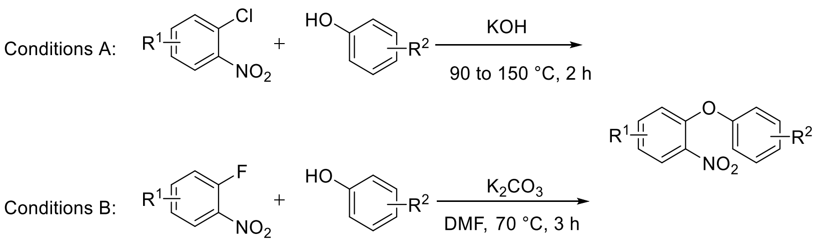

Supplementary Figure 1. Synthesis of aryloxy nitroarenes. Conditions A: from 2-chloro nitroarenes; Conditions B: from 2-fluoro nitroarenes.

Condition A: At 90 ℃, KOH (616 mg, 11 mmol, 1.1 eq.) was added to substituted phenol (11 mmol, 1.1 eq.) in a pressure tube, and the reaction mixture was stirred for 15 min at the same temperature. 1-Chloro-2-nitrobenzene derivative (10 mmol, 1 eq.) was then added slowly and stirring continued for another 2 h at 150 ℃. Then the reaction mixture was cooled down, added 20 ml EtOAc and transferred into a separating funnel. The organic layer was washed with NaOH solution (5%, 3 × 20 mL), dried over anhydrous Na_2SO_4 and evaporated under reduced pressure to afford substituted 1-(2-nitrophenoxy)benzene (75–95% yield) which was used directly without further purification.

Condition B: At room temperature, substituted nitrofluorobenzene (10 mmol, 1 eq.) was added slowly to a mixture of substituted phenol (11 mmol, 1.1 eq.) and K_2CO_3 (6.91 g, 50 mmol, 5 eq.) in DMF. The resulting mixture was stirred for 3 h at 70 ℃**.** Upon completion of the reaction, the reaction mixture was cooled down, diluted with water (30 mL) and extracted with EA (3 × 30 mL). The combined organic layer was washed with H_2O (2 × 30 mL), dried over anhydrous Na_2SO_4, filtered and concentrated. The residue was purified by silica gel column chromatography using a mixture of petroleum ether and EtOAc as eluent to give (2-nitrophenoxy)benzene.

Synthesis of substituted 2-aryloxy aryl azides:

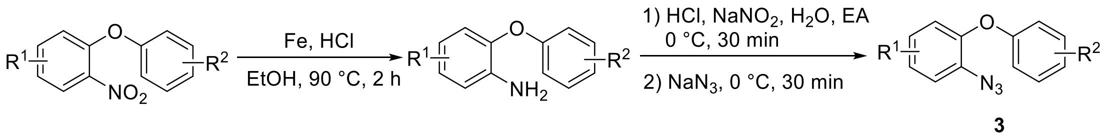

3

Supplementary Figure 2. Synthesis of aryloxy aryl azides 3.

To a slurry mixture of iron powder (2.8 g, 50 mmol, 5 eq.) in H_2O (10.0 mL) and EtOH (10.0 mL) was added substituted 1-(2-nitrophenoxy)benzene (10 mmol, 1 eq.) at room temperature. After conc.HCl (0.3 mL) was added dropwise, the reaction mixture was stirred for 5 h at 90 ℃. Then the reaction mixture was alkalized by 5% NaOH aqueous solution and then filtered. The filtrate was evaporated under reduced pressure to remove EtOH. The resulting mixture was then extracted with

CH_2Cl_2 (3 × 30 mL). The combined organic layer was dried over anhydrous Na_2SO_4, filtered and concentrated. The residue was purified by silica gel column chromatography using a mixture of petroleum ether and EtOAc as eluent to give 2-aryloxy anilines (70–98% yield).

To a mixture of substituted 2-aryloxy aniline (5.00 mmol, 1 eq.) in EA (9.2 mL) and H_2O (1.2 mL) was added conc. HCl (2.7 mL). The resulting miture was stirred for 10 min at room temperature and then it was cooled to 0 ℃ and a solution of $NaNO_2$ (583 mg, 8.45 mmol, 1.69 eq.) in H_2O (2.0 mL) was added slowly. Upon completion of the addition, stirring continued for 30 min at 0 ℃ followed by a slow addition of NaN_3 (553 mg in 2 mL H_2O, 8.50 mmol, 1.7 eq.) aqueous solution. After 30 min at 0 ℃, the reaction mixture was diluted with 15 mL water, extracted with EtOAc (3 × 20 mL). The combined organic layer was washed with 5% NaOH solution (1 × 50 mL), then with water (1 × 50 mL), dried over Na_2SO_4, filtered and concentrated to afford azide **3**.

Procedure 2: Photochemical synthesis of substituted compound 5

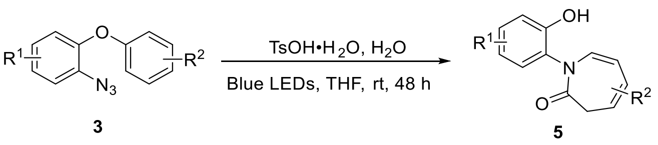

Supplementary Figure 3. Synthesis of azepinone 5.

Under nitrogen atmosphere, to a solution of substrate **3** (0.2 mmol) in 2.0 mL THF were added TsOH H₂O (19 mg, 0.1 mmol, 0.5 eq.) and H_2O (36 mg, 2.0 mmol, 10 eq.). The resulting mixture was irradiated at room temperature with 29 W blue LEDs for 48 h. The solvent was removed under reduced pressure and the residue was purified by silica gel column chromatography using a mixture of petroleum ether and EtOAc as eluent to provide the desired product **5**.

Procedure 3: Synthesis of compound 6[4]

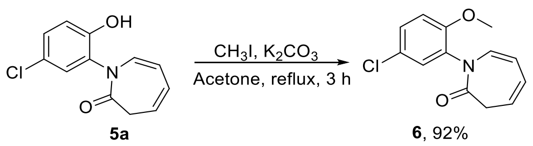

Supplementary Figure 4. Synthesis of 6 through methylation of 5a.

To a solution of **5a** (48 mg, 0.2 mmol, 1.0 eq.) in 2.0 mL acetone were added K_2CO_3 (42 mg, 0.3 mmol, 1.5 eq.) and CH_3I (43 mg, 0.3 mmol, 1.5 eq.). The resulting mixture was heated to reflux for 3 h. Then the reaction mixture was cooled, diluted with water (15 mL) and extracted with EA (3 × 15 mL). The combined organic layer was dried over Na_2SO_4, filtered and concentrated. The residue was purified by silica gel column chromatography using a mixture of petroleum ether and EtOAc as eluent to provide the desired product **6** (46 mg, 92%)

Procedure 4: Synthesis of compound 7[5]

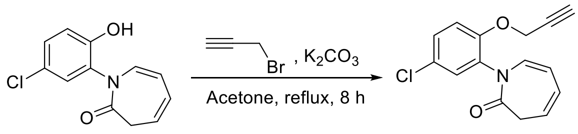

Supplementary Figure 5. Synthesis of 7 through propargylation of 5a.

To a solution of **5a** (47 mg, 0.2 mmol, 1.0 eq.) in 2.0 mL acetone was added K_2CO_3 (110 mg, 0.8 mmol, 4.0 eq.) at room temperature. The resulting mixture was stirred at 70 ℃ for 0.5 h followed by the dropwise addition of 3-bromoprop-1-yne (48 mg, 0.4 mmol, 2.0 eq.) and then stirring continued for 8 h at 70 ℃. Upon completion, the reaction mixture was filtered through a pad of silica gel. The filtrate was concentrated under reduced pressure and the residue was purified by flash silica gel column chromatography to give product **7** (47 mg, 87%).

Procedure 5: Synthesis of compound 9[6]

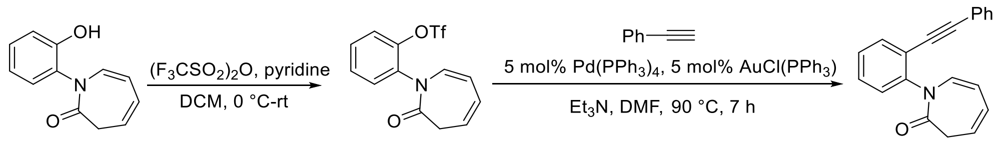

Supplementary Figure 6. Synthesis of 9 through sequential protection and Suzuki coupling.

To a solution of **5b** (140 mg, 0.7 mmol, 1.0 eq.) and pyridine (0.12 mL, 1.4 mmol, 2.0 eq.) in anhydrous CH_2Cl_2 (5 mL) was added dropwise a solution of Tf_2O (0.14 mL, 0.84 mmol, 1.2 eq.) in 5 mL anhydrous CH_2Cl_2 at 0 ℃. The resulting mixture was stirred at room temperature for 1.5 h and then quenched by the addition of 1 mL H_2O. The resulting mixture was transferred into a separating funnel followed by the addition of 10% $NaHCO_3$ aqueous solution (20 mL) and 15 mL CH_2Cl_2. The organic layer was collected and the aqueous phase was extracted with CH_2Cl_2 (2 × 20 mL). The combined organic layer was dried over Na_2SO_4, filtered and concentrated. The residue was purified by column chromatography on silica gel to provide compound **8** (163 mg, 70%).

A mixture of $Pd(PPh_3)_4$ (5.0%, 12 mg, 0.01 mmol), $AuCl(PPh_3)$ (5.0%, 5 mg, 0.01 mmol) and compound **8** (67 mg, 0.2 mmol, 1.0 eq.) in an oven-dried flask was degassed and backfilled with nitrogen. A solution of ethynylbenzene (32 mg, 0.3 mmol, 1.5 eq.) and Et_3N (62 mg, 0.6 mmol, 3.0 eq.) in degassed DMF (1.0 mL) was added via syringe. The resulting mixture was stirred at 90 ℃ for 7 h, cooled to room temperature and then filtered. The filtrate was evaporated and the residue was purified by silica gel column chromatography to afford product **9** (54 mg, 95%).

Procedure 6: Synthesis of compound 10 and 12

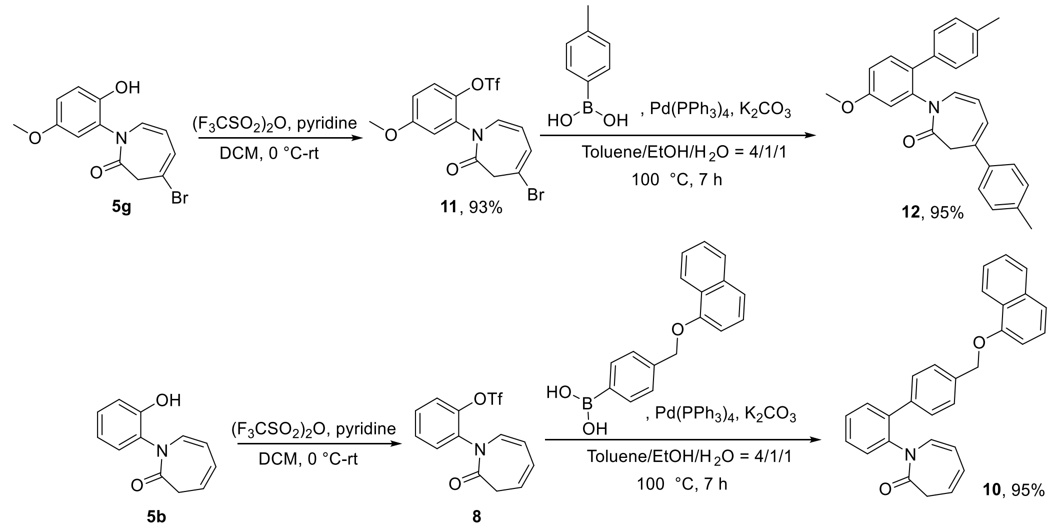

Supplementary Figure 7. Synthesis of 10 or 12 through sequential protection and Suzuki coupling.

To a solution of **5 g** (118 mg, 0.38 mmol, 1.0 eq.) and pyridine (60 mg, 0.76 mmol, 2.0 eq.) in anhydrous CH_2Cl_2 (3 mL) was added dropwise a solution of Tf_2O (0.08 mL, 0.46 mmol, 1.2 eq.) in 3 mL anhydrous CH_2Cl_2 at 0 ℃. The resulting mixture was stirred at room temperature for 1.5 h and then quenched by the addition of 1 mL H_2O. The resulting mixture was transferred into a separating funnel followed by the addition of 10% $NaHCO_3$ aqueous solution (20 mL) and 15 mL CH_2Cl_2. The organic layer was collected and the aqueous phase was extracted with CH_2Cl_2 (2 × 20 mL). The combined organic layer was dried over Na_2SO_4, filtered and concentrated. The residue was purified by column chromatography on silica gel to provide compound **11** (156 mg, 93%).

A mixture of $Pd(PPh_3)_4$ (3.0%, 6.9 mg, 0.006 mmol), arylboronic acid (0.95 mmol, 3.0 eq.), compound **11** (140 mg, 0.32 mmol, 1.0 eq.) or **8** (67 mg, 0.2 mmol, 1.0 eq.), toluene (4.0 mL), ethanol (1.0 mL), H_2O (1.0 mL) and K_2CO_3 (150 mg, 1.1 mmol, 3.4 eq.) was degasses and stirred at 100 ℃ for 6 h under nitrogen. Upon completion, the reaction was neutralized by 5% aqueous HCl, then the aqueous phase was separated and further extracted with EtOAc (2 × 5 mL). The combined organic layers were dried over Na_2SO_4, filtered and concentrated. The residue was purified by silica gel column chromatography to afford product **12** or **10**.

Characterization data

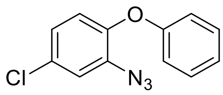

2-azido-4-chloro-1-phenoxybenzene (3a)

Yield: 1.16 g, 95%; brown oil; R_f = 0.26 (PE); ^{1}H NMR (300 MHz, $CDCl_3$) δ 7.26-7.18 (m, 2H), 7.04-6.96 (m, 2H), 6.93 (dd, J = 2.4, 8.7 Hz, 1H), 6.88-6.80 (m, 2H), 6.76 (d, J = 8.7 Hz, 1H) ppm; ^{13}C NMR (75 MHz, $CDCl_3$) δ 157.0 (s), 147.1 (s), 132.9 (s), 130.0 (d, 2C), 129.7 (s), 125.6 (d), 123.7 (d), 121.9 (d), 121.1 (d), 117.6 (d, 2C) ppm; IR (reflection) $\tilde{\nu}$ = 3065, 3041, 2387, 2112, 1654, 1587, 1489, 1404, 1297, 1236, 1196, 1163, 1110, 1023, 893, 852, 836, 750, 690 cm^{-1}; HRMS (EI)

(m/z) [M] $C_{12}H_8{}^{35}ClN_3O$ calcd for 245.0350, found 245.0351.

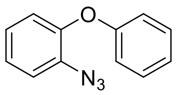

1-azido-2-phenoxybenzene (3b)

Yield: 1.00 g, 95%; brown oil; R_f = 0.16 (PE); ^{1}H NMR (300 MHz, $CDCl_3$) δ 7.26-7.19 (m, 2H), 7.05-6.95 (m, 4H), 6.90-6.79 (m, 3H) ppm; ^{13}C NMR (75 MHz, $CDCl_3$) δ 157.3 (s), 148.2 (s), 131.7 (s), 129.8 (d, 2C), 125.8 (d), 124.9 (d), 123.3 (d), 121.2 (d), 121.0 (d), 117.6 (d, 2C) ppm; IR (reflection) $\tilde{\nu}$ = 2427, 2135, 1942, 1598, 1581, 1488, 1453, 1292, 1273, 1230, 1165, 1152, 1096, 1073, 1025, 935, 873, 790, 749, 729, 689, 658 cm^{-1}; HRMS (EI) (m/z) [M] $C_{12}H_9N_3O$ calcd for 211.0740, found 211.0745.

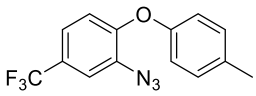

2-azido-1-(p-tolyloxy)-4-(trifluoromethyl)benzene (3c)

Yield: 950 mg, 65%; orange oil; R_f = 0.29 (PE); ^{1}H NMR (500 MHz, $CDCl_3$) δ 7.25 (s, 1H), 7.20 (d, J = 8.5 Hz, 1H), 7.11 (d, J = 8.5 Hz, 2H), 6.85 (d, J = 8.5 Hz, 2H), 6.81 (d, J = 8.5 Hz, 1H), 2.28 (s, 3H) ppm; ^{13}C NMR (125 MHz, $CDCl_3$) δ 153.2 (s), 152.5 (s), 134.4 (s), 131.1 (s), 130.6 (d, 2C), 125.9 (q, J_{C-F} = 32.5 Hz), 123.6 (q, J_{C-F} = 270.0 Hz), 122.6 (q, J_{C-F} = 3.8 Hz), 119.1 (d, 2C), 118.6 (d), 118.3 (q, J_{C-F} = 3.8 Hz), 20.8 (q) ppm; ^{19}F NMR (471 MHz, $CDCl_3$) δ -62.08 ppm; IR (reflection) $\tilde{\nu}$ = 3063, 3039, 2963, 2904, 2868, 2127, 1651, 1588, 1508, 1490, 1452, 1364, 1295, 1268, 1234, 1172, 1096, 1013, 882, 831, 750 cm^{-1}; HRMS (EI) (m/z) [M] $C_{14}H_{10}F_3N_3O$ calcd for 293.0771, found 293.0779.

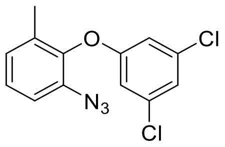

1-azido-2-(3,5-dichlorophenoxy)-3-methylbenzene (3d)

Yield: 1.00 g, 68%; brown solid, mp 71-72 ℃; R_f = 0.36 (PE); ^{1}H NMR (500 MHz, $CDCl_3$) δ 7.15-7.10 (m, 1H), 7.00-6.94 (m, 3H), 6.63-6.56 (m, 2H), 2.07 (s, 3H) ppm; ^{13}C NMR (125 MHz, $CDCl_3$) δ 158.7 (s), 142.9 (s), 135.8 (s, 2C), 133.4 (s), 133.3 (s), 127.8 (d), 126.7 (d), 122.6 (d), 118.2 (d), 113.9 (d, 2C), 16.1 (q) ppm; IR (reflection) $\tilde{\nu}$ = 3090, 2926, 2222, 2113, 1575, 1469, 1429, 1309, 1259, 1239, 1223, 1186, 1092, 942, 836, 799, 779, 750, 665, 614 cm^{-1}; HRMS (EI) (m/z) [M] $C_{13}H_9{}^{35}Cl_2N_3O$ calcd for 293.0117, found 293.0120.

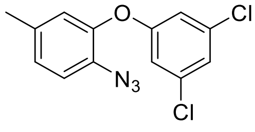

1-azido-2-(3,5-dichlorophenoxy)-4-methylbenzene (3e)

Yield: 840 mg, 57%; brown solid, mp 38-39 ℃; R_f = 0.34 (PE); ^{1}H NMR (500 MHz, $CDCl_3$) δ 7.01-6.95 (m, 3H), 6.77 (s, 1H), 6.72 (s, 2H), 2.26 (s, 3H) ppm; ^{13}C NMR (125 MHz, $CDCl_3$) δ 159.0 (s), 145.5 (s), 136.5 (s), 135.7 (s, 2C), 129.5 (s), 127.2 (d), 123.1 (d), 123.0 (d), 120.7 (d), 115.5 (d, 2C), 20.9 (q) ppm; IR (reflection) $\tilde{\nu}$ = 3085, 2923, 2858, 2400, 2130, 2095, 1667, 1575, 1503, 1435, 1312, 1245, 1162, 1098, 965, 910, 840, 806, 667 cm^{-1}; HRMS (EI) (m/z) [M] $C_{13}H_9Cl_2N_3O$ calcd for

293.0117, found 293.0127.

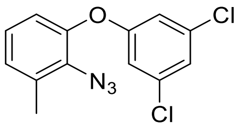

2-azido-1-(3,5-dichlorophenoxy)-3-methylbenzene (3f)

Yield: 440 mg, 30%; green solid, mp 67-68 ℃; R_f = 0.57 (PE); ^{1}H NMR (500 MHz, $CDCl_3$) δ 7.03 (s, 1H), 6.98-6.94 (m, 2H), 6.81-6.74 (m, 3H), 2.22 (s, 3H) ppm; ^{13}C NMR (125 MHz, $CDCl_3$) δ 158.7 (s), 148.3 (s), 135.8 (s, 2C), 132.9 (s), 130.6 (s), 127.6 (d), 125.3 (d), 123.5 (d), 119.2 (d), 115.7 (d, 2C), 18.0 (q) ppm; IR (reflection) $\tilde{\nu}$ = 3084, 2926, 2853, 2454,, 2108, 1576, 1466, 1434, 1377, 1326, 1300, 1283, 1250, 1217, 1132, 1098, 1078, 1033, 971, 908, 838, 810, 785, 744, 713, 663, 647 cm^{-1}; HRMS (EI) (*m/z*) [M] $C_{13}H_9{}^{35}Cl_2N_3O$ calcd for 293.0117, found 293.0113.

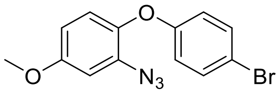

2-azido-1-(4-bromophenoxy)-4-methoxybenzene (3g)

Yield: 1.53 g, 96%; yellow oil; R_f = 0.50 (EA/PE = 1/20); ^{1}H NMR (300 MHz, $CDCl_3$) δ 7.33-7.24 (m, 2H), 6.88-6.82 (m, 1H), 6.71-6.63 (m, 2H), 6.61-6.53 (m, 2H), 3.71 (s, 3H) ppm; ^{13}C NMR (75 MHz, $CDCl_3$) δ 157.7 (s), 157.4 (s), 140.5 (s), 133.2 (s), 132.6 (d, 2C), 123.3 (d), 117.9 (d, 2C), 114.9 (s), 111.2 (d), 106.4 (d), 55.8 (q) ppm; IR (reflection) $\tilde{\nu}$ = 2958, 2934, 2909, 2835, 2114, 1606, 1591, 1581, 1500, 1479, 1330, 1212, 1163, 1091, 1069, 1035, 1006, 924, 821, 710, 654 cm^{-1}; HRMS (EI) (*m/z*) [M] $C_{13}H_{10}BrN_3O_2$ calcd for 318.9951, found 318.9941.

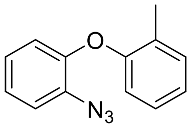

1-azido-2-(*o*-tolyloxy)benzene (3h)

Yield: 1.00 g, 89%; yellow solid, mp 36-37 ℃; R_f = 0.20 (PE); ^{1}H NMR (300 MHz, $CDCl_3$) δ 7.19-7.14 (m, 1H), 7.10-6.91 (m, 5H), 6.71 (dd, J = 0.9, 8.1 Hz, 1H), 6.68-6.63 (m, 1H), 2.21 (s, 3H) ppm; ^{13}C NMR (75 MHz, $CDCl_3$) δ 154.5 (s), 149.3 (s), 131.6 (d), 130.5 (s), 129.2 (s), 127.2 (d), 125.7 (d), 124.0 (d), 123.8 (d), 121.0 (d), 118.9 (d), 118.1 (d), 16.1 (q) ppm; IR (reflection) $\tilde{\nu}$ = 2926, 2136, 1946, 1908, 1579, 1488, 1453, 1296, 1271, 1227, 1182, 1116, 1096, 1048, 937, 879, 818, 781, 753, 653 cm^{-1}; HRMS (EI) (*m/z*) [M] $C_{13}H_{11}N_3O$ calcd for 225.0897, found 225.0903.

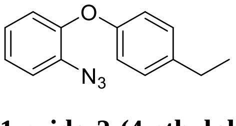

1-azido-2-(4-ethylphenoxy)benzene (3i)

Yield: 1.01 g, 85%; brown oil; R_f = 0.20 (PE); ^{1}H NMR (300 MHz, $CDCl_3$) δ 7.08-7.03 (m, 2H), 7.02-6.92 (m, 3H), 6.85-6.75 (m, 3H), 2.53 (q, J = 7.5 Hz, 2H), 1.14 (t, J = 7.5 Hz, 3H) ppm; ^{13}C NMR (75 MHz, $CDCl_3$) δ 155.0 (s), 148.9 (s), 139.4 (s), 131.4 (s), 129.1 (d, 2C), 125.7 (d), 124.5 (d), 120.9 (d), 120.6 (d), 117.8(d, 2C), 28.2 (t), 15.7 (q) ppm; IR (reflection) $\tilde{\nu}$ = 3062, 3032, 2965, 2931, 2872, 2121, 1653, 1588, 1506, 1491, 1452, 1314, 1300, 1235, 1201, 1166, 1097, 1016, 880, 831, 753, 659 cm^{-1}; HRMS (EI) (*m/z*) [M] $C_{14}H_{13}N_3O$ calcd for 239.1053, found 239.1050.

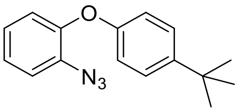

1-azido-2-(4-(*tert*-butyl)phenoxy)benzene (3j)

Yield: 1.28 g, 96%; yellow oil; R_f = 0.17 (PE); ^{1}H NMR (300 MHz, $CDCl_3$) δ 7.28-7.22 (m, 2H), 7.05-7.01 (m, 2H), 7.00-6.94 (m, 1H), 6.88-6.83 (m, 1H), 6.83-6.77 (m, 2H), 1.23 (s, 9H) ppm; ^{13}C NMR (75 MHz, $CDCl_3$) δ 154.8 (s), 148.8 (s), 146.2 (s), 131.5 (s), 126.6 (d, 2C), 125.7 (d), 124.5 (d), 120.9 (d), 120.7 (d), 117.3 (d, 2C), 34.3 (s), 31.5 (q, 3C) ppm; IR (reflection) $\tilde{\nu}$ = 3063, 3039, 2963, 2904, 2868, 2410, 2126, 1651, 1587, 1508, 1489, 1452, 1364, 1310, 1296, 1268, 1235, 1172, 1096, 1013, 882, 831, 750 cm^{-1}; HRMS (EI) (*m/z*) [M] $C_{16}H_{17}N_3O$ calcd for 267.1369, found 267.1366.

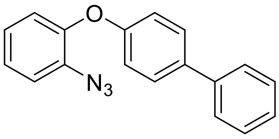

4-(2-azidophenoxy)-1,1'-biphenyl (3k)

Yield: 1.38 g, 96%; grey solid, mp 98-99 ℃; R_f = 0.20 (PE); ^{1}H NMR (300 MHz, $CDCl_3$) δ 7.51-7.45 (m, 4H), 7.39-7.32 (m, 2H), 7.29-7.22 (m, 1H), 7.11-7.01 (m, 3H), 6.98-6.91 (m, 3H) ppm; ^{13}C NMR (75 MHz, $CDCl_3$) δ 156.9 (s), 148.1 (s), 140.5 (s), 136.4 (s), 131.9 (s), 128.8 (d, 2C), 128.5 (d, 2C), 127.1 (d), 127.0 (d, 2C), 125.8 (d), 125.1 (d), 121.3 (d), 121.0 (d), 117.8 (d, 2C) ppm; IR (reflection) $\tilde{\nu}$ = 3056, 3040, 2450, 2123, 2091, 1584, 1518, 1486, 1450, 1406, 1308, 1272, 1238, 1195, 1172, 1142, 1095, 1037, 1003, 931, 877, 858, 837, 808, 758, 736, 689, 661, 639 cm^{-1}; HRMS (EI) (*m/z*) [M] $C_{18}H_{13}N_3O$ calcd for 287.1053, found 287.1046.

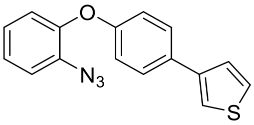

3-(4-(2-azidophenoxy)phenyl)thiophene (3l)

Yield: 1.41 g, 96%; grey solid, mp 136-137 ℃; R_f = 0.17 (PE); ^{1}H NMR (300 MHz, $CDCl_3$) δ 7.52-7.44 (m, 2H), 7.34-7.24 (m, 3H), 7.10-7.06 (m, 2H), 7.06-6.99 (m, 1H), 6.97-6.85 (m, 3H) ppm; ^{13}C NMR (75 MHz, $CDCl_3$) δ 156.5 (s), 148.1 (s), 141.6 (s), 131.8 (s), 131.3 (s), 127.9 (d, 2C), 126.3 (d), 126.3 (d), 125.8 (d), 125.0 (d), 121.2 (d), 121.0 (d), 119.8 (d), 117.9 (d, 2C) ppm; IR (reflection) $\tilde{\nu}$ = 3097, 3064, 2447, 2121, 2090, 1889, 1607, 1593, 1534, 1491, 1454, 1428, 1408, 1364, 1304, 1274, 1233, 1206, 1173, 1143, 1112, 1096, 1037, 1010, 940, 897, 876, 863, 829, 804, 788, 775, 755, 732, 710, 695, 658, 625 cm^{-1}; HRMS (EI) (*m/z*) [M] $C_{16}H_{11}N_3OS$ calcd for 293.0617, found 293.0620.

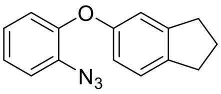

5-(2-azidophenoxy)-2,3-dihydro-1*H*-indene (3m)

Yield: 1.13 g, 90%; light yellow oil; R_f = 0.40 (PE); ^{1}H NMR (300 MHz, $CDCl_3$) δ 7.08 (d, J = 8.1 Hz, 1H), 7.05-7.01 (m, 2H), 7.00-6.93 (m, 1H), 6.88-6.81 (m, 1H), 6.77-6.73 (m, 1H), 6.68 (dd, J = 2.1, 7.8 Hz, 1H), 2.84-2.76 (m, 4H), 2.07-1.96 (m, 2H) ppm; ^{13}C NMR (75 MHz, $CDCl_3$) δ 155.8 (s), 149.1 (s), 146.2 (s), 139.2 (s), 131.3 (s), 125.7 (d), 125.1 (d), 124.3 (d), 120.9 (d), 120.5 (d),

115.9 (d), 114.1 (d), 33.1 (t), 32.1 (t), 25.8 (t) ppm; IR (reflection) $\tilde{\nu}$ = 3062, 3020, 2951, 2844, 2126, 2096, 1612, 1585, 1488, 1451, 1315, 1244, 1219, 1187, 1158, 1126, 1097, 1036, 942, 868, 814, 744, 660 cm^{-1}; HRMS (EI) (*m/z*) [M] $C_{15}H_{13}N_3O$ calcd for 251.1053, found 251.1045.

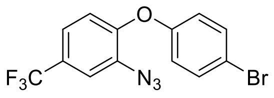

2-azido-1-(4-bromophenoxy)-4-(trifluoromethyl)benzene (3n)

Yield: 1.51 g, 85%; yellow oil; R_f = 0.37 (PE); ^{1}H NMR (500 MHz, $CDCl_3$) δ 7.41 (d, *J* = 8.0 Hz, 2H), 7.29 (s, 1H), 7.26 (d, *J* = 8.5 Hz, 1H), 6.89 (d, *J* = 8.5 Hz, 1H), 6.84-6.76 (m, 2H) ppm; ^{13}C NMR (125 MHz, $CDCl_3$) δ 155.1 (s), 151.0 (s), 133.1 (d, 2C), 131.9 (s), 127.1 (q, J_{C-F} = 33.5 Hz), 123.4 (q, J_{C-F} = 270.0 Hz), 122.8 (q, J_{C-F} = 3.8 Hz), 120.2 (d, 2C), 119.9 (d), 118.5 (q, J_{C-F} = 3.8 Hz), 117.1 (s) ppm; ^{19}F NMR (471 MHz, $CDCl_3$) δ -62.22 ppm; IR (reflection) $\tilde{\nu}$ = 3072, 2437, 2392, 2244, 2202, 2120, 1887, 1665, 1612, 1582, 1507, 1483, 1425, 1337, 1275, 1242, 1199, 1170, 1129, 1104, 1071, 1010, 903, 875, 843, 822, 693, 642 cm^{-1}; HRMS (EI) (*m/z*) [M] $C_{13}H_7BrF_3N_3O$ calcd for 356.9719, found 356.9712.

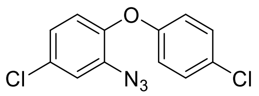

2-azido-4-chloro-1-(4-chlorophenoxy)benzene (3o)

Yield: 1.34 g, 96%; yellow solid, mp 36-37 ℃; R_f = 0.29 (PE); ^{1}H NMR (300 MHz, $CDCl_3$) δ 7.24-7.18 (m, 2H), 7.04 (d, *J* = 2.1 Hz, 1H), 6.99 (dd, *J* = 2.4, 8.7 Hz, 1H), 6.83-6.76 (m, 3H) ppm; ^{13}C NMR (75 MHz, $CDCl_3$) δ 155.7 (s), 146.4 (s), 133.0 (s), 130.3 (s), 129.9 (d, 2C), 128.7 (s), 125.8 (d), 122.1 (d), 121.1 (d), 118.7 (d, 2C) ppm; IR (reflection) $\tilde{\nu}$ = 2111, 1883, 1586, 1484, 1399, 1296, 1235, 1199, 1143, 1094, 899, 853, 823, 786, 683, 650 cm^{-1}; HRMS (EI) (*m/z*) [M] $C_{12}H_7{}^{35}Cl_2N_3O$ calcd for 278.9961, found 278.9963.

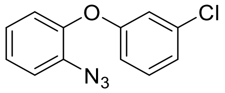

1-azido-2-(3-chlorophenoxy)benzene (3p)

Yield: 1.18 g, 96%; brown oil; R_f = 0.25 (PE); ^{1}H NMR (300 MHz, $CDCl_3$) δ 7.18-7.13 (m, 1H), 7.13-7.07 (m, 2H), 7.07-7.02 (m, 1H), 7.01-6.96 (m, 1H), 6.95-6.89 (m, 1H), 6.86-6.82 (m, 1H), 6.79-6.73 (m, 1H) ppm; ^{13}C NMR (75 MHz, $CDCl_3$) δ 158.3 (s), 147.0 (s), 135.2 (s), 132.1 (s), 130.6 (d), 125.9 (d), 125.7 (d), 123.3 (d), 121.9 (d), 121.0 (d), 117.5 (d), 115.4 (d) ppm; IR (reflection) $\tilde{\nu}$ = 3067, 2427, 2126, 1657, 1581, 1492, 1472, 1452, 1430, 1301, 1268, 1234, 1157, 1097, 1069, 1036, 998, 912, 860, 756, 679, 661 cm^{-1}; HRMS (EI) (*m/z*) [M] $C_{12}H_8{}^{35}ClN_3O$ calcd for 245.0350, found 245.0359.

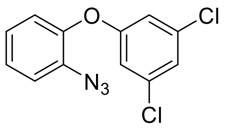

1-(2-azidophenoxy)-3,5-dichlorobenzene (3q)

Yield: 1.34 g, 96%; yellow solid, mp 51-52 ℃; R_f = 0.27 (PE); ^{1}H NMR (500 MHz, $CDCl_3$) δ 7.19-7.14 (m, 1H), 7.13-7.06 (m, 2H), 7.01-6.98 (m, 1H), 6.96 (d, *J* = 8.0 Hz, 1H), 6.72 (d, *J* = 1.5 Hz, 2H) ppm; ^{13}C NMR (125 MHz, $CDCl_3$) δ 158.8 (s), 145.8 (s), 135.7 (s, 2C), 132.4 (s), 126.5 (d),

126.1 (d), 123.2 (d), 122.5 (d), 121.0 (d), 115.6 (d, 2C) ppm; IR (reflection) $\tilde{\nu}$ = 3074, 2359, 2340, 2121, 1576, 1492, 1454, 1440, 1420, 1299, 1272, 1245, 1229, 1144, 1099, 947, 856, 835, 807, 759, 736, 666 cm^{-1}; HRMS (EI) (*m/z*) [M] $C_{12}H_7{}^{35}Cl_2N_3O$ calcd for 278.9961, found 278.9974.

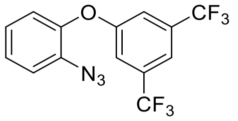

1-(2-azidophenoxy)-3,5-bis(trifluoromethyl)benzene (3r)

Yield: 1.59 g, 92%; yellow oil; R_f = 0.43 (PE); ^{1}H NMR (300 MHz, $CDCl_3$) δ 7.50 (s, 1H), 7.27-7.20 (m, 3H), 7.19-7.17 (m, 1H), 7.16-7.10 (m, 1H), 7.00 (dd, J = 1.2, 7.8 Hz, 1H) ppm; ^{13}C NMR (75 MHz, $CDCl_3$) δ 158.6 (s), 145.2 (s), 133.2 (q, J_{C-F} = 33.8 Hz, 2C), 132.5 (s), 126.9 (d), 126.3 (d), 122.5 (d), 122.9 (q, J_{C-F} = 271.3 Hz, 2C), 121.0 (d), 116.73-116.60 (m, 2C), 116.4 (hept, J_{C-F} = 3.8 Hz) ppm; ^{19}F NMR (471 MHz, $CDCl_3$) δ -62.98 ppm; IR (reflection) $\tilde{\nu}$ = 3071, 2126, 1690, 1615, 1597, 1494, 1461, 1370, 1309, 1277, 1235, 1173, 1132, 950, 881, 850, 759, 701, 682 cm^{-1}; HRMS (EI) (*m/z*) [M] $C_{14}H_7N_3OF_6$ calcd for 347.0488, found 347.0490.

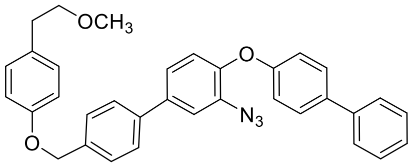

4-([1,1'-biphenyl]-4-yloxy)-3-azido-4'-((4-(2-methoxyethyl)phenoxy)methyl)-1,1'-biphenyl (3s)

Yield (1.0 mmol): 411 mg, 78%; white solid, mp 115-116 ℃; R_f = 0.27 (EA/PE = 1/10); ^{1}H NMR (300 MHz, $CDCl_3$) δ 7.53-7.46 (m, 6H), 7.45-7.40 (m, 2H), 7.39-7.31 (m, 2H), 7.29-7.22 (m, 3H), 7.07 (d, J = 8.7 Hz, 2H), 7.02-6.96 (m, 3H), 6.85 (d, J = 8.7 Hz, 2H), 5.01 (s, 2H), 3.49 (t, J = 7.2 Hz, 2H), 3.27 (s, 3H), 2.75 (t, J = 6.9 Hz, 2H) ppm; ^{13}C NMR (75 MHz, $CDCl_3$) δ 157.3 (s), 156.8 (s), 147.6 (s), 140.5 (s), 139.3 (s), 138.0 (s), 136.8 (s), 136.6 (s), 132.0 (s), 131.5 (s), 129.9 (d, 2C), 128.8 (d, 2C), 128.6 (d, 2C), 128.1 (d, 2C), 127.2 (d, 2C), 127.1 (d), 127.0 (d, 2C), 124.4 (d), 121.4 (d), 119.5 (d), 117.9 (d, 2C), 114.8 (d, 2C), 73.9 (t), 69.7 (t), 58.7 (q), 35.3 (t) ppm; IR (reflection) $\tilde{\nu}$ = 3037, 2981, 2932, 2858, 2119, 1609, 1511, 1484, 1458, 1394, 1319, 1239, 1167, 1112, 1099, 1052, 1015, 1005, 966, 898, 884, 832, 818, 782, 764, 732, 713, 698, 673 cm^{-1}; HRMS (EI) (*m/z*) [M] $C_{34}H_{29}N_3O_3$ calcd for 527.2203, found 527.2203.

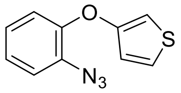

3-(2-azidophenoxy)thiophene (3t)

Yield (1.0 mmol): 195 mg, 90%; pale brown oil; ^{1}H NMR (400 MHz, $CDCl_3$) δ 7.27-7.25 (m, 1H), 7.14-7.06 (m, 3H), 7.05-7.01 (m, 1H), 6.86 (dd, J = 5.2, 1.2 Hz, 1H), 6.52 (dd, J = 3.2, 1.6 Hz, 1H) ppm; ^{13}C NMR (101 MHz, $CDCl_3$) 154.3 (s), 149.4 (s), 130.8 (s), 125.7 (d), 125.5 (d), 124.7 (d), 120.9 (d), 120.1 (d), 119.8 (d), 105.8 (d) ppm; HRMS (EI) (*m/z*) [M] $C_{10}H_7N_3OS$ calcd for 217.0310, found 217.0310.

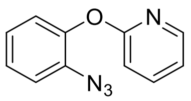

2-(2-azidophenoxy)pyridine (3u)

Yield (1.0 mmol): 188 mg, 89%; pale brown oil; ^{1}H NMR (400 MHz, $CDCl_3$) δ 8.21-8.18 (m, 1H), 7.77-7.71 (m, 1H), 7.30-7.25 (m, 1H), 7.24-7.17 (m, 3H), 7.06-6.99 (m, 2H) ppm; ^{13}C NMR (101

MHz, $CDCl_3$) δ 163.4 (s), 147.7 (d), 145.3 (s), 139.7 (d), 132.8 (s), 126.3 (d), 125.8 (d), 124.0 (d), 120.7 (d), 118.8 (d), 111.2 (d) ppm; HRMS (EI) (*m/z*) [M] $C_{11}H_8N_4O$ calcd for 212.0698, found 212.0698.

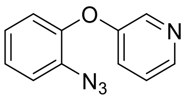

3-(2-azidophenoxy)pyridine (3v)

Yield (1.0 mmol): 202 mg, 95%; pale brown oil; ^{1}H NMR (400 MHz, $CDCl_3$) δ 8.39-8.31 (m, 2H), 7.30-7.18 (m, 4H), 7.17-7.11 (m, 1H), 7.02-6.97 (m, 1H) ppm; ^{13}C NMR (101 MHz, $CDCl_3$) 154.0 (s), 146.8 (s), 144.2 (d), 139.9 (d), 132.0 (s), 126.0 (d), 125.9 (d), 124.21 (d), 124.17 (d), 121.4 (d), 121.0 (d) ppm; HRMS (EI) (*m/z*) [M] $C_{11}H_8N_4O$ calcd for 212.0698, found 212.0700.

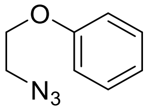

(2-azidoethoxy)benzene (3w)

Yield (3.0 mmol): 465 mg, 95%; pale yellow oil; ^{1}H NMR (300 MHz, $CDCl_3$) δ 7.28-7.19 (m, 2H), 6.96-6.81 (m, 3H), 4.09 (t, *J* = 5.1 Hz, 2H), 3.52 (t, *J* = 5.1 Hz, 2H) ppm; ^{13}C NMR (75 MHz, $CDCl_3$) 158.2 (s), 129.6 (d, 2C), 121.4 (d), 114.6 (d, 2C), 66.9 (t), 50.2 (t) ppm; HRMS (EI) (*m/z*) [M] $C_8H_9N_3O$ calcd for 163.0746, found 163.0746.

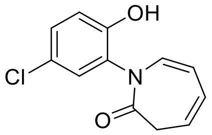

1-(5-chloro-2-hydroxyphenyl)-1,3-dihydro-2*H*-azepin-2-one (5a)

Yield: 38 mg, 80%; white solid, mp 156-157 ℃; R_f = 0.25 (EA/PE = 1/3); ^{1}H NMR (300 MHz, $CDCl_3$) δ 7.09 (dd, *J* = 2.4, 8.7 Hz, 1H), 6.97 (d, *J* = 2.4 Hz, 1H), 6.85 (d, *J* = 8.7 Hz, 1H), 6.59 (brs, 1H), 6.29-6.23 (m, 1H), 6.21 (d, *J* = 8.7 Hz, 1H), 6.07-5.99 (m, 1H), 5.80-5.70 (m, 1H), 3.08 (d, *J* = 6.9 Hz, 2H) ppm; ^{13}C NMR (75 MHz, $CDCl_3$) δ 166.1 (s), 148.9 (s), 129.6 (d), 129.1 (s), 128.2 (d), 126.4 (d), 126.2 (d), 124.5 (s), 122.7 (d), 119.6 (d), 116.7 (d), 36.5 (t) ppm; IR (ATR) $\tilde{\nu}$ = 3317, 3076, 1645, 1592, 1504, 1462, 1417, 1391, 1334, 1286, 1181, 1166, 1133, 1110, 1036, 1015, 977, 958, 884, 871, 853, 808, 774, 751, 702, 653, 612 cm^{-1}; HRMS (ESI) (*m/z*) $[M+Na]^+$ $C_{12}H_{10}NNaO_2{}^{35}Cl$ calcd for 258.0292, found 258.0297.

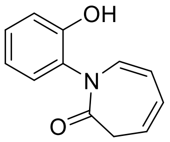

1-(2-hydroxyphenyl)-1,3-dihydro-2*H*-azepin-2-one (5b)

Yield: 32 mg, 80%; brown solid, mp 165-167 ℃; R_f = 0.25 (EA/PE = 1/3); ^{1}H NMR (500 MHz, $CDCl_3$) δ 7.17-7.12 (m, 1H), 6.98 (dd, *J* = 1.5, 8.0 Hz, 1H), 6.94 (dd, *J* = 1.0, 8.0 Hz, 1H), 6.90-6.84 (m, 1H), 6.45 (brs, 1H), 6.28-6.21 (m, 2H), 6.02-5.96 (m, 1H), 5.78-5.71 (m, 1H), 3.09 (d, *J* = 7.0 Hz, 2H) ppm; ^{13}C NMR (125 MHz, $CDCl_3$) δ 167.0 (s), 151.0 (s), 131.2 (d), 129.4 (s), 129.4 (d), 127.6 (d), 127.2 (d), 123.6 (d), 121.2 (d), 119.5 (d), 117.1 (d), 37.6 (t) ppm; IR (ATR) $\tilde{\nu}$ = 3246, 3040, 2988, 1627, 1598, 1506, 1453, 1428, 1391, 1335, 1281, 1194, 1168, 1133, 1095, 1034, 1013, 974, 948, 870, 830, 783, 757, 699, 682, 616 cm^{-1}; HRMS (ESI) (*m/z*) $[M+Na]^+$ $C_{12}H_{11}NNaO_2$ calcd

for 224.0682, found 224.0683.

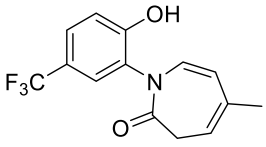

1-(2-hydroxy-5-(trifluoromethyl)phenyl)-5-methyl-1,3-dihydro-2H-azepin-2-one (5c)

Yield: 40 mg, 71%; white solid, mp 170-172 ℃; R_f = 0.26 (EA/PE = 1/3); ^{1}H NMR (300 MHz, $CDCl_3$) δ 7.34 (dd, *J* = 2.1, 8.4 Hz, 1H), 7.24-7.20 (m, 1H), 6.93 (d, *J* = 8.7 Hz, 1H), 6.13 (d, *J* = 9.0 Hz, 1H), 5.93 (d, *J* = 8.7 Hz, 1H), 5.52-5.40 (m, 1H), 3.01 (d, *J* = 6.6 Hz, 2H), 1.89 (s, 3H) ppm; ^{13}C NMR (75 MHz, $CDCl_3$) δ 168.2 (s), 154.2 (s), 135.7 (s), 129.2 (d), 129.0 (s), 126.3 (q, $J_{C\text{-}F}$ = 3.8 Hz), 125.1 (q, $J_{C\text{-}F}$ = 3.8 Hz), 123.8 (q, $J_{C\text{-}F}$ = 269.3 Hz), 123.1 (q, $J_{C\text{-}F}$ = 33.8 Hz), 121.0 (d), 119.5 (d), 118.9 (d), 37.1 (t), 20.5 (q) ppm; ^{19}F NMR (283 MHz, $CDCl_3$) δ -61.48 ppm; IR (ATR) $\tilde{\nu}$ = 3287, 1655, 1619, 1591, 1523, 1473, 1443, 1328, 1300, 1267, 1201, 1160, 1112, 1073, 1035, 954, 891, 834, 781, 702, 665, 631, 616 cm^{-1}; HRMS (ESI) (*m/z*) $[M+H]^+$ $C_{14}H_{13}F_3NO_2$ calcd for 284.0893, found 284.0892.

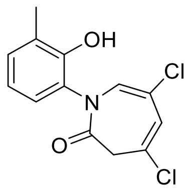

4,6-dichloro-1-(2-hydroxy-3-methylphenyl)-1,3-dihydro-2*H*-azepin-2-one (5d)

Yield: 37 mg, 65%; white solid, mp 138-140 ℃; R_f = 0.63 (EA/PE = 1/3); ^{1}H NMR (500 MHz, $CDCl_3$) δ 7.12-7.05 (m, 1H), 6.85-6.78 (m, 2H), 6.54-6.28 (m, 2H), 5.81 (s, 1H), 3.44 (s, 2H), 2.20 (s, 3H) ppm; ^{13}C NMR (125 MHz, $CDCl_3$) δ 163.8 (s), 149.0 (s), 131.2 (d), 128.9 (d), 128.8 (s), 128.2 (s), 127.6 (s), 125.16 (d), 125.15 (d), 122.0 (s), 121.2 (d), 45.7 (t), 16.2 (q) ppm; IR (reflection) $\tilde{\nu}$ = 3131, 2924, 1650, 1621, 1597, 1474, 1421, 1385, 1332, 1300, 1249, 1206, 1171, 1081, 1039, 959, 895, 839, 822, 7937, 775, 732, 707, 674, 618 cm^{-1}; HRMS (DART) (*m/z*) $[M+H]^+$ $C_{13}H_{12}{}^{35}Cl_2NO_2$ calcd for 284.0240, found 284.0237.

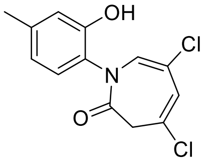

4,6-dichloro-1-(2-hydroxy-4-methylphenyl)-1,3-dihydro-2*H*-azepin-2-one (5e)

Yield: 38 mg, 67%; white solid, mp 138-139 ℃; R_f = 0.43 (EA/PE = 1/3); ^{1}H NMR (300 MHz, $CDCl_3$) δ 6.82 (d, *J* = 8.1 Hz, 1H), 6.66 (d, *J* = 8.1 Hz, 1H), 6.61 (s, 1H), 6.54 (s, 1H), 6.45-6.34 (m, 2H), 3.40 (s, 2H), 2.17 (s, 3H) ppm; ^{13}C NMR (75 MHz, $CDCl_3$) δ 163.8 (s), 150.6 (s), 140.5 (s), 129.1 (d), 127.7 (d), 127.2 (s), 125.23 (s), 125.19 (d), 121.9 (d), 121.4 (s), 119.4 (d), 45.7 (t), 21.1 (q) ppm; IR (reflection) $\tilde{\nu}$ = 3253, 3069, 2908, 1731, 1655, 1598, 1522, 1424, 1387, 1296, 1261, 1198, 1170, 1152, 1116, 1036, 976, 956, 929, 876, 846, 815, 788, 755, 739, 719, 673, 619 cm^{-1}; HRMS (DART) (*m/z*) $[M+H]^+$ $C_{13}H_{12}{}^{5}Cl_2NO_2$ calcd for 284.0240, found 284.0238.

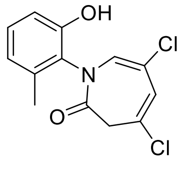

4,6-dichloro-1-(2-hydroxy-3-methylphenyl)-1,3-dihydro-2*H*-azepin-2-one (5f)

Yield: 35 mg, 61%; grey solid, mp 159-160 ℃; R_f = 0.60 (EA/PE = 1/1); ^{1}H NMR (500 MHz, $CDCl_3$) δ 7.15-6.73 (m, 2H), 6.67 (d, *J* = 7.5 Hz, 1H), 6.56 (d, *J* = 8.0 Hz, 1H), 6.37 (s, 1H), 6.31 (s, 1H), 3.41-3.26 (m, 2H), 1.92 (s, 3H) ppm; ^{13}C NMR (125 MHz, $CDCl_3$) δ 164.6 (s), 151.6 (s), 137.6 (s), 129.8 (d), 128.6 (d), 127.2 (s), 125.6 (s), 125.1 (d), 122.2 (d), 121.2 (s), 114.9 (d), 45.4 (t), 17.0 (q) ppm; IR (reflection) $\tilde{\nu}$ = 3308, 1657, 1620, 1590, 1473, 1418, 1383, 1340, 1295, 1268, 1202, 1151, 1034, 956, 888, 843, 782, 737, 702, 666, 616 cm^{-1}; HRMS (DART) (*m/z*) $[M+H]^+$ $C_{13}H_{12}{}^{35}Cl_2NO_2$ calcd for 284.0240, found 284.0237.

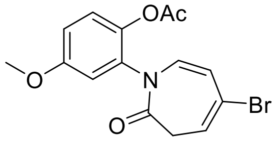

2-(4-bromo-2-oxo-2,3-dihydro-1*H*-azepin-1-yl)-4-methoxyphenyl acetate (5g')

Yield: 42 mg, 60%; white solid, mp 101-102 ℃; R_f = 0.45 (EA/PE = 1/1); ^{1}H NMR (300 MHz, $CDCl_3$) δ 7.05-7.00 (m, 1H), 6.88-6.82 (m, 1H), 6.71 (d, *J* = 2.7 Hz, 1H), 6.09 (d, *J* = 9.0 Hz, 1H), 5.95 (t, *J* = 7.5 Hz, 1H), 5.88 (d, *J* = 9.0 Hz, 1H), 3.73 (s, 3H), 3.11-2.88 (m, 2H), 2.16 (s, 3H) ppm; ^{13}C NMR (75 MHz, $CDCl_3$) δ 169.1 (s), 165.1 (s), 157.8 (s), 139.9 (s), 133.4 (s), 131.6 (d), 124.1 (d), 122.7 (d), 118.9 (s), 116.2 (d), 115.1 (d), 113.9 (d), 55.7 (q), 38.0 (t), 20.6 (q) ppm; IR (reflection) $\tilde{\nu}$ = 2935, 2834, 1759, 1679, 1593, 1507, 1438, 1370, 1339, 1246, 1209, 1183, 1168, 1134, 1033, 980, 900, 883, 866, 852, 820, 789, 772, 730, 638 cm^{-1}; HRMS (EI) (*m/z*) [M] $C_{15}H_{14}BrNO_4$ calcd for 351.0101, found. 351.0099.

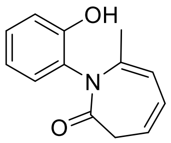

1-(2-hydroxyphenyl)-7-methyl-1,3-dihydro-2*H*-azepin-2-one (5h)

Yield: 20 mg, 47%; brown solid, mp 190-192 ℃; R_f = 0.14 (EA/PE = 1/3); ^{1}H NMR (500 MHz, $CDCl_3$) δ 7.17-7.12 (m, 1H), 6.94 (d, *J* = 7.5 Hz, 1H), 6.91-6.82 (m, 2H), 6.24 (s, 1H), 6.19-6.12 (m, 1H), 6.03-5.98 (m, 1H), 5.80-5.71 (m, 1H), 3.27-2.83 (m, 2H), 1.72 (s, 3H) ppm; ^{13}C NMR (125 MHz, $CDCl_3$) δ 169.0 (s), 152.1 (s), 138.8 (s), 129.3 (d), 128.0 (s), 127.7 (d), 127.2 (d), 123.9 (d), 121.2 (d), 119.2 (d), 119.1 (d), 37.3 (t), 22.0 (q) ppm; IR (ATR) $\tilde{\nu}$ = 2954, 2923, 1637, 1621, 1601, 1511, 1460, 1423, 1392, 1362, 1332, 1288, 1258, 1219, 1206, 1146, 1108, 997, 944, 866, 828, 797, 761, 736, 692, 666, 616 cm^{-1}; HRMS (ESI) (*m/z*) $[M+Na]^+$ $C_{13}H_{13}NNaO_2$ calcd for 238.0838, found 238.0844.

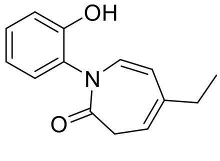

5-ethyl-1-(2-hydroxyphenyl)-1,3-dihydro-2*H*-azepin-2-one (5i)

Yield: 20 mg, 44 %; white solid, mp 164-166 ℃; R_f = 0.51 (EA/PE = 1/3); ^{1}H NMR (500 MHz, Acetone-d_6) δ 8.30 (s, 1H), 7.24-7.16 (m, 1H), 7.04-6.94 (m, 2H), 6.92-6.83 (m, 1H), 6.33 (d, *J* = 8.5 Hz, 1H), 5.88 (d, *J* = 9.0 Hz, 1H), 5.52 (t, *J* = 6.5 Hz, 1H), 2.99 (d, *J* = 6.5 Hz, 2H), 2.26 (q, *J* = 7.5 Hz, 2H), 1.10 (t, *J* = 7.5 Hz, 3H) ppm; ^{13}C NMR (125 MHz, Acetone-d_6) δ 166.4 (s), 152.4 (s), 141.6 (s), 131.3 (d), 129.3 (d), 129.2 (s), 128.9 (d), 119.8 (d), 117.1 (d), 116.5 (d), 116.1 (d), 37.1

(t), 27.8 (t), 13.3 (q) ppm; IR (ATR) $\tilde{\nu}$ = 3048, 2967, 2901, 2840, 2732, 2575, 1631, 1600, 1508, 1456, 1431, 1372, 1342, 1301, 1285, 1206, 1190, 1160, 1123, 1103, 1087, 1040, 980, 967, 944, 895, 864, 840, 787, 766, 755, 740, 726, 680, 625 cm^{-1}; HRMS (ESI) (*m/z*) $[M+Na]^+$ $C_{14}H_{15}NNaO_2$ calcd for 252.0995, found 252.0997.

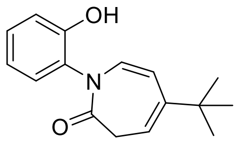

5-(tert-butyl)-1-(2-hydroxyphenyl)-1,3-dihydro-2*H*-azepin-2-one (5j)

Yield: 30 mg, 58%; white solid, mp 141-143 ℃; R_f = 0.19 (EA/PE = 1/5); ^{1}H NMR (500 MHz, $CDCl_3$) δ 7.20-7.15 (m, 1H), 6.99 (d, *J* = 8.5 Hz, 1H), 6.93-6.86 (m, 2H), 6.28-6.19 (m, 2H), 5.55 (t, *J* = 7.0 Hz, 1H), 3.01 (d, *J* = 6.5 Hz, 2H), 1.10 (s, 9H) ppm; ^{13}C NMR (125 MHz, $CDCl_3$) δ 168.8 (s), 150.9 (s), 148.2 (s), 130.6 (d), 129.3 (s), 129.2 (d), 127.2 (d), 121.3 (d), 120.0 (d), 119.1 (d), 116.0 (d), 37.2 (t), 34.6 (s), 29.6 (q, 3C) ppm; IR (ATR) $\tilde{\nu}$ = 3228, 3073, 2963, 2904, 2867, 1644, 1599, 1510, 1457, 1425, 1356, 1299, 1287, 1276, 1195, 1181, 1152, 1104, 1077, 1036, 973, 934, 897, 858, 845, 826, 776, 751, 675, 649 cm^{-1}; HRMS (ESI) (*m/z*) $[M+Na]^+$ $C_{16}H_{19}NNaO_2$ calcd for 280.1308, found 280.1312.

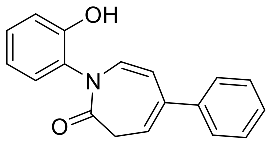

1-(2-hydroxyphenyl)-5-phenyl-1,3-dihydro-2*H*-azepin-2-one (5k)

Yield: 29 mg, 53%; white solid, mp 218-219 ℃; R_f = 0.22 (EA/PE = 1/5); ^{1}H NMR (300 MHz, THF-d_8) δ 8.31 (s, 1H), 7.50-7.44 (m, 2H), 7.36-7.23 (m, 3H), 7.16-7.09 (m, 1H), 7.00 (dd, *J* = 1.8, 7.8 Hz, 1H), 6.87 (dd, *J* = 1.2, 8.1 Hz, 1H), 6.83-6.76 (m, 1H), 6.52 (d, *J* = 9.0 Hz, 1H), 6.10 (d, *J* = 9.3 Hz, 1H), 6.05 (t, *J* = 7.2 Hz, 1H), 3.17 (d, *J* = 7.2 Hz, 2H) ppm; ^{13}C NMR (75 MHz, THF-d_8) δ 165.6 (s), 152.5 (s), 139.4 (s), 139.2 (s), 133.0 (d), 129.3 (d), 129.2 (s), 128.5 (d), 128.1 (d, 2C), 127.3 (d), 126.6 (d, 2C), 119.2 (d), 118.2 (d), 116.8 (d), 113.6 (d), 37.7 (t) ppm; IR (reflection) $\tilde{\nu}$ = 3054, 2957, 2892, 2749, 2586, 1630, 1600, 1509, 1458, 1434, 1416, 1372, 1295, 1284, 1212, 1187, 1174, 1157, 1143, 1106, 1077, 1060, 107, 966, 945, 891, 870, 838, 756, 740, 729, 698, 647, 614 cm^{-1}; HRMS (ESI) (*m/z*) $[M+H]^+$ $C_{18}H_{16}NO_2$ calcd for 278.1176, found 278.1173.

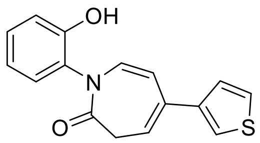

1-(2-hydroxyphenyl)-5-(thiophen-3-yl)-1,3-dihydro-2*H*-azepin-2-one (5l)

Yield: 23 mg, 40%; white solid, mp 186-187 ℃; R_f = 0.29 (EA/PE =1/5); ^{1}H NMR (300 MHz, $CDCl_3$) δ 7.30-7.25 (m, 1H), 7.23-7.15 (m, 3H), 7.03-6.95 (m, 2H), 6.93-6.85 (m, 1H), 6.44-6.38 (m, 1H), 6.36-6.16 (m, 2H), 6.04 (t, *J* = 6.9 Hz, 1H), 3.21 (d, *J* = 6.9 Hz, 2H) ppm; ^{13}C NMR (75 MHz, $CDCl_3$) δ 167.7 (s), 150.9 (s), 139.6 (s), 134.0 (s), 131.9 (d), 129.3 (d), 129.2 (s), 127.4 (d), 126.3 (d), 125.7 (d), 121.5 (d), 121.3 (d), 119.8 (d), 118.4 (d), 117.6 (d), 37.5 (t) ppm; IR (reflection) $\tilde{\nu}$ = 3096, 2958, 2890, 2748, 1628, 1602, 1591, 1509, 1459, 1416, 1390, 1358, 1295, 1282, 1213, 1178, 1157, 1141, 1106, 1057, 1038, 966, 945, 903, 872, 859, 834, 782, 758, 740, 701, 644, 620, 608 cm^{-1}; HRMS (ESI) (*m/z*) $[M+H]^+$ $C_{16}H_{14}NO_2S$ calcd for 284.0740, found 284.0743.

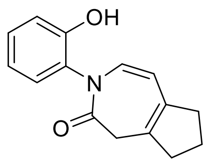

2-(2-hydroxyphenyl)-4,6,7,8-tetrahydrocyclopenta[*c*]azepin-3(2*H*)-one (5m)

Yield: 26 mg, 54%; white solid, mp 201-202 ℃; R_f = 0.30 (EA/PE =1/5); ^{1}H NMR (300 MHz, THF-d_8) δ 8.10 (s, 1H), 7.13-7.05 (m, 1H), 6.93 (dd, *J* = 1.8, 7.8 Hz, 1H), 6.84 (dd, *J* = 1.2, 8.1 Hz, 1H), 6.80-6.74 (m, 1H), 6.13 (d, *J* = 9.0 Hz, 1H), 5.75 (d, *J* = 8.7 Hz, 1H), 3.07 (s, 2H), 2.56-2.49 (m, 4H), 2.08-1.95 (m, 2H) ppm; ^{13}C NMR (75 MHz, THF-d_8) δ 164.5 (s), 152.4 (s), 135.0 (s), 134.0 (s), 130.1 (s), 129.5 (d), 129.1 (d), 128.2 (d), 119.1 (d), 116.8 (d), 111.5 (d), 38.1 (t), 36.4 (t), 33.7 (t), 23.5 (t) ppm; IR (reflection) $\tilde{\nu}$ = 3094, 2955, 2887, 2854, 2728, 2571, 1625, 1602, 1509, 1460, 1370, 1328, 1281, 1222, 1203, 1160, 1146, 1124, 1094, 1039, 956, 918, 862, 827, 755, 626 cm^{-1}; HRMS (ESI) (*m/z*) $[M+H]^+$ $C_{15}H_{16}NO_2$ calcd for 242.1176, found 242.1175.

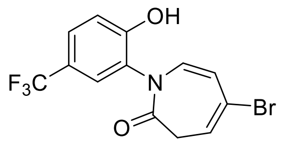

5-bromo-1-(2-hydroxy-5-(trifluoromethyl)phenyl)-1,3-dihydro-2*H*-azepin-2-one (5n)

Yield: 39 mg, 56%; grey solid, mp 173-175 ℃; R_f = 0.23 (EA/PE = 1/3); ^{1}H NMR (500 MHz, $CDCl_3$) δ 7.51 (s, 1H), 7.30 (d, *J* = 8.0 Hz, 1H), 7.24 (s, 1H), 6.81 (d, *J* = 8.5 Hz, 1H), 6.15-6.01 (m, 2H), 5.98 (t, *J* = 7.0 Hz, 1H), 3.06 (d, *J* = 6.5 Hz, 2H) ppm; ^{13}C NMR (125 MHz, $CDCl_3$) δ 166.8 (s), 154.3 (s), 131.4 (d), 128.1 (q, $J_{C\text{-}F}$ = 3.8 Hz), 127.0 (q, $J_{C\text{-}F}$ = 3.8 Hz), 125.9 (d), 123.7 (q, $J_{C\text{-}F}$ = 270.0 Hz), 123.1 (d), 123.1 (q, $J_{C\text{-}F}$ = 33.8 Hz), 119.1 (s), 118.7 (d), 118.4 (d), 38.0 (t) ppm; ^{19}F NMR (471 MHz, $CDCl_3$) δ -61.47 ppm; IR (reflection) $\tilde{\nu}$ = 3093, 2961, 2899, 2822, 2757, 1644, 1617, 1524, 1455, 1430, 1363, 1326, 1292, 1266, 1209, 1173, 1146, 1104, 1074, 1052, 988, 955, 883, 834, 763, 731, 658, 627, 614 cm^{-1}; HRMS (DART) (*m/z*) $[M+H]^+$ $C_{13}H_{10}BrF_3NO_2$ calcd for 347.9842, found 347.9836.

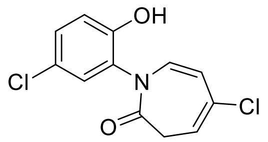

1-(5-chloro-2-hydroxyphenyl)-1,3-dihydro-2*H*-azepin-2-one (5o)

Yield: 32 mg, 67%; white solid, mp 48-50 ℃; R_f = 0.28 (EA/PE = 1/3); ^{1}H NMR (300 MHz, CD_2Cl_2) δ 7.13 (dd, *J* = 8.7, 2.7 Hz, 1H), 7.02 (d, *J* = 2.7 Hz, 1H), 6.83 (d, *J* = 8.7 Hz, 1H), 6.30-6.21 (m, 2H), 5.93 (d, *J* = 9.0 Hz, 1H), 5.79 (dt, *J* = 0.6, 7.5 Hz, 1H), 3.06 (d, *J* = 7.2 Hz, 2H) ppm; ^{13}C NMR (75 MHz, CD_2Cl_2) δ 166.8 (s), 150.0 (s), 131.6 (d), 130.6 (s), 129.5 (s), 129.4 (d), 127.7 (d), 125.3 (s), 119.9 (d), 119.7 (d), 116.4 (d), 36.7 (t) ppm; IR (ATR) $\tilde{\nu}$ = 3081, 2955, 1732, 1647, 1594, 1504, 1420, 1355, 1291, 1272, 1214, 1177, 1117, 1056, 1003, 958, 887, 852, 822, 774, 748, 728, 664, 648, 624 cm^{-1}; HRMS (ESI) (*m/z*) $[M+Na]^+$ $C_{12}H_9{}^{35}Cl_2NNaO_2$ calcd for 291.9903, found 291.9907.

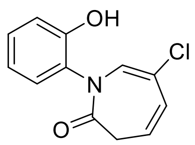

6-chloro-1-(2-hydroxyphenyl)-1,3-dihydro-2H-azepin-2-one (5p)

Yield: 26 mg, 55%; grey solid, mp 179-180 ℃; R_f = 0.25 (EA/PE = 1/3); ^{1}H NMR (500 MHz, Acetone-d_6) δ 8.61 (s, 1H), 7.26-7.19 (m, 1H), 7.06 (d, J = 7.5 Hz, 1H), 7.00 (d, J = 8.0 Hz, 1H), 6.94-6.86 (m, 1H), 6.62 (s, 1H), 6.37 (d, J = 9.5 Hz, 1H), 6.03-5.92 (m, 1H), 3.16 (d, J = 6.5 Hz, 2H) ppm; ^{13}C NMR (125 MHz, Acetone-d_6) δ 165.2 (s), 152.5 (s), 130.0 (d), 129.5 (d), 129.44 (d), 128.41 (d), 128.40 (s), 125.3 (d), 120.4 (s), 119.9 (d), 116.9 (d), 37.5 (t) ppm; IR (ATR) $\tilde{\nu}$ = 3168, 1643, 1624, 1591, 1511, 1455, 1422, 1371, 1287, 1230, 1197, 1147, 1106, 1046, 1032, 996, 963, 949, 850, 832, 780, 749, 708, 633, 607cm^{-1}; HRMS (ESI) (m/z) [M+Na]$^+$ $C_{12}H_{10}{}^{35}ClNNaO_2$ calcd for 258.0292, found 258.0294.

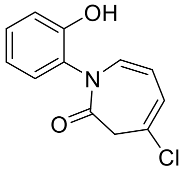

4-chloro-1-(2-hydroxyphenyl)-1,3-dihydro-2*H*-azepin-2-one (5p')

Yield: 17 mg, 36%; brown solid, mp 198-200 ℃; R_f = 0.31 (EA/PE = 1/3); ^{1}H NMR (500 MHz, Acetone-d_6) δ 8.41 (s, 1H), 7.12-7.05 (m, 1H), 6.91 (d, J = 7.5 Hz, 1H), 6.78-6.71 (m, 1H), 6.78-6.71 (m, 1H), 6.34 (d, J = 5.5 Hz, 1H), 6.24 (d, J = 9.0 Hz, 1H), 5.76-5.67 (m, 1H), 3.26 (s, 2H) ppm; ^{13}C NMR (125 MHz, Acetone-d_6) δ 162.9 (s), 152.4 (s), 131.8 (d), 129.4 (d), 129.3 (s), 128.8 (d), 124.3 (d), 122.5 (s), 119.9 (d), 116.9 (d), 111.7 (d), 45.8 (t) ppm; IR (ATR) $\tilde{\nu}$ = 3183, 2731, 2568, 1642, 1603, 1513, 1462, 1424, 1366, 1304, 1281, 1186, 1161, 1135, 1099, 1040, 1009, 966, 942, 881, 863, 828, 781, 755, 724, 699, 630, 609 cm^{-1}; HRMS (ESI) (m/z) [M+Na]$^+$ $C_{12}H_{10}{}^{35}ClNNaO_2$ calcd for 258.0292, found 258.0294.

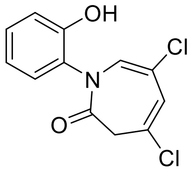

4,6-dichloro-1-(2-hydroxyphenyl)-1,3-dihydro-2*H*-azepin-2-one (5q)

Yield: 46 mg, 85%; white solid, mp 111-113 ℃; R_f = 0.34 (EA/PE = 1/3); ^{1}H NMR (500 MHz, $CDCl_3$) δ 7.17-7.11 (m, 1H), 6.96 (d, J = 8.0 Hz, 1H), 6.91-6.83 (m, 2H), 6.46 (s, 1H), 6.38 (s, 1H), 3.44 (s, 2H) ppm; ^{13}C NMR (125 MHz, $CDCl_3$) δ 163.8 (s), 151.0 (s), 130.1 (d), 128.9 (d), 128.0 (d), 127.9 (s), 127.3 (s), 125.2 (d), 121.8 (s), 121.3 (d), 119.1 (d), 45.7 (t) ppm; IR (ATR) $\tilde{\nu}$ = 3184, 3063, 2912, 1648, 1593, 1514, 1459, 1385, 1348, 1300, 1274, 1233, 1199, 1151, 1104, 1034, 974, 948, 888, 852, 830, 792, 749, 729, 676, 633 cm^{-1}; HRMS (ESI) (m/z) [M+Na]$^+$ $C_{12}H_9{}^{35}Cl_2NNaO_2$ calcd for 291.9903, found 291.9905.

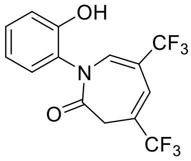

1-(2-hydroxyphenyl)-4,6-bis(trifluoromethyl)-1,3-dihydro-2*H*-azepin-2-one (5r)

Yield: 52 mg, 77%; white solid, mp 144-145 ℃; R_f = 0.51 (EA/PE = 1/3); ^{1}H NMR (500 MHz, $CDCl_3$) δ 7.16-7.11 (m, 1H), 6.98-6.92 (m, 2H), 6.90-6.85 (m, 2H), 6.76 (d, J = 8.0 Hz, 1H), 6.48 (s, 1H), 3.22 (s, 2H) ppm; ^{13}C NMR (125 MHz, $CDCl_3$) δ 164.7 (s), 150.9 (s), 135.9 (q, $J_{C\text{-}F}$ = 6.3 Hz), 130.7 (d), 128.5 (d), 127.7 (s), 123.9 (q, $J_{C\text{-}F}$ = 32.5 Hz), 123.7-123.4 (m), 123.3 (q, $J_{C\text{-}F}$ =

270.0 Hz), 121.8 (q, *JC-F* = 271.3 Hz), 121.2 (d), 118.1 (d), 114.8 (q, *JC-F* = 31.3 Hz), 35.5 (t) ppm; ^{19}F NMR (471 MHz, $CDCl_3$) δ -63.64, -68.49 ppm; IR (ATR) $\tilde{\nu}$ = 3280, 3092, 2930, 1659, 1623, 1600, 1512, 1461, 1378, 1344, 1300, 1278, 1227, 1190, 1169, 1106, 1061, 1036, 975, 958, 906, 890, 858, 840, 816, 786, 761, 731, 703, 664, 641, 626, 607 cm^{-1}; HRMS (ESI) (*m/z*) $[M+Na]^+$ $C_{14}H_9F_6NNaO_2$ calcd for 360.0430, found 360.0434.

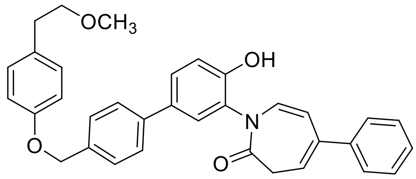

1-(4-hydroxy-4'-((4-(2-methoxyethyl)phenoxy)methyl)-[1,1'-biphenyl]-3-yl)-5-phenyl-1,3-dihydro-2*H*-azepin-2-one (5s)

Yield: 55 mg, 53%; white solid, mp 193-194 ℃; R_f = 0.14 (EA/PE =1/5); ^{1}H NMR (300 MHz, $CDCl_3$) δ 7.47-7.26 (m, 10H), 7.21 (d, *J* = 2.1 Hz, 1H), 7.10-6.99 (m, 3H), 6.89-6.81 (m, 2H), 6.58-6.41 (m, 2H), 6.27 (d, *J* = 9.0 Hz, 1H), 6.02 (t, *J* = 6.9 Hz, 1H), 4.99 (s, 2H), 3.50 (t, *J* = 7.2 Hz, 2H), 3.30-3.18 (m, 5H), 2.75 (t, *J* = 6.9 Hz, 2H) ppm; ^{13}C NMR (75 MHz, $CDCl_3$) δ 167.7 (s), 157.2 (s), 150.6 (s), 139.6 (s), 139.5 (s), 138.2 (s), 136.0 (s), 134.3 (s), 132.1 (d), 131.3 (s), 129.8 (d, 2C), 129.4 (s), 128.6 (d, 2C), 128.1 (d), 128.0 (d, 2C), 127.0 (d, 2C), 126.8 (d, 2C), 126.2 (d), 119.9 (d), 119.2 (d), 119.1 (d), 118.0 (d), 114.8 (d, 2C), 73.8 (t), 69.7 (t), 58.6 (q), 37.7 (t), 35.2 (t) ppm; IR (reflection) $\tilde{\nu}$ = 3033, 2932, 2864, 2806, 1637, 1613, 1589, 1511, 1440, 1366, 1311, 1237, 1178, 1114, 1047, 1017, 967, 890, 868, 806, 782, 765, 730, 692, 656, 627 cm^{-1}; HRMS (ESI) (*m/z*) $[M+H]^+$ $C_{34}H_{32}NO_4$ calcd for 518.2326, found 518.2337.

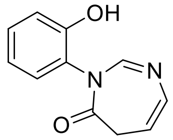

3-(2-hydroxyphenyl)-3,5-dihydro-4*H*-1,3-diazepin-4-one (5v)

Yield: 4 mg, 10%; pale yellow oil; ^{1}H NMR (300 MHz, $CDCl_3$) δ 8.58 (brs, 1H), 8.34-8.25 (m, 1H), 7.72-7.64 (m, 1H), 7.54-7.46 (m, 1H), 7.38-7.30 (m, 2H), 7.04-6.96 (m, 1H), 5.19-5.02 (m, 1H), 3.71-3.65 (m, 2H) ppm; ^{13}C NMR (75 MHz, $CDCl_3$) δ 163.1, 158.6, 150.7, 140.8, 125.1, 124.6, 123.9, 119.6, 110.6, 103.6, 25.4 ppm; HRMS (ESI) (*m/z*) $[M+H]^+$ $C_{11}H_{11}N_2O_2$ calcd for 203.0815, found 203.0815.

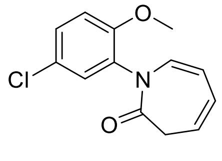

1-(5-chloro-2-methoxyphenyl)-1,3-dihydro-2*H*-azepin-2-one (6)

Yield: 46 mg, 92%; yellow oil; R_f = 0.31 (EA/PE = 1/3); ^{1}H NMR (300 MHz, $CDCl_3$) δ 7.24-7.19 (m, 1H), 7.03 (d, *J* = 2.4 Hz, 1H), 6.84 (d, *J* = 8.7 Hz, 1H), 6.26 (dd, *J* = 5.1, 9.3 Hz, 1H), 6.10 (d, *J* = 8.7 Hz, 1H), 5.80 (dd, *J* = 5.1, 9.0 Hz, 1H), 5.74-5.64 (m, 1H), 3.74 (s, 3H), 3.32-2.75 (m, 2H) ppm; ^{13}C NMR (75 MHz, $CDCl_3$) δ 166.2 (s), 153.3 (s), 131.1 (s), 131.0 (d), 129.6 (d), 129.2 (d), 127.2 (d), 125.4 (s), 122.9 (d), 114.4 (d), 113.3 (d), 56.2 (q), 37.7 (t) ppm; IR (reflection) $\tilde{\nu}$ = 2941, 2839, 1680, 1593, 1496, 1462, 1441, 1380, 1321, 1284, 1246, 1224, 1164, 1143, 1119, 1024, 957, 868, 809, 766, 698, 646, 612 cm^{-1}; HRMS (EI) (*m/z*) [M] $C_{13}H_{12}{}^{35}ClNO_2$ calcd for 249.0551, found

249.0553.

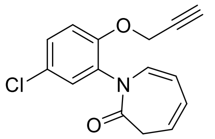

1-(5-chloro-2-(prop-2-yn-1-yloxy)phenyl)-1,3-dihydro-2*H*-azepin-2-one (7)

Yield: 47 mg, 87%; yellow oil; R_f = 0.42 (EA/PE = 1/3); ^{1}H NMR (400 MHz, $CDCl_3$) δ 7.22 (dd, *J* = 2.8, 8.8 Hz, 1H), 7.06 (d, *J* = 2.4 Hz, 1H), 7.01 (d, *J* = 8.8 Hz, 1H), 6.24-6.18 (m, 1H), 6.11 (d, *J* = 8.8 Hz, 1H), 5.84-5.79 (m, 1H), 5.74-5.66 (m, 1H), 4.61 (s, 2H), 3.30-2.80 (m, 2H), 2.44 (t, *J* = 2.4 Hz, 1H) ppm; ^{13}C NMR (100 MHz, $CDCl_3$) δ 166.3 (s), 151.4 (s), 132.0 (s), 130.9 (d), 129.8 (d), 129.1 (d), 127.3 (d), 126.7 (s), 122.9 (d), 115.6 (d), 114.6 (d), 78.0 (s), 76.1 (d), 57.1 (t), 37.7 (t) ppm; IR (reflection) $\tilde{\nu}$ = 3292, 2121, 1671, 1594, 1492, 1380, 1322, 1286, 1214, 1165, 1146, 1120, 1013, 928, 868, 808, 766, 697, 645 cm^{-1}; HRMS (EI) (*m/z*) [M] $C_{15}H_{12}ClNO_2$ calcd for 273.0551, found 273.0559.

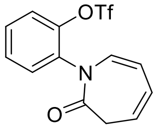

2-(2-oxo-2,3-dihydro-1*H*-azepin-1-yl)phenyl trifluoromethanesulfonate (8)

Yield (0.7 mmol): 163 mg, 70%; white solid, mp 81-82 ℃; R_f = 0.54 (EA/PE = 1/3); ^{1}H NMR (500 MHz, $CDCl_3$) δ 7.39-7.33 (m, 2H), 7.31-7.26 (m, 1H), 7.24-7.19 (m, 1H), 6.24 (dd, *J* = 5.0, 9.0 Hz, 1H), 6.18 (d, *J* = 9.0 Hz, 1H), 5.93 (dd, *J* = 5.0, 9.0 Hz, 1H), 5.76-5.69 (m, 1H), 3.31-2.85 (m, 2H) ppm; ^{13}C NMR (125 MHz, $CDCl_3$) δ 165.5 (s), 144.7 (s), 134.4 (s), 130.2 (d), 129.9 (d), 129.8 (d), 129.2 (d), 127.3 (d), 123.2 (d), 122.4 (d), 118.5 (q, J_{C-F} = 318.8 Hz), 116.0 (d), 37.5 (t) ppm; ^{19}F NMR (283 MHz, $CDCl_3$) δ -73.77 ppm; IR (reflection) $\tilde{\nu}$ = 1686, 1594, 1489, 1421, 1379, 1319, 1285, 1249, 1216, 1152, 1131, 1087, 1036, 1012, 975, 951, 882, 872, 792, 770, 745, 697, 628 cm^{-1}; HRMS (EI) (*m/z*) [M] $C_{13}H_{10}F_3NO_4S$ calcd for 333.0277, found 333.0266.

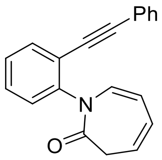

1-(2-(phenylethynyl)phenyl)-1,3-dihydro-2*H*-azepin-2-one (9)

Yield: 54 mg, 95%; white solid, mp 95-96 ℃; R_f = 0.44 (EA/PE = 1/3); ^{1}H NMR (300 MHz, $CDCl_3$) δ 7.58-7.52 (m, 1H), 7.40-7.34 (m, 2H), 7.33-7.22 (m, 5H), 7.17-7.11 (m, 1H), 6.27-6.23 (m, 1H), 6.22-6.18 (m, 1H), 5.84 (dd, *J* = 5.1, 8.7 Hz, 1H), 5.77-5.68 (m, 1H), 3.20-2.96 (m, 2H) ppm; ^{13}C NMR (75 MHz, $CDCl_3$) δ 166.4 (s), 142.9 (s), 133.0 (d), 131.6 (d, 2C), 130.9 (d), 129.2 (d), 128.5 (d), 128.4 (d), 128.2 (d, 2C), 128.1 (d), 127.3 (d), 122.9 (s), 122.6 (d), 122.2 (s), 114.2 (d), 94.3 (s), 85.4 (s), 38.0 (t) ppm; IR (reflection) $\tilde{\nu}$ = 3064, 2887, 2222, 1969, 1682, 1627, 1589, 1494, 1446, 1419, 1377, 1321, 1287, 1251, 1204, 1157, 1129, 1011, 973, 867, 846, 759, 742, 713, 692, 612 cm^{-1}; HRMS (EI) (*m/z*) [M] $C_{20}H_{15}NO$ calcd for 285.1148, found 285.1146.

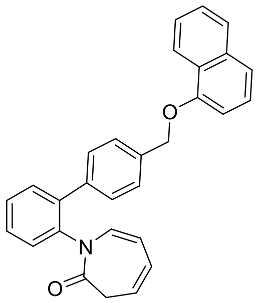

1-(4'-((naphthalen-1-yloxy)methyl)-[1,1'-biphenyl]-2-yl)-1,3-dihydro-2*H*-azepin-2-one (10)

Yield: 79 mg, 95%; white solid, mp 61-62 ℃; R_f = 0.43 (EA/PE =1/5); ^{1}H NMR (300 MHz, $CDCl_3$) δ 8.31-8.24 (m, 1H), 7.76-7.70 (m, 1H), 7.45-7.32 (m, 8H), 7.31-7.26 (m, 1H), 7.24-7.17 (m, 3H), 6.81 (dd, *J* = 0.9, 7.5 Hz, 1H), 5.97 (dd, *J* = 0.6, 9.0 Hz, 1H), 5.84 (dd, *J* = 5.1, 9.0 Hz, 1H), 5.54-5.44 (m, 2H), 5.20 (s, 2H), 2.94-2.75 (m, 2H) ppm; ^{13}C NMR (75 MHz, $CDCl_3$) δ 166.3 (s), 154.3 (s), 140.1 (s), 139.2 (s), 138.2 (s), 136.1 (s), 134.5 (s), 130.9 (d), 130.8 (d), 128.7 (d, 2C), 128.6 (d, 2C), 128.5 (d), 127.4 (d), 127.1 (d, 3C, overlap), 126.4 (d), 125.8 (s), 125.7 (d), 125.2 (d), 122.1 (d), 122.0 (d), 120.5 (d), 114.4 (d), 105.3 (d), 69.8 (t), 37.7 (t) ppm; IR (reflection) $\tilde{\nu}$ = 3051, 1674, 1631, 1592, 1579, 1509, 1483, 1461, 1398, 1380, 1322, 1269, 1241, 1206, 1163, 1130, 1097, 1068, 1009, 867, 823, 791, 760, 720, 696, 676, 611 cm^{-1}; HRMS (EI) (*m/z*) [M] $C_{29}H_{23}NO_2$ calcd for 417.1723, found 417.1715.

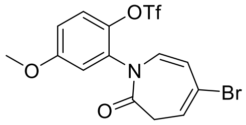

2-(4-bromo-2-oxo-2,3-dihydro-1*H*-azepin-1-yl)-4-methoxyphenyl trifluoromethanesulfonate (11)

Yield (0.38 mmol): 156 mg, 93%; brown solid, mp 90-92 ℃; R_f = 0.63 (EA/PE = 1/3); ^{1}H NMR (300 MHz, $CDCl_3$) δ 7.19 (d, *J* = 9.0 Hz, 1H), 6.87 (dd, *J* = 3.0, 9.0 Hz, 1H), 6.72 (d, *J* = 3.0 Hz, 1H), 6.16 (d, *J* = 9.0 Hz, 1H), 6.00-5.93 (m, 2H), 3.76 (s, 3H), 3.31-2.81 (m, 2H) ppm; ^{13}C NMR (100 MHz, $CDCl_3$) δ 64.8 (s), 159.6 (s), 137.7 (s), 134.7 (s), 130.8 (d), 123.2 (d), 123.1 (d), 119.1 (s), 118.5 (q, $J_{C\text{-}F}$ = 318.0 Hz), 117.2 (d), 115.4 (d), 115.1 (d), 56.0 (q), 38.0 (t) ppm; ^{19}F NMR (283 MHz, $CDCl_3$) δ -73.68 ppm; IR (reflection) $\tilde{\nu}$ = 3058, 2842, 1694, 1600, 1493, 1461, 1414, 1335, 1303, 1250, 1204, 1164, 1136, 1102, 1026, 982, 930, 878, 825, 811, 770, 731, 700, 638, 621, 606 cm^{-1}; HRMS (EI) (*m/z*) [M] $C_{14}H_{11}BrF_3NO_5S$ calcd for 440.9488, found 440.9474.

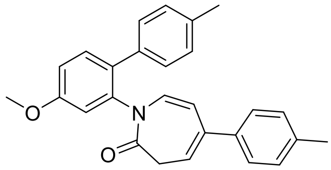

1-(4-methoxy-4'-methyl-[1,1'-biphenyl]-2-yl)-4-(p-tolyl)-1,3-dihydro-2*H*-azepin-2-one (12)

Yield (0.32 mmol): 120 mg, 95%; white solid, mp 145-147 ℃; R_f = 0.40 (EA/PE = 1/3); ^{1}H NMR (300 MHz, $CDCl_3$) δ 7.24 (d, *J* = 8.4 Hz, 1H), 7.06-6.99 (m, 4H), 6.93-6.82 (m, 5H), 6.76 (d, *J* = 2.7 Hz, 1H), 6.09 (d, *J* = 9.0 Hz, 1H), 5.78 (t, *J* = 7.2 Hz, 1H), 5.71 (d, *J* = 9.0 Hz, 1H), 3.77 (s, 3H), 3.26-2.85 (m, 2H), 2.28 (s, 3H), 2.09 (s, 3H) ppm; ^{13}C NMR (75 MHz, $CDCl_3$) δ 167.1 (s), 159.4 (s), 139.4 (s, 2C, overlap), 137.3 (s), 136.5 (s), 135.8 (s), 135.2 (s), 133.2 (s), 131.7 (d), 131.6 (d),

129.0 (d, 2C), 128.8 (d, 2C), 128.2 (d, 2C), 126.9 (d, 2C), 116.8 (d), 115.5 (d), 114.8 (d), 113.4 (d), 55.5 (q), 37.9 (t), 21.1 (q), 21.0 (q) ppm; IR (reflection) $\tilde{\nu}$ = 3021, 2916, 1677, 1611, 1587, 1490, 1419, 1347, 1294, 1247, 1203, 1171, 1112, 1040, 1004, 977, 958, 891, 830, 806, 777, 746, 734, 657, 640 cm^{-1}; HRMS (EI) (*m/z*) [M] $C_{27}H_{25}NO_2$ calcd for 395.1880, found 395.1894.

3. Supplementary Discussion

Mechanistic investigation

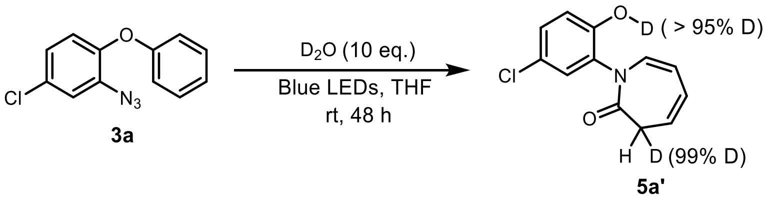

Supplementary Figure 8. Synthesis of 5a' using D_2O.

5a': HRMS (EI) (*m/z*) [M] $C_{12}H_8{}^{35}ClNO_2D_2$ calcd for 237.0520, found 237.0495.

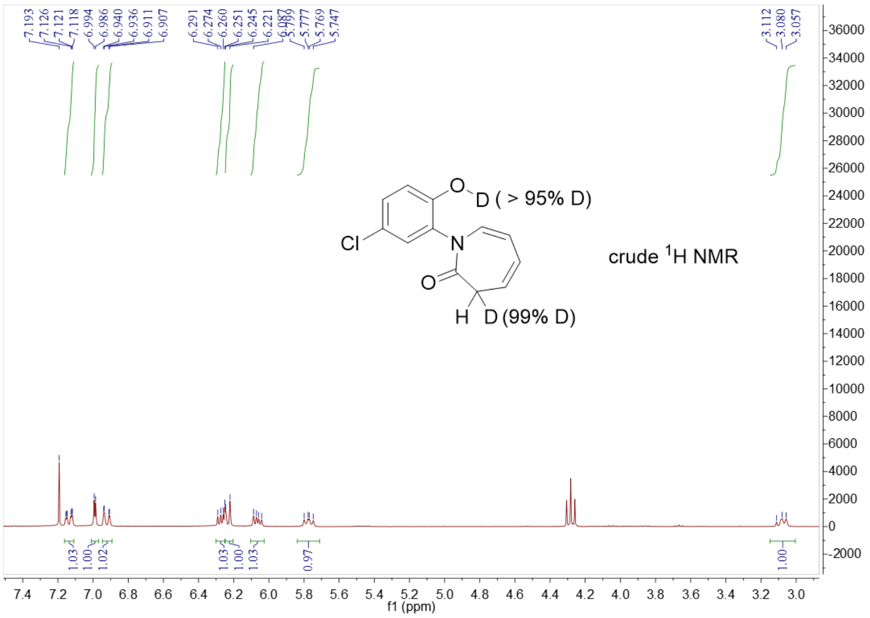

Supplementary Figure 9. Crude 300 MHz ^{1}H NMR of 5a' in $CDCl_3$.

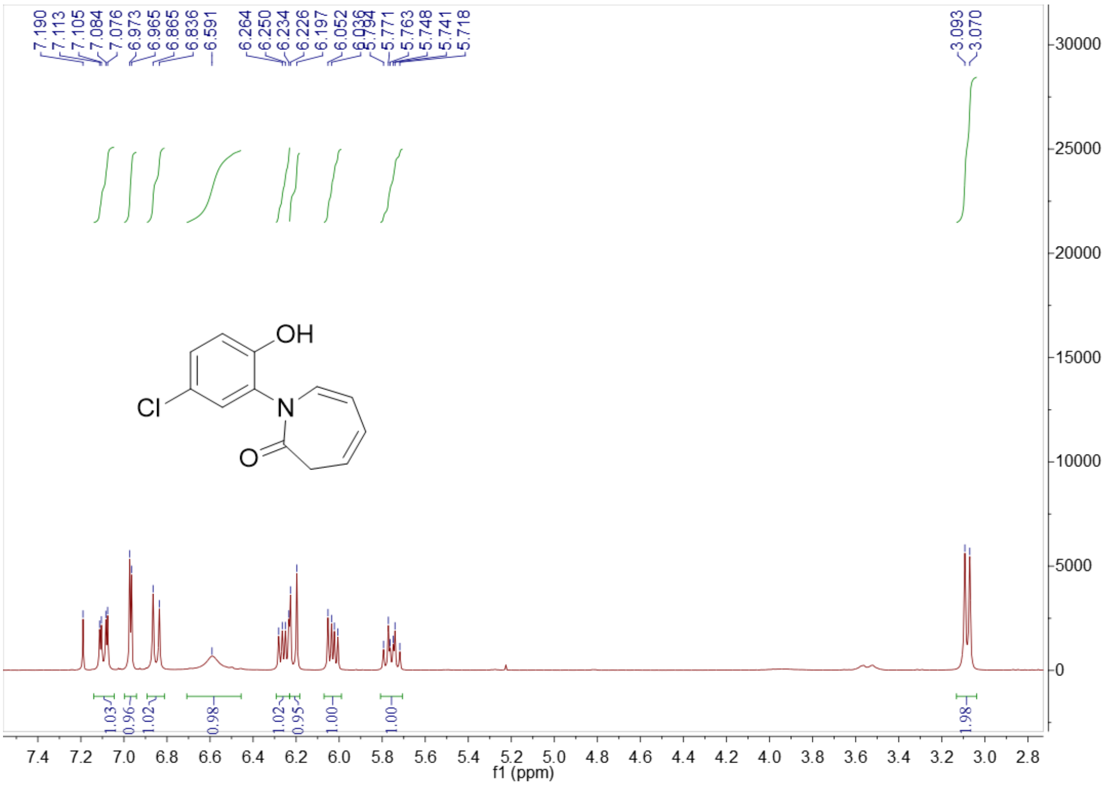

Supplementary Figure 10. 300 MHz ^{1}H NMR of 5a in $CDCl_3$.

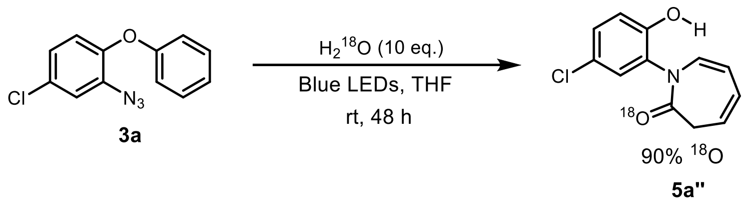

Supplementary Figure 11. Synthesis of 5a''.

5a'': HRMS (EI) (*m/z*) [M] $C_{12}H_{10}{}^{35}ClNO^{18}O$ calcd for 237.0437, found 237.0426.

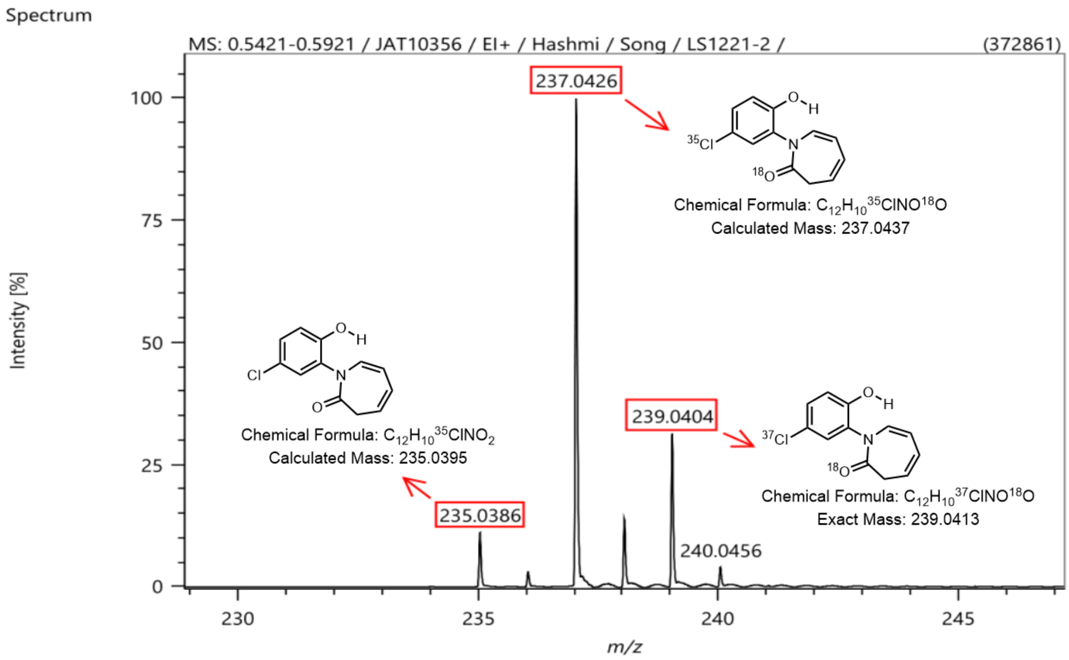

Supplementary Figure 12. HRMS (EI) of 5a'' and 5a.

The incorporation of ^{18}O atom was determined by EI: height (%): 235.03857 (11.14), 237.04259 (100.00).

^{13}C NMR (150 MHz) of **5a''** in $CDCl_3$

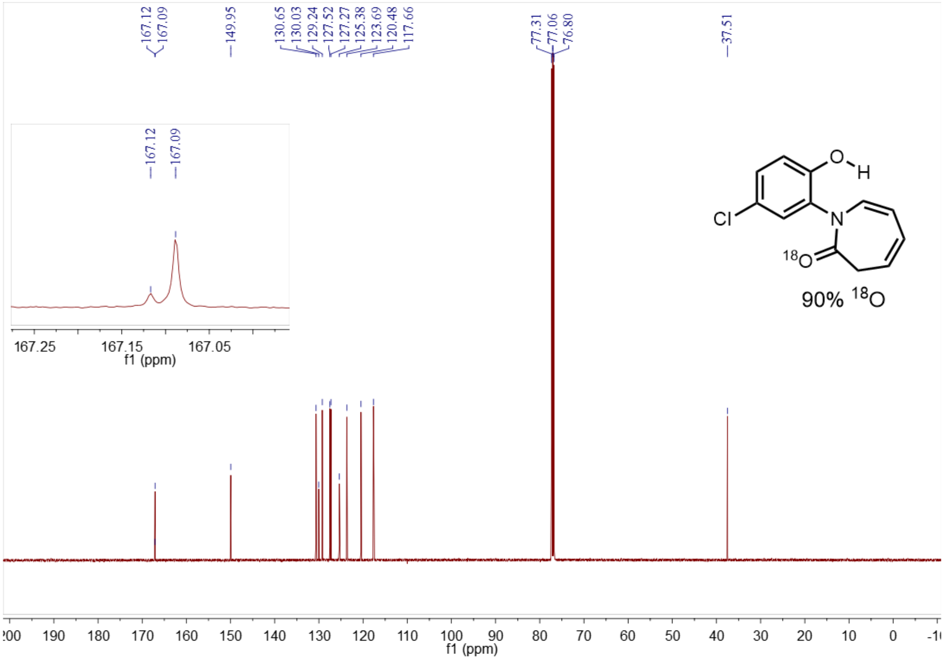

Supplementary Figure 13. 125 MHz ^{13}C NMR of 5a'' in $CDCl_3$.

^{13}C NMR (150 MHz) of **5a** in $CDCl_3$

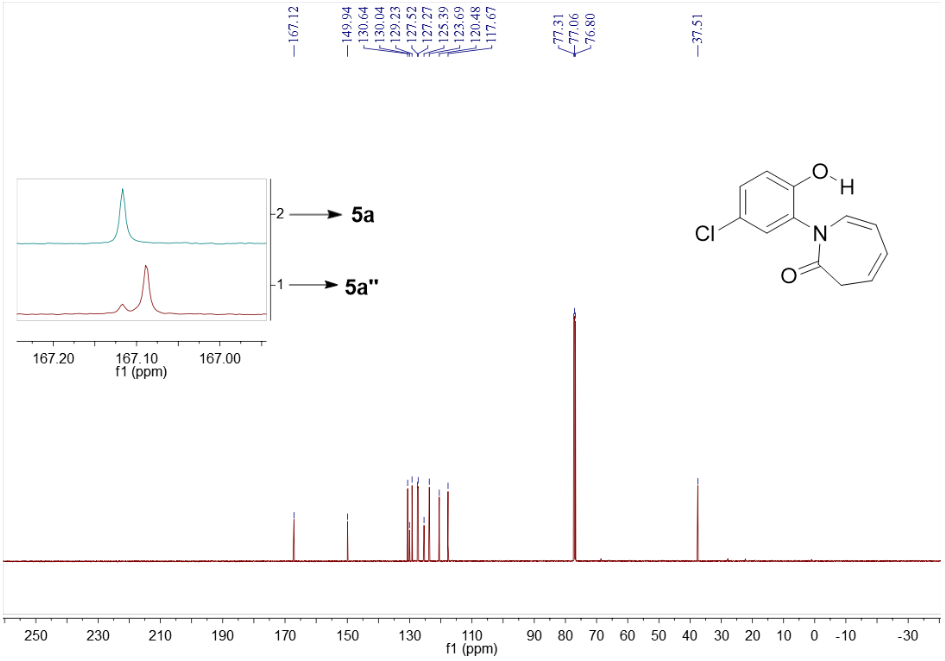

Supplementary Figure 14. Comparison of 5a and 5a'' by 125 MHz ^{13}C NMR in $CDCl_3$.

Additional calculation results and computational details

Benchmark calculations. The transformation given in Supplementary Fig. 15 was experimentally reported by Ellison et al. to have a ΔE_{ST} value of 18 ± 2 kcal/mol in the gas phase[7]. Consistent with this experimental finding, this ΔE_{ST} is calculated to be 19.4 kcal/mol at the CCSD(T)/def2-TZVP//M06-2X/6-31G(d) level of theory. To find out which DFT method gives more accurate result, the single point calculations were carried out at the different levels of theory using the Method/def2-TZVP//M06-2X/6-31G(d) calculations. As can be seen from Supplementary Fig. 15a, compared to other methods, the ΔE_{ST} calculated by the CAM-B3LYP functional is in closer agreement with the CCSD(T) reference and the experimental value. To investigate whether this is also true for a different nitrene molecule, we calculated the ΔE_{ST} for the transformation given in Supplementary Fig. 15b at different levels of theory, SDD/Method/def2-TZVP//SDD/M06-2X/6-31G(d), in THF and again found that the CAM-B3LYP functional gives a ΔE_{ST} closer to that obtained by the CCSD(T) reference.

Although our calculations indicated that M06-2X is not a very accurate method for estimating the ΔE_{ST} of nitrenes, it is benchmarked as a reliable method for studying pericyclic reactions (Supplementary Fig. 15c). We found that compared to other functionals, the M06-2X functional show better agreement with the CCSD(T) reference; for this benchmark evaluation, the calculations were carried out at the SDD/Method/def2-TZVP//SDD/M06-2X/6-31G(d) level of theory in THF.

As can be seen from Fig. 7a, transformation **3a** → $\mathbf{A^T}$ + N_2 is calculated to be exergonic. This exergonicity is supported by an additional calculation at the SMD/CCSD(T)/def2-TZVP//SMD/M06-2X/6-31G(d) level of theory in THF for N_2 release from phenyl azide with ΔG = -11.1 kcal/mol (Supplementary Fig. 15d). This result explicitly indicates that the liberation of N_2 from aryl azides to give a triplet nitrene is most likely a downhill process.

(a)

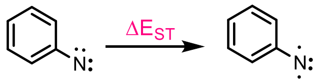

1^{OSS} → 2^{T}

	ΔEST
CCSD(T)	19.4
CAM-B3LYP	18.5
B3LYP-D3	16.7
M06-2X	26.0
M06-2X-D3	26.0
M06	25.7
M06-D3	25.7

(b)

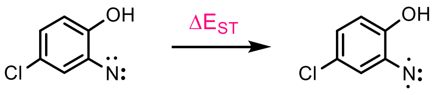

2^{OSS} → $2'^{T}$

	ΔEST
CCSD(T)	15.7
CAM-B3LYP	16.0
B3LYP-D3	14.3
M06-2X	21.7
M06-2X-D3	21.7
M06	20.8
M06-D3	20.8

(c)

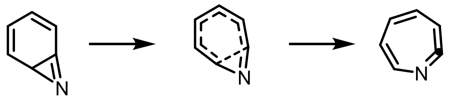

	iii	TS_{ii}	iv
CCSD(T)	0.0 (0.0)	5.4 (6.4)	-3.8 (-3.5)
M06-2X	0.0 (0.0)	4.1.(5.1)	-2.9 (-2.6)
M06-2X-D3	0.0 (0.0)	4.1 (5.1)	-2.9 (-2.6)
M06	0.0 (0.0)	4.9 (5.9)	-2.0 (-1.7)
M06-D3	0.0 (0.0)	4.9 (5.9)	-2.0 (-1.7)
B3LYP-D3	0.0 (0.0)	2.4 (3.5)	-7.6 (-7.3)
CAM-B3LYP	0.0 (0.0)	4.5 (5.5)	-6.1 (-5.8)

(d)

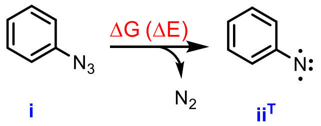

i → ii^{T}

	ΔG (ΔE)
CCSD(T)	-11.1 (1.3)
M06-2X	-7.0 (5.4)

Supplementary Figure 15. Results of benchmark calculations. **a** Calculated ΔE_{ST} for phenyl nitrene. **b** Calculated ΔE_{ST} for a substituted phenyl nitrene. **c** Calculations on a pericyclic reaction. **d** Calculations on N_2 release from phenyl azide. Free energies (potential energies) are given in kcal/mol.

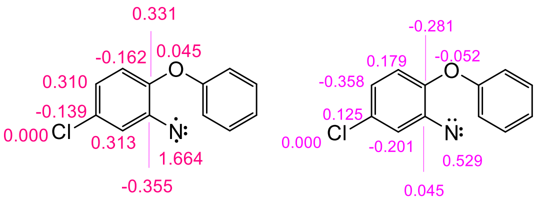

A^{T} A^{OSS}

Supplementary Figure 16. Distribution of the unpaired electrons over triplet structure A^{T} and A^{OSS} obtained by Mulliken spin density calculation.

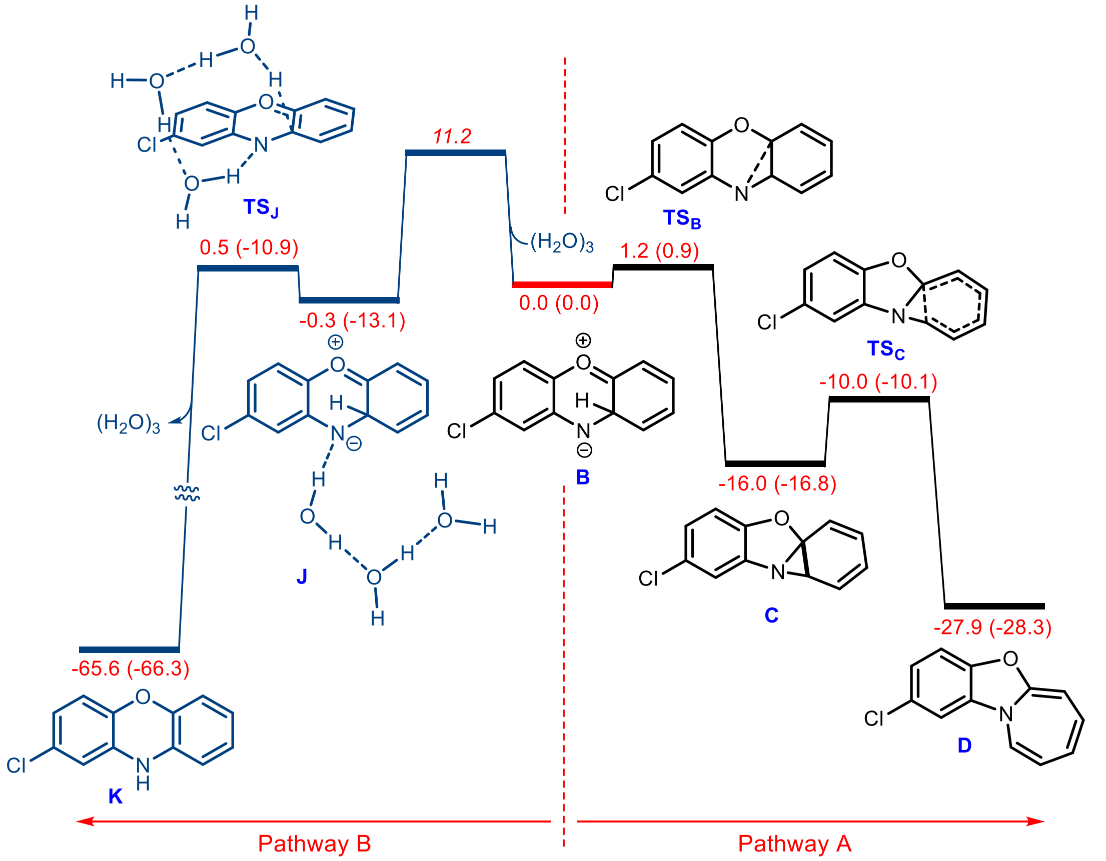

Supplementary Figure 17. Calculated energy profile for ring-closing and ring-opening through sequence B → C → D (pathway A) and aromatization process via sequence B → J → K (pathway B). Free energies (potential energies) calculated at the SMD/M06-2X/def2-TZVP//SMD/M06-2X/6-31G(d) level of theory in THF are given in kcal/mol. As can be seen from this figure, pathway A is kinetically more favorable than pathway B, supported by the fact that $\mathbf{TS_B}$ lies lower in energy than transition structure connecting **B** to **J**. This comparison clearly shows that although the aromatization process (pathway B) is calculated to be fast with an activation barrier of 11.2 kcal/mol, it occurs much slower than transformation **B** → **D**. This explains why no phenoxazine is experimentally formed during the light-promoted reaction.

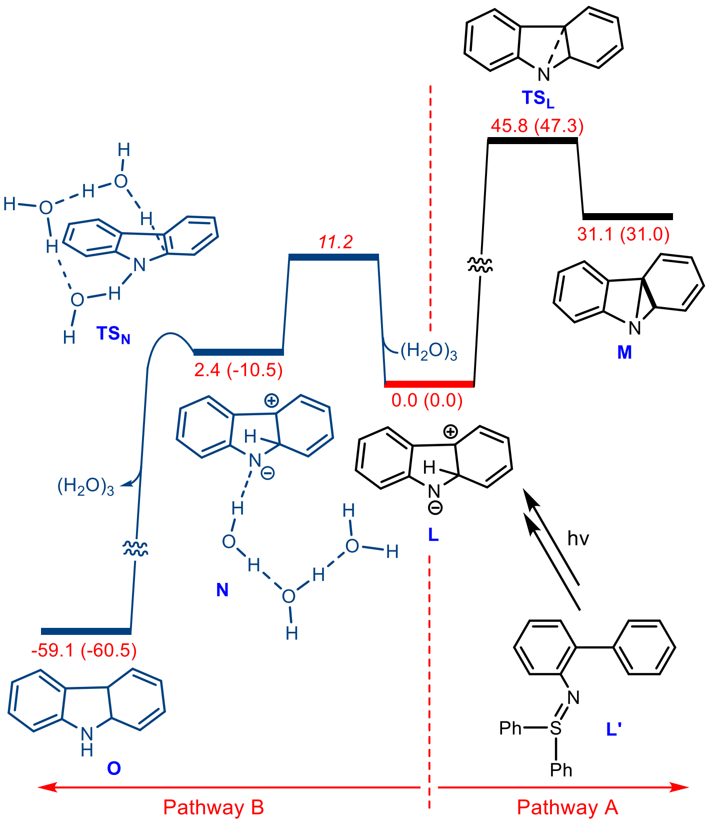

Supplementary Figure 18. Calculated energy profile for ring-closing through transformation L → M (pathway A) and aromatization process via sequence L → N → O (pathway B). Free energies (potential energies) calculated at the SMD/M06-2X/def2-TZVP//SMD/M06-2X/6-31G(d) level of theory in THF are given in kcal/mol. Attempts to locate transition structure **TS$_N$** were unsuccessful due to the flatness of the potential energy surface in the vicinity of intermediate **N**. Species **L** is an intermediate formed during the light-promoted synthesis of carbazole **O** from aryl sulfilimine **L′**. As can be seen from this figure, pathway A is kinetically less favorable than pathway B, supported by the fact that **TS$_L$** lies much higher in energy than transition structure connecting **L** to **N**. This comparison clearly show that, in contrast to intermediate **B** (Supplementary Figure 17), intermediate **L** prefers pathway B. This result explains why the nitrene produced from sulfilimine **L′** practically only affords carbazole **O**, as reported by us in a recent study (ref 7a).

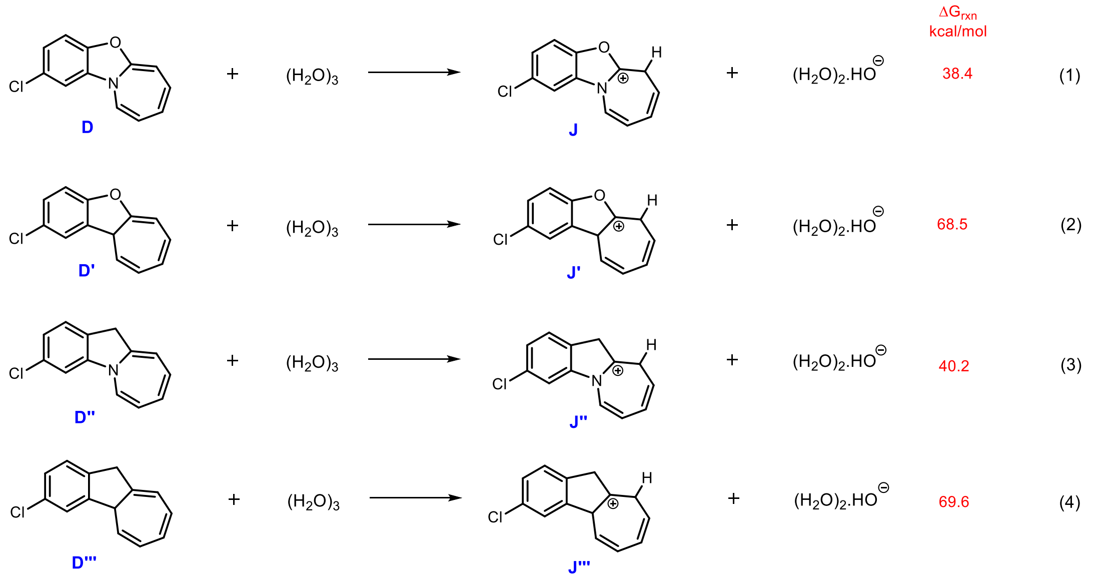

Supplementary Figure 19. Calculated reaction free energies for deprotonation of $(H_2O)_3$ by model molecules **D-D‴**. The endergonicity for reactions described in eqs 1 and 3 is much lower than that in eqs 2 and 4. This indicates that the basicity of such molecules are mainly enhanced if the N atom is present on the ring.

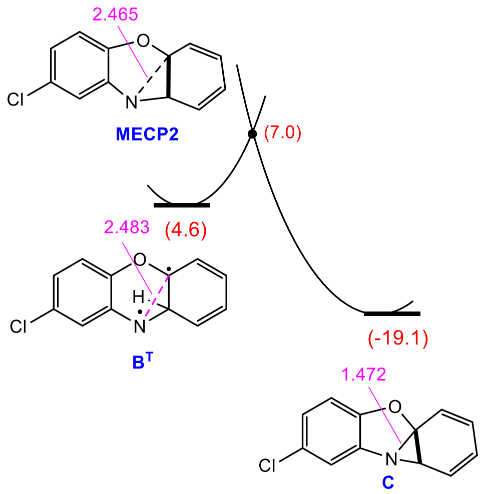

Supplementary Figure 20. Calculated energy profile for direct formation of **C** from $\mathbf{B^T}$. The red values are relative potential energies in kcal/mol, and the pink values are selective bond distances in Å. Since **MECP2** lies 1.8 kcal/mol higher in energy than **MECP1**, the formation of C via the pathway depicted in this figure is less likely.

Computational details

We used TD-DFT calculations using the Coulomb-attenuating method functional by Handy and coworkers[8] (CAM-B3LYP) to optimize structures and investigate the mechanism of photoactivation using Q-Chem 5.4.[9] For this part of the calculations, solvent effects were considered using the closely-related conductor-like PCM (C-PCM) model[10] with tetrahydrofuran as the solvent.

Gaussian 16[11] was used to fully optimize all the structures reported in this paper at the M06-2X level of theory.[12] It is well-documented that the M06-2X functional gives more accurate activation barrier and reaction energies than other DFT methods for an organic transformation.[13] For this part

of the calculations, solvent effects were considered using the SMD solvation model[14] with tetrahydrofuran as the solvent.

The 6-31G(d) basis set[15] was used for optimization. Frequency calculations were carried out at the same level of theory as those for structural optimization. Transition structures were located using the Berny algorithm. Intrinsic reaction coordinate (IRC) calculations were used to confirm the connectivity between transition structures and minima.[16] To further refine the energies obtained from the 6-31G(d) calculations, we carried out single-point energy calculations using the def2-TZVP basis set.[17]

Tight convergence criterion and ultrafine integral grid were also employed to increase the accuracy of all calculations. In this work, the free energy for each species in solution was calculated using the following formula:

$$G = E(\text{def2-TZVP}) + G(\text{6-31G(D)}) - E(\text{6-31G(D)}) + \Delta G^{1atm\rightarrow 1M} \quad (5)$$

where $\Delta G^{1atm\rightarrow 1M}$ = 1.89 kcal/mol is the free-energy change for compression of 1 mol of an ideal gas from 1 atm to the 1 M solution phase standard state. In simple word, the $\Delta G^{1atm\rightarrow 1M}$ term results in a correction of -1.89 (or +1.89) kcal/mol for a 2 to 1 (or a 1 to 2) transformation.

Minimum energy crossing points (MECPs) between closed-shell singlet and triplet states were located using both Q-Chem for the photoactivation part and the code developed by Harvey et al[18] interfaced with Gaussian 16 for others. In this study, the open-shell singlet and triplet structures were denoted by superscripts OSS and T, respectively.

The wave functions obtained for structures of unrestricted open-shell singlet state are contaminated with triplet state wave functions. To exclude the triplet state contribution, spin-projection corrections suggested by Yamaguchi and co-workers were employed.[19] Accordingly, the spin-projected singlet energy ($E_{singlet}$) for a given molecule is calculated based on the following formulas:

$$E_{singlet} = E_{OSS} + \chi[E_{OSS} - E_T] \quad (6)$$

$$\chi = \frac{\langle S^2\rangle_{OSS}/\langle S^2\rangle_T}{1-(\langle S^2\rangle_{OSS}/\langle S^2\rangle_T)} \quad (7)$$

where E_{OSS} and E_T are the potential energies of the unrestricted open-shell singlet and triplet states, respectively, and $\langle S^2\rangle_{OSS}$ and $\langle S^2\rangle_T$ corresponds to the $\langle S^2\rangle$ value obtained from the calculations of the open-shell singlet and triplet states, respectively.

The free energy barriers for formation of **N** from **L** + $(H_2O)_3$ and **J** from **B** + $(H_2O)_3$ were estimated according to the protocol presented by Hall and Hartwig. In this protocol, for example, the Gibbs free energy barrier for a dissociation reaction such as A-B → A + B is estimated as $\Delta G^‡ \approx \Delta H$ = HA + HB − HA-B.[20]

For computational details by usuing Q-chem and Gaussian, see files: **Supplementary Data 1** and **Supplementary Data 2**.

4. Supplementary Figures

X-ray structure

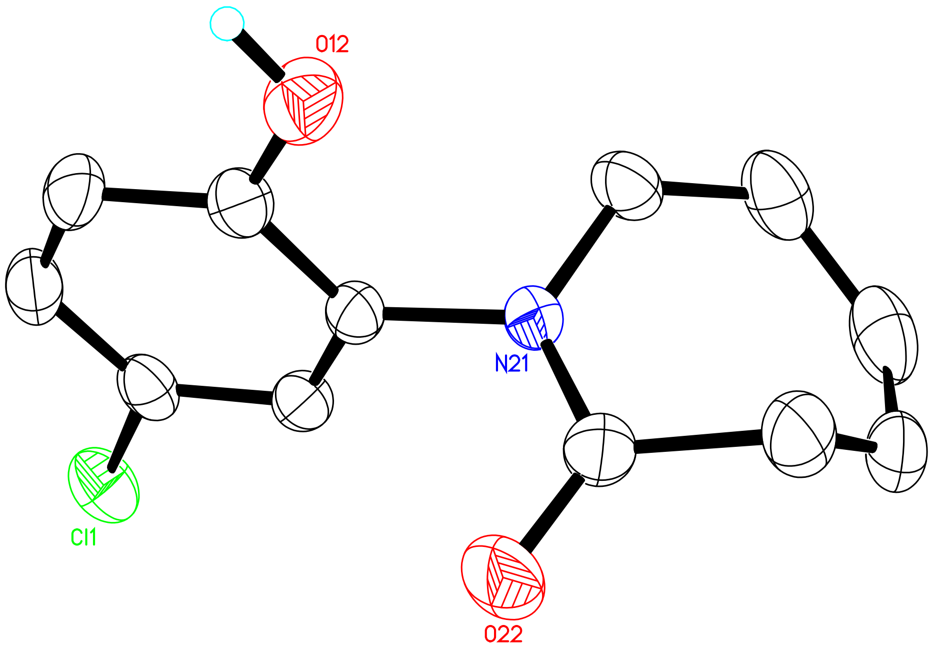

Supplementary Figure 21. Solid state molecular structure of 5a

NMR spectra

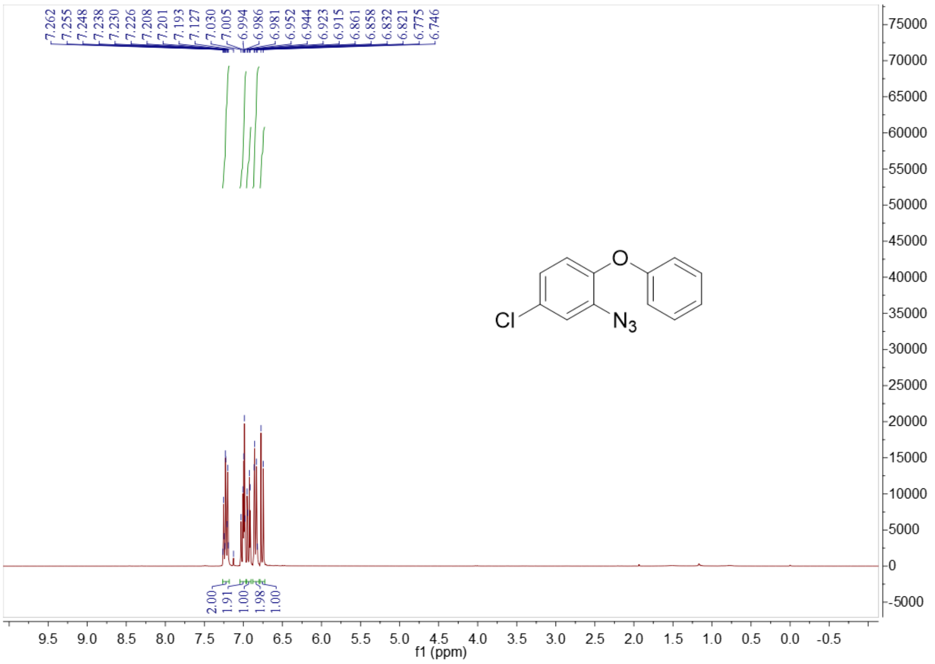

Supplementary Figure 22. 300 MHz ^{1}H NMR of compound 3a in $CDCl_3$

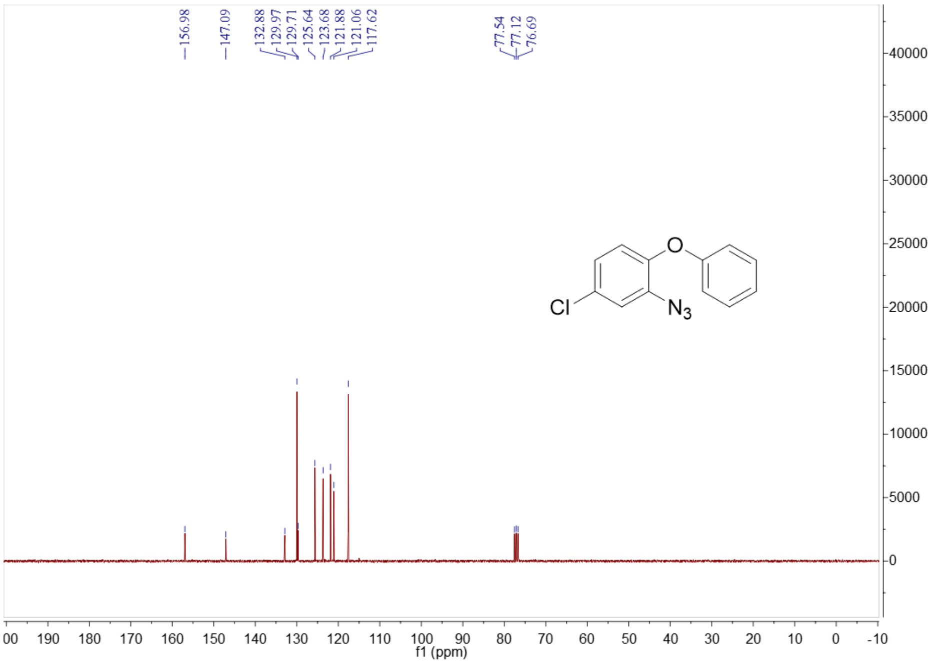

Supplementary Figure 23. 75 MHz ^{13}C NMR of compound 3a in $CDCl_3$

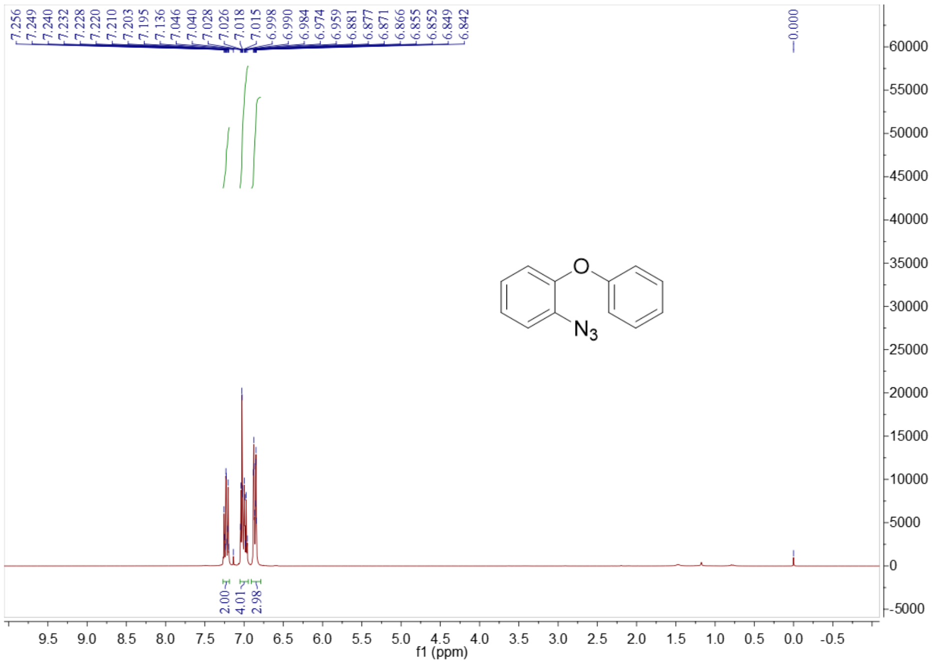

Supplementary Figure 24. 300 MHz ^{1}H NMR of compound 3b in $CDCl_3$

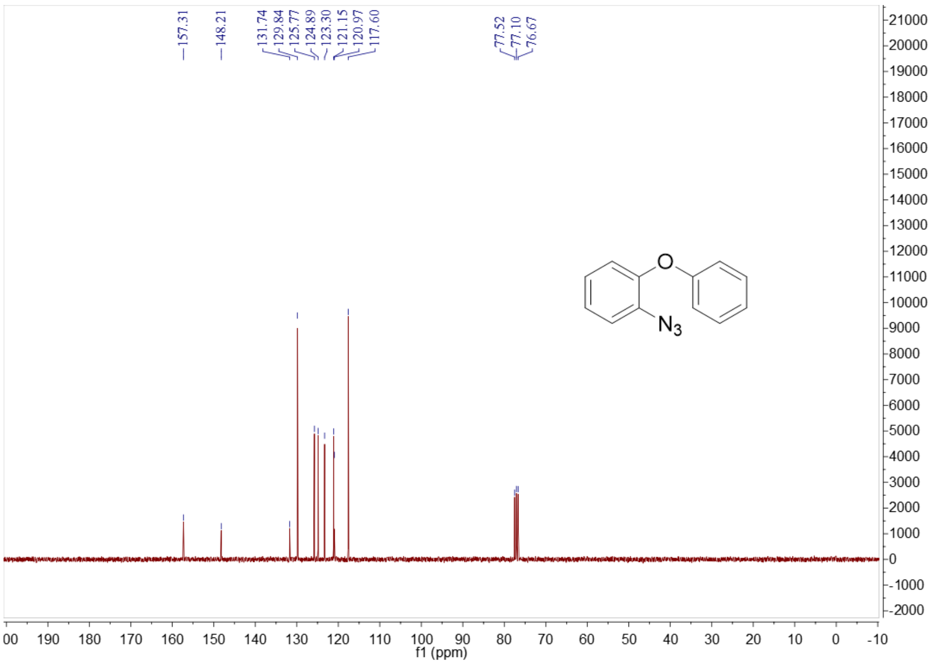

Supplementary Figure 25. 75 MHz ^{13}C NMR of compound 3b in $CDCl_3$

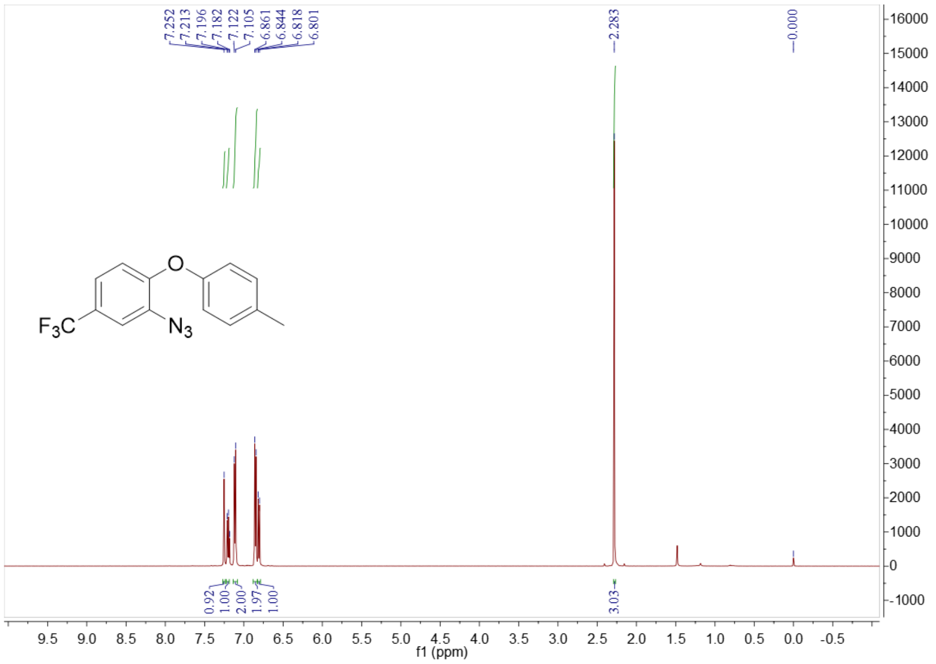

Supplementary Figure 26. 500 MHz ^{1}H NMR of compound 3c in $CDCl_3$

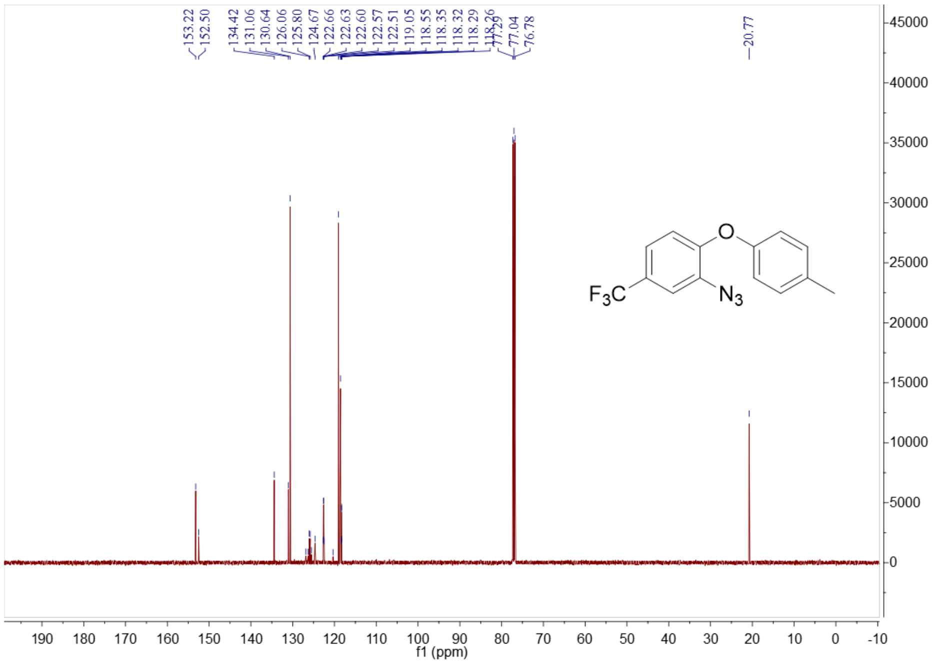

Supplementary Figure 27. 125 MHz ^{13}C NMR of compound 3c in $CDCl_3$

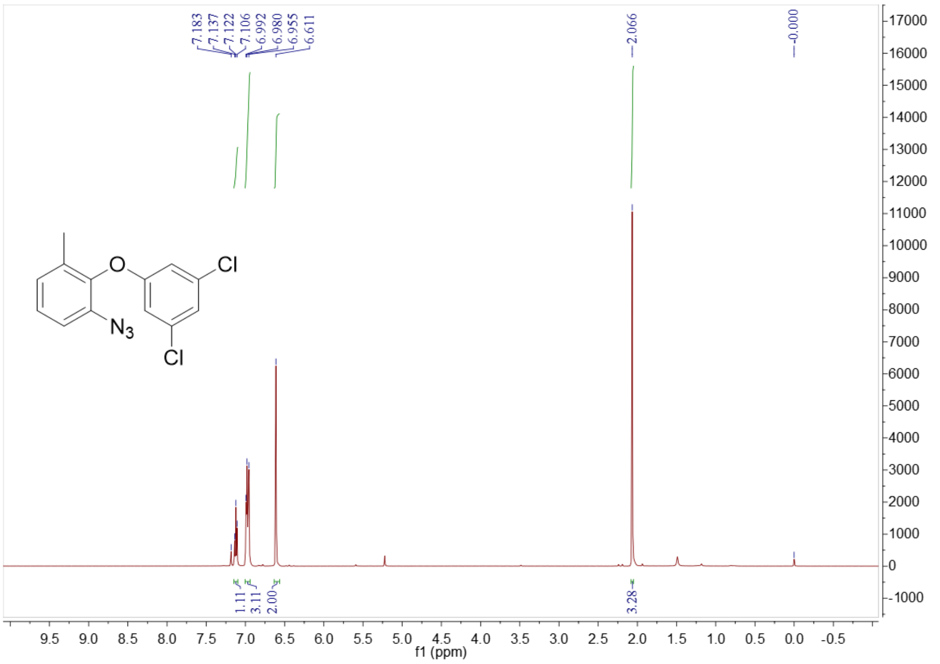

Supplementary Figure 28. 500 MHz ^{1}H NMR of compound 3d in $CDCl_3$

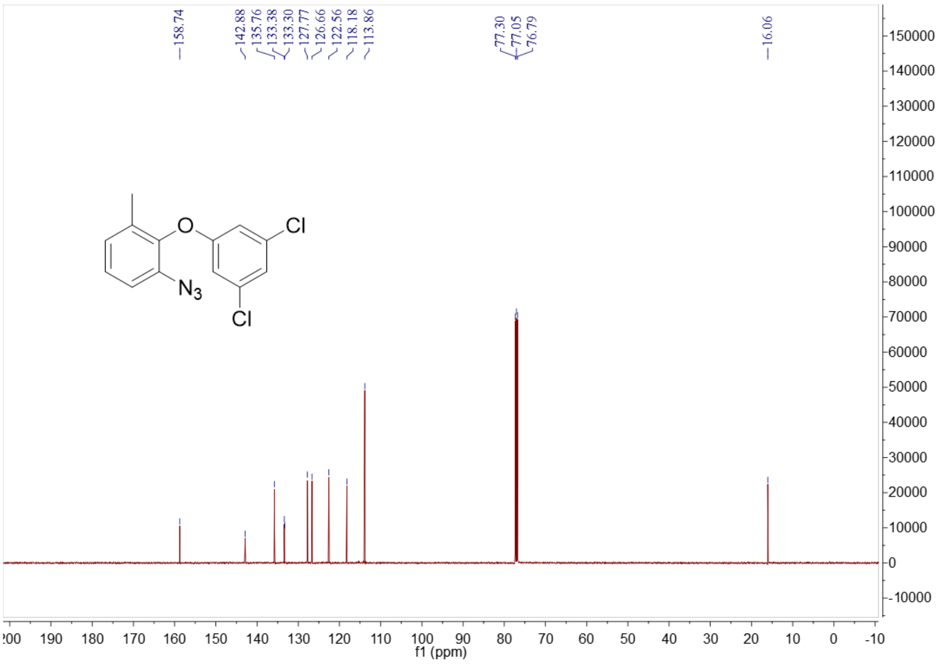

Supplementary Figure 29. 125 MHz ^{13}C NMR of compound 3d in $CDCl_3$

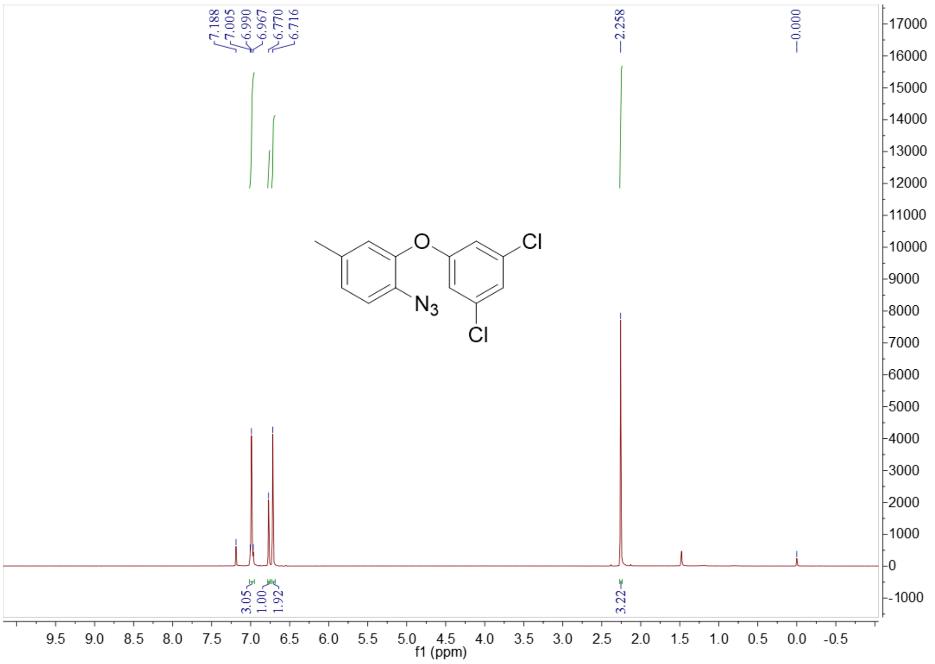

Supplementary Figure 30. 500 MHz ^{1}H NMR of compound 3e in $CDCl_3$

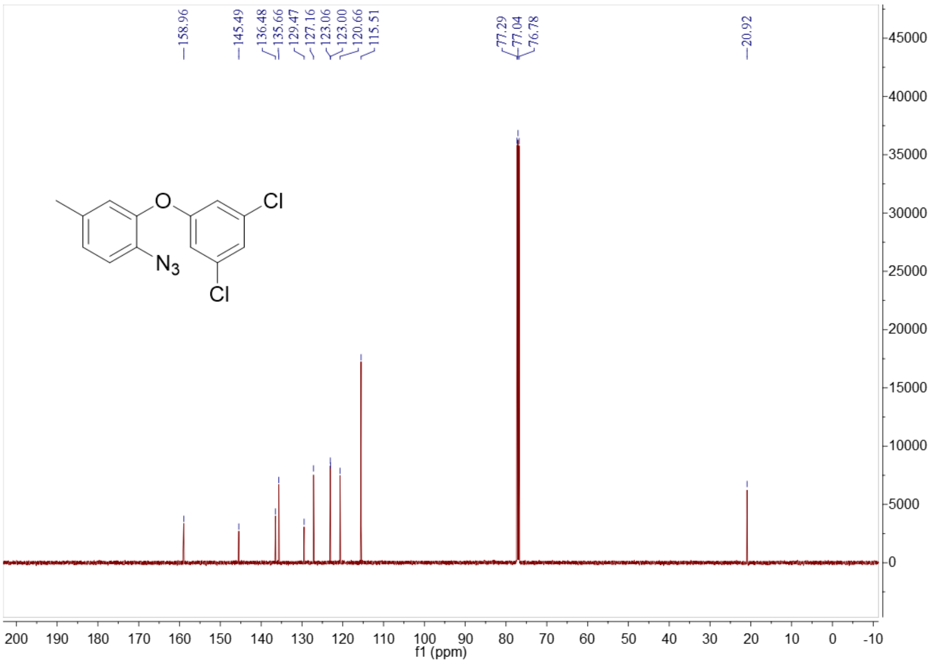

Supplementary Figure 31. 125 MHz ^{13}C NMR of compound 3e in $CDCl_3$

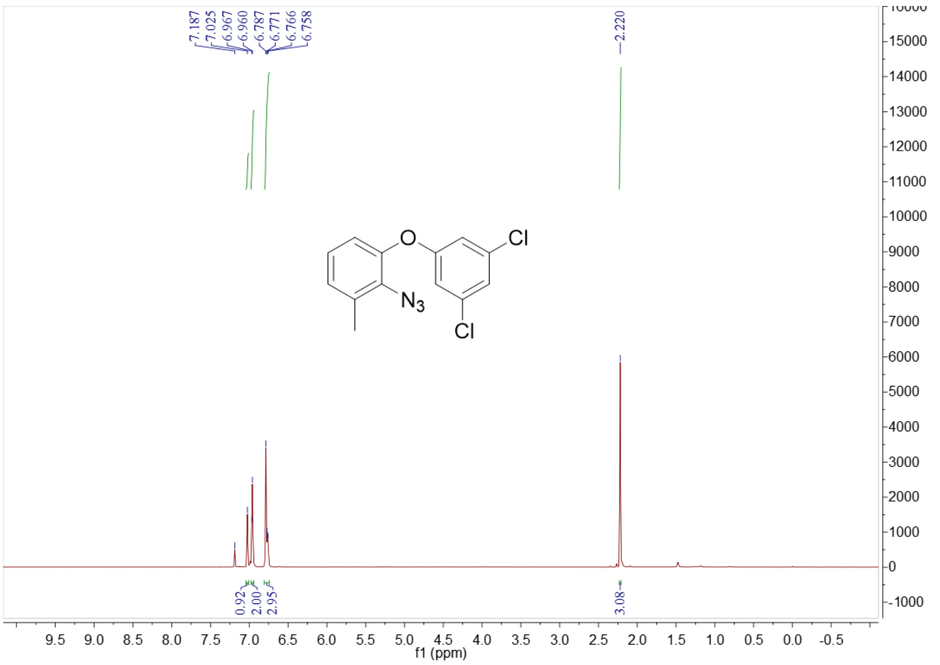

Supplementary Figure 32. 500 MHz ^{1}H NMR of compound 3f in $CDCl_3$

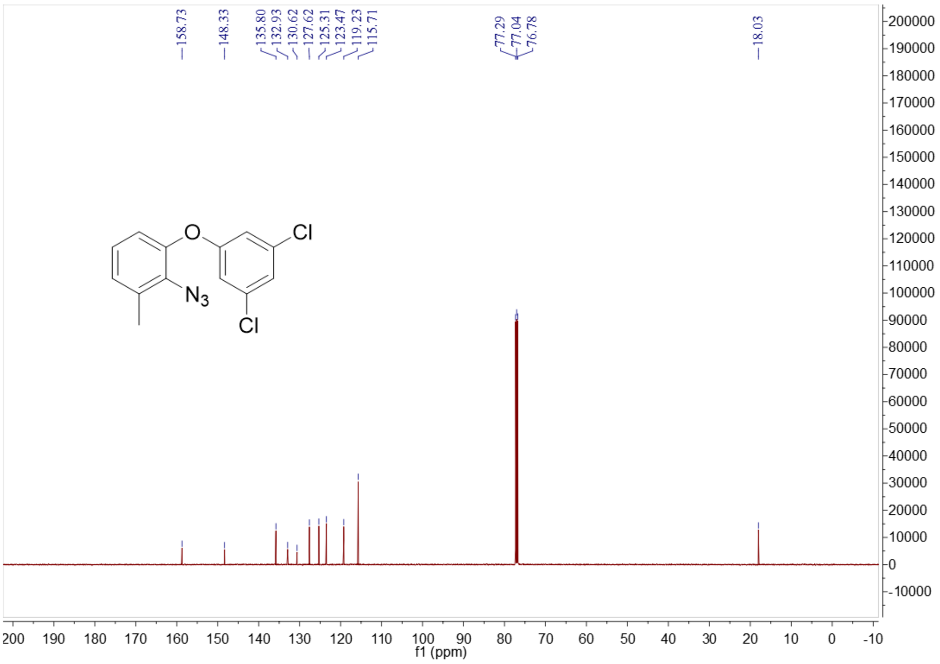

Supplementary Figure 33. 125 MHz ^{13}C NMR of compound 3f in $CDCl_3$

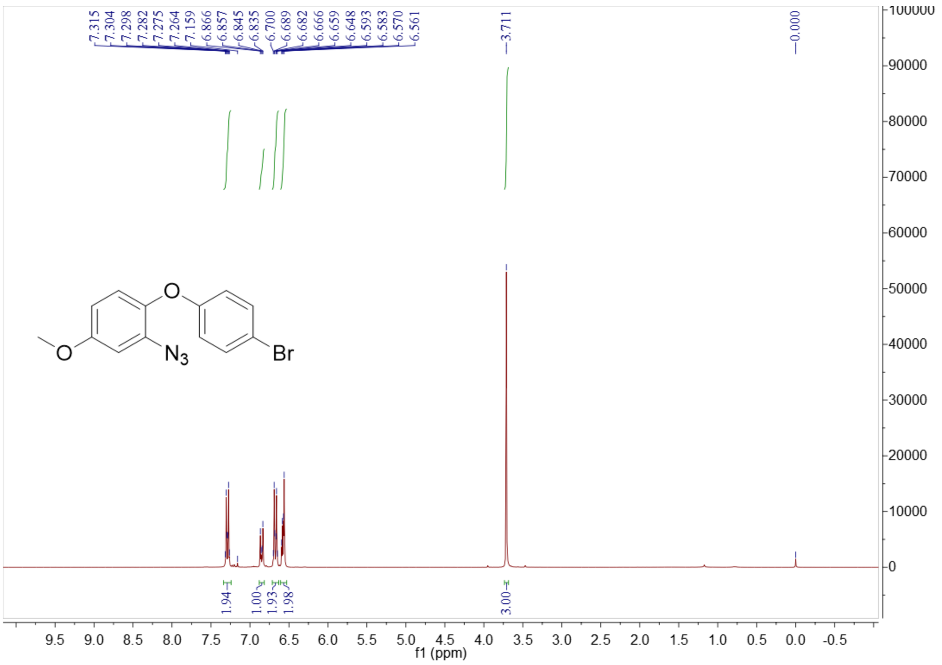

Supplementary Figure 34. 300 MHz ^{1}H NMR of compound 3g in $CDCl_3$

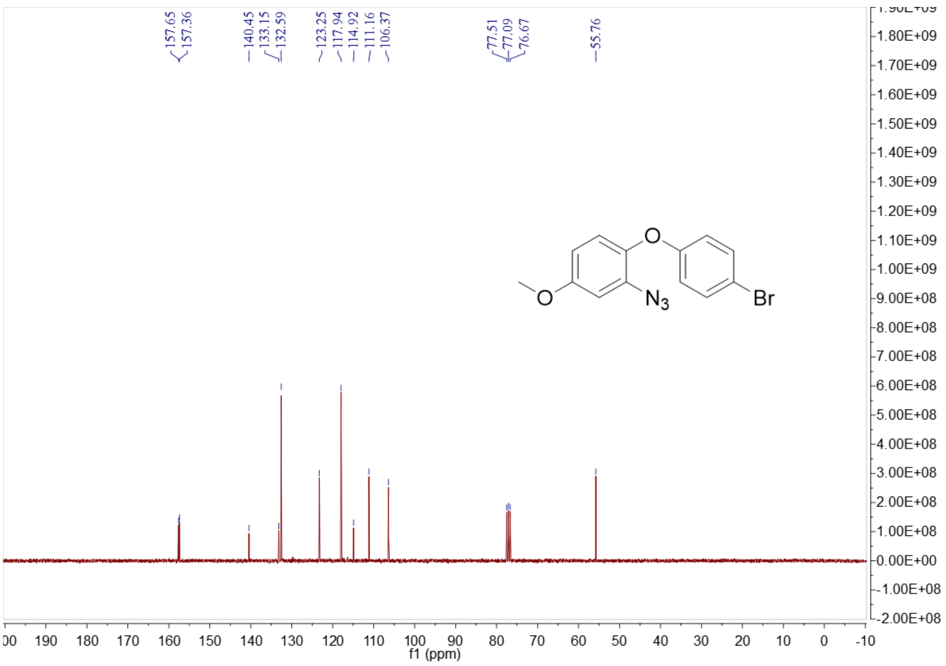

Supplementary Figure 35. 75 MHz ^{13}C NMR of compound 3g in $CDCl_3$

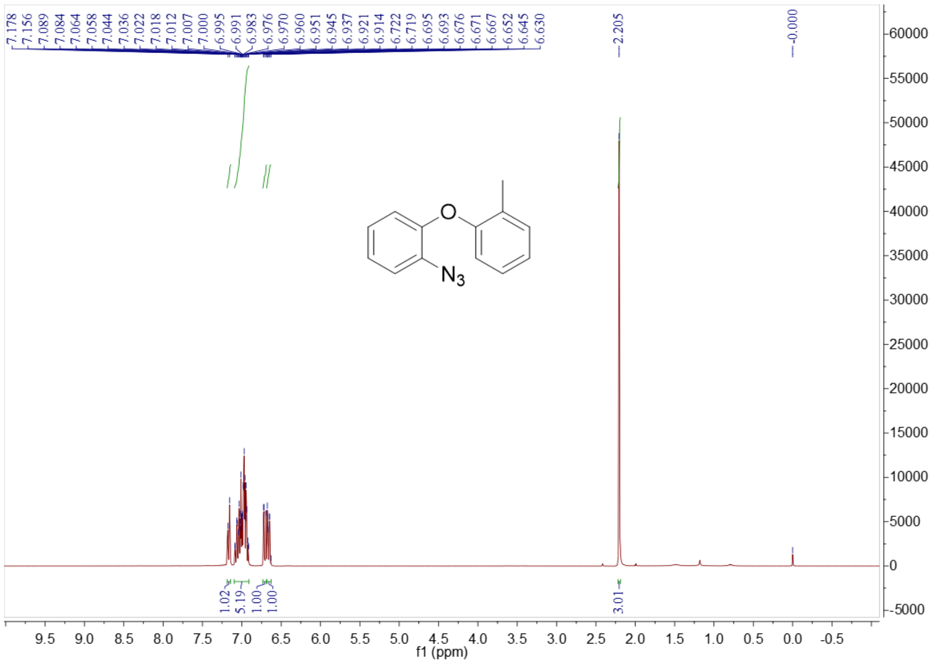

Supplementary Figure 36. 300 MHz ^{1}H NMR of compound 3h in $CDCl_3$

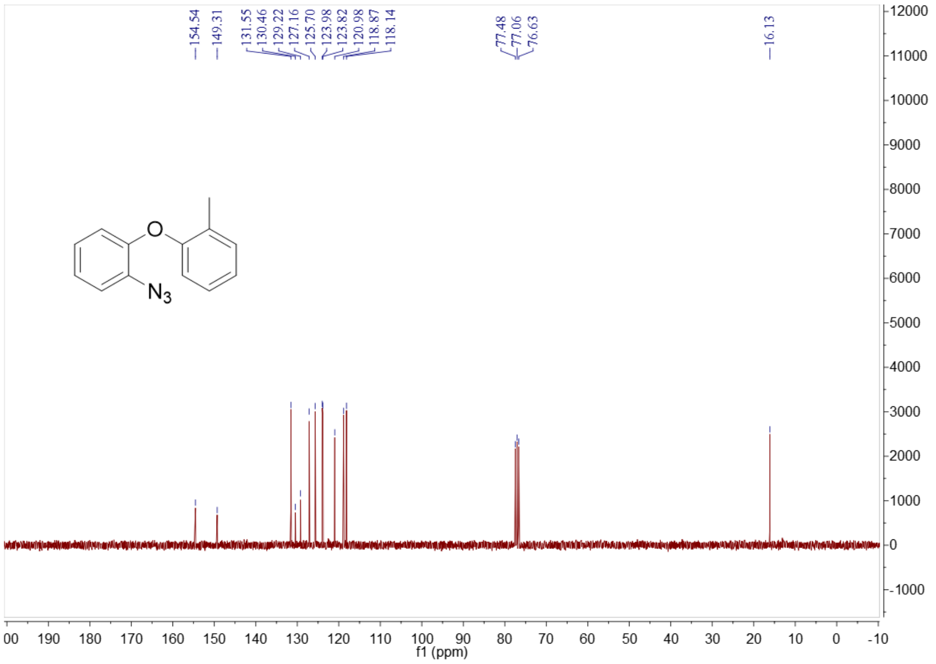

Supplementary Figure 37. 75 MHz ^{13}C NMR of compound 3h in $CDCl_3$

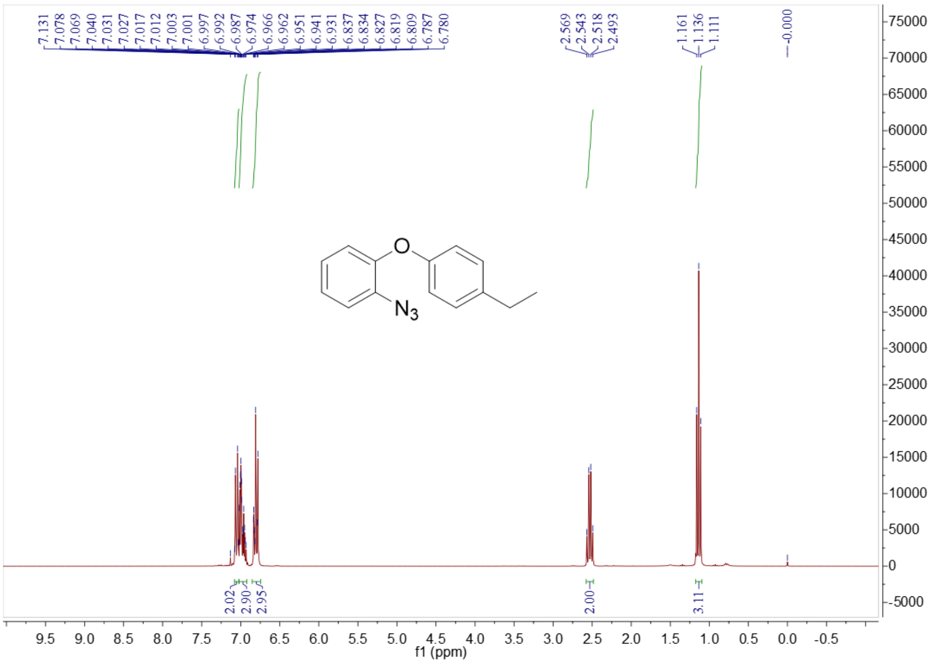

Supplementary Figure 38. 300 MHz ^{1}H NMR of compound 3i in $CDCl_3$

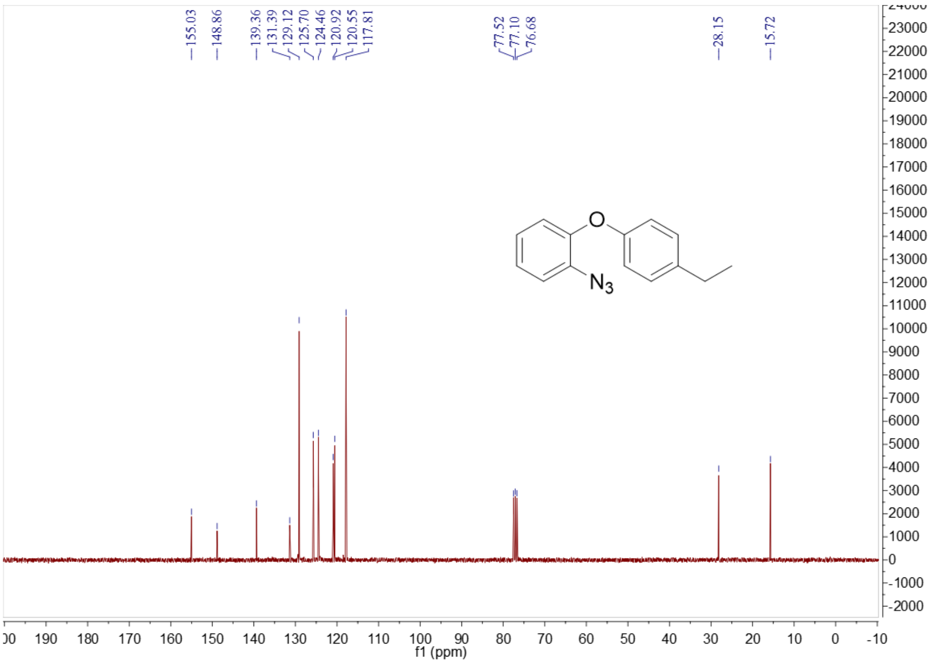

Supplementary Figure 39. 75 MHz ^{13}C NMR of compound 3i in $CDCl_3$

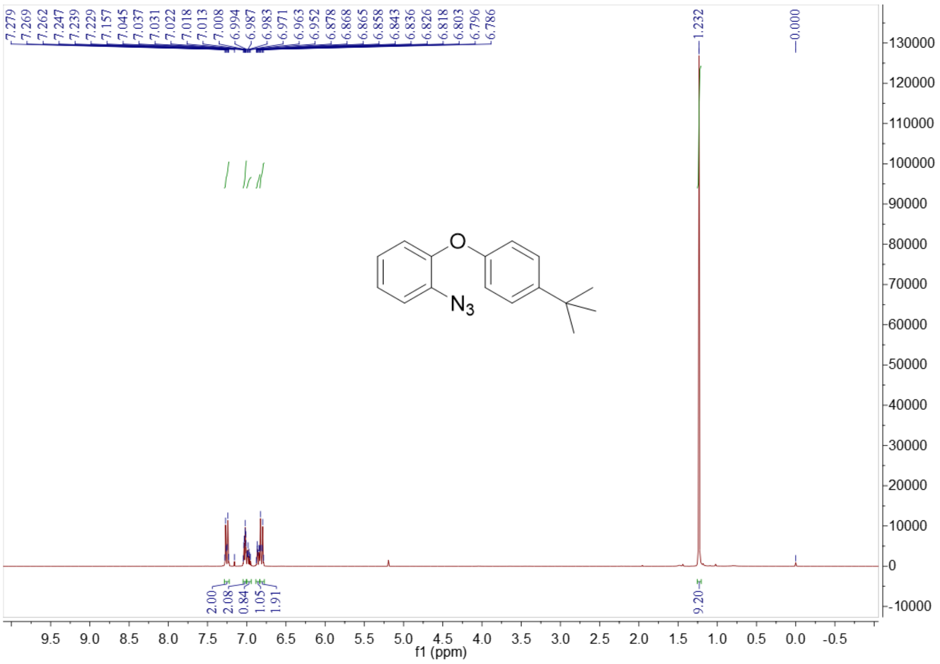

Supplementary Figure 40. 300 MHz ^{1}H NMR of compound 3j in $CDCl_3$

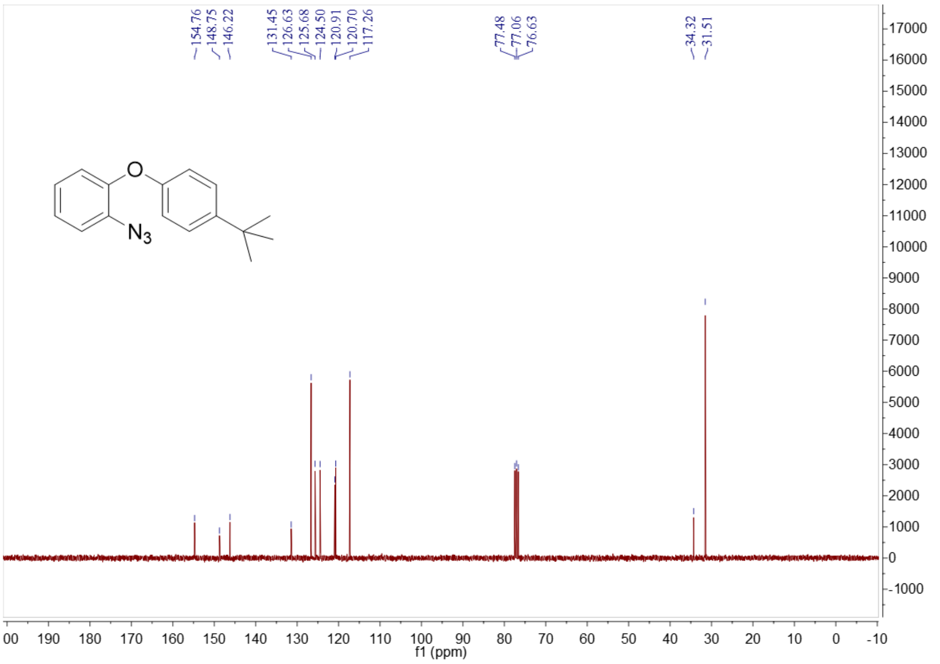

Supplementary Figure 41. 75 MHz ^{13}C NMR of compound 3j in $CDCl_3$

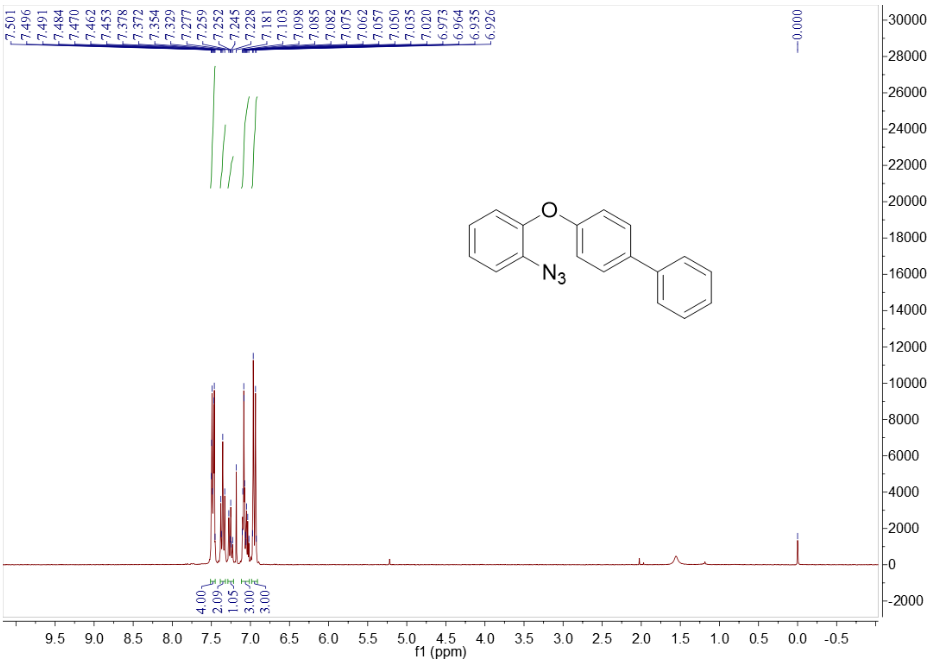

Supplementary Figure 42. 300 MHz ^{1}H NMR of compound 3k in $CDCl_3$

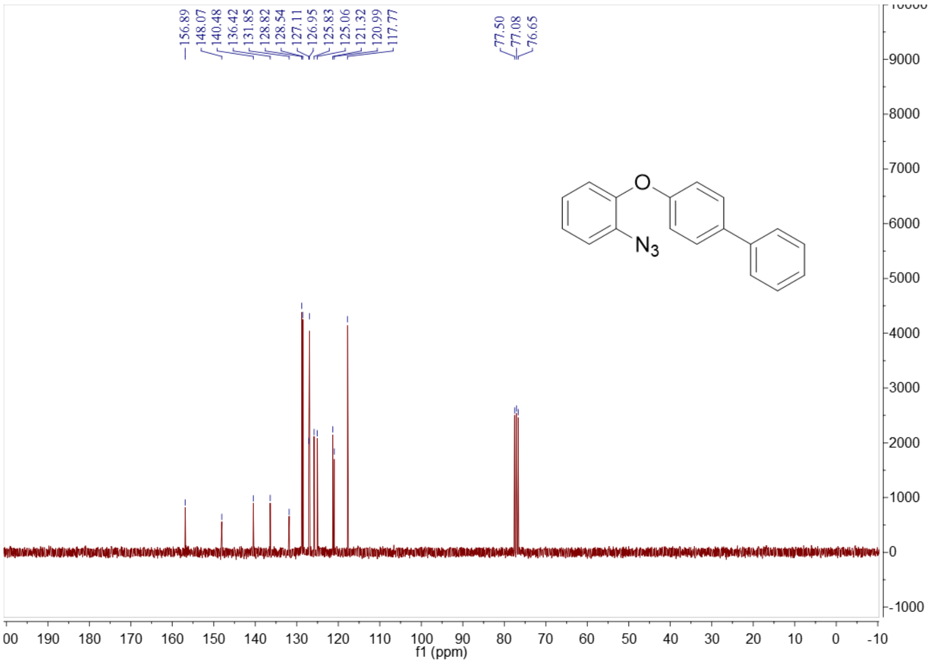

Supplementary Figure 43. 75 MHz ^{13}C NMR of compound 3k in $CDCl_3$

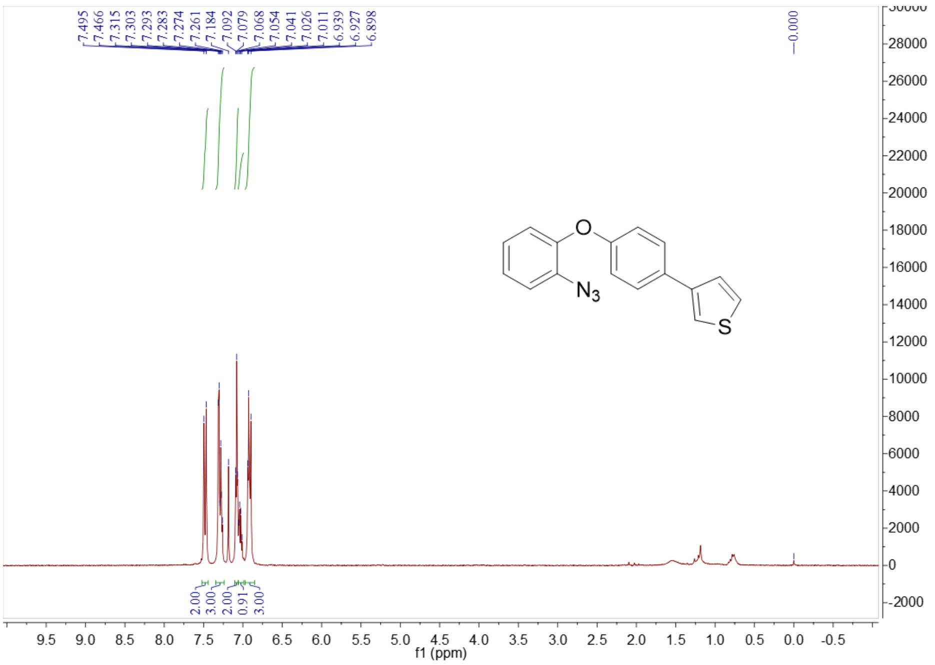

Supplementary Figure 44. 300 MHz ^{1}H NMR of compound 3l in $CDCl_3$

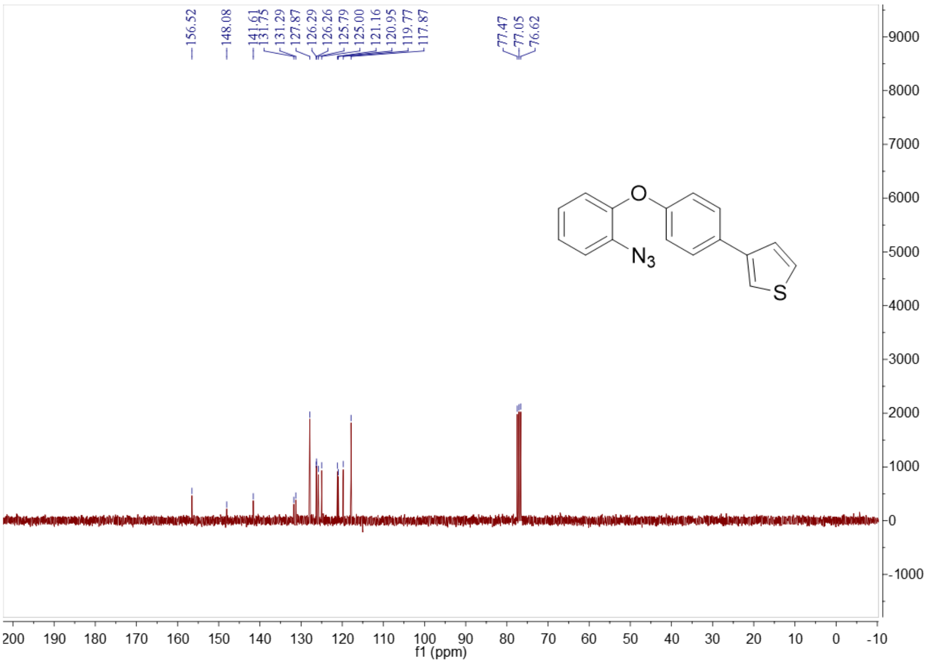

Supplementary Figure 45. 75 MHz ^{13}C NMR of compound 3l in $CDCl_3$

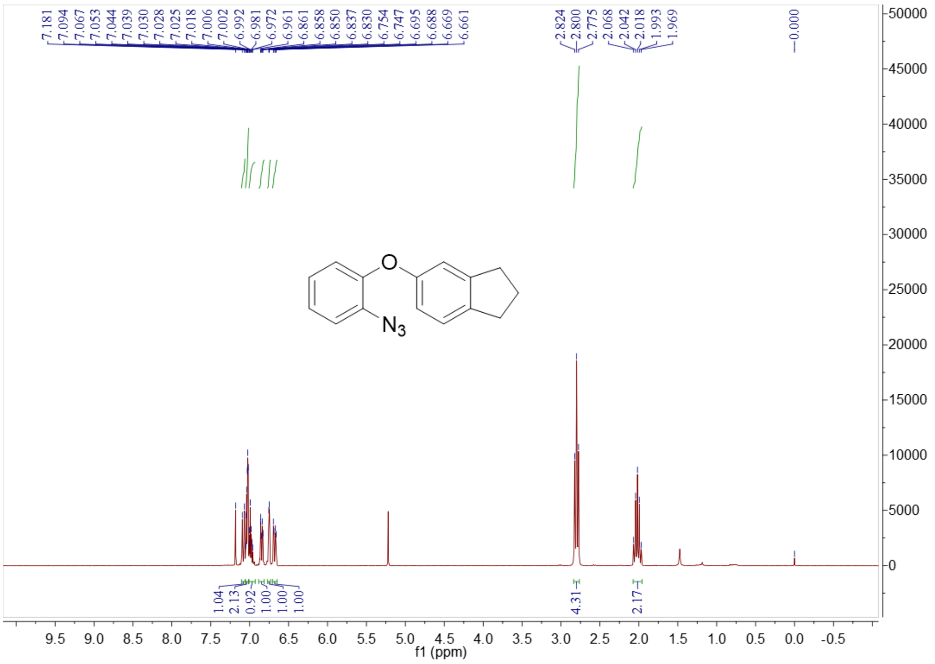

Supplementary Figure 46. 300 MHz ^{1}H NMR of compound 3m in $CDCl_3$

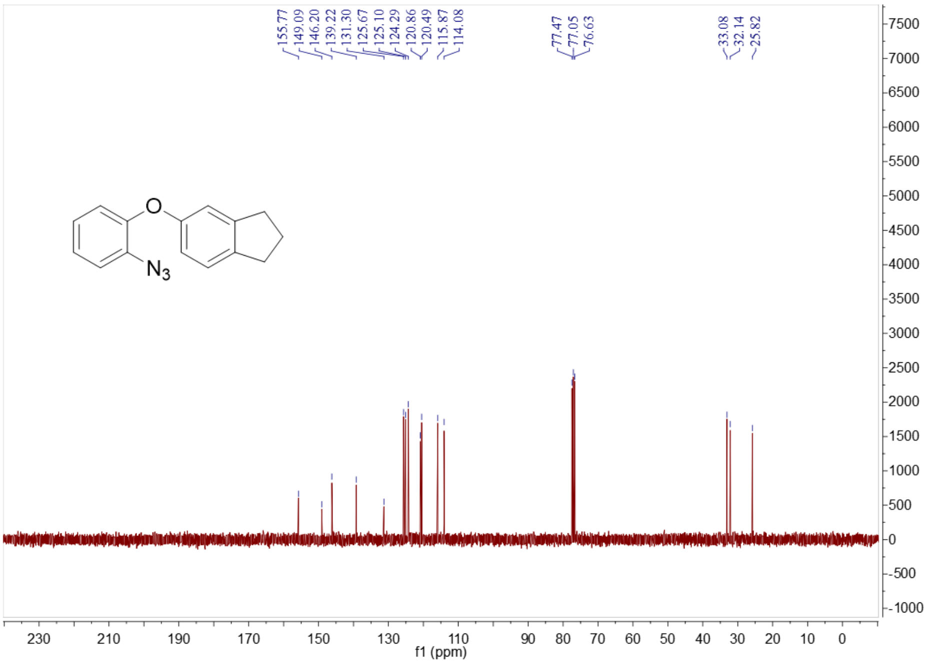

Supplementary Figure 47. 75 MHz ^{13}C NMR of compound 3m in $CDCl_3$

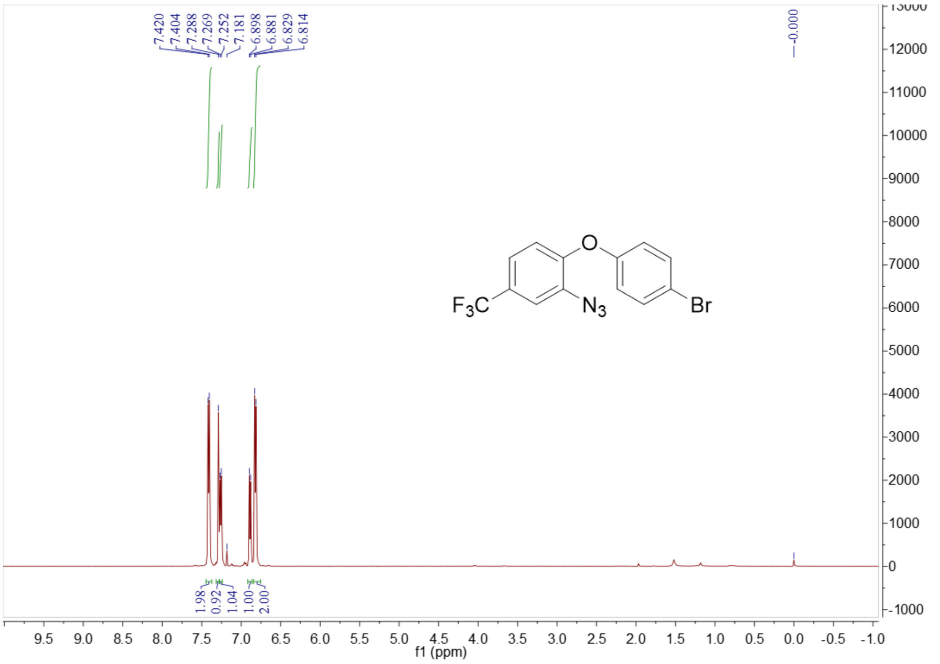

Supplementary Figure 48. 500 MHz ^{1}H NMR of compound 3n in $CDCl_3$

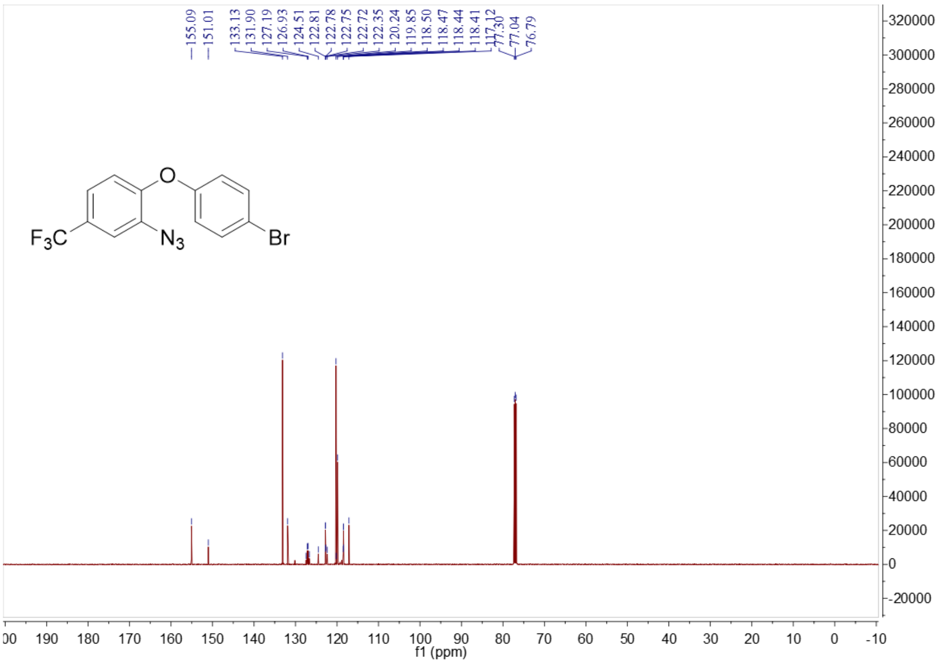

Supplementary Figure 49. 125 MHz ^{13}C NMR of compound 3n in $CDCl_3$

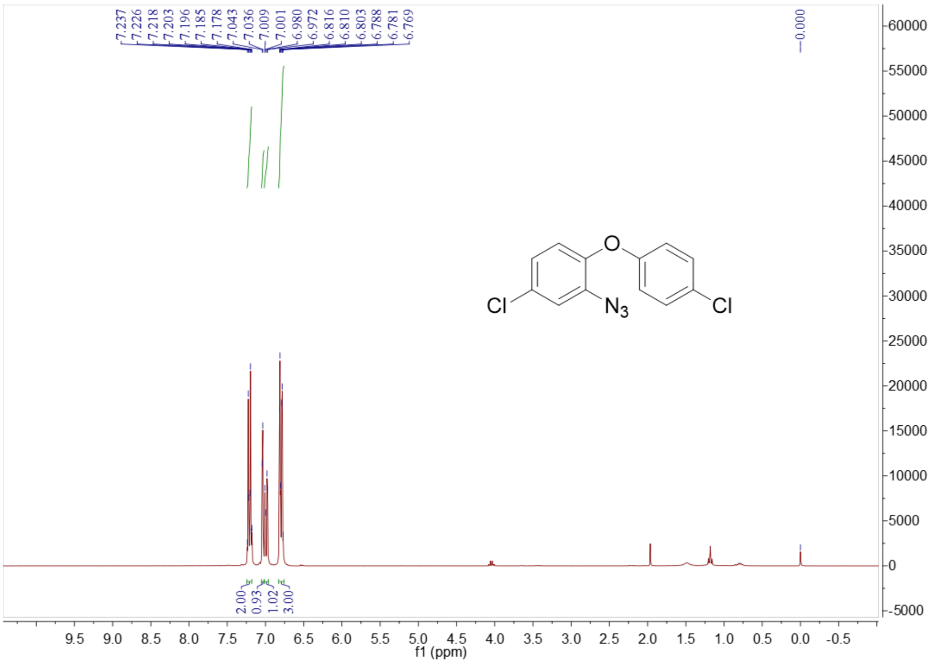

Supplementary Figure 50. 300 MHz ^{1}H NMR of compound 3o in CDCl$_3$

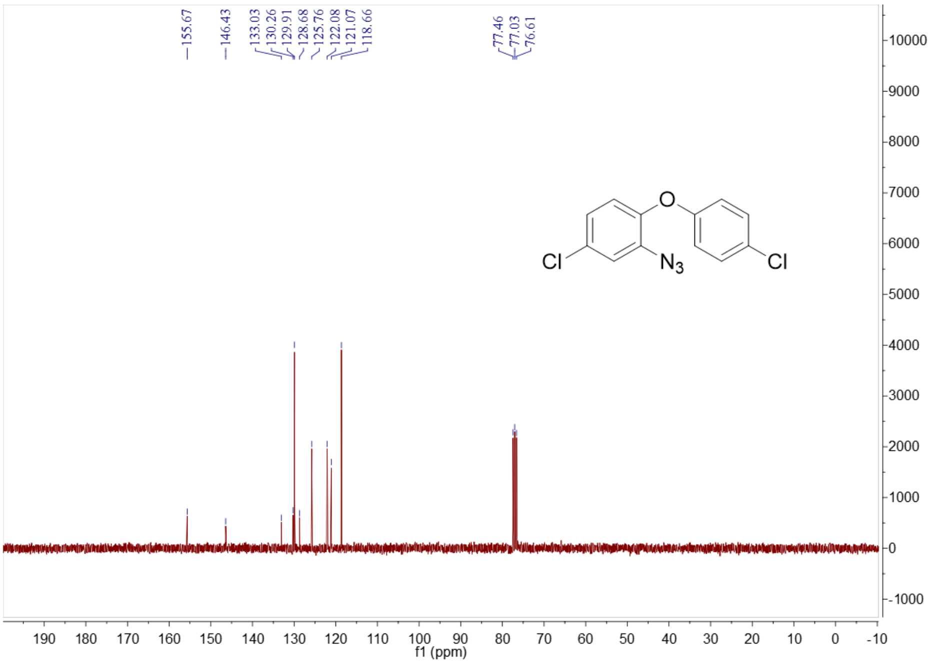

Supplementary Figure 51. 75 MHz ^{13}C NMR of compound 3o in CDCl$_3$

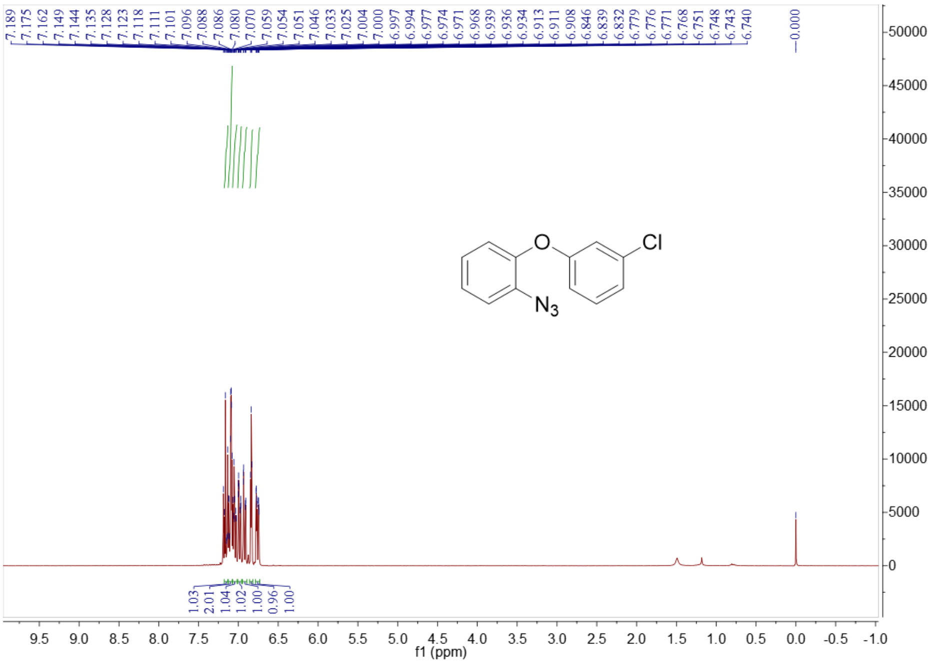

Supplementary Figure 52. 300 MHz ^{1}H NMR of compound 3p in $CDCl_3$

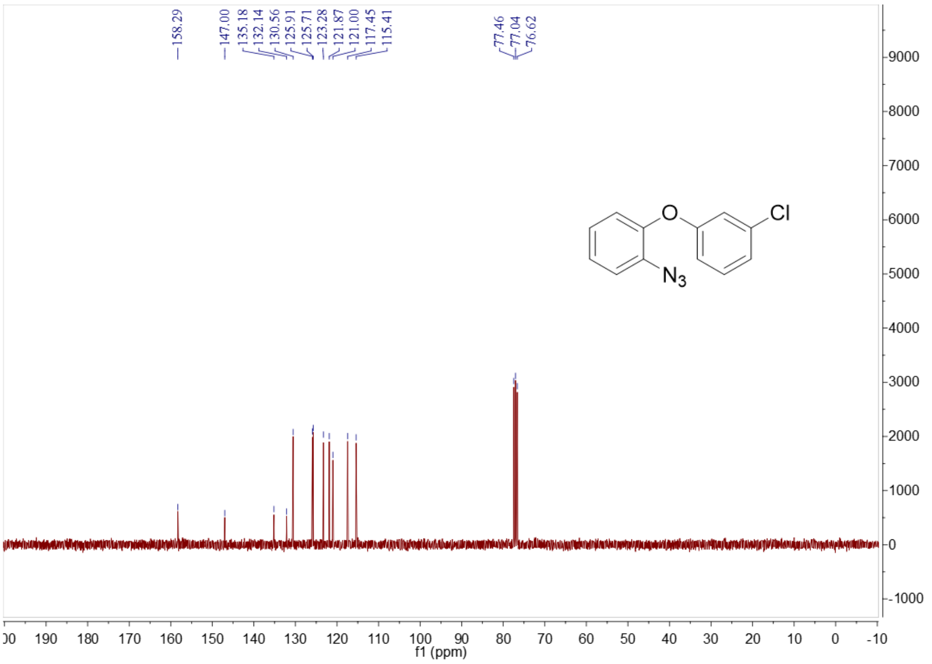

Supplementary Figure 53. 75 MHz ^{13}C NMR of compound 3p in $CDCl_3$

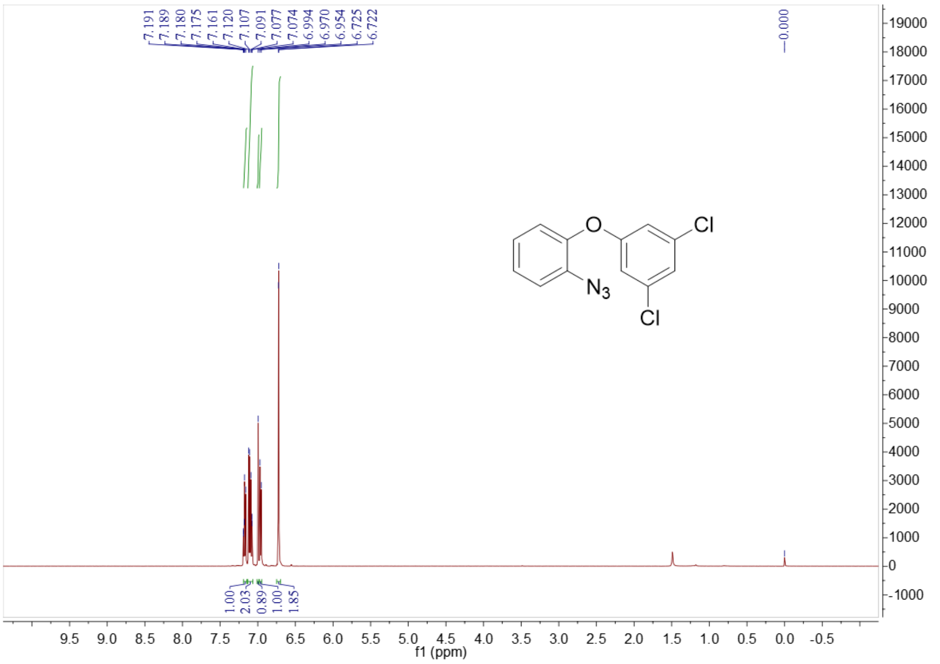

Supplementary Figure 54. 500 MHz ^{1}H NMR of compound 3q in $CDCl_3$

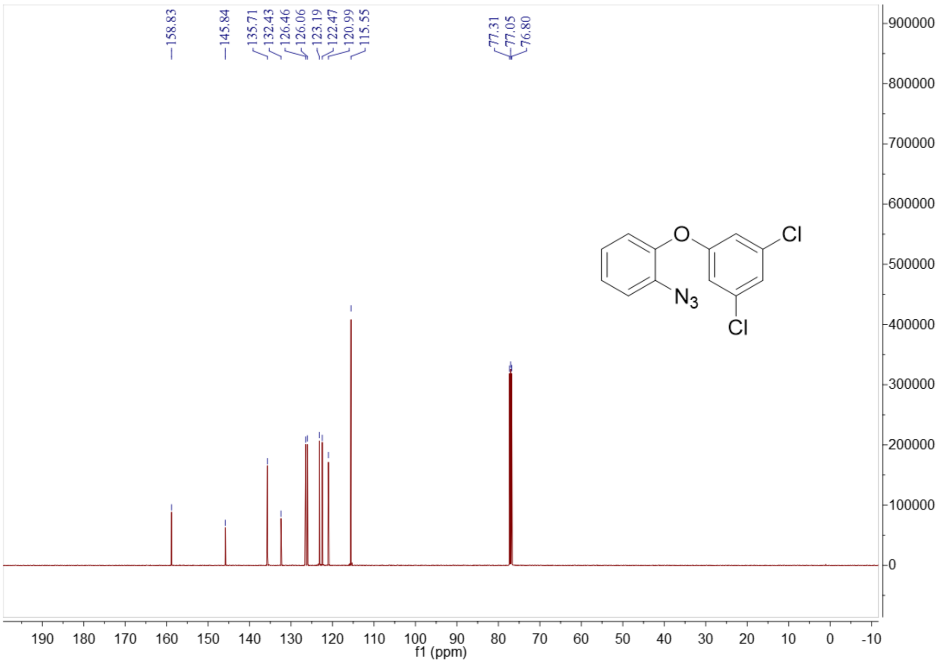

Supplementary Figure 55. 125 MHz ^{13}C NMR of compound 3q in $CDCl_3$

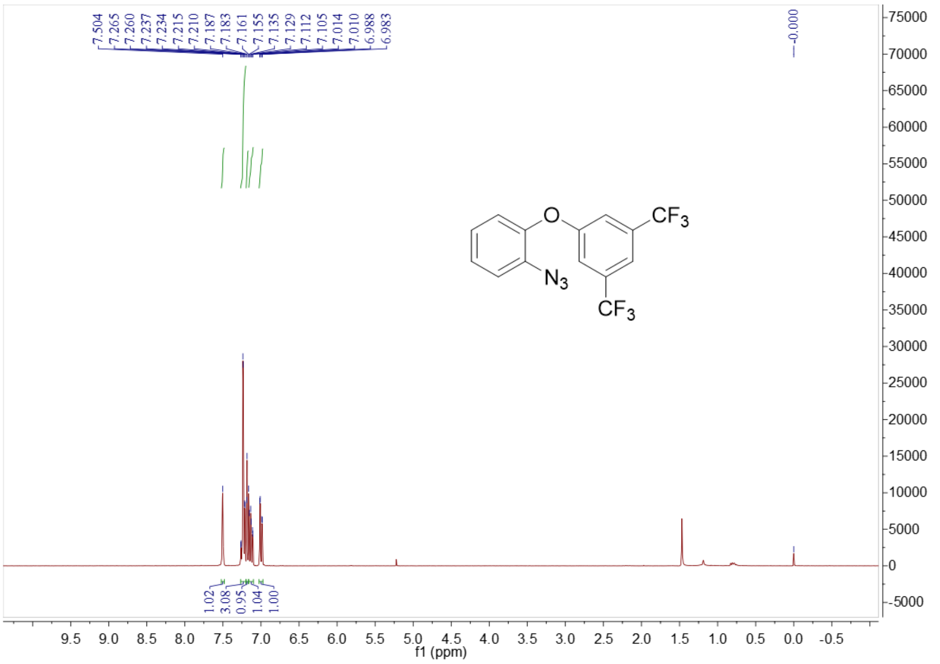

Supplementary Figure 56. 300 MHz ^{1}H NMR of compound 3r in $CDCl_3$

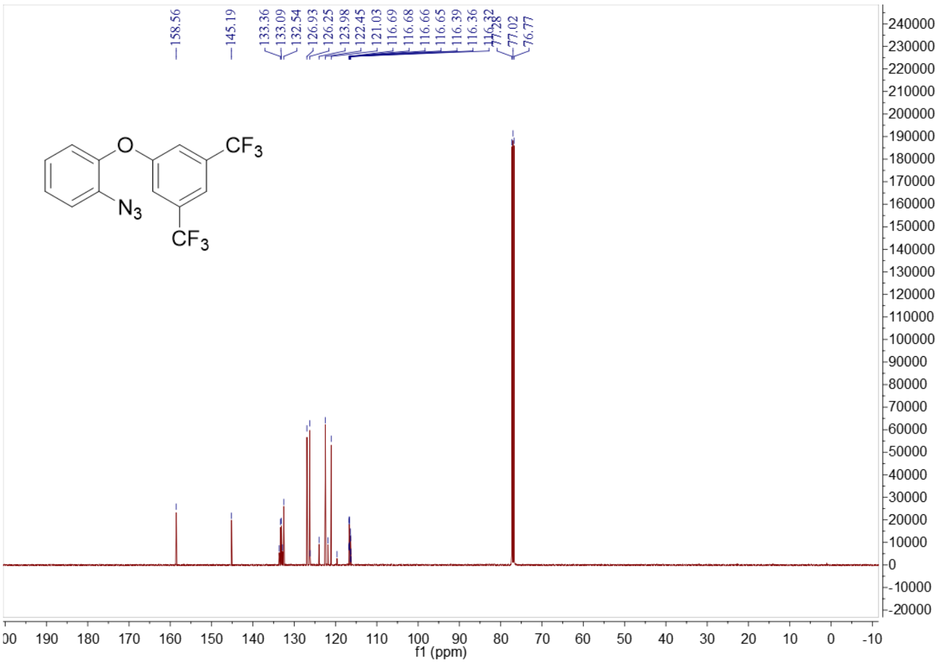

Supplementary Figure 57. 75 MHz ^{13}C NMR of compound 3r in $CDCl_3$

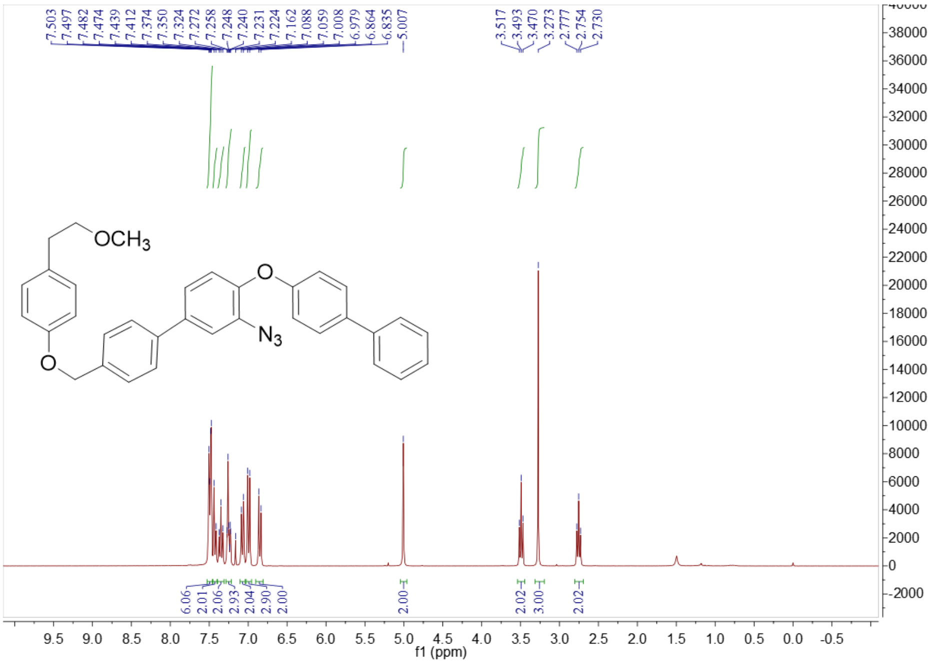

Supplementary Figure 58. 300 MHz ^{1}H NMR of compound 3s in $CDCl_3$

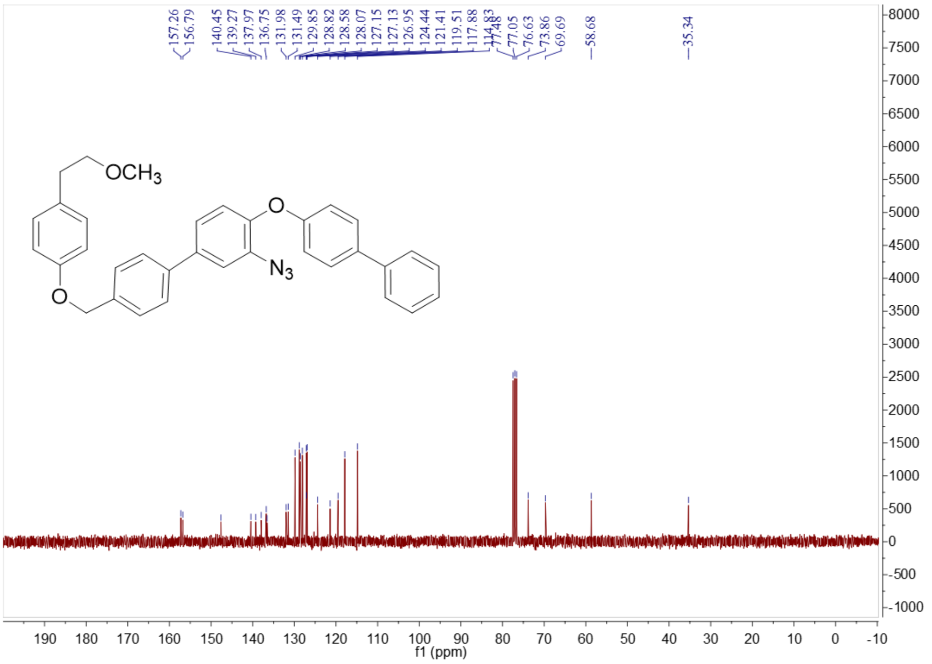

Supplementary Figure 59. 75 MHz ^{13}C NMR of compound 3s in $CDCl_3$

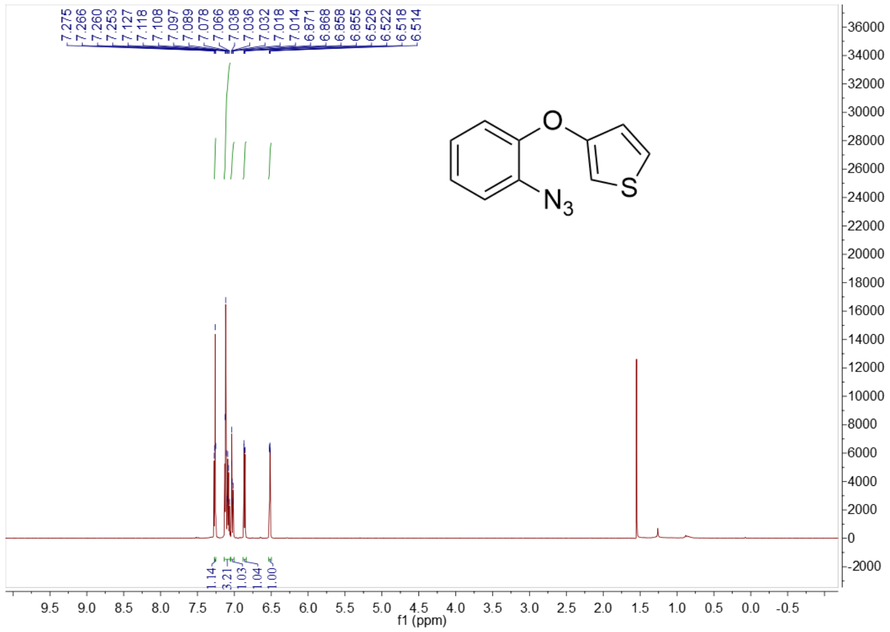

Supplementary Figure 60. 400 MHz ^{1}H NMR of compound 3t in $CDCl_3$

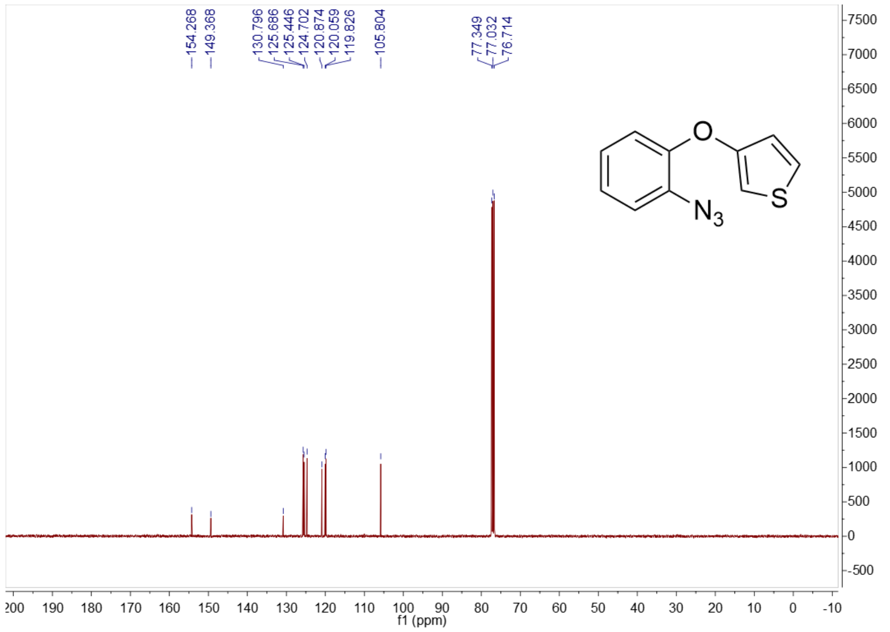

Supplementary Figure 61. 100 MHz ^{13}C NMR of compound 3t in $CDCl_3$

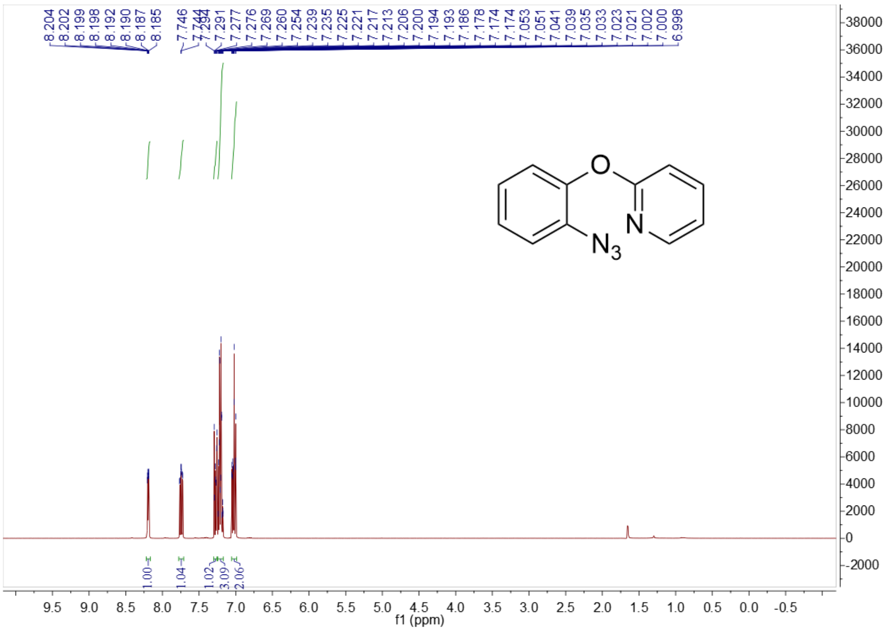

Supplementary Figure 62. 400 MHz ^{1}H NMR of compound 3u in $CDCl_3$

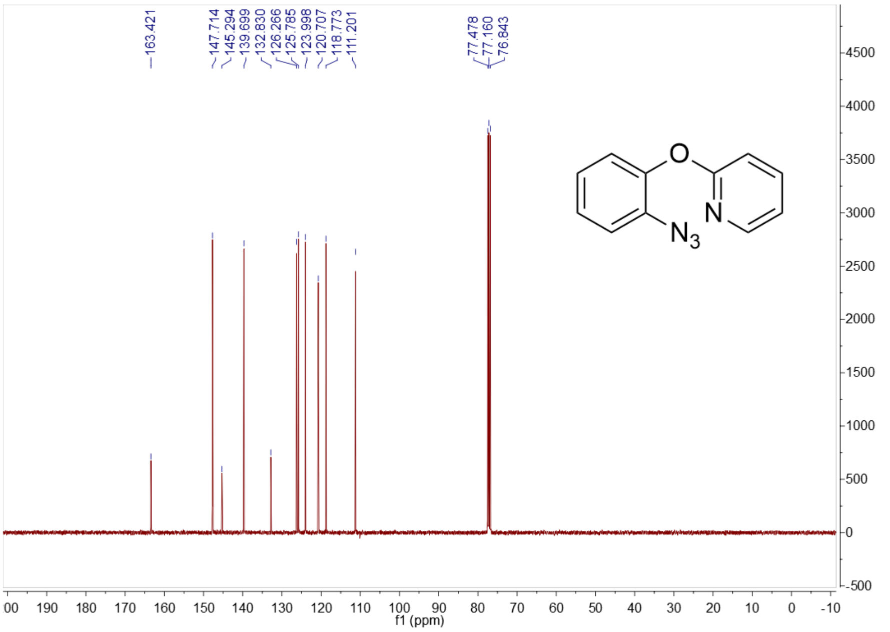

Supplementary Figure 63. 100 MHz ^{13}C NMR of compound 3u in $CDCl_3$

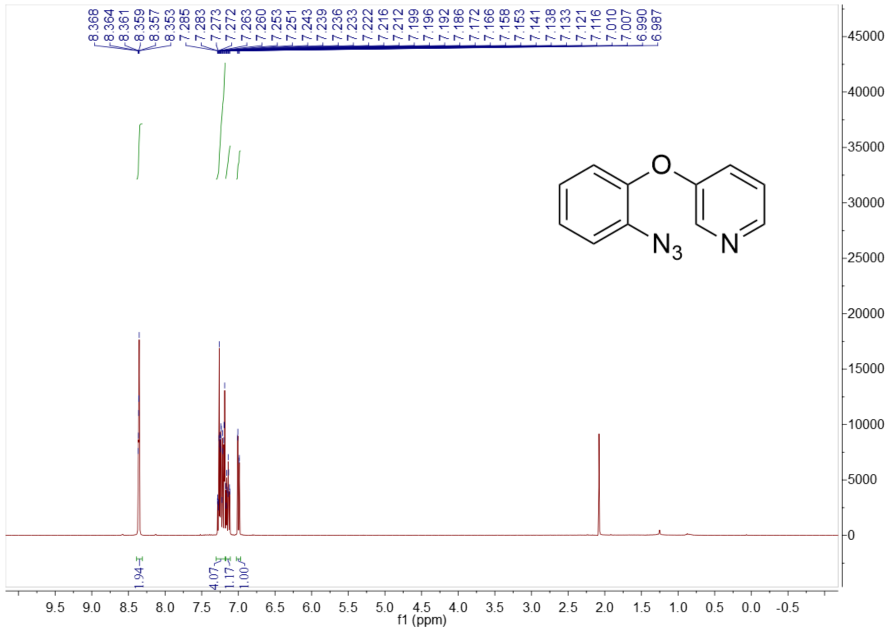

Supplementary Figure 64. 400 MHz ^{1}H NMR of compound 3v in CDCl$_3$

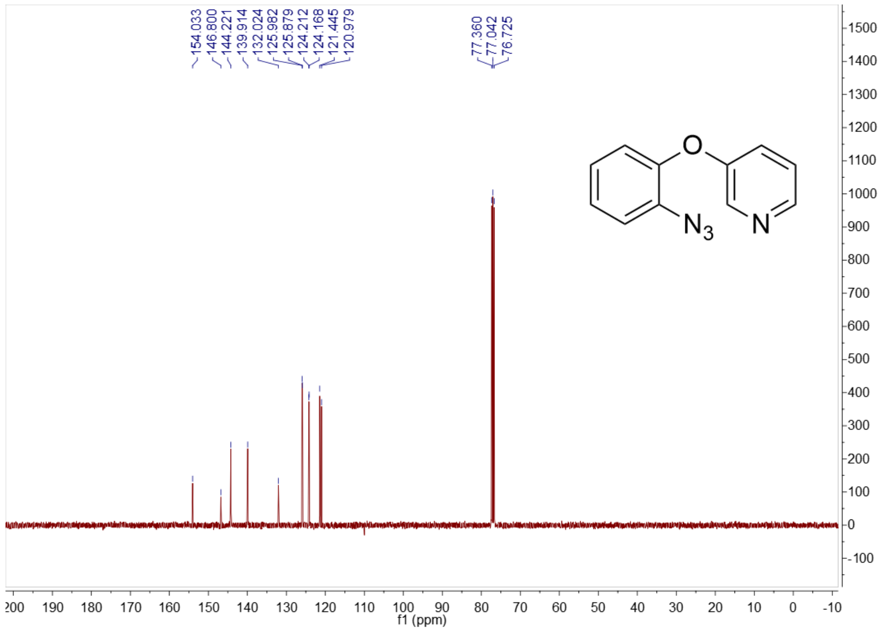

Supplementary Figure 65. 100 MHz ^{13}C NMR of compound 3v in CDCl$_3$

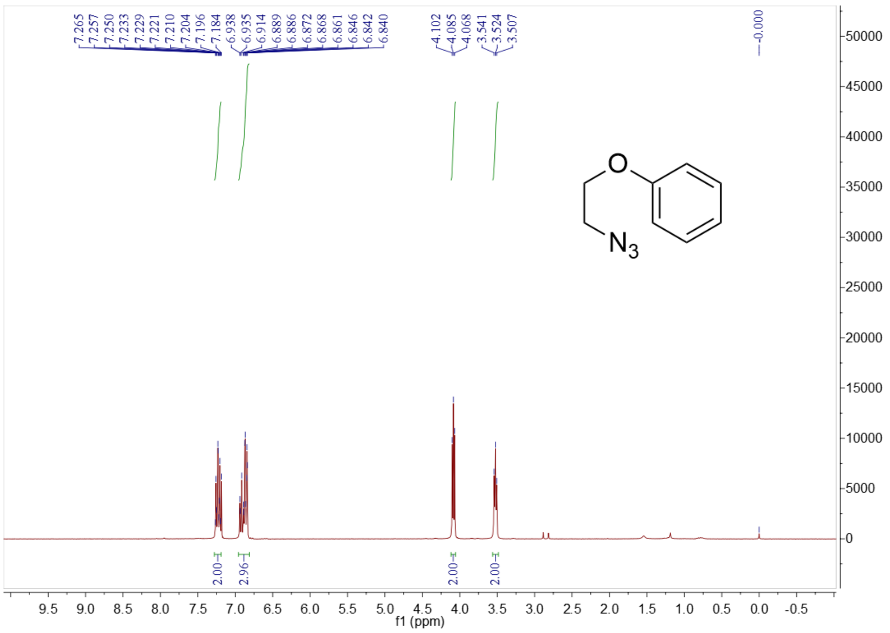

Supplementary Figure 66. 300 MHz ^{1}H NMR of compound 3w in $CDCl_3$

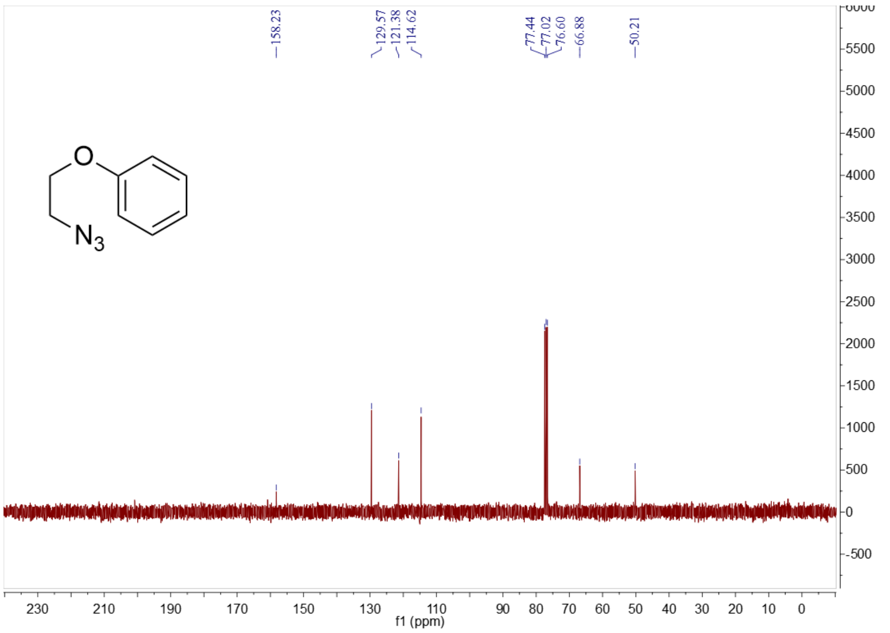

Supplementary Figure 67. 75 MHz ^{13}C NMR of compound 3w in $CDCl_3$

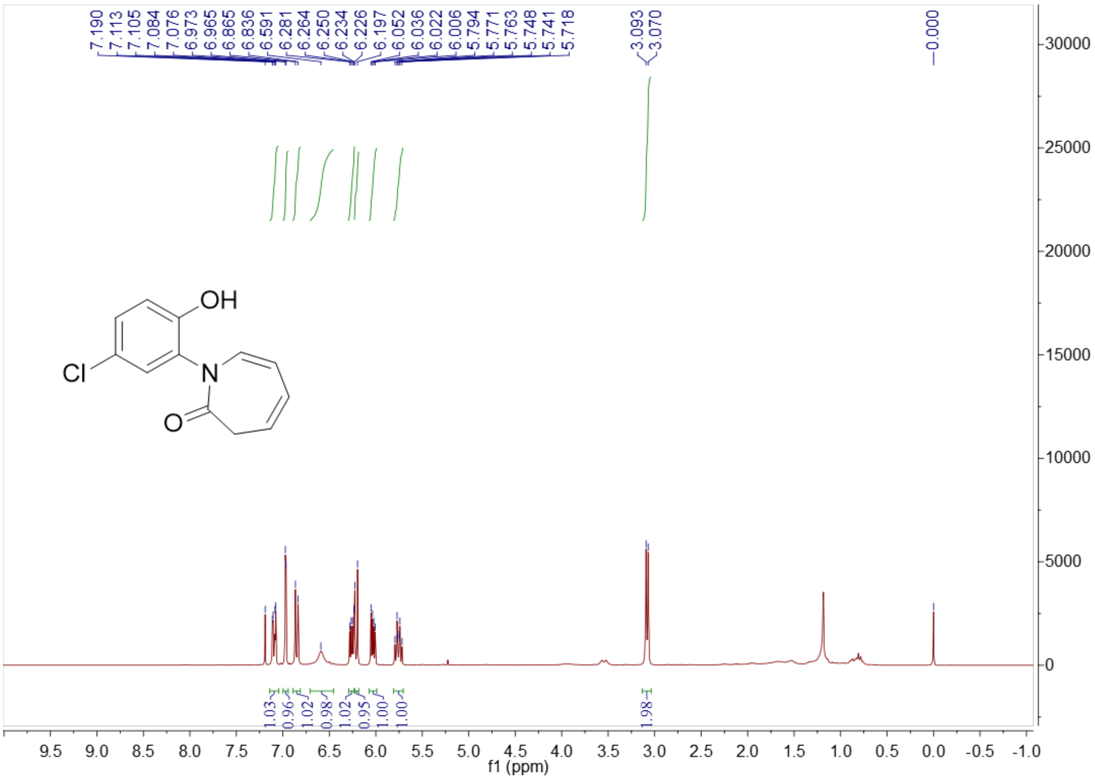

Supplementary Figure 68. 300 MHz ^{1}H NMR of compound 5a in $CDCl_3$

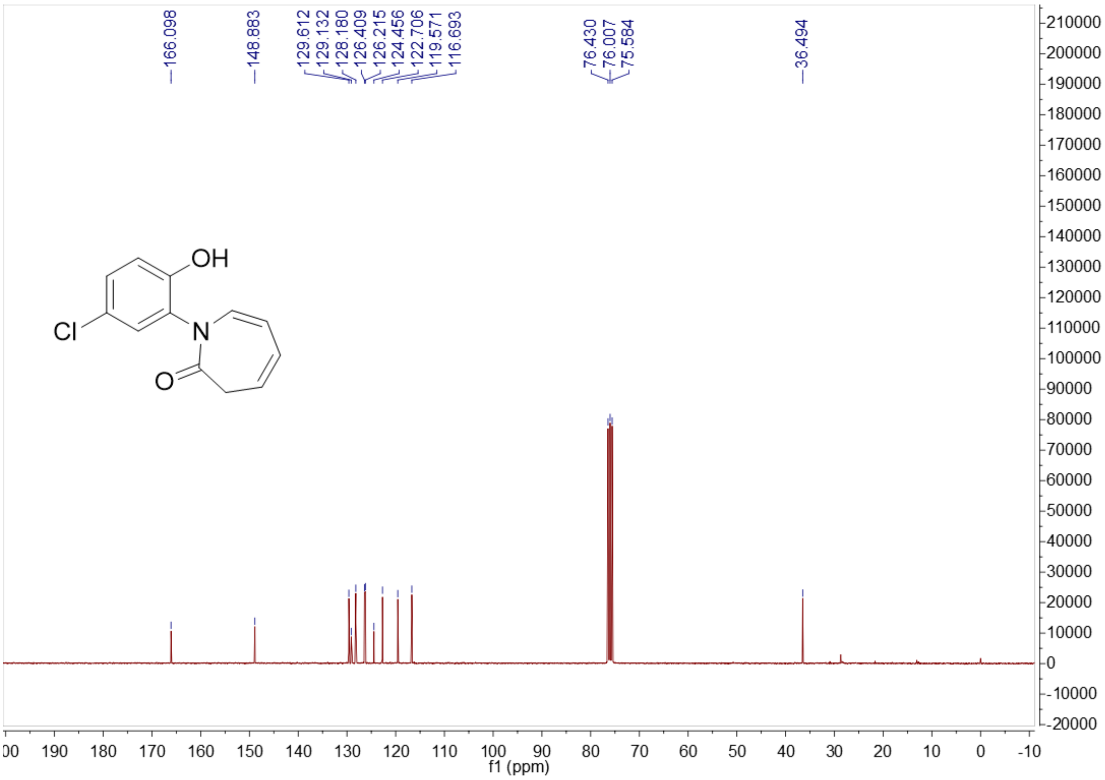

Supplementary Figure 69. 75 MHz ^{13}C NMR of compound 5a in $CDCl_3$

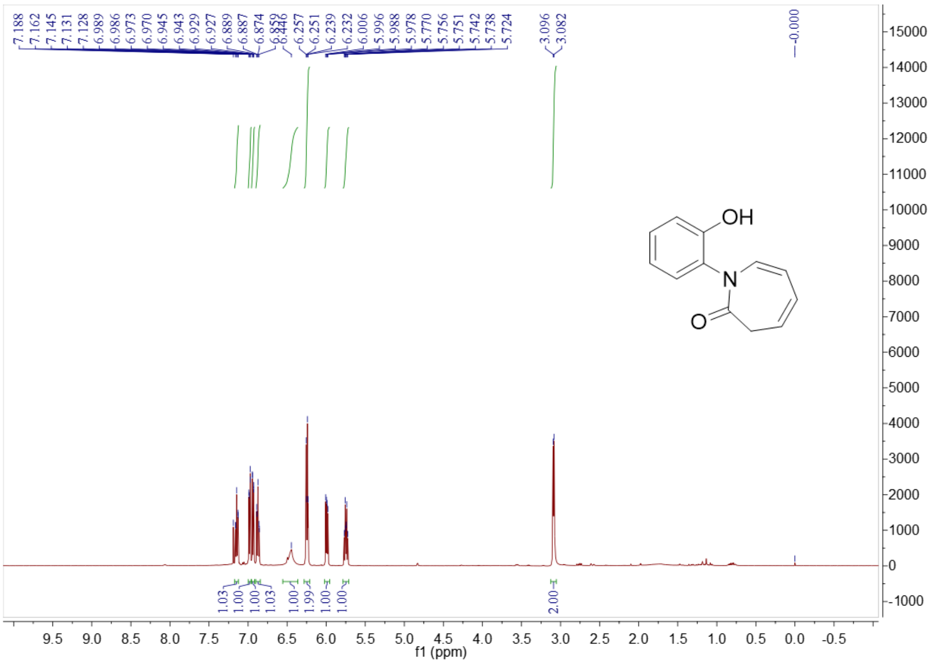

Supplementary Figure 70. 500 MHz ^{1}H NMR of compound 5b in $CDCl_3$

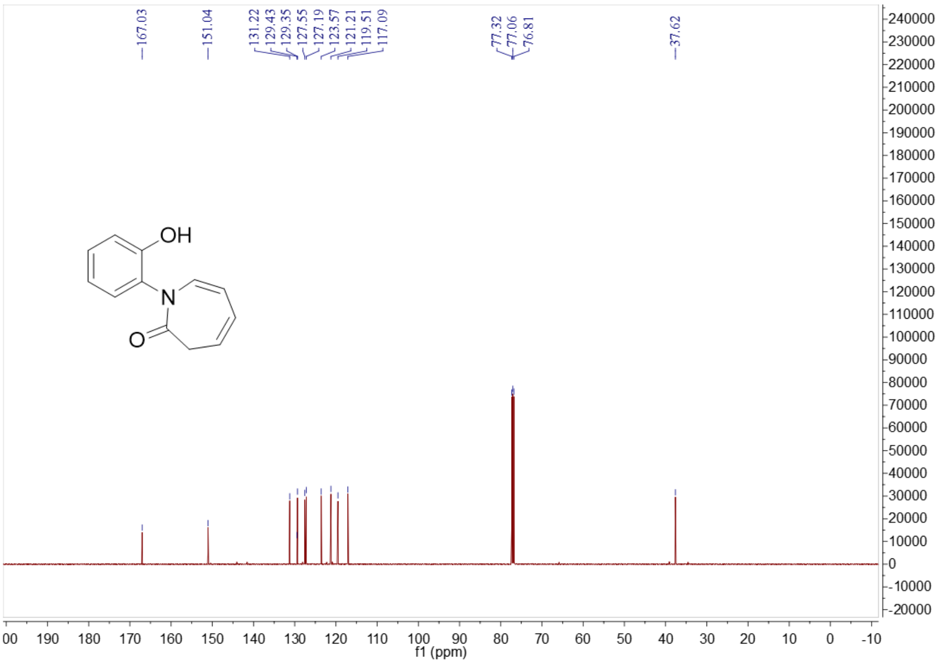

Supplementary Figure 71. 125 MHz ^{13}C NMR of compound 5b in $CDCl_3$

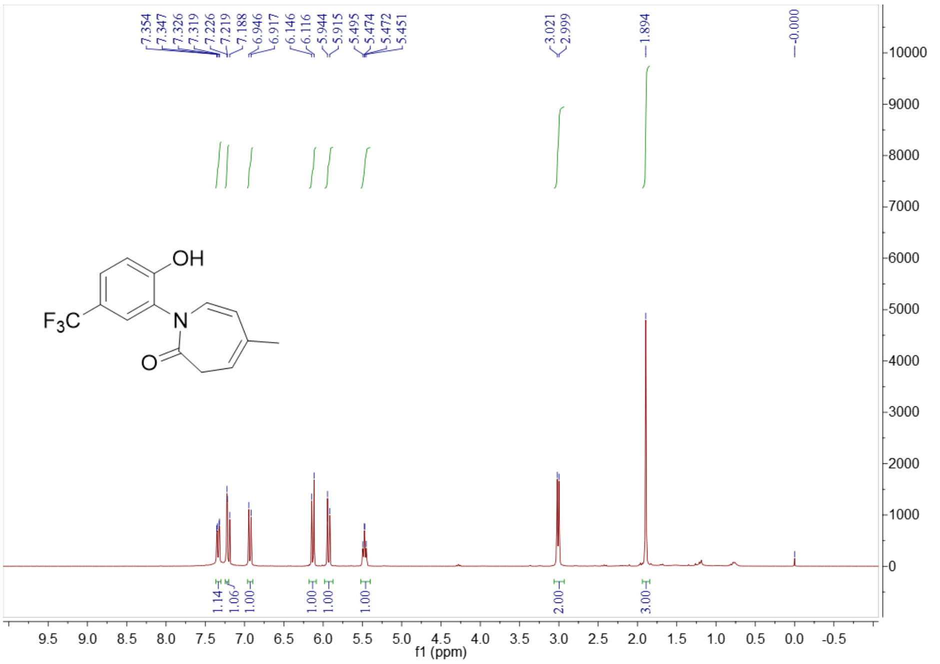

Supplementary Figure 72. 300 MHz ^{1}H NMR of compound 5c in $CDCl_3$

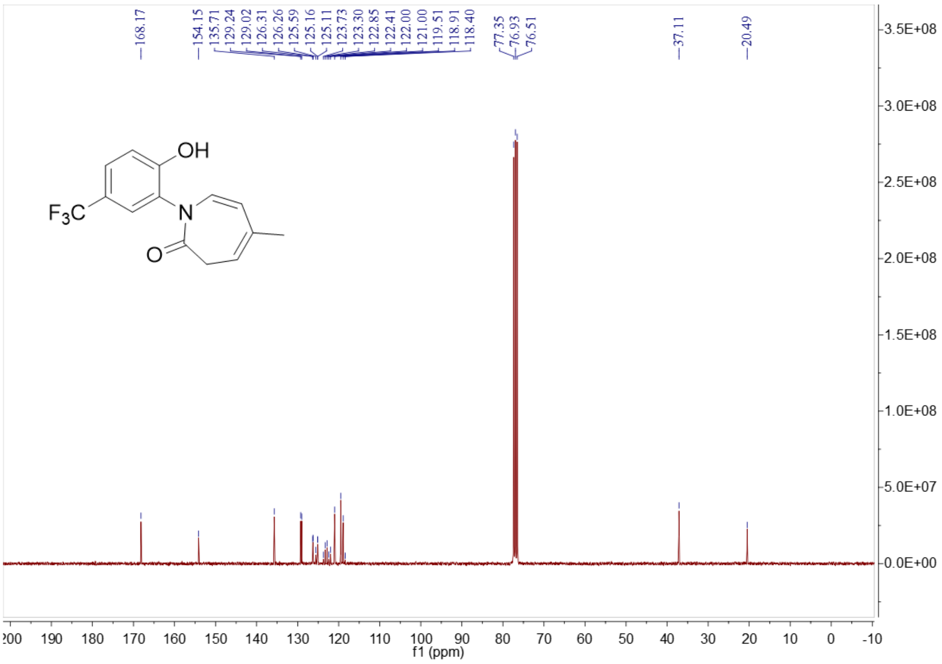

Supplementary Figure 73. 75 MHz ^{13}C NMR of compound 5c in $CDCl_3$

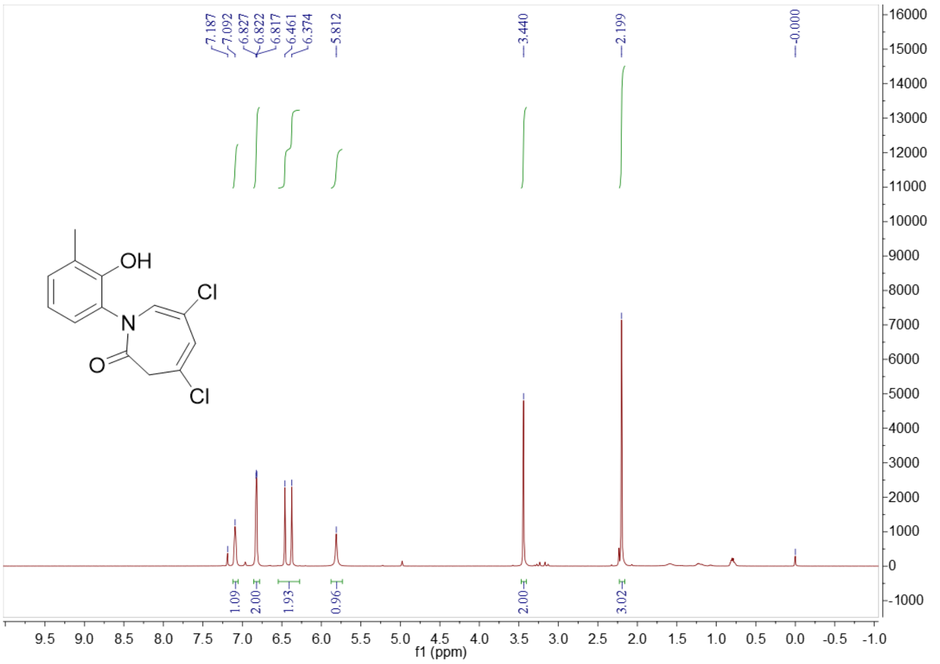

Supplementary Figure 74. 500 MHz ^{1}H NMR of compound 5d in $CDCl_3$

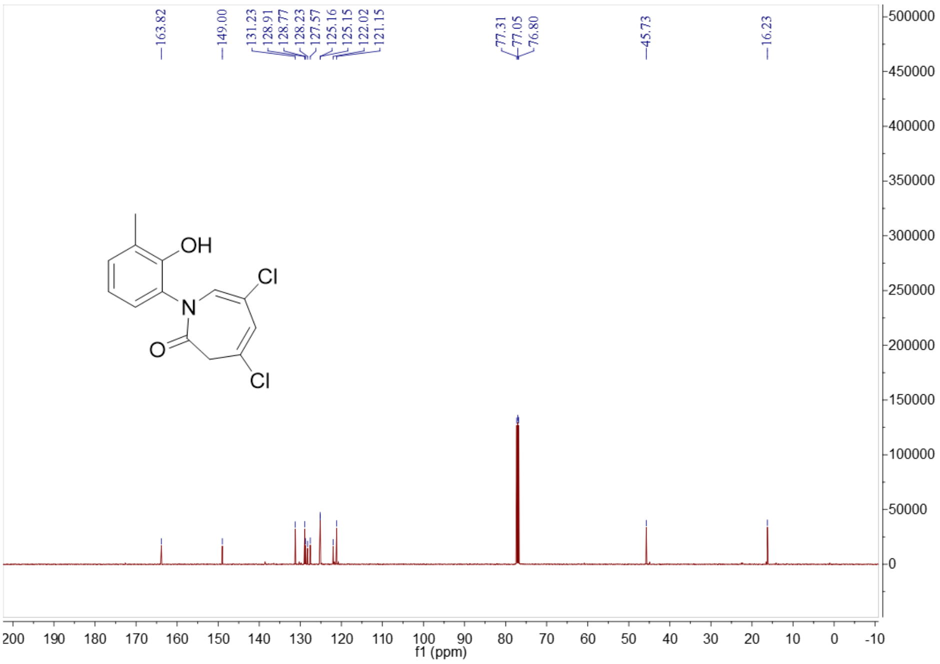

Supplementary Figure 75. 125 MHz ^{13}C NMR of compound 5d in $CDCl_3$

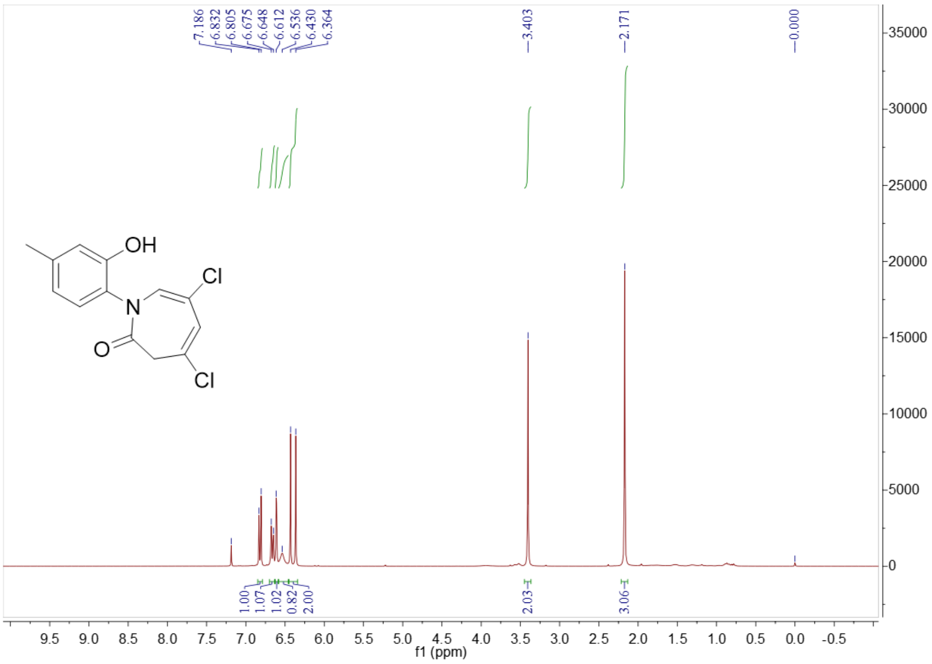

Supplementary Figure 76. 300 MHz ^{1}H NMR of compound 5e in $CDCl_3$

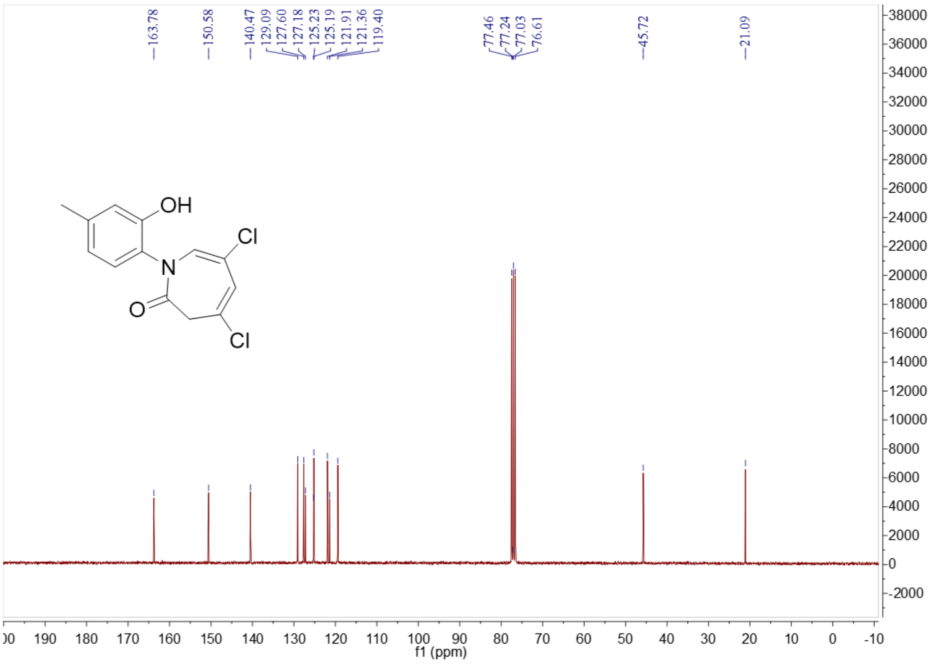

Supplementary Figure 77. 75 MHz ^{13}C NMR of compound 5e in $CDCl_3$

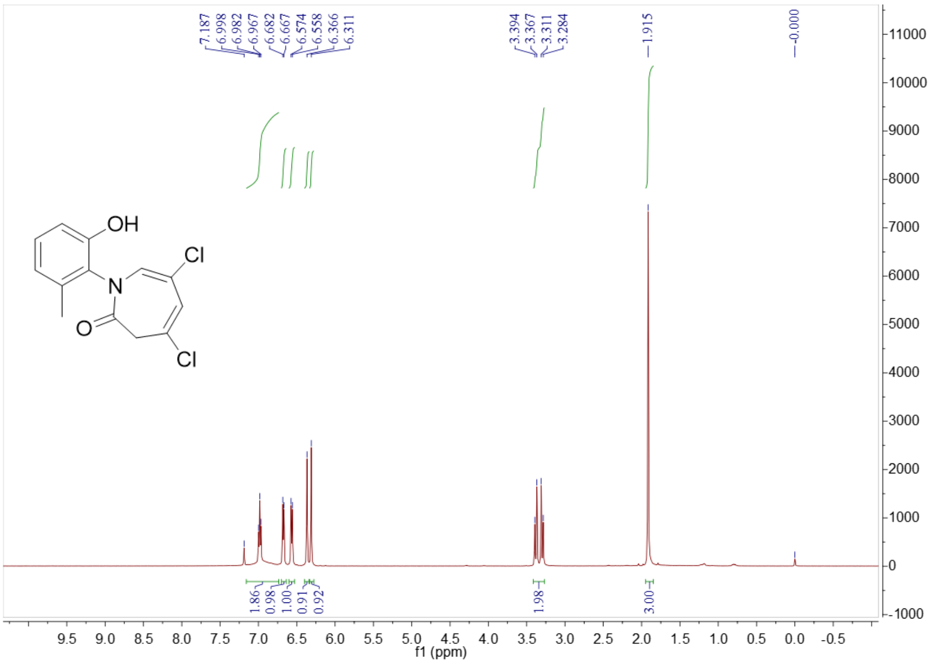

Supplementary Figure 78. 500 MHz ^{1}H NMR of compound 5f in $CDCl_3$

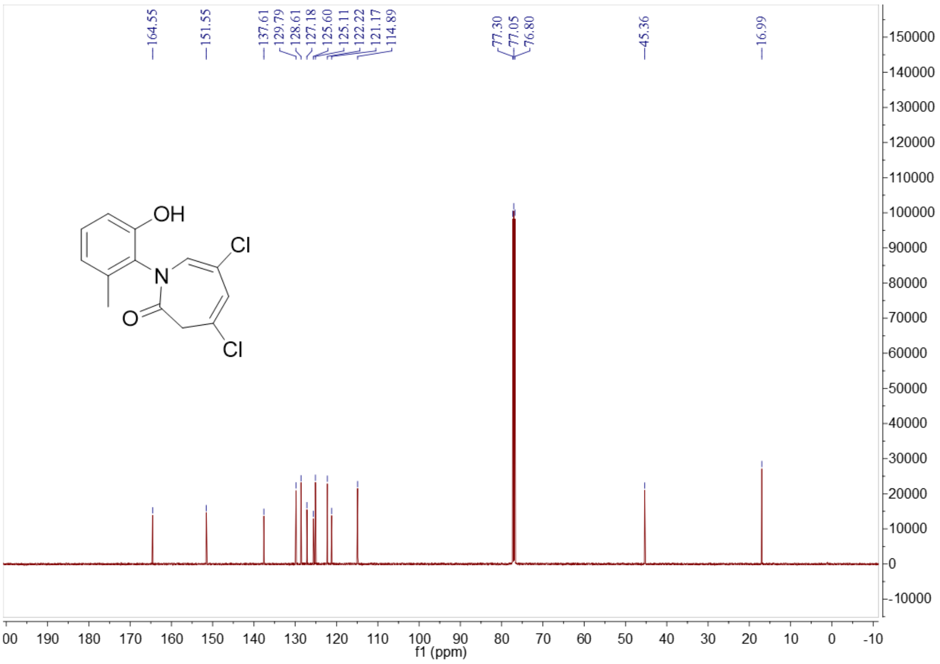

Supplementary Figure 79. 125 MHz ^{13}C NMR of compound 5f in $CDCl_3$

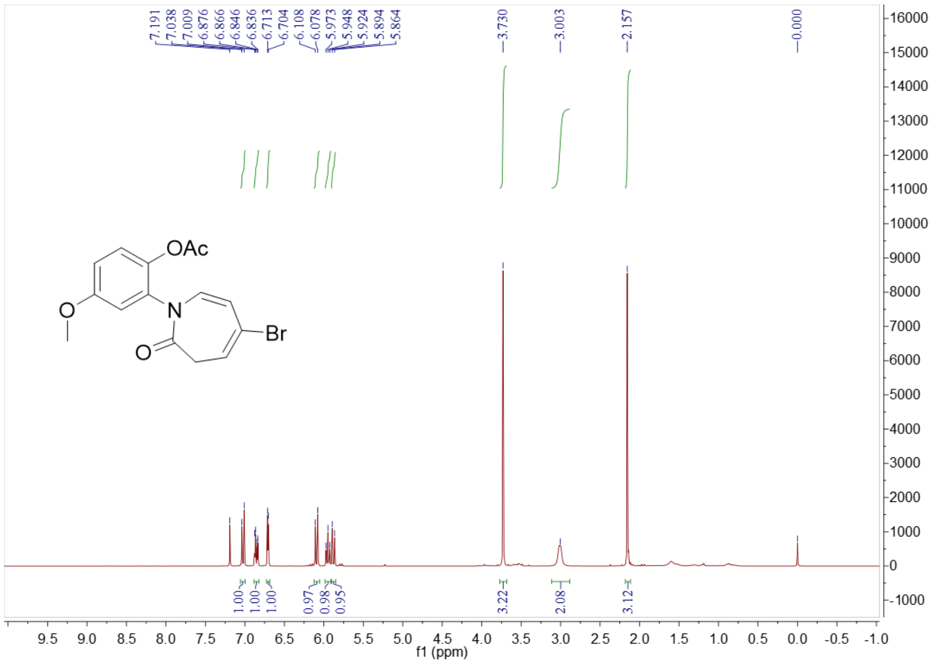

Supplementary Figure 80. 300 MHz ^{1}H NMR of compound 5g' in $CDCl_3$

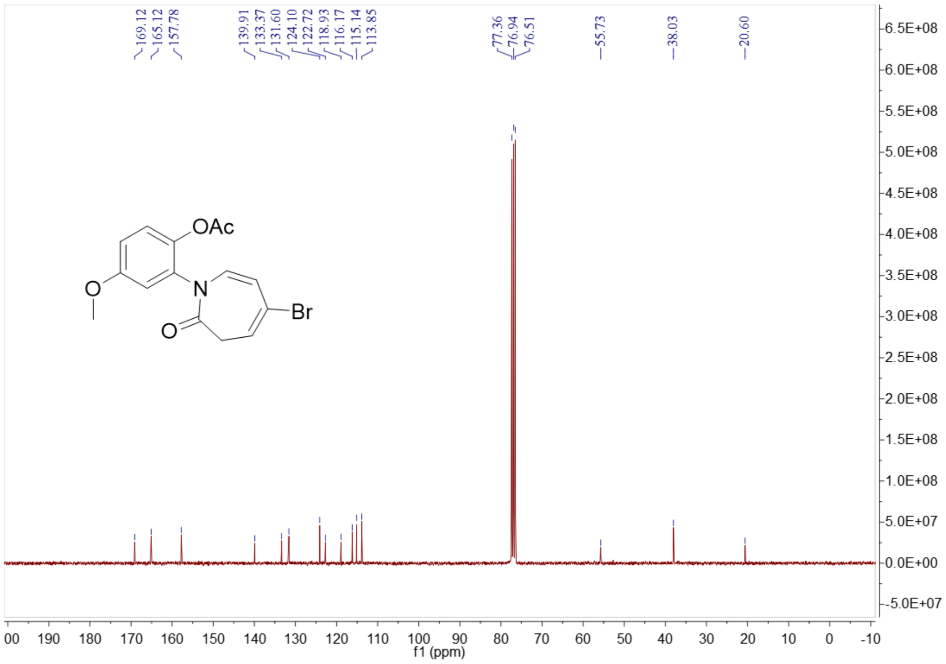

Supplementary Figure 81. 75 MHz ^{13}C NMR of compound 5g' in $CDCl_3$

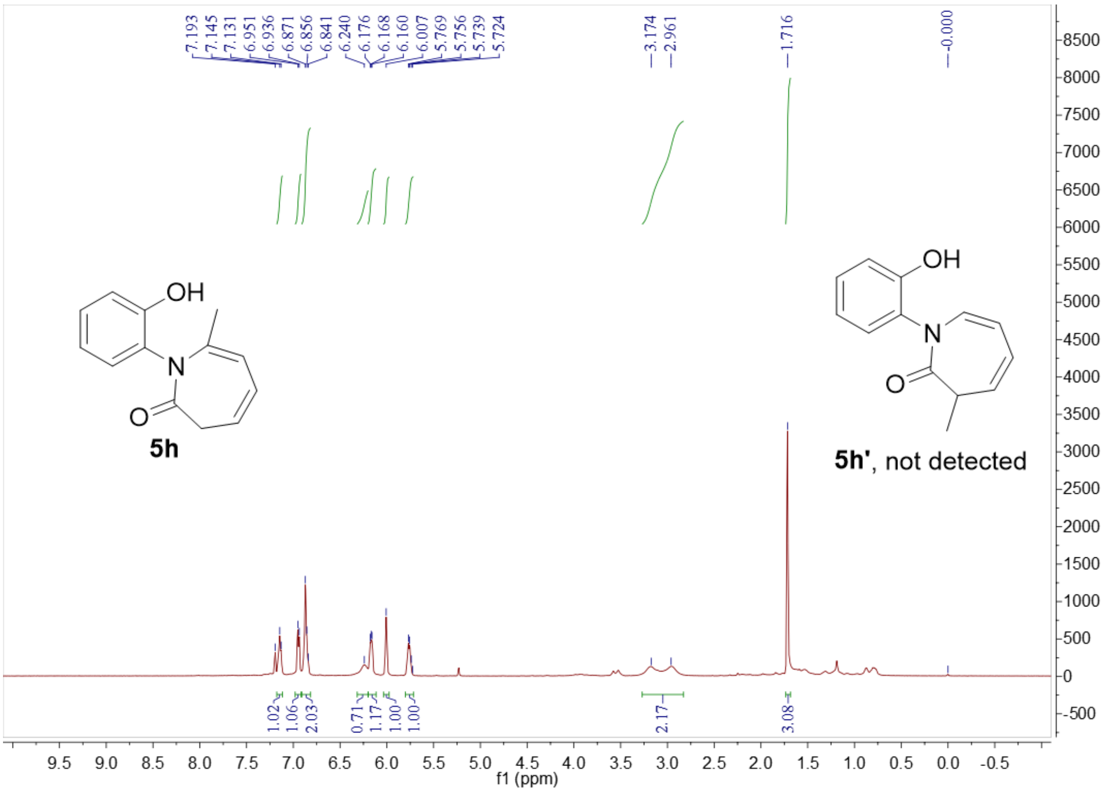

Supplementary Figure 82. 500 MHz ^{1}H NMR of compound 5h in $CDCl_3$

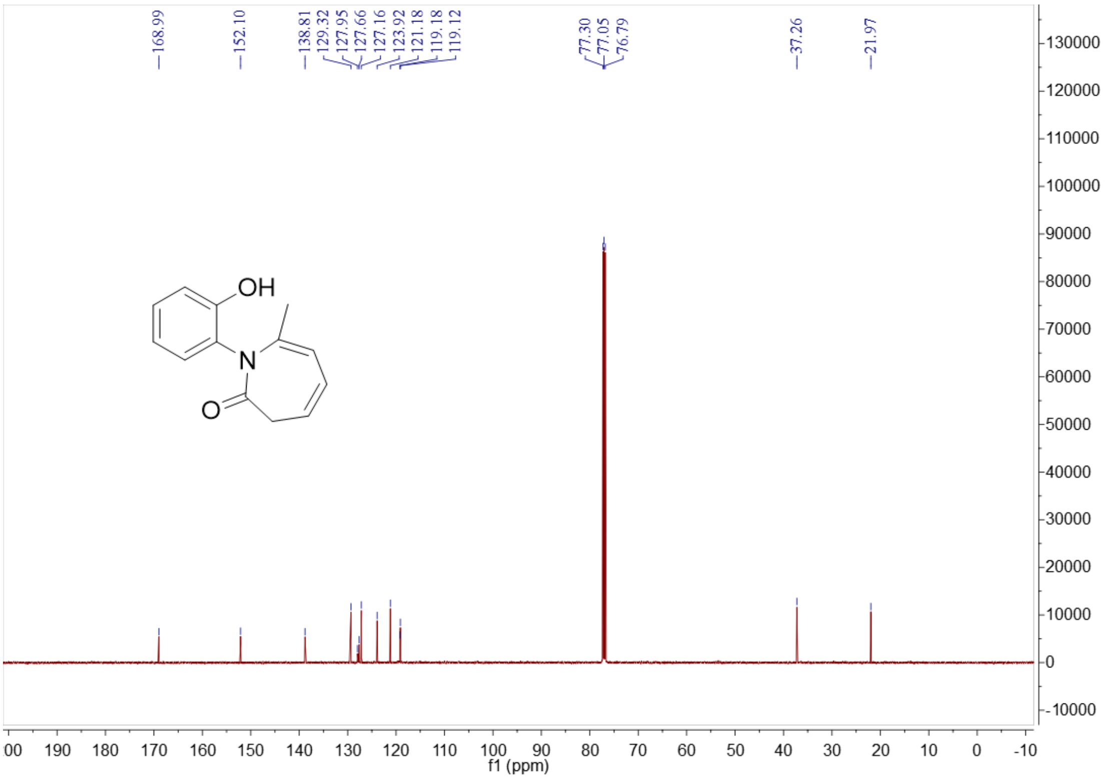

Supplementary Figure 83. 125 MHz ^{13}C NMR of compound 5h in $CDCl_3$

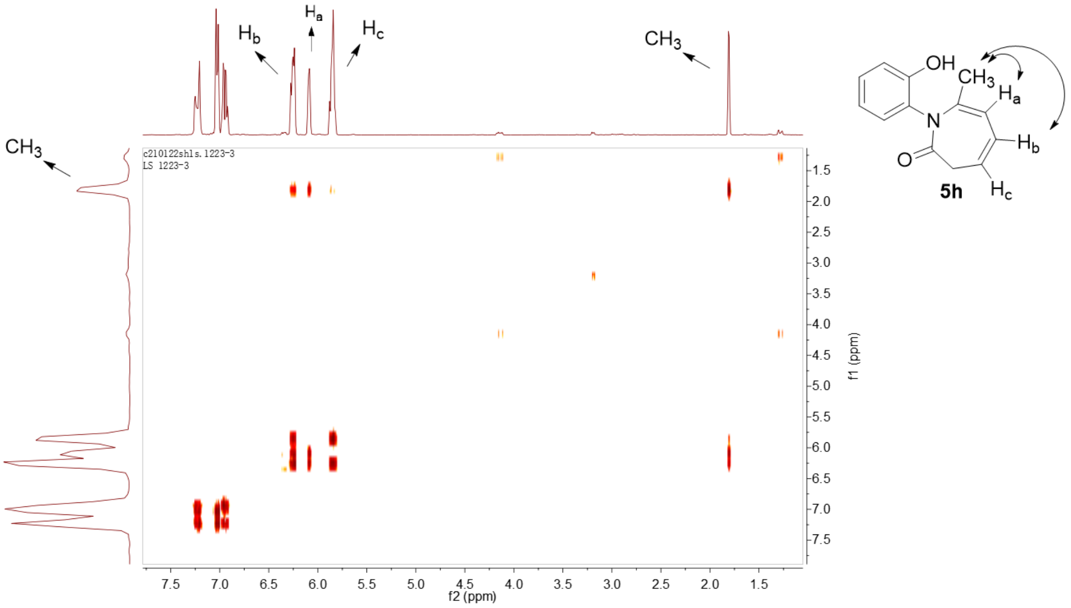

Supplementary Figure 84. 400 MHz 2D-COSY of compound 5h in $CDCl_3$

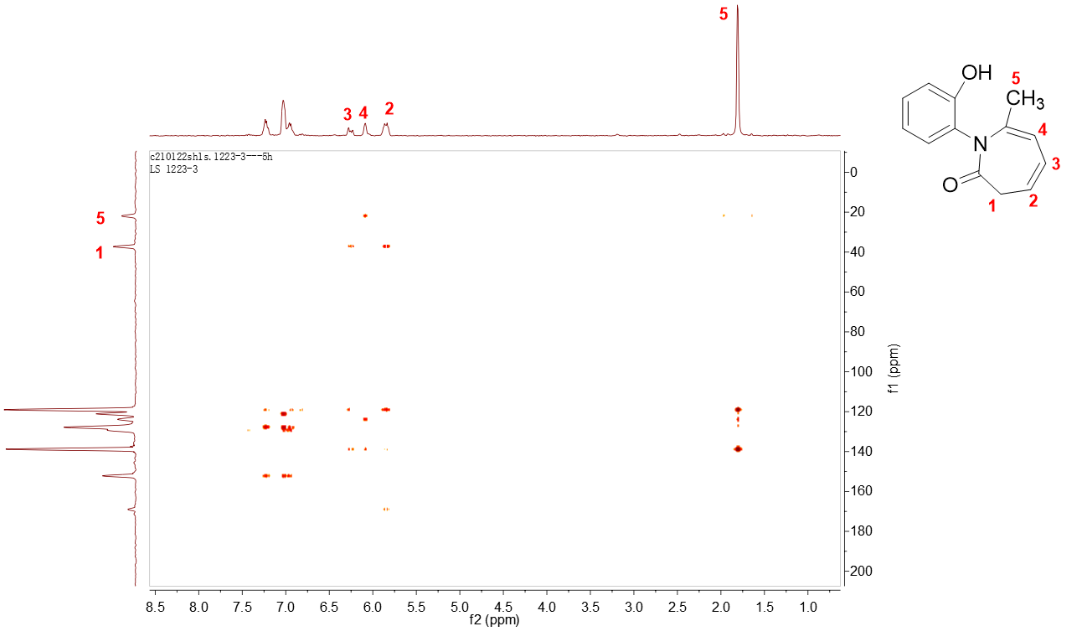

Supplementary Figure 85. 400 MHz 2D-HMBC of compound 5h in $CDCl_3$

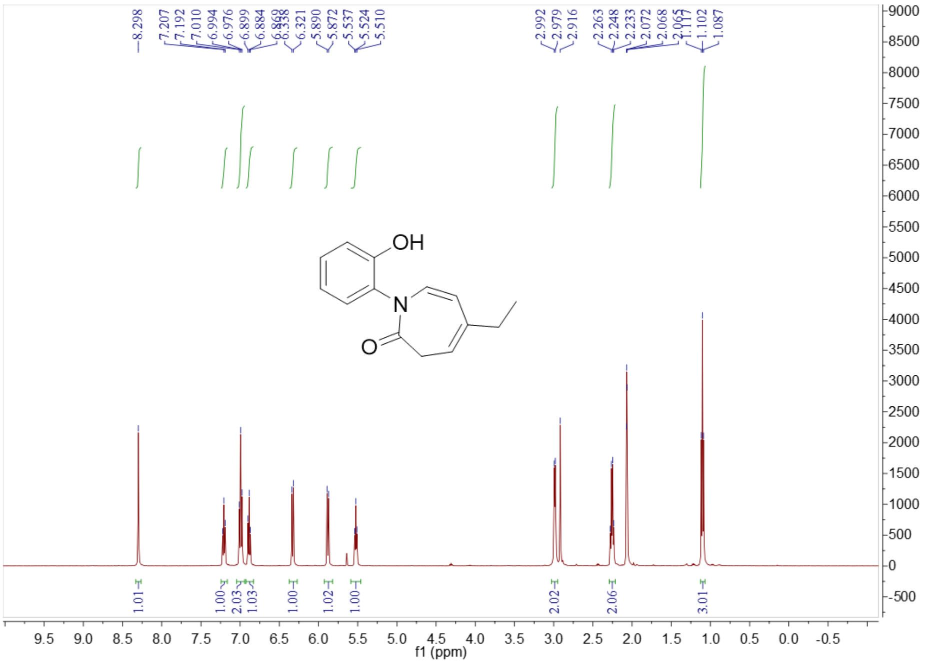

Supplementary Figure 86. 500 MHz ^{1}H NMR of compound 5i in Acetone-d_6

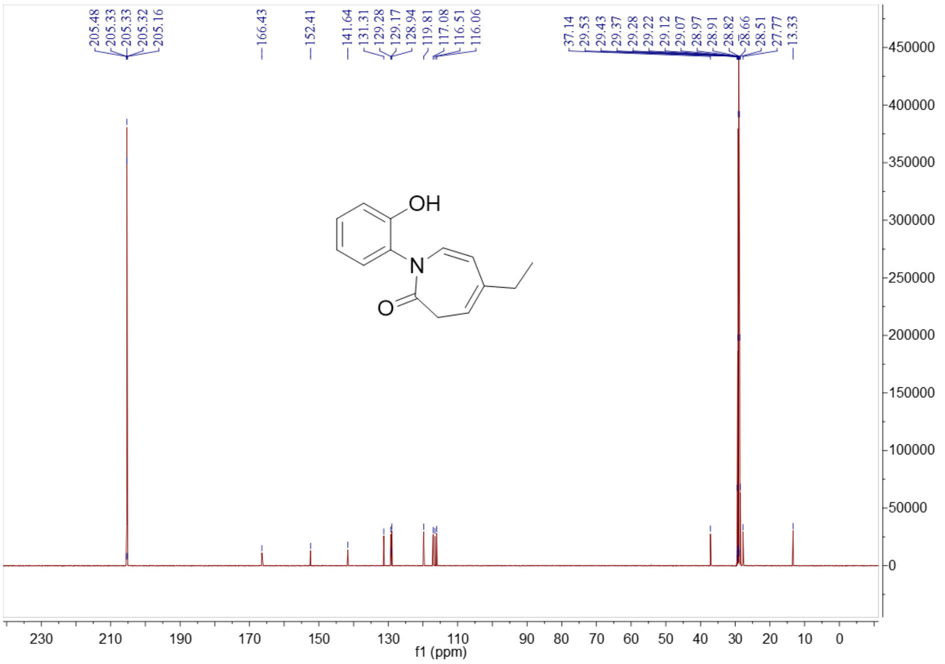

Supplementary Figure 87. 125 MHz ^{13}C NMR of compound 5i in Acetone-d_6

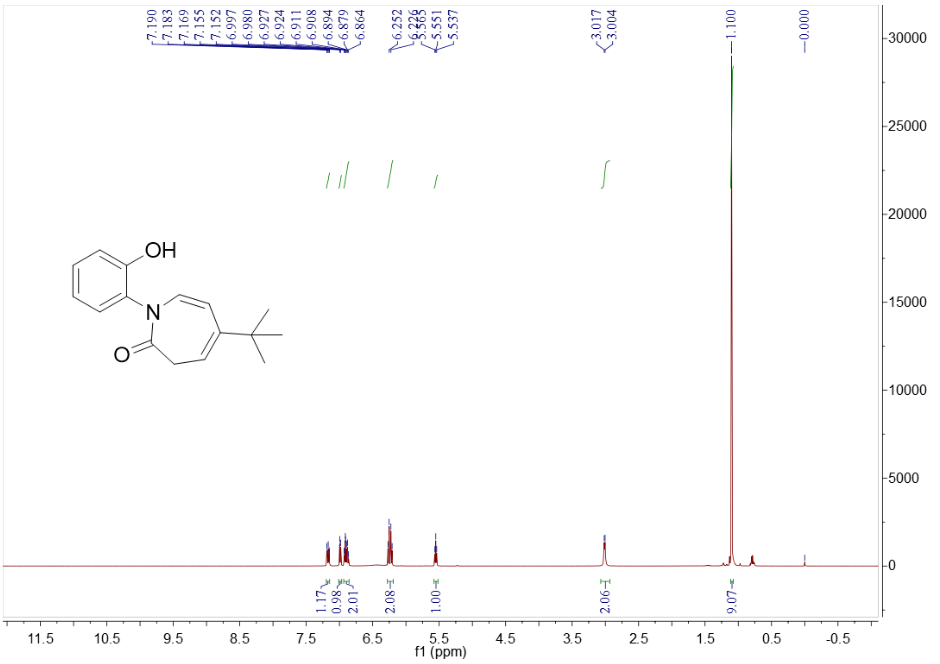

Supplementary Figure 88. 500 MHz ^{1}H NMR of compound 5j in $CDCl_3$

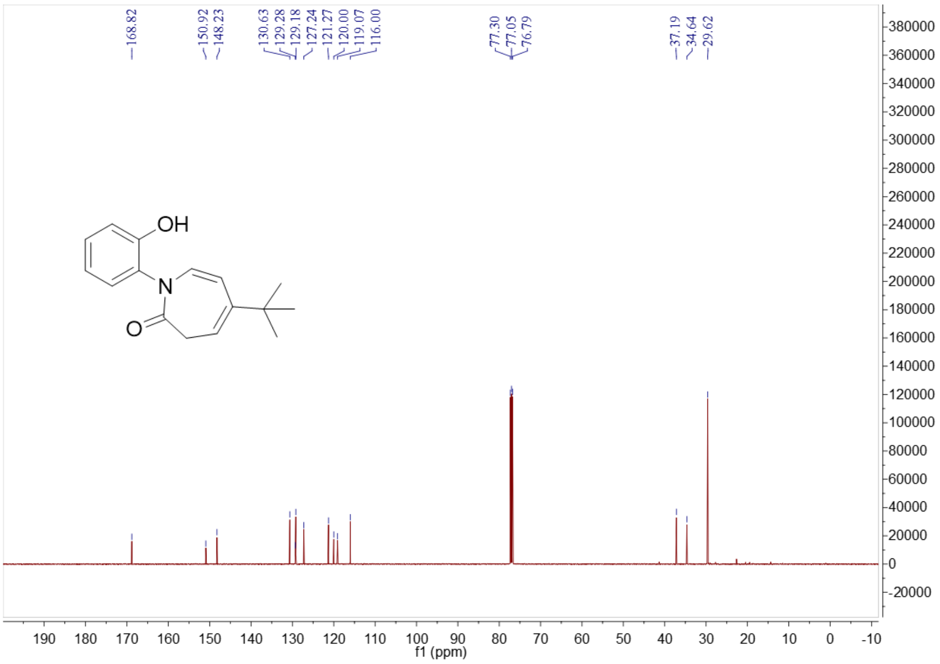

Supplementary Figure 89. 125 MHz ^{13}C NMR of compound 5j in $CDCl_3$

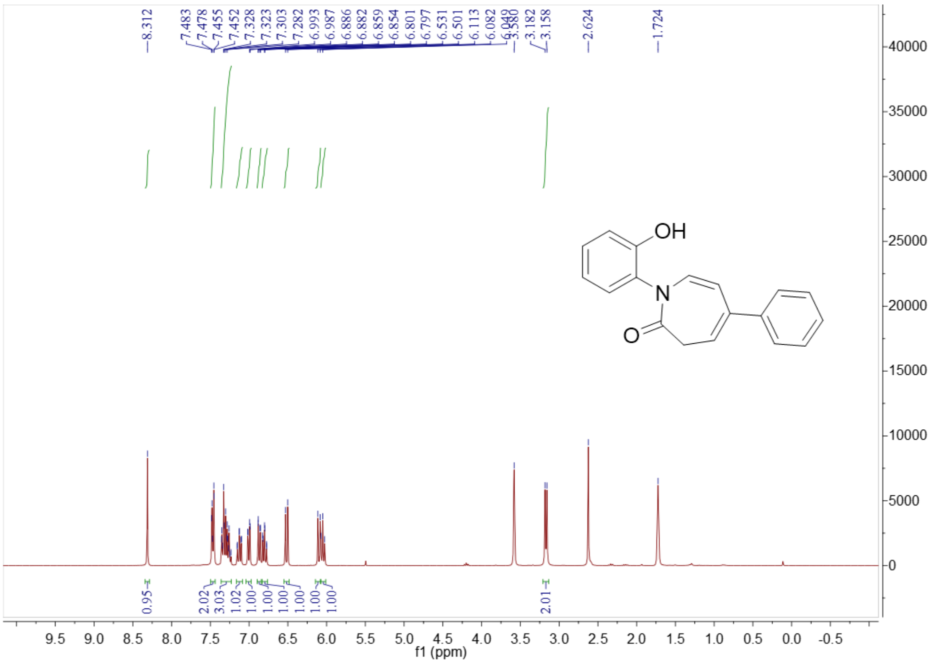

Supplementary Figure 90. 300 MHz ^{1}H NMR of compound 5k in THF-d_8

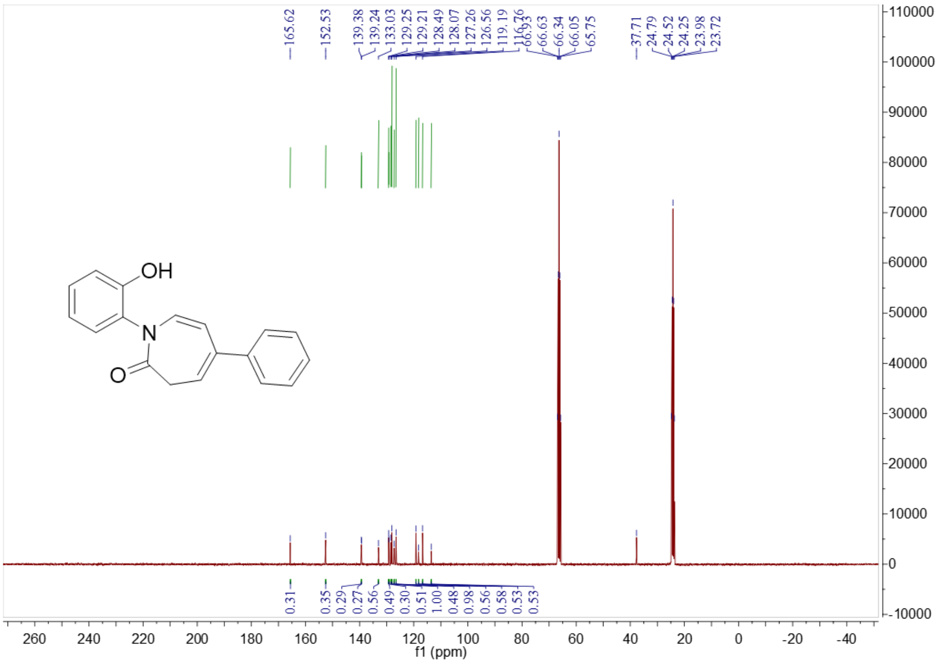

Supplementary Figure 91. 75 MHz ^{13}C NMR of compound 5k in THF-d_8

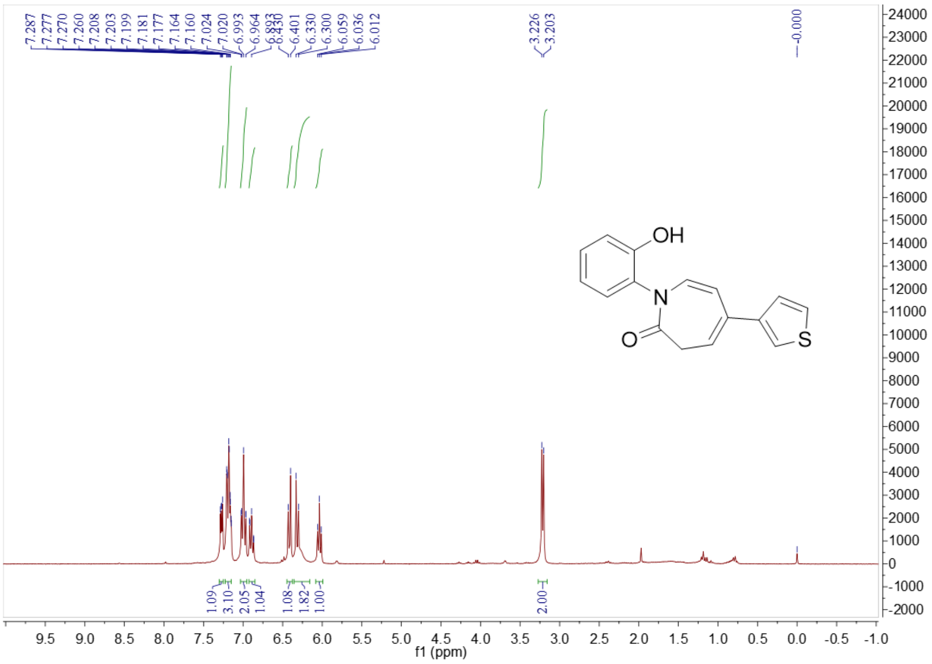

Supplementary Figure 92. 300 MHz ^{1}H NMR of compound 5l in $CDCl_3$

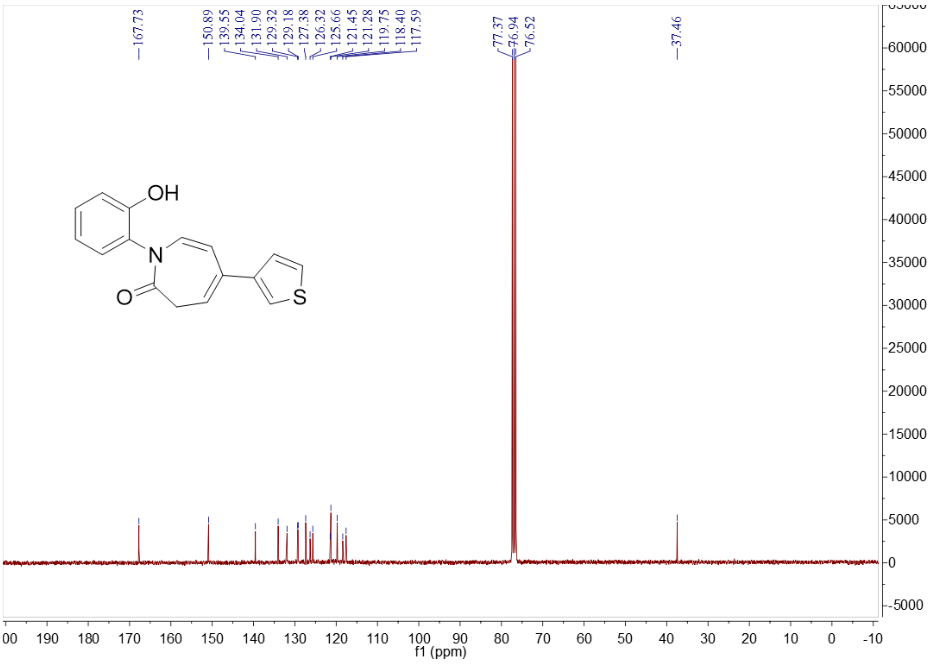

Supplementary Figure 93. 75 MHz ^{13}C NMR of compound 5l in $CDCl_3$

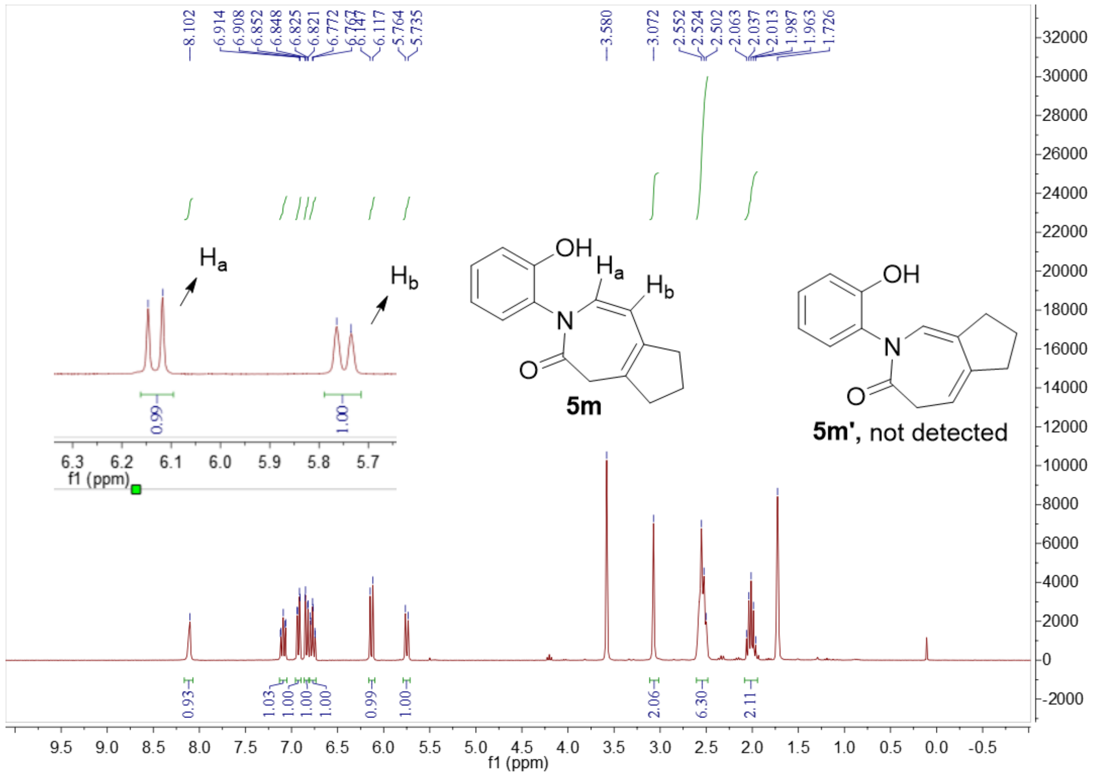

Supplementary Figure 94. 300 MHz ^{1}H NMR of compound 5m in THF-d_8

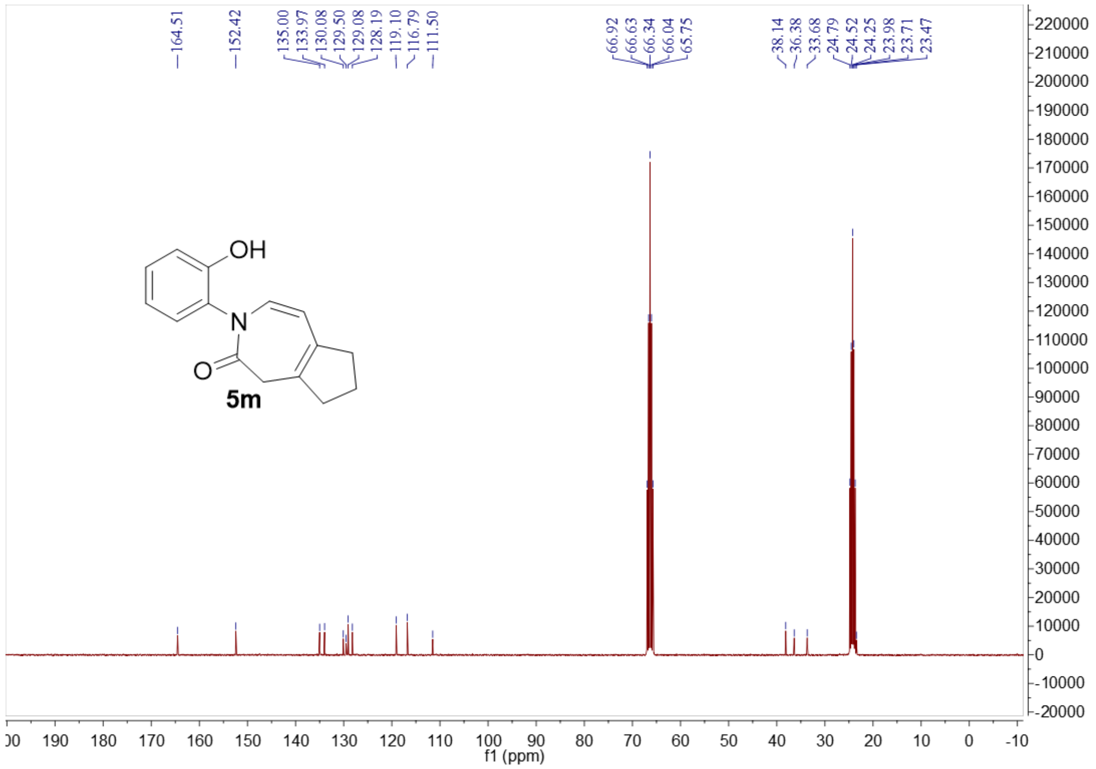

Supplementary Figure 95. 75 MHz ^{13}C NMR of compound 5m in THF-d_8

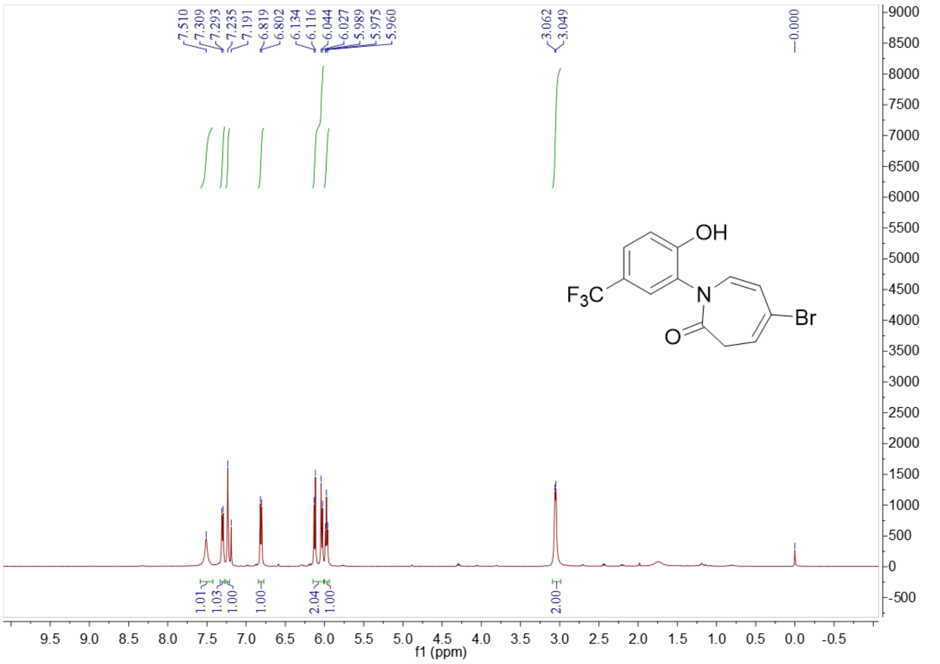

Supplementary Figure 96. 500 MHz ^{1}H NMR of compound 5n in $CDCl_3$

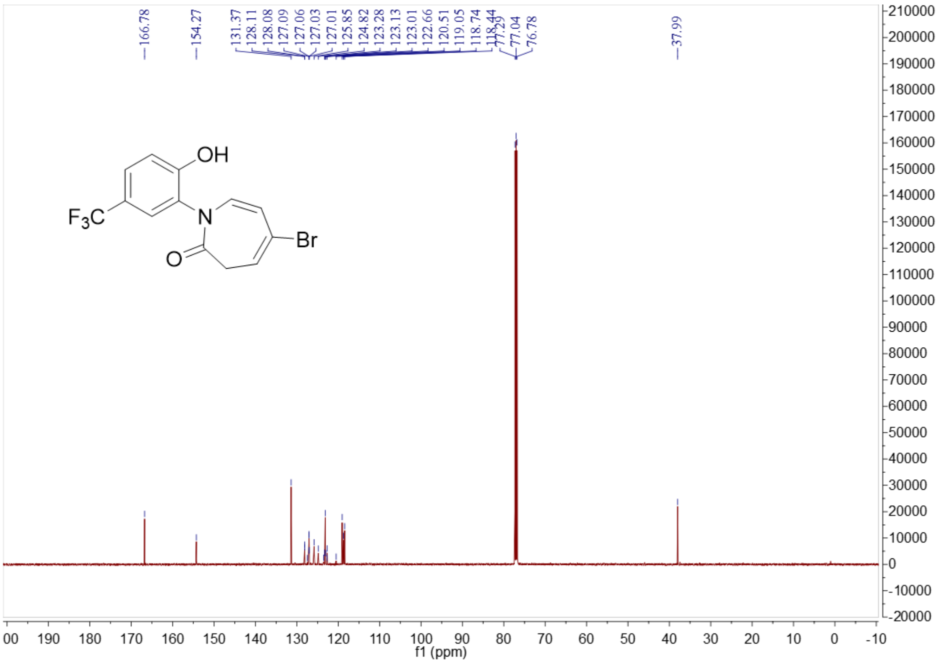

Supplementary Figure 97. 125 MHz ^{13}C NMR of compound 5n in $CDCl_3$

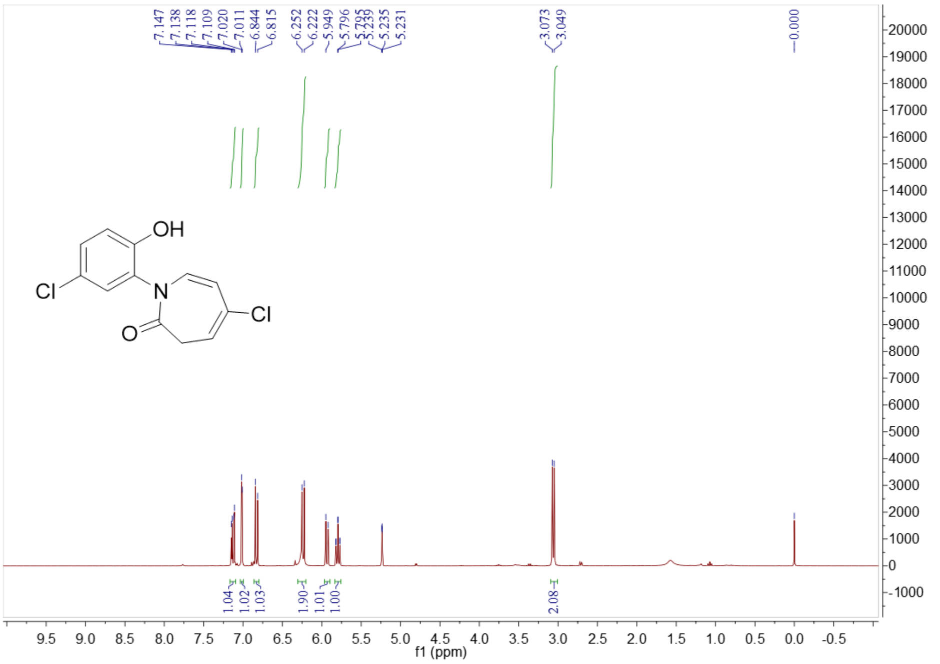

Supplementary Figure 98. 300 MHz ^{1}H NMR of compound 5o in CD_2Cl_2

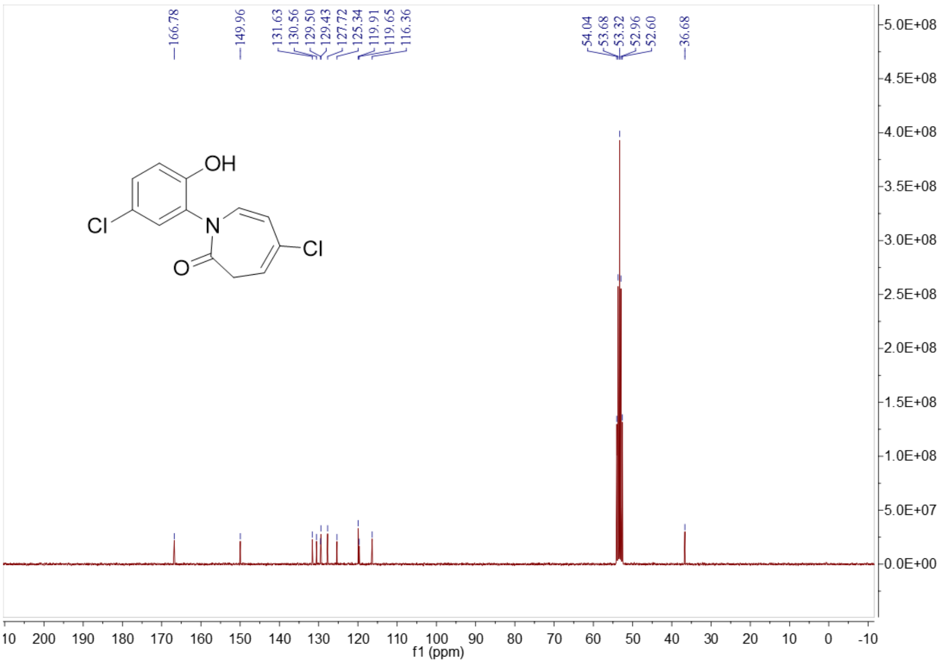

Supplementary Figure 99. 75 MHz ^{13}C NMR of compound 5o in CD_2Cl_2

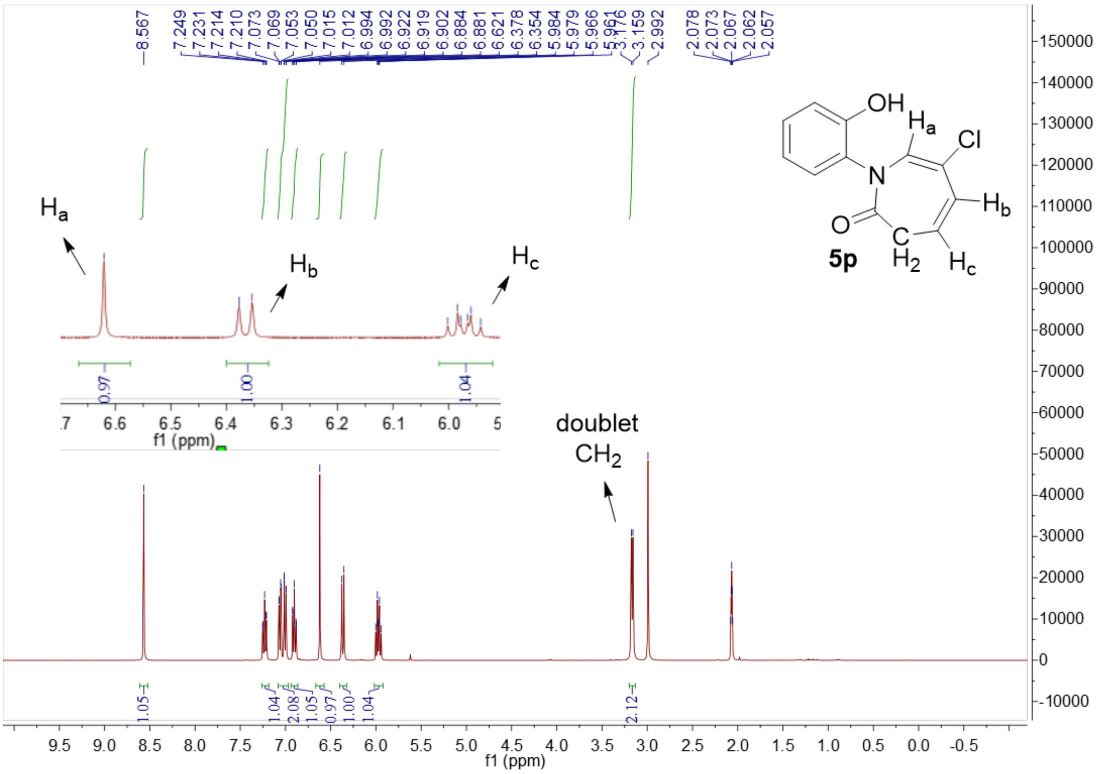

Supplementary Figure 100. 500 MHz ^{1}H NMR of compound 5p in Acetone-d_6

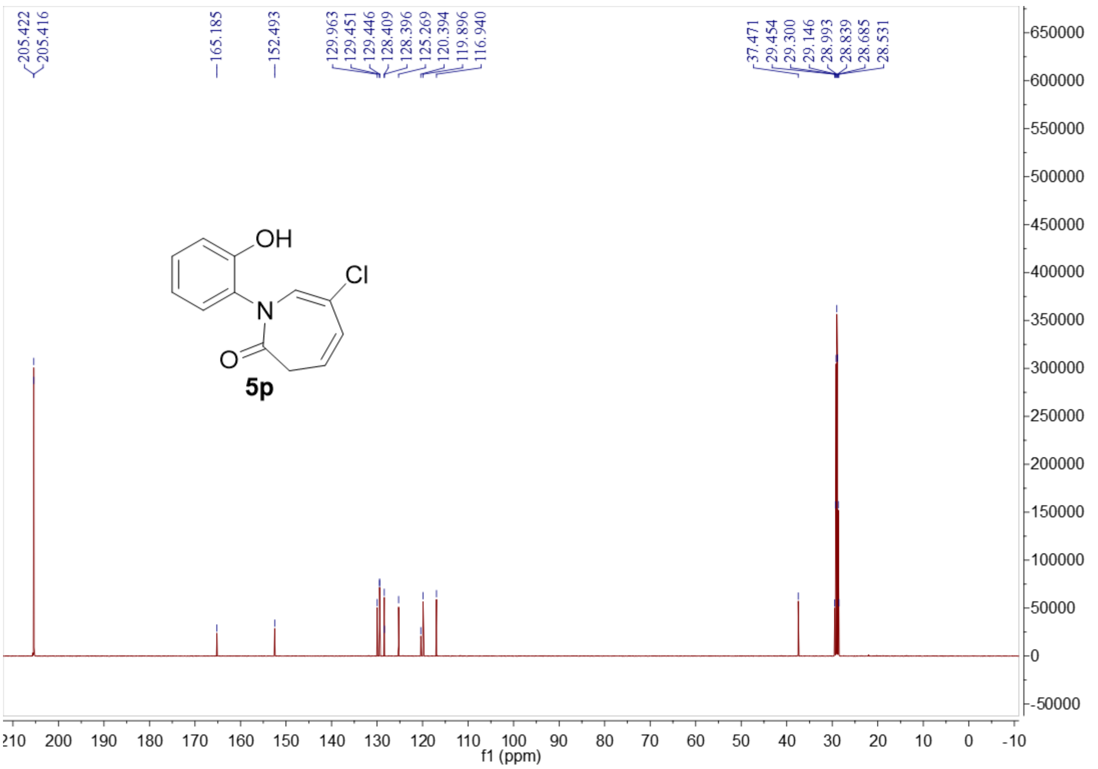

Supplementary Figure 101. 125 MHz ^{13}C NMR of compound 5p in Acetone-d_6

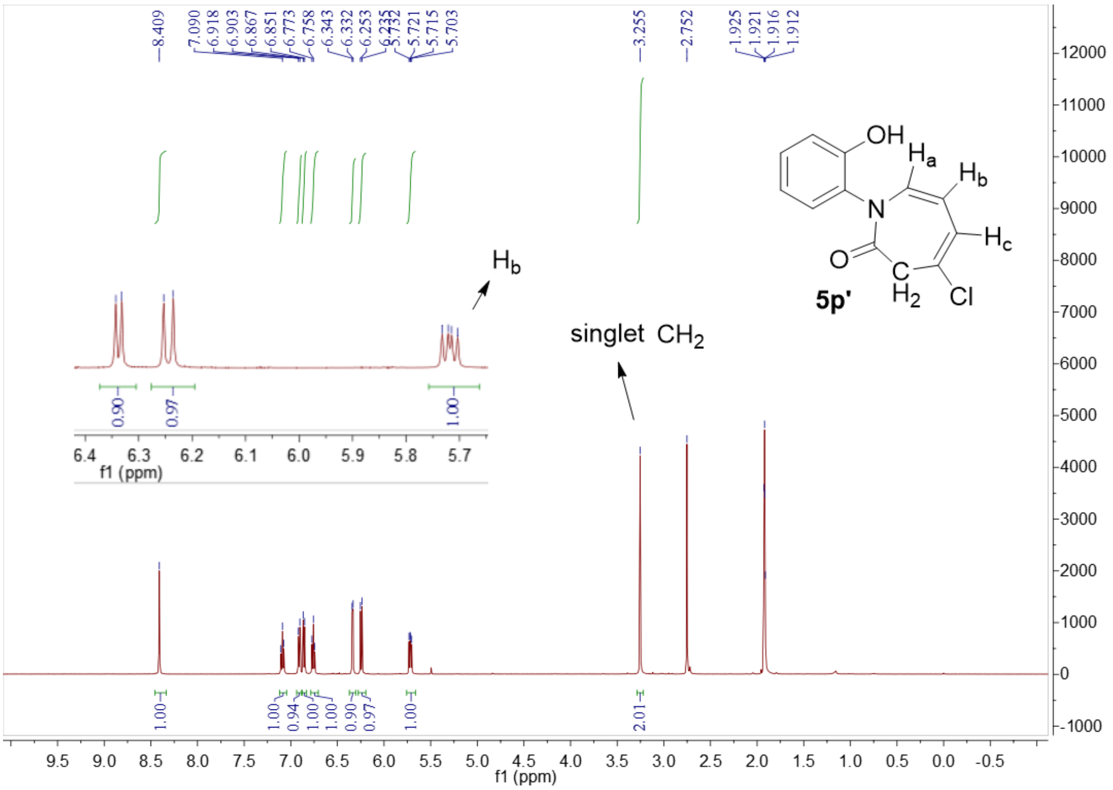

Supplementary Figure 102. 500 MHz ^{1}H NMR of compound 5p' in Acetone-d_6

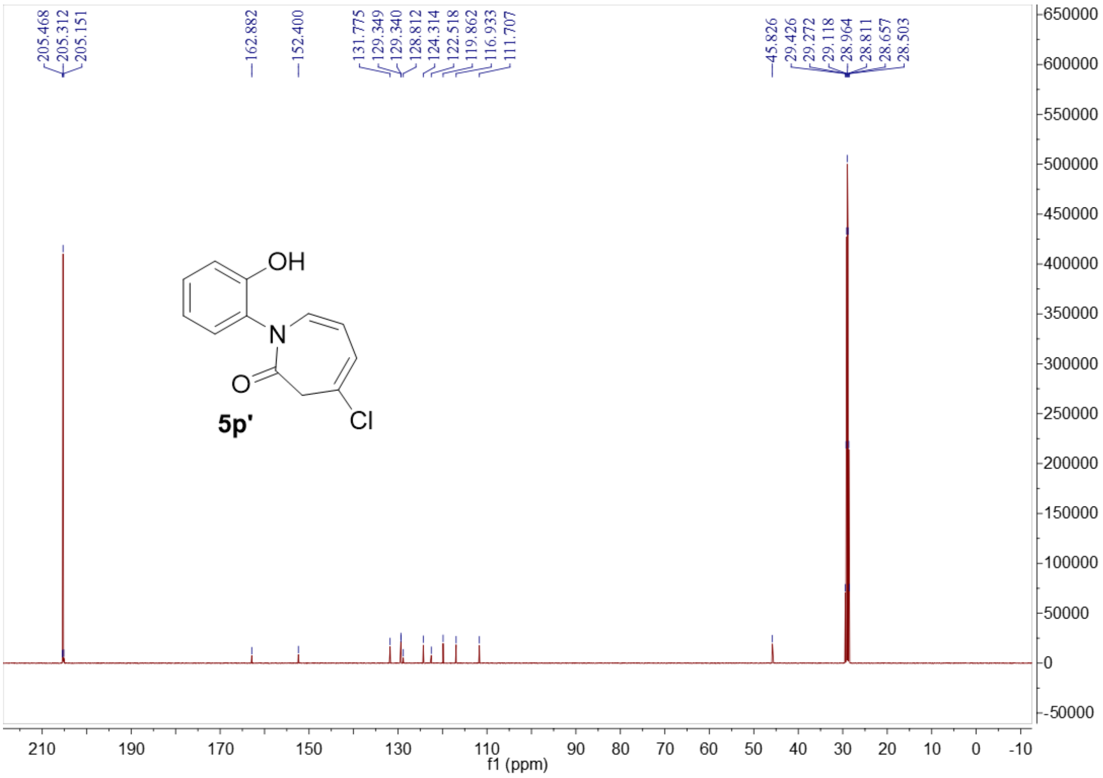

Supplementary Figure 103. 125 MHz ^{13}C NMR of compound 5p' in Acetone-d_6

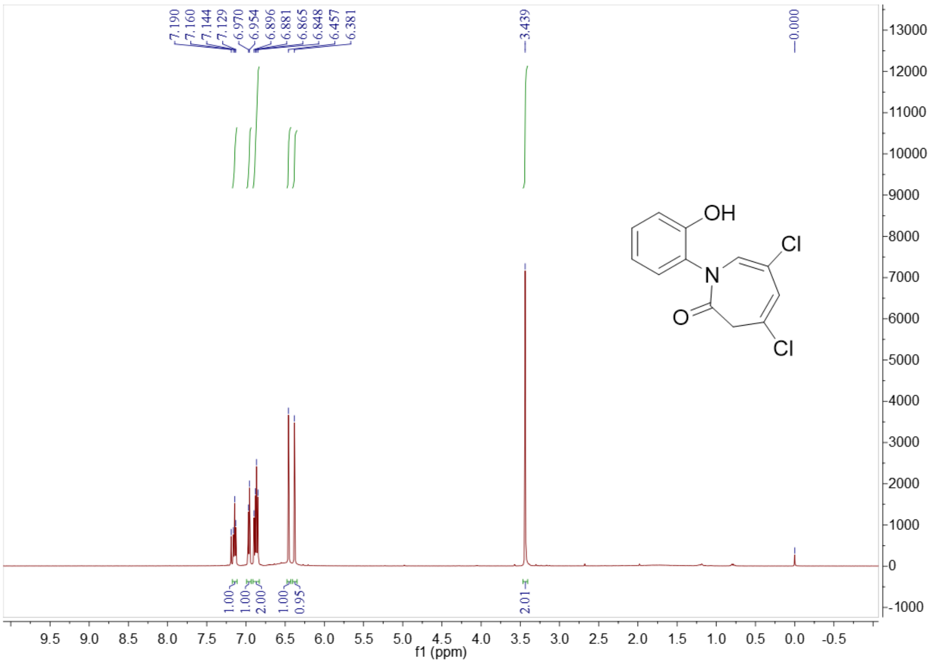

Supplementary Figure 104. 500 MHz ^{1}H NMR of compound 5q in $CDCl_3$

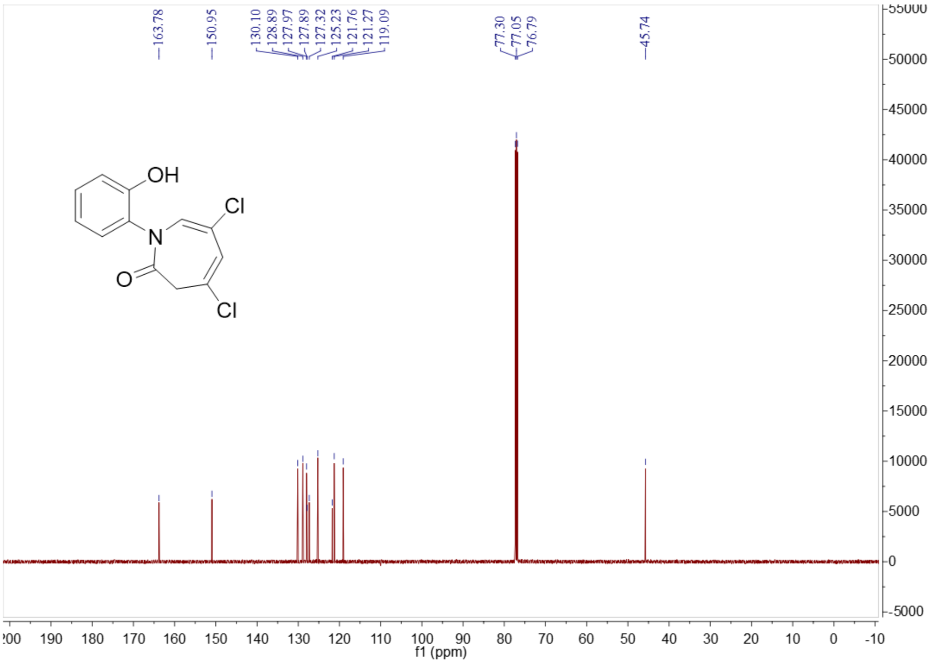

Supplementary Figure 105. 125 MHz ^{13}C NMR of compound 5q in $CDCl_3$

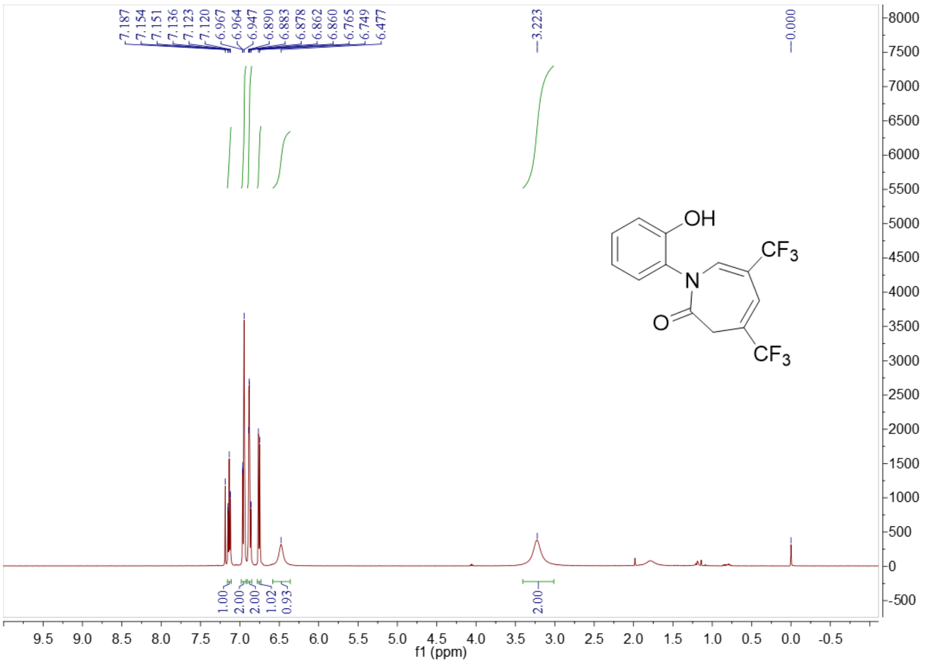

Supplementary Figure 106. 500 MHz ^{1}H NMR of compound 5r in $CDCl_3$

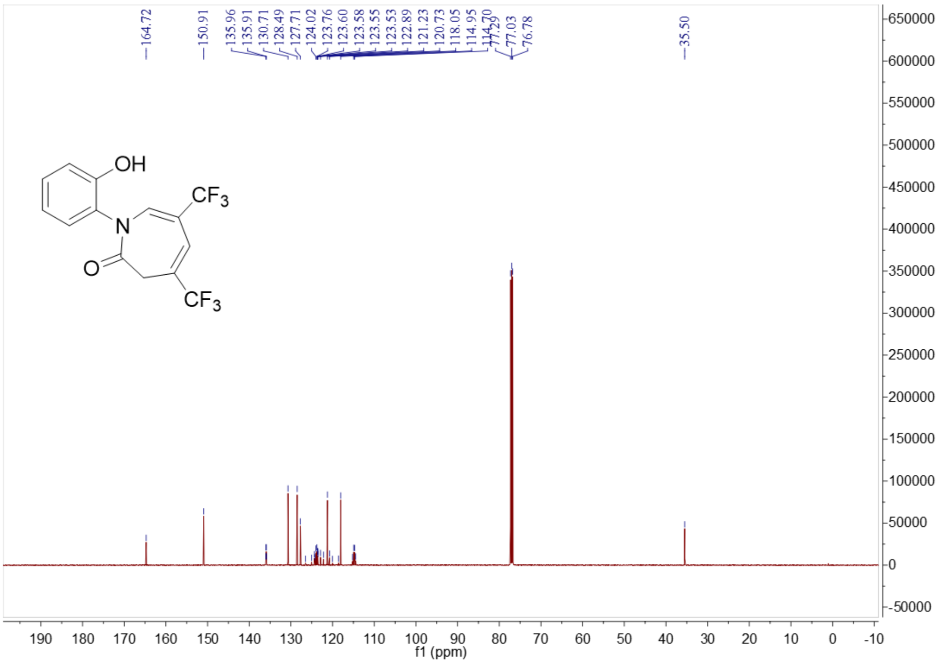

Supplementary Figure 107. 125 MHz ^{13}C NMR of compound 5r in $CDCl_3$

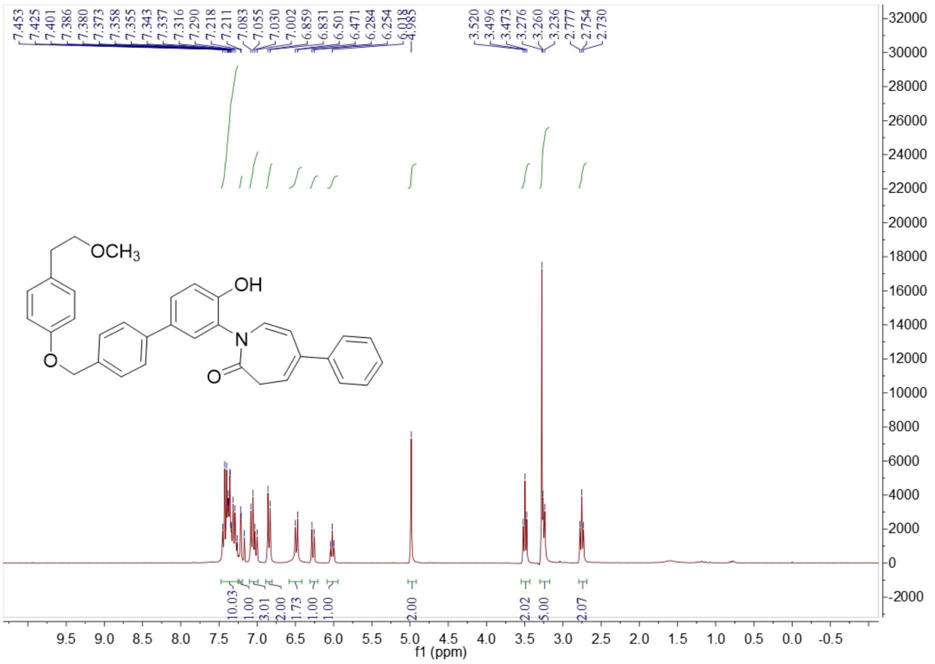

Supplementary Figure 108. 300 MHz ^{1}H NMR of compound 5s in $CDCl_3$

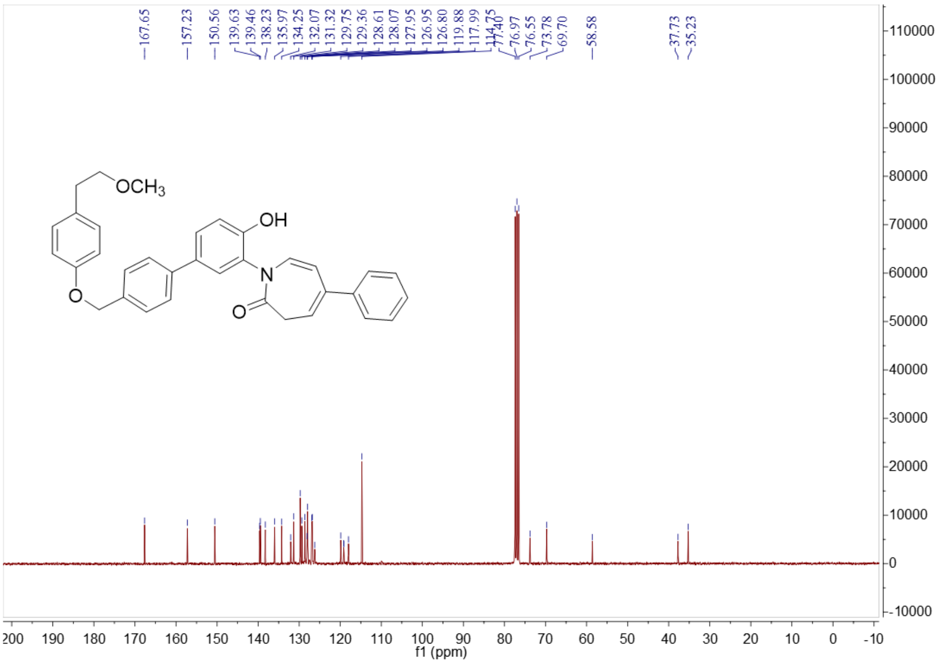

Supplementary Figure 109. 75 MHz ^{13}C NMR of compound 5s in $CDCl_3$

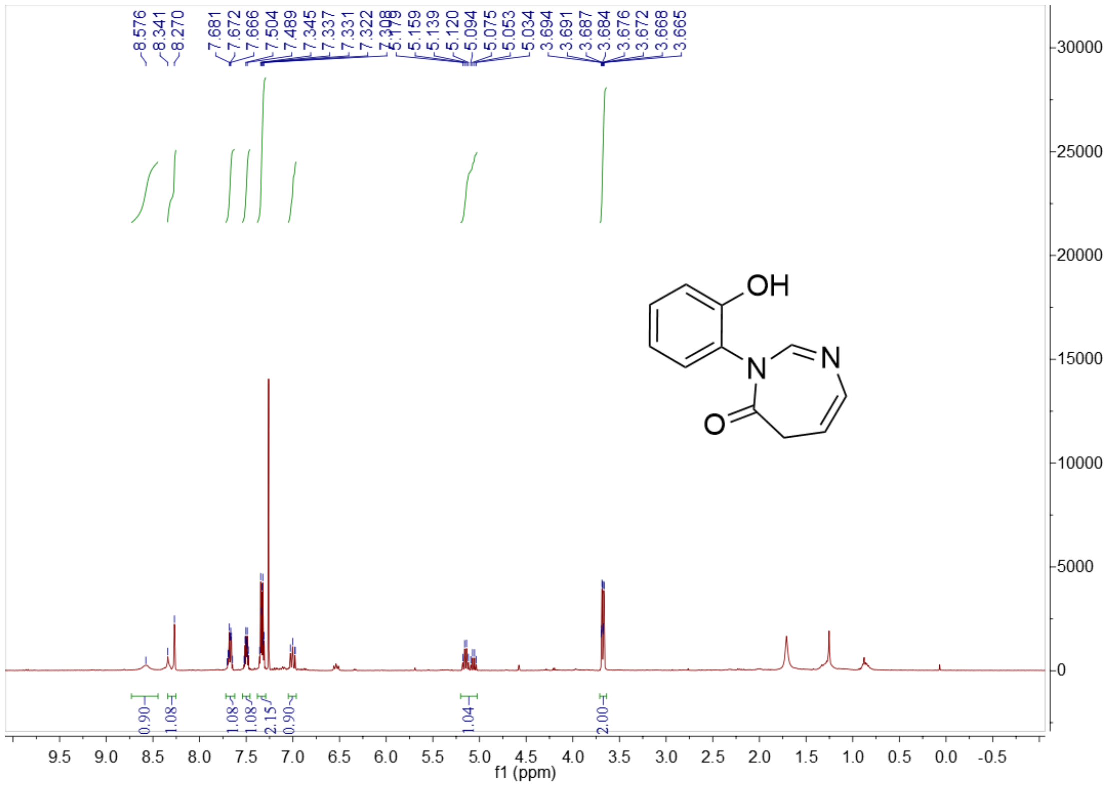

Supplementary Figure 110. 300 MHz ^{1}H NMR of compound 5v in $CDCl_3$

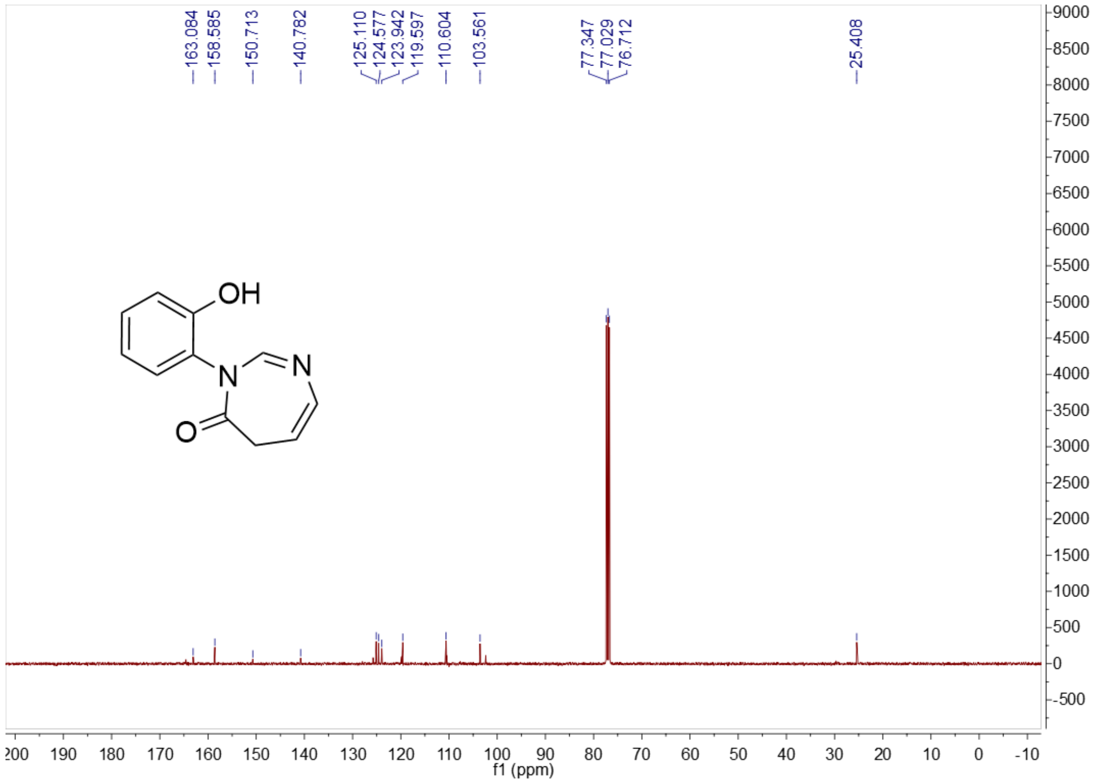

Supplementary Figure 111. 75 MHz ^{13}C NMR of compound 5v in $CDCl_3$

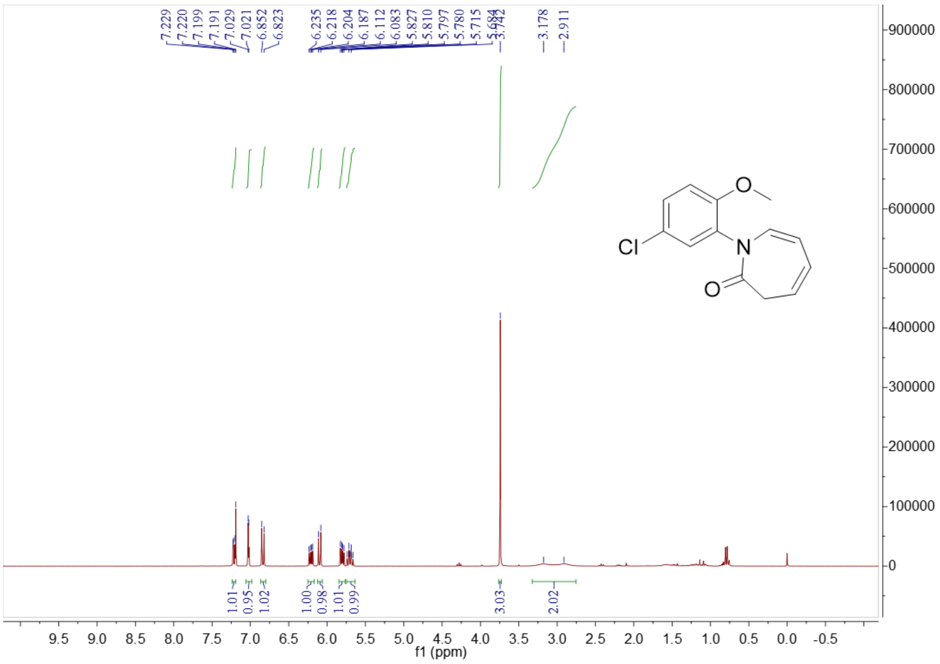

Supplementary Figure 112. 300 MHz ^{1}H NMR of compound 6 in $CDCl_3$

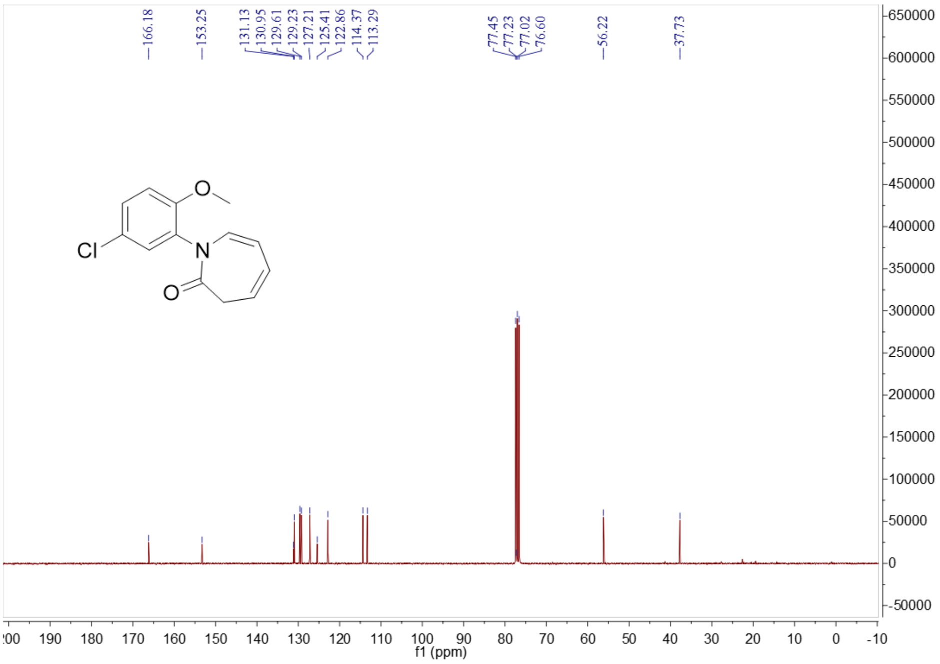

Supplementary Figure 113. 75 MHz ^{13}C NMR of compound 6 in $CDCl_3$

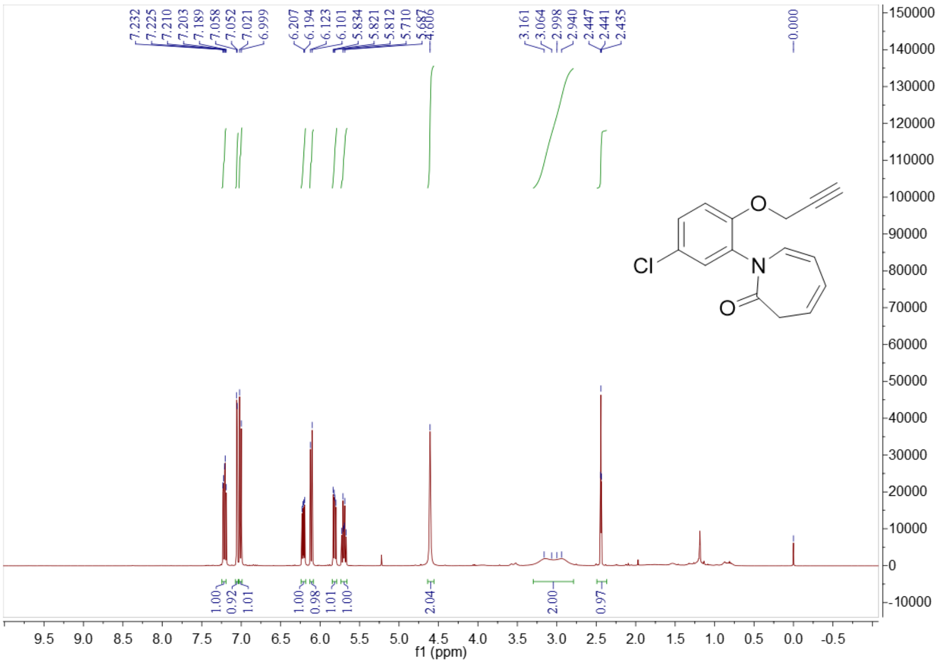

Supplementary Figure 114. 400 MHz ^{1}H NMR of compound 7 in $CDCl_3$

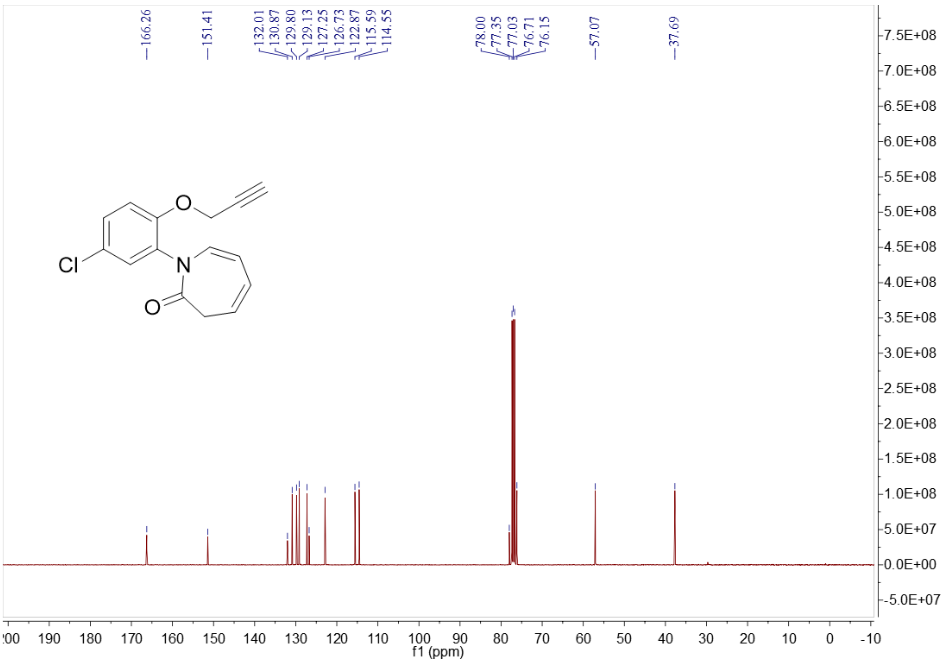

Supplementary Figure 115. 100 MHz ^{13}C NMR of compound 7 in $CDCl_3$

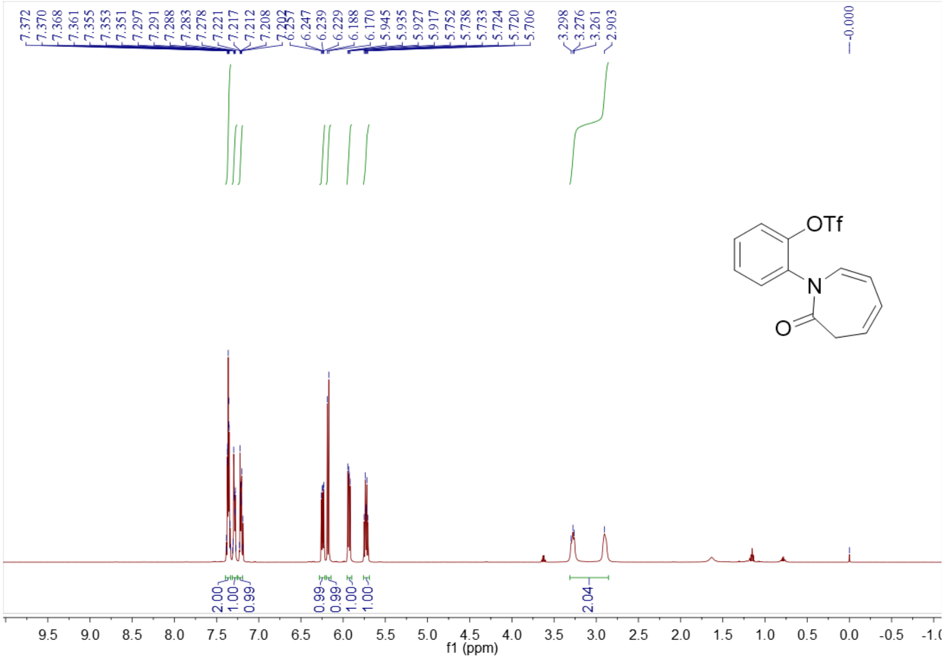

Supplementary Figure 116. 500 MHz ^{1}H NMR of compound 8 in $CDCl_3$

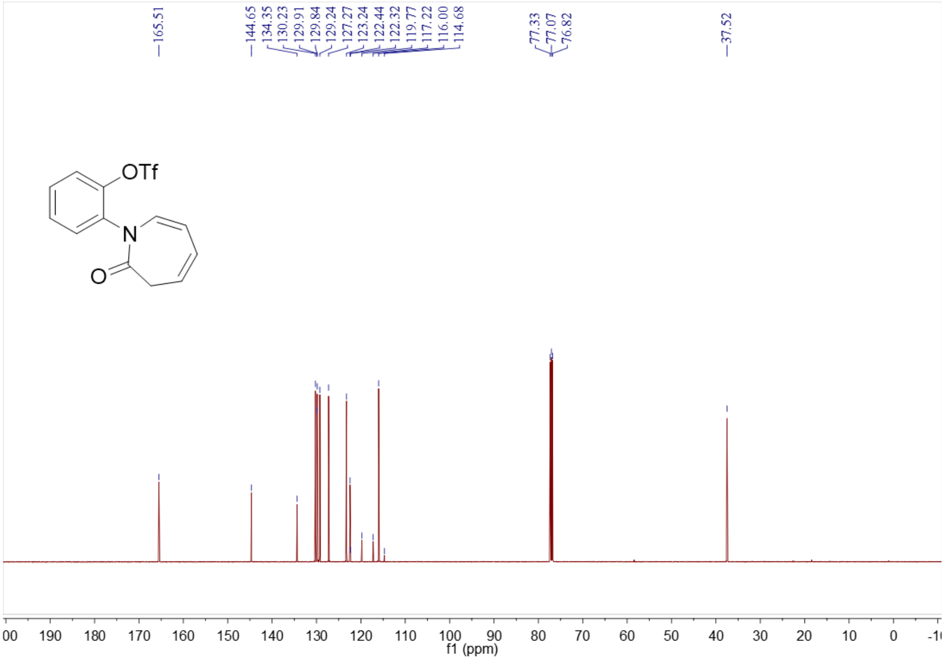

Supplementary Figure 117. 125 MHz ^{13}C NMR of compound 8 in $CDCl_3$

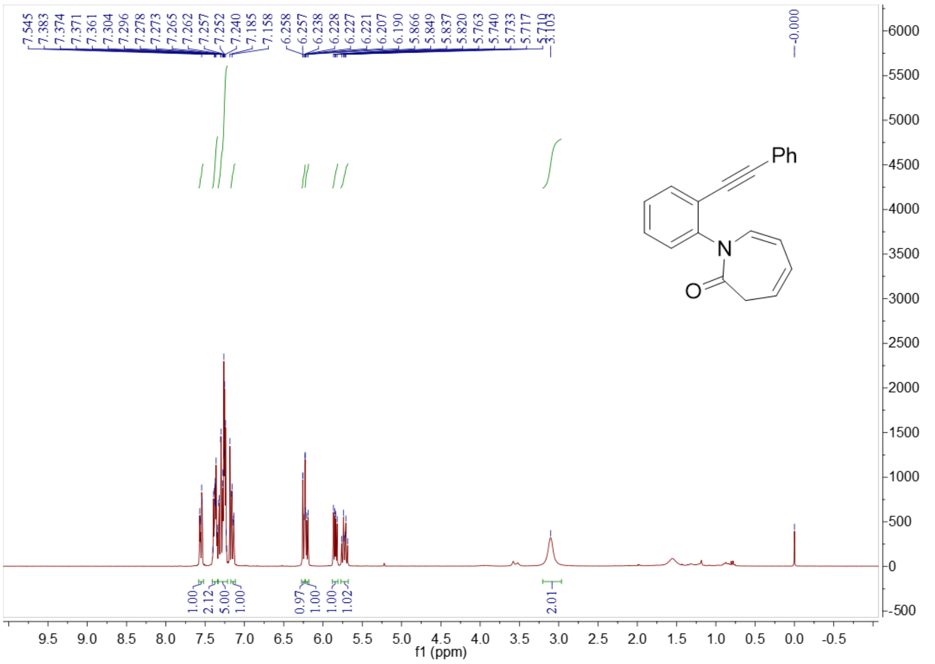

Supplementary Figure 118. 300 MHz ^{1}H NMR of compound 9 in $CDCl_3$

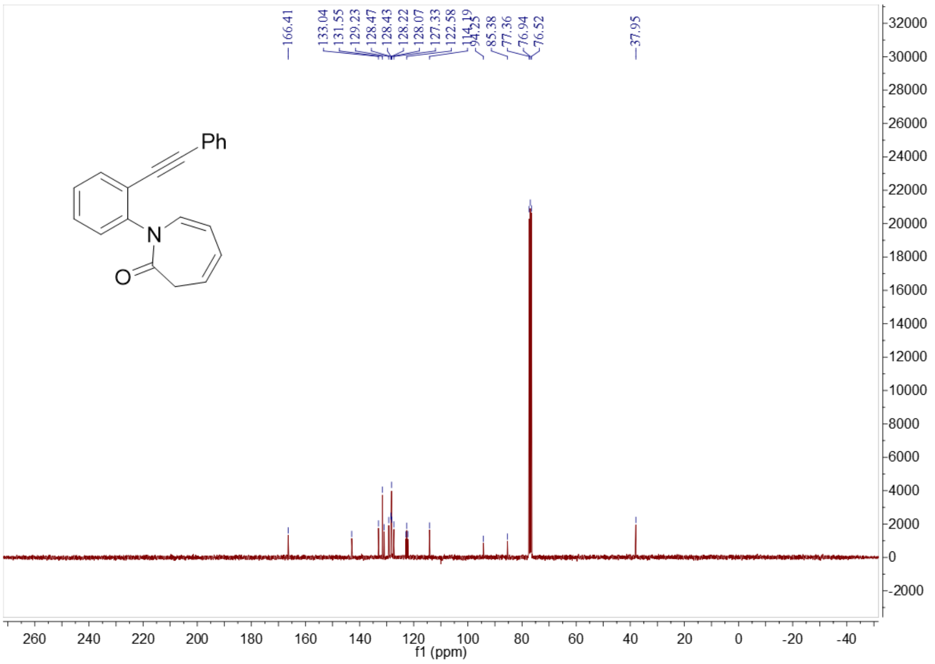

Supplementary Figure 119. 75 MHz ^{13}C NMR of compound 9 in $CDCl_3$

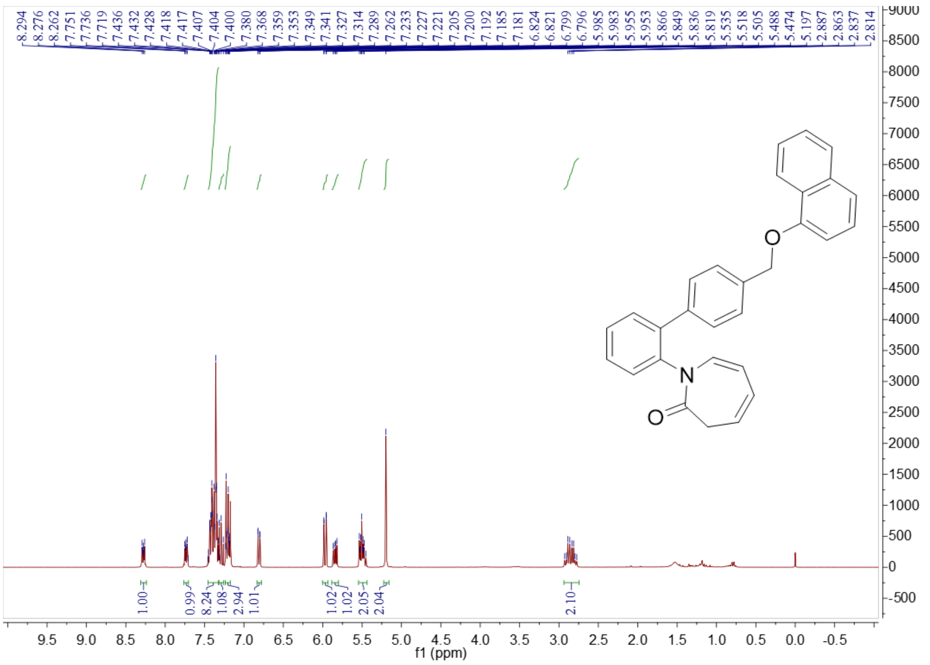

Supplementary Figure 120. 300 MHz ^{1}H NMR of compound 10 in $CDCl_3$

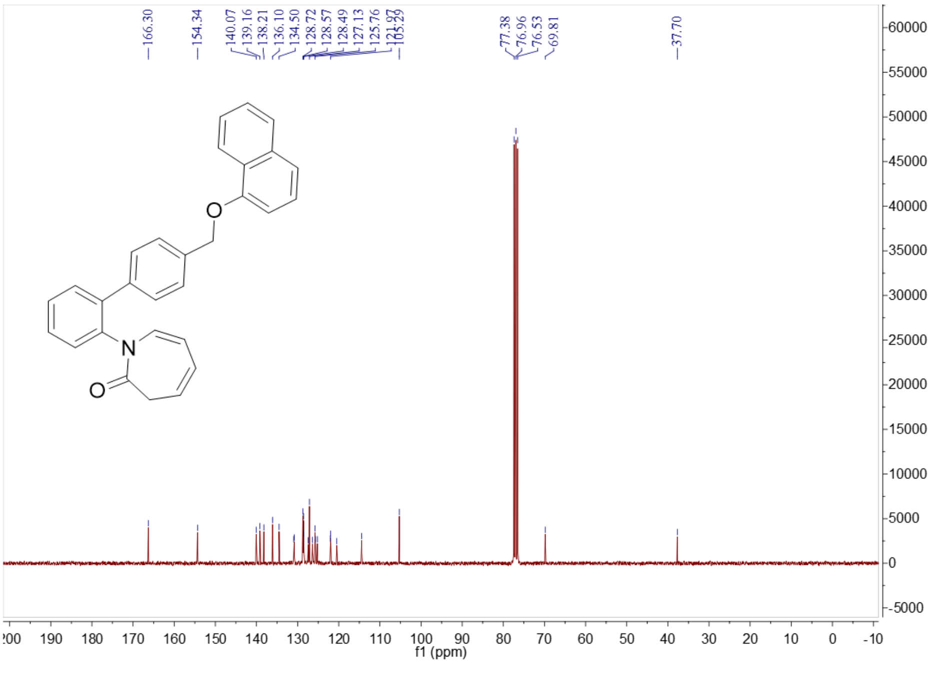

Supplementary Figure 121. 75 MHz ^{13}C NMR of compound 10 in $CDCl_3$

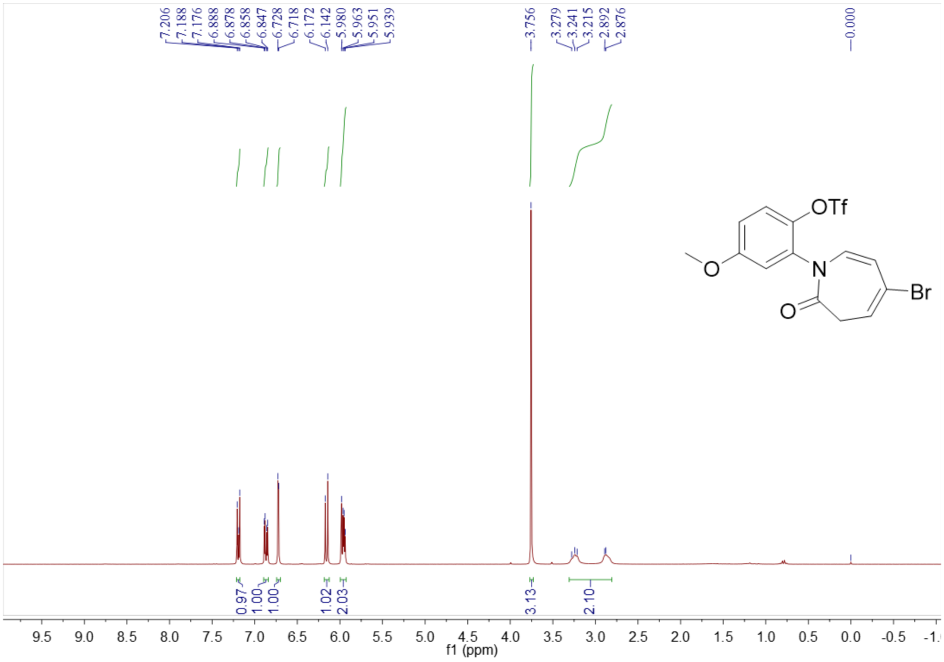

Supplementary Figure 122. 300 MHz ¹H NMR of compound 11 in $CDCl_3$

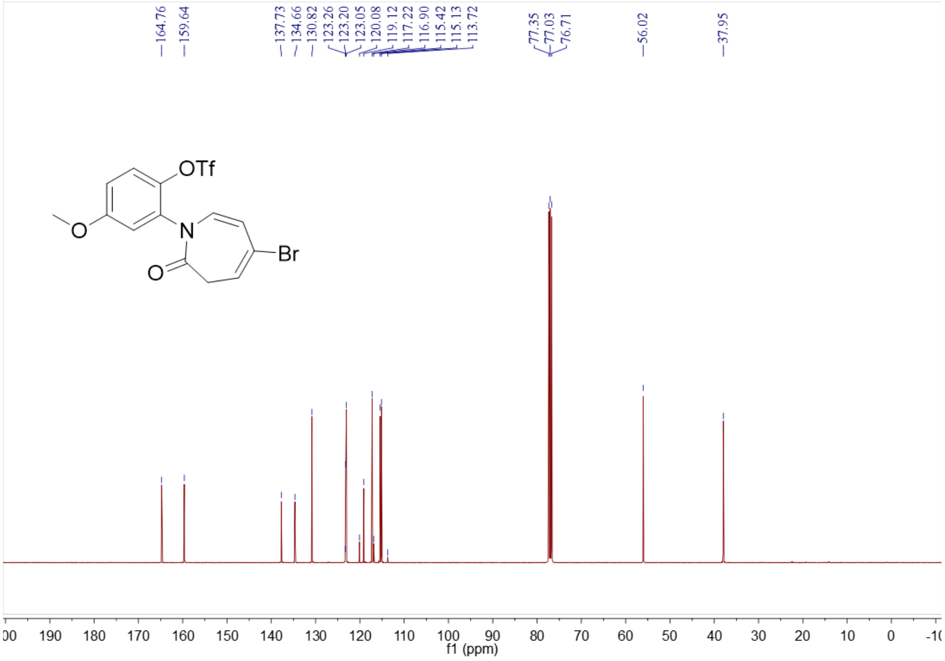

Supplementary Figure 123. 100 MHz ¹³C NMR of compound 11 in $CDCl_3$

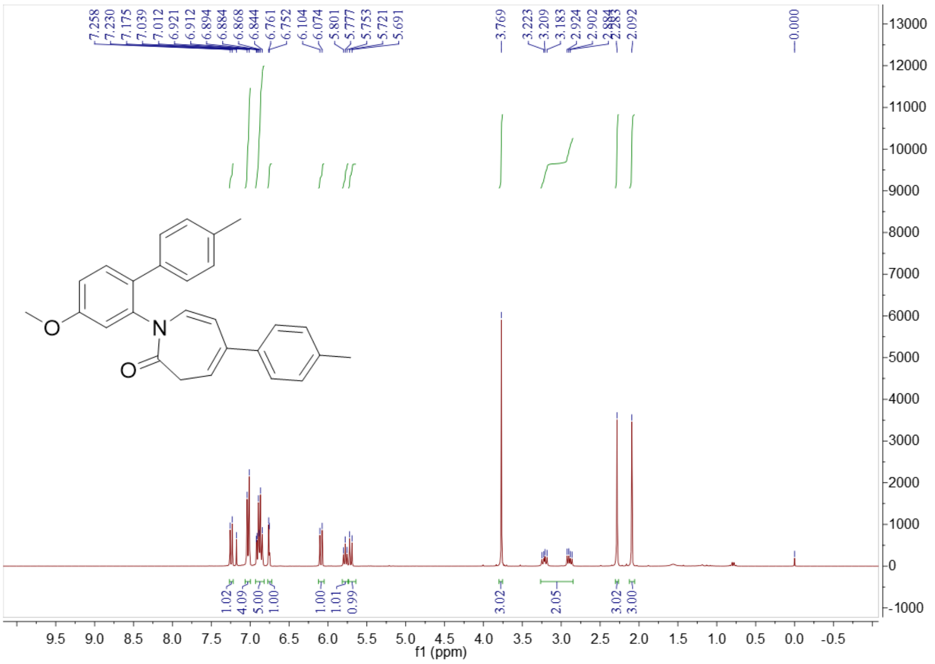

Supplementary Figure 124. 300 MHz ^{1}H NMR of compound 12 in $CDCl_3$

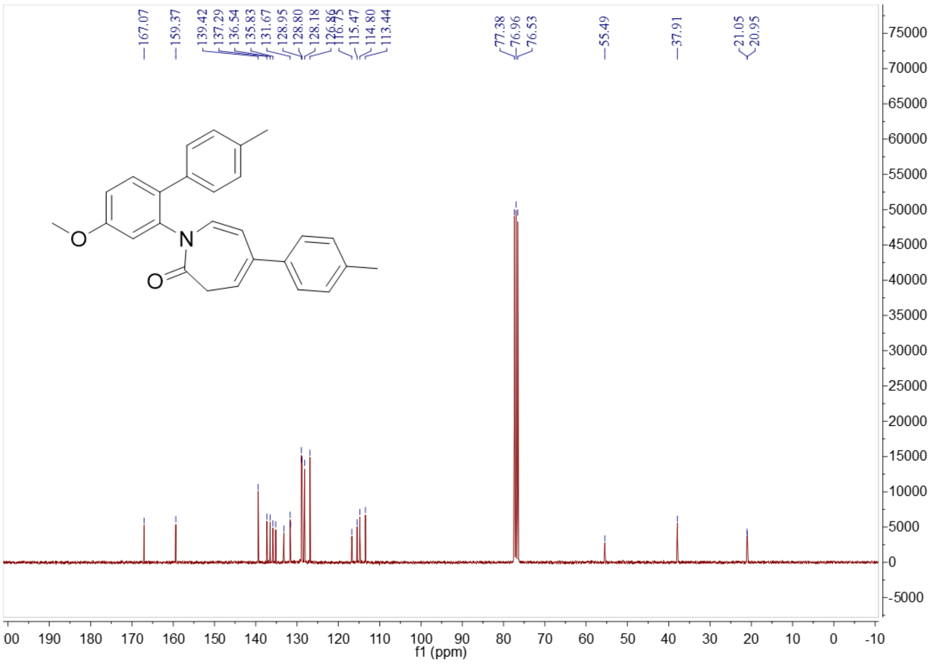

Supplementary Figure 125. 75 MHz ^{13}C NMR of compound 12 in $CDCl_3$

UV-Vis spectra of substrates 3

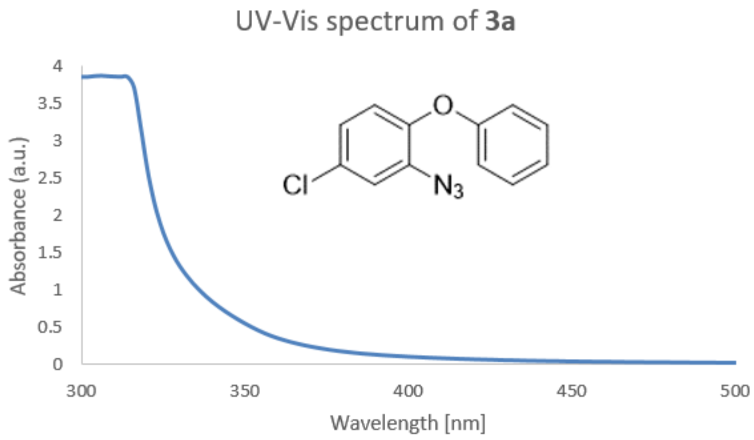

Supplementary Figure 126. UV-Vis spectrum of 3a in THF (0.01 M) at room temperature

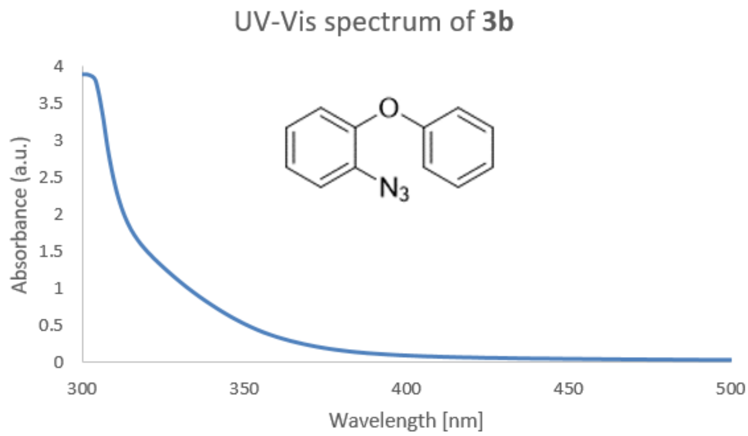

Supplementary Figure 127. UV-Vis spectrum of 3b in THF (0.01 M) at room temperature

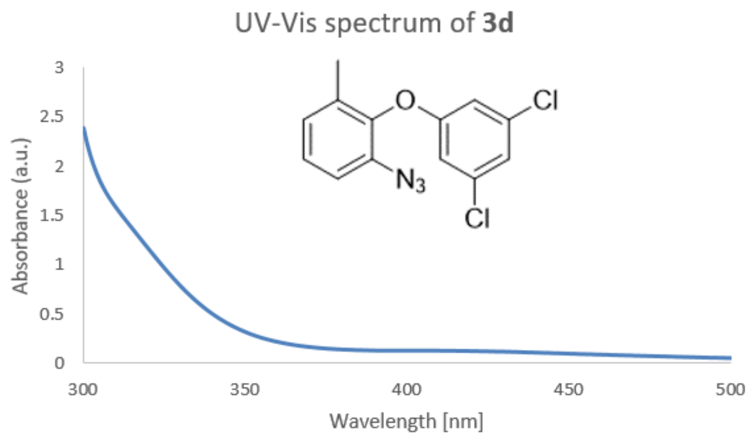

Supplementary Figure 128. UV-Vis spectrum of 3d in THF (0.01 M) at room temperature

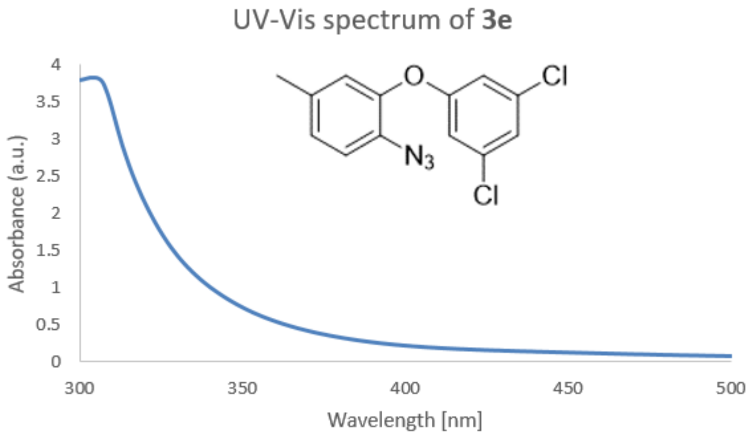

Supplementary Figure 129. UV-Vis spectrum of 3e in THF (0.01 M) at room temperature

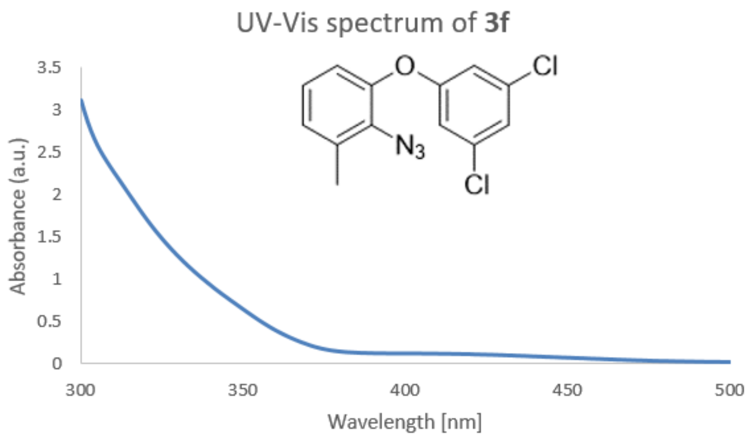

Supplementary Figure 130. UV-Vis spectrum of 3f in THF (0.01 M) at room temperature

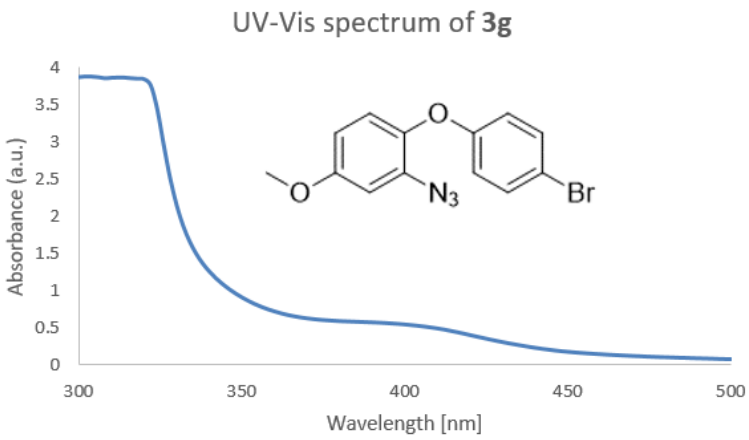

Supplementary Figure 131. UV-Vis spectrum of 3g in THF (0.01 M) at room temperature

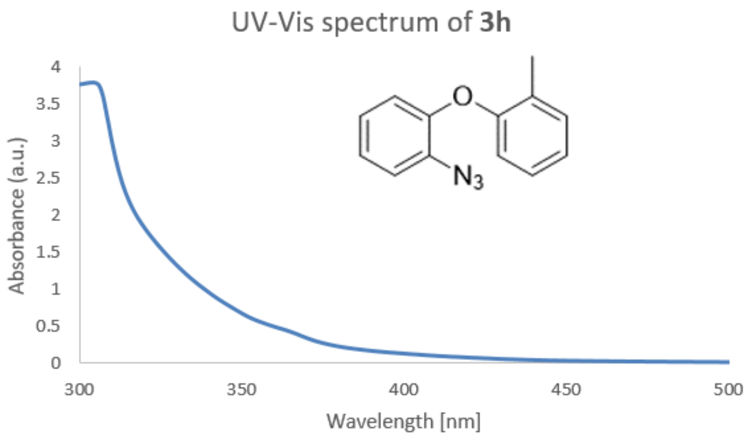

Supplementary Figure 132. UV-Vis spectrum of 3h in THF (0.01 M) at room temperature

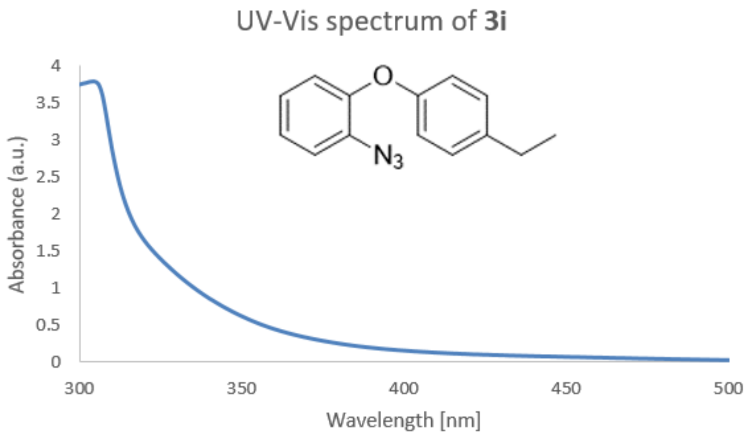

Supplementary Figure 133. UV-Vis spectrum of 3i in THF (0.01 M) at room temperature

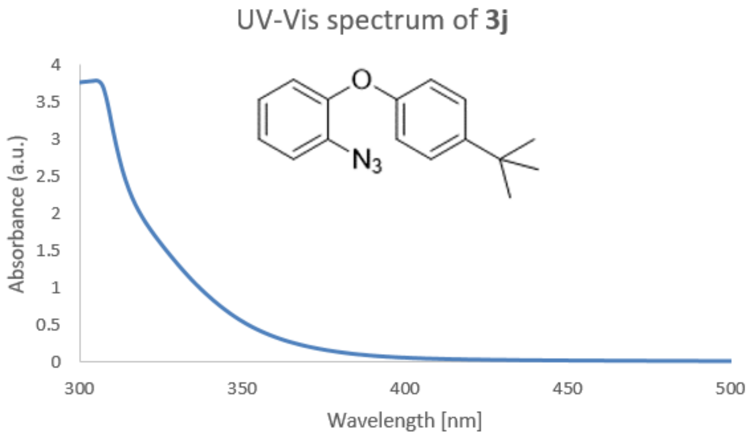

Supplementary Figure 134. UV-Vis spectrum of 3j in THF (0.01 M) at room temperature

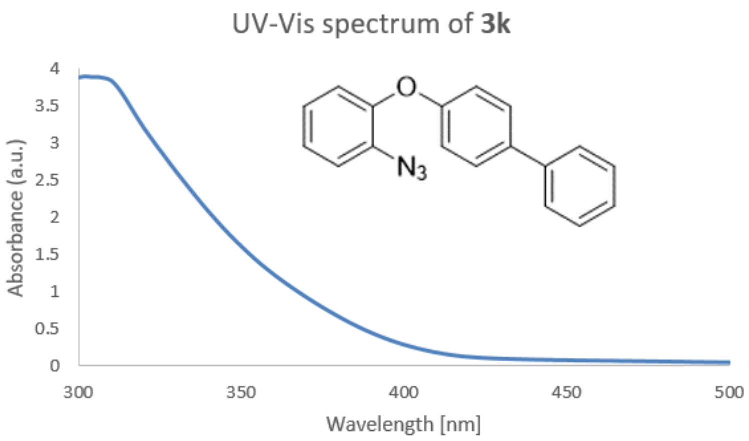

Supplementary Figure 135. UV-Vis spectrum of 3k in THF (0.01 M) at room temperature

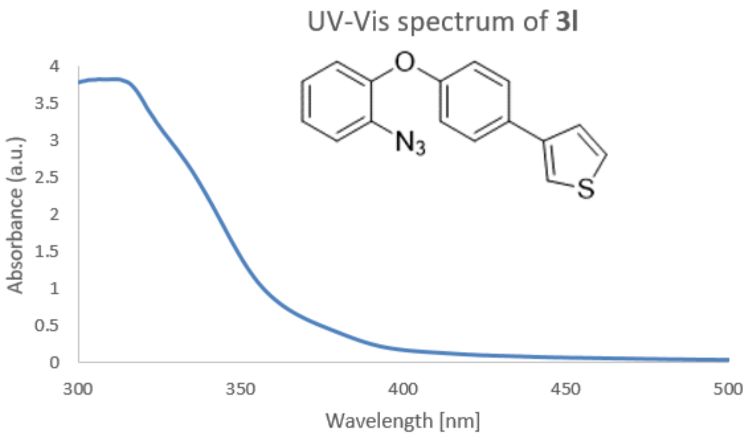

Supplementary Figure 136. UV-Vis spectrum of 3l in THF (0.01 M) at room temperature

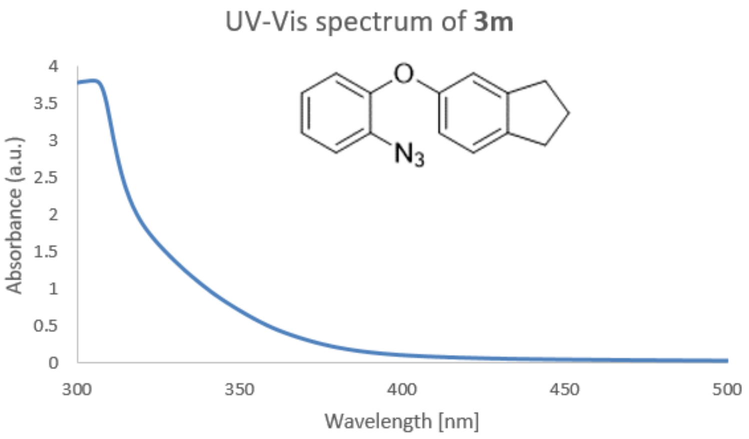

Supplementary Figure 137. UV-Vis spectrum of 3m in THF (0.01 M) at room temperature

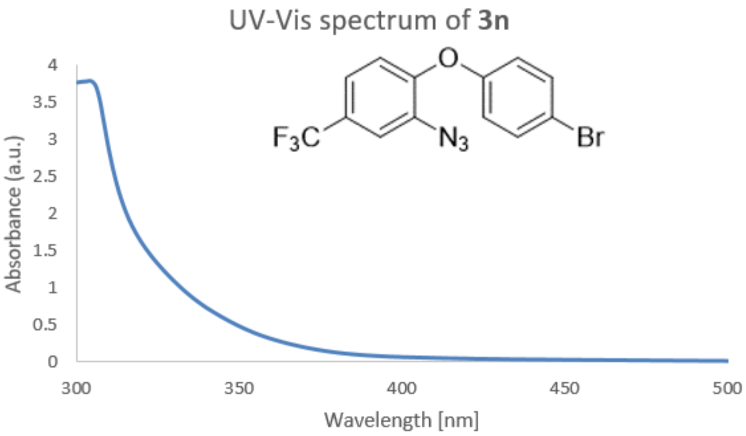

Supplementary Figure 138. UV-Vis spectrum of 3n in THF (0.01 M) at room temperature

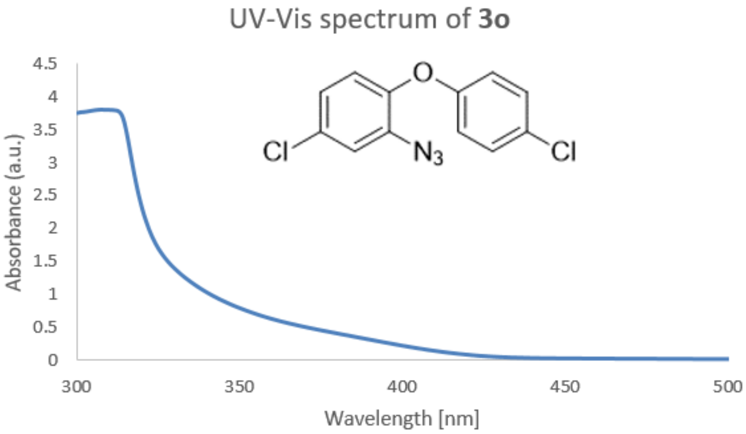

Supplementary Figure 139. UV-Vis spectrum of 3o in THF (0.01 M) at room temperature

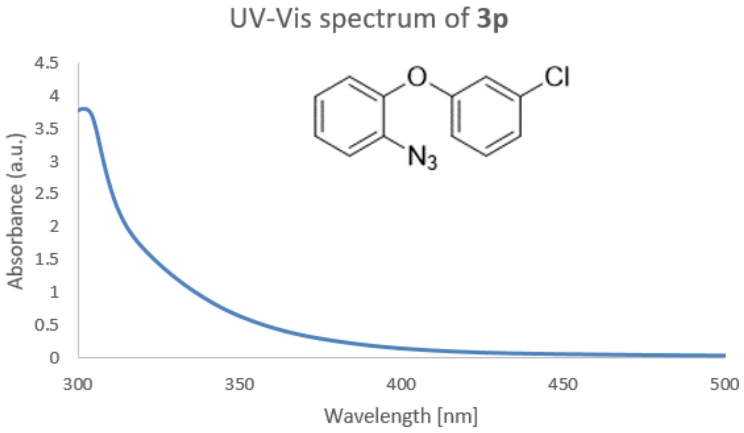

Supplementary Figure 140. UV-Vis spectrum of 3p in THF (0.01 M) at room temperature

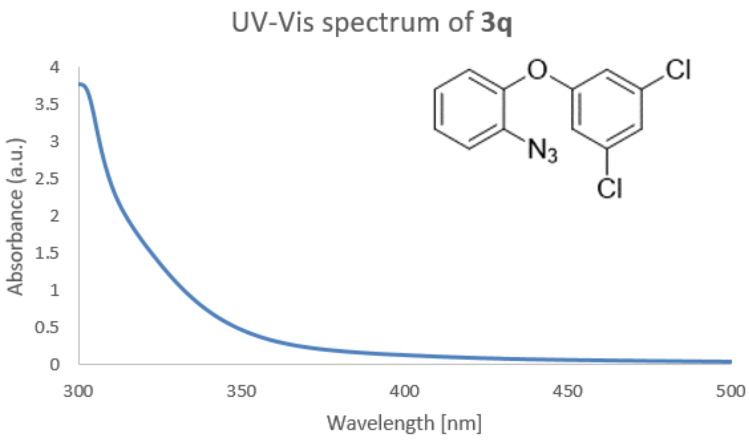

Supplementary Figure 141. UV-Vis spectrum of 3q in THF (0.01 M) at room temperature

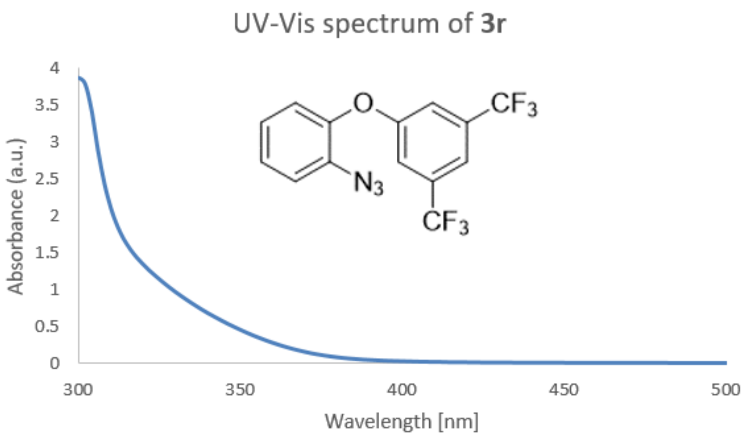

Supplementary Figure 142. UV-Vis spectrum of 3r in THF (0.01 M) at room temperature

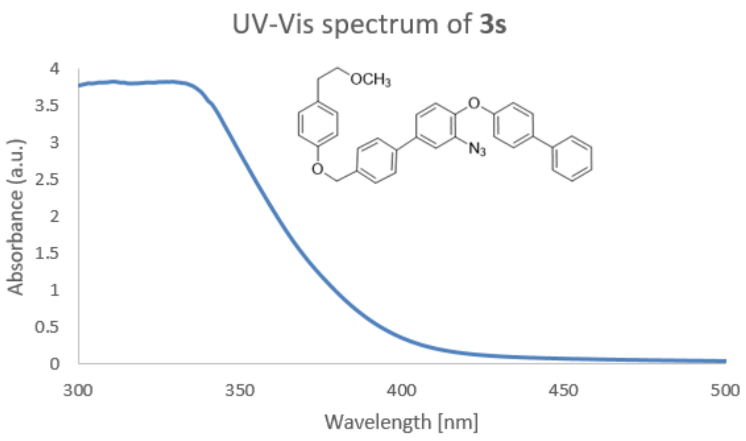

Supplementary Figure 143. UV-Vis spectrum of 3s in THF (0.01 M) at room temperature

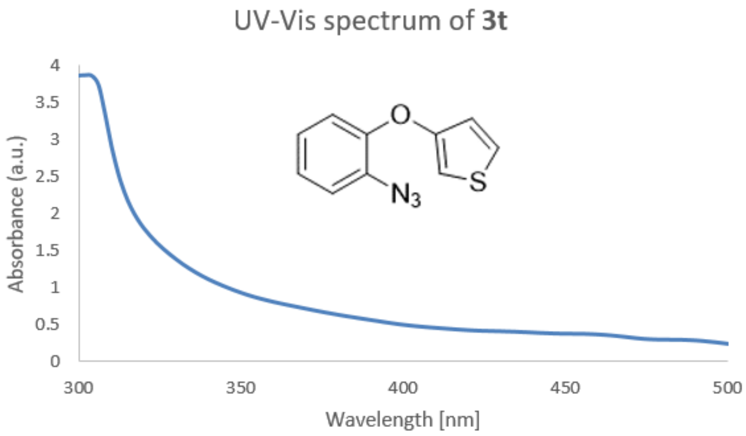

Supplementary Figure 144. UV-Vis spectrum of 3t in THF (0.01 M) at room temperature

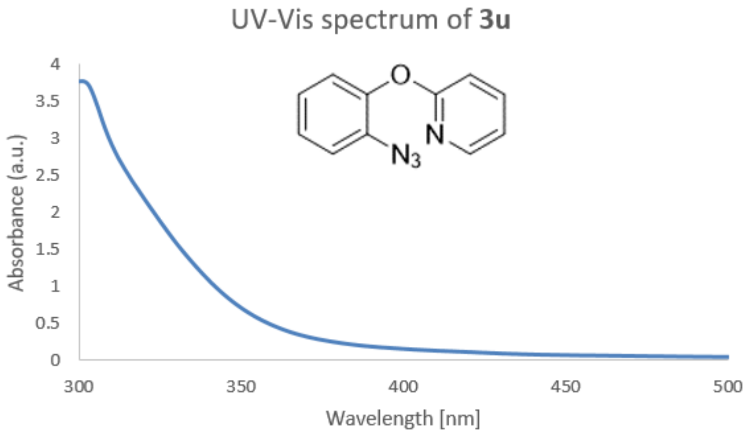

Supplementary Figure 145. UV-Vis spectrum of 3u in THF (0.01 M) at room temperature

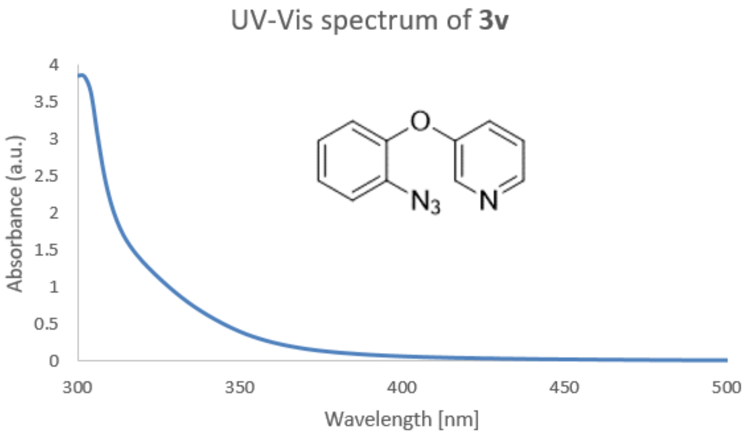

Supplementary Figure 146. UV-Vis spectrum of 3v in THF (0.01 M) at room temperature

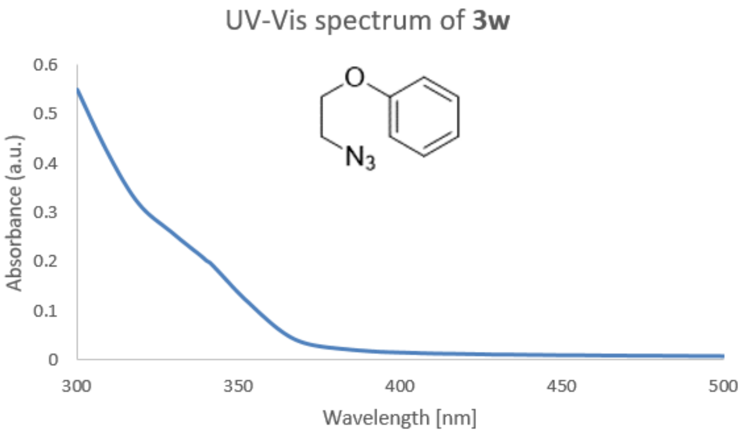

Supplementary Figure 147. UV-Vis spectrum of 3w in THF (0.01 M) at room temperature

5. Supplementary References

1) Xie, J., Sekine, K., Witzel, S., Krämer, P., Rudolph, M., Rominger, F. & Hashmi, A. S. K. Light-Induced Gold-Catalyzed Hiyama Arylation: A Coupling Access to Biarylboronates. *Angew. Chem. Int. Ed.* **57**, 16648–16653 (2018).
2) Jin, H., Zhou, J., Pu, T., Zhang, A., Gao, X., Tao, K. & Hou, T. Synthesis of novel fenfuram-diarylether hybrids as potent succinate dehydrogenase inhibitors. *Bioorg. Med. Chem. Lett.* **73**, 76–82 (2017).
3) Zhang, P. L., Zuckett, J. F., Woolfrey, J., Tran, K., Huang, B., Wong, P., Sinha, U., Park, G., Reed, A., Malinowski, J., Hollenbach, S., Scarborougha R. M. & Zhua, B. Y. Design, synthesis, and SAR of monobenzamidines and aminoisoquinolines as factor Xa inhibitors. *Bioorg. Med. Chem. Lett.* **12**, 1657–1661 (2002).
4) Bouider, N., Fhayli, W., Ghandour, Z., Boyer, M., Harrouche, K., Florence, X., Pirotte, B., Lebrun, P., Faury, G. & Khelili, S. Design and synthesis of new potassium channel activators derived from the ring opening of diazoxide: Study of their vasodilatory effect, stimulation of elastin synthesis and inhibitory effect on insulin release. *Bioorg. Med. Chem.* **23**, 1735–1746 (2015).
5) Mathur, D., Rana, N., Olsen, C. E., Parmar, V. S. & Prasad, A. K. Cu(I)-catalyzed efficient synthesis of 2′-triazolo-nucleoside conjugates. *J. Heterocycl. Chem.* **52**, 701–710 (2015).
6) Xia, Y., Qu, P., Liu, Z., Ge, R., Xiao, Q., Zhang, Y. & Wang, J. Catalyst-free intramolecular formal carbon insertion into σ-C–C bonds: a new approach toward phenanthrols and naphthols. *Angew. Chem. Int. Ed.* **52**, 2543–2546 (2013).
7) Travers, M. J., Cowles, D. C., Clifford, E. P. & Ellison, G. B. Photoelectron spectroscopy of the phenylnitrene anion. *J. Am. Chem. Soc.* **114**, 8699-8701 (1992).
8) Bannwarth, C. & Grimme, S. A simplified time-dependent density functional theory approach for electronic ultraviolet and circular dichroism spectra of very large molecules. *Computational and Theoretical Chemistry*, **1040**, 45-53 (2014).

9) Shao, Y., Gan, Z., Epifanovsky, E., Gilbert, A.T., Wormit, M., Kussmann, J., Lange, A.W., Behn, A., Deng, J., Feng, X. & Ghosh, D. Advances in molecular quantum chemistry contained in the Q-Chem 4 program package. *Molecular Physics,* **113**, 184-215 (2015).

10) Cossi, M., Rega, N., Scalmani, G. & Barone, V. Energies, structures, and electronic properties of molecules in solution with the C-PCM solvation model. *Journal of computational chemistry,* **24**, 669-681 (2003).

11) Frisch, M. J., Trucks, G. W., Schlegel, H. B., Scuseria, G. E., Robb, M. A., Cheeseman, J. R., Scalmani, G., Barone, V., Petersson, G. A., Nakatsuji, H., Li, X., Caricato, M., Marenich, A. V., Bloino, J., Janesko, B. G., Gomperts, R., Mennucci, B., Hratchian, H. P., Ortiz, J. V., Izmaylov, A. F., Sonnenberg, J. L., Williams-Young, D., Ding, F., Lipparini, F., Egidi, F., Goings, J., Peng, B., Petrone, A., Henderson, T., Ranasinghe, D., Zakrzewski, V. G., Gao, J., Rega, N., Zheng, G., Liang, W., Hada, M., Ehara, M., Toyota, K., Fukuda, R., Hasegawa, J., Ishida, M., Nakajima, T., Honda, Y., Kitao, O., Nakai, H., Vreven, T., Throssell, K., Montgomery, J. A., Peralta, J. E., Ogliaro, F., Bearpark, M. J., Heyd, J. J., Brothers, E. N., Kudin, K. N., Staroverov, V. N., Keith, T. A., Kobayashi, R., Normand, J., Raghavachari, K., Rendell, A. P., Burant, J. C., Iyengar, S. S., Tomasi, J., Cossi, M., Millam, J. M., Klene, M., Adamo, C., Cammi, R., Ochterski, J. W., Martin, R. L., Morokuma, K., Farkas, O., Foresman, J. B. & Fox, D. J. Gaussian 16, Revision A.03, Gaussian, Inc., Wallingford CT, 2016.

12) (a) Zhao, Y. & Truhlar, D. G. The M06 suite of density functionals for main group thermochemistry, thermochemical kinetics, noncovalent interactions, excited states, and transition elements: two new functionals and systematic testing of four M06-class functionals and 12 other functionals. *Theor. Chem. Acc.* **120**, 215-241 (2008); (b) Y. Zhao and D. G. Truhlar, Density functionals with broad applicability in chemistry. *Acc. Chem. Res.* **41**, 157-167 (2008).

13) (a) Lan, Y., Zou, L., Cao, Y. & Houk, K. N. Computational methods to calculate accurate activation and reaction energies of 1,3-dipolar cycloadditions of 24 1,3-dipoles. *J. Phys. Chem. A* **115**, 13906-13920 (2011); (b) Lopez, S. A., Munk, M. E. & Houk, K. N. Mechanisms and transition states of 1,3-dipolar cycloadditions of phenyl azide with enamines: a computational analysis. *J. Org. Chem.* **78**, 1576-1582 (2013); (c) Paton, R.S., Mackey, J. L., Kim, W. H., Lee, J. H., Danishefsky, S. J. & Houk, K. N. Origins of stereoselectivity in the trans Diels−Alder paradigm. *J. Am. Chem. Soc.* **132**, 9335-9340 (2010); (d) Pieniazek, S. N., Clemente, F. R. & Houk, K. N. Sources of error in DFT computations of C–C bond formation thermochemistries: $\pi \rightarrow \sigma$ transformations and error cancellation by DFT methods. *Angew. Chem. Int. Ed.* **47**, 7746-7749 (2008); (e) James, N. C., Um, J. M., Padias, A. B., Jr Hall, J. K. & Houk, K. N. Computational investigation of the competition between the concerted Diels–Alder reaction and formation of diradicals in reactions of acrylonitrile with nonpolar dienes. *J. Org. Chem.* **78**, 6582-6592 (2013); (f) Jalali, M., Ho, C. C., Fuller, R. O., Lucas, N.T., Ariafard, A. & Bissember, A. C. Photochemical activation of a hydroxyquinone-derived phenyliodonium ylide by visible light: synthetic and mechanistic investigations. *J. Org. Chem.* **86**, 1758–1768 (2020).

14) Marenich, A. V., Cramer, C. J. & Truhlar, D. G. Universal solvation model based on solute electron density and on a continuum model of the solvent defined by the bulk dielectric constant and atomic surface tensions. *J. Phys. Chem. B* **113**, 6378-6396 (2009).

15) Hariharan, P. C. & Pople, J. A. The influence of polarization functions on molecular orbital hydrogenation energies. *Theor. Chem. Acta.* **28**, 213-222 (1973).

16) (a) Fukui, K. A formulation of the reaction coordinate. *J. Chem. Phys.* **74**, 4161-4163 (1970); (b) Fukui, K. The path of chemical reactions-the IRC approach. *Acc. Chem. Res.* **14**, 363-368 (1981).

17) Weigend, F., Furche, F. & Ahlrichs, R. J. Gaussian basis sets of quadruple zeta valence quality for atoms H–Kr. *J. Chem. Phys.* **119**, 12753-12762 (2003).

18) Harvey, J. N., Aschi, M., Schwarz, H. & Koch, W. The singlet and triplet states of phenyl cation. A hybrid approach for locating minimum energy crossing points between non-interacting potential energy surfaces. *Theor. Chem. Acc.* **99**, 95-99 (1998).

19) (a) Yamaguchi, K., Takahara, Y., Fueno, T. & Houk, K. N. Extended Hartree-Fock (EHF) theory of chemical reactions. *Theor. Chim. Acta* **73**, 337-364 (1988); (b) Kawakami, T., Nagao, H. & Yamaguchi, K. Effective exchange integrals for open-shell species by density functional methods. *Chem. Phys. Lett.* **231**, 25-33 (1994).

20) Hartwig, J. F., Cook, K. S., Hapke, M., Incarvito, C. D., Fan, Y., Webster, C. E. & Hall, M. B. Rhodium boryl complexes in the catalytic, terminal functionalization of alkanes. *J. Am. Chem. Soc.* **127**, 2538−2552 (2005).
