# Supplementary material for: An unexpected synthesis of azepinone derivatives through a metal-free photochemical cascade reaction
Source: Nat Commun. 2023 Feb 14;14:831. doi: 10.1038/s41467-023-36190-z (PMC9929248; doi:10.1038/s41467-023-36190-z)
Supplement: Supplementary file 2 — Description of Additional Supplementary Files [file 41467_2023_36190_MOESM2_ESM.docx]

**Description of Additional Supplementary Files**

**File name**: **Supplementary Data 1**

Description: Supplementary Table 1. (Calculated by Q-chem) Total potential (E), Total Enthalpy (H), and Total Entropy (S) of all structures optimized at the CAM-B3LYP-CPCM/6-31G(d) level of theory along with the total potential energies calculated by CAM-B3LYP-CPCM/def2-TZVP//CAM-B3LYP-CPCM/6-31G(d) and Cartesian coordinates for all of the calculated structures.

**File name**: **Supplementary Data 2**

Description: Supplementary Table 2 (Calculated by Gaussian). Total potential (E), enthalpy (H) and Gibbs free energies (G) of all structures optimized at the M06-2X-SMD/6-31G(d) level of theory along with the total potential energies calculated by M06-2X-SMD/def2-TZVP//M06-2X-SMD/6-31G(d) and Cartesian coordinates for all of the calculated structures.
