# Supplementary material for: An unexpected synthesis of azepinone derivatives through a metal-free photochemical cascade reaction
Source: Nat Commun. 2023 Feb 14;14:831. doi: 10.1038/s41467-023-36190-z (PMC9929248; doi:10.1038/s41467-023-36190-z)
Supplement: Supplementary file 3 — Dataset 1 [file 41467_2023_36190_MOESM3_ESM.docx]

**Supplementary Table 1**. **(Calculated by Q-chem) Total potential (E), Total Enthalpy (H), and Total Entropy (S) of all structures optimized at the CAM-B3LYP-CPCM/6-31G(d) level of theory along with the total potential energies calculated by CAM-B3LYP-CPCM/def2-TZVP//CAM-B3LYP-CPCM/6-31G(d) and Cartesian coordinates for all of the calculated structures.**

**3a**

E (CAM-B3LYP-SMD/6-31G(D)) = -1161.3405832376 au

Total Enthalpy (CAM-B3LYP-SMD/6-31G(D)) = 123.159 kcal/mol

Total Entropy (CAM-B3LYP-SMD/6-31G(D)) = 126.484 cal/mol.K

E (CAM-B3LYP-SMD/DEF2-TZVP//CAM-B3LYP-SMD/6-31G(D)) = -1161.6421296282 au

O 1.1164764 0.7995249 -0.5536557

C -0.0321721 0.0847871 -0.3697264

C -1.1954003 0.8180163 -0.0990040

C -0.1024678 -1.2950343 -0.5106236

C -2.4149243 0.1581465 0.0283712

C -1.3159045 -1.9586534 -0.3759467

H 0.7986512 -1.8559110 -0.7303622

C -2.4589714 -1.2206035 -0.1091214

H -3.3216118 0.7152867 0.2375579

H -1.3660482 -3.0355694 -0.4872542

C 2.3358614 0.2662510 -0.1832533

C 2.5332781 -0.3081838 1.0684718

C 3.3848676 0.4047665 -1.0825522

C 3.8030931 -0.7583163 1.4110988

H 1.7054876 -0.3966259 1.7642024

C 4.6521561 -0.0409547 -0.7230716

H 3.1992921 0.8631008 -2.0481664

C 4.8650833 -0.6268927 0.5205791

H 3.9623828 -1.2075383 2.3866125

H 5.4745892 0.0680414 -1.4233345

H 5.8545721 -0.9769041 0.7969534

Cl -4.0003506 -2.0384204 0.0572873

N -1.0402250 2.2173127 0.0282532

N -2.0543059 2.8806308 0.2538941

N -2.9050583 3.5969070 0.4603773

**MECP S_0_/S_1_**

E (CAM-B3LYP-SMD/DEF2-TZVP) = -1161.5492184530 au

O 1.0710828 0.9403230 0.0030554

C -0.0088135 0.1497932 -0.0075578

C -1.2822332 0.8468124 0.0080533

C 0.0374761 -1.2399166 -0.0333598

C -2.4791380 0.0490483 0.0030420

C -1.1403842 -1.9723922 -0.0393520

H 0.9911239 -1.7538677 -0.0494509

C -2.3861531 -1.3145838 -0.0194485

H -3.4397974 0.5451624 0.0151358

H -1.1008456 -3.0564284 -0.0598842

C 2.3452853 0.3773559 0.0055097

C 2.9350191 0.0430644 1.2175498

C 3.0138040 0.2392057 -1.2038888

C 4.2378803 -0.4492618 1.2115714

H 2.3828737 0.1749497 2.1433239

C 4.3161679 -0.2533325 -1.1940376

H 2.5199345 0.5215398 -2.1286752

C 4.9279319 -0.5981041 0.0099777

H 4.7139499 -0.7123807 2.1518893

H 4.8530814 -0.3640775 -2.1317788

H 5.9450404 -0.9795111 0.0122854

Cl -3.8441778 -2.2858078 -0.0253218

N -1.2236063 2.1663794 0.0245218

N -2.6919949 2.7511989 0.0319328

N -2.8338291 3.8946472 0.0441950

**3a***

E (CAM-B3LYP-SMD/6-31G(D)) = -1161.2737713852 au

Total Enthalpy (CAM-B3LYP-SMD/6-31G(D)) = 121.692 kcal/mol

Total Entropy (CAM-B3LYP-SMD/6-31G(D)) = 124.876 cal/mol.K

E (CAM-B3LYP-SMD/DEF2-TZVP//CAM-B3LYP-SMD/6-31G(D)) = -1161.5720742268 au

O 1.0574583 0.9337847 0.0740293

C -0.0267229 0.1438956 0.0166426

C -1.2912650 0.8269489 0.0207679

C 0.0204358 -1.2437091 -0.0395525

C -2.4782971 0.0424534 -0.0316688

C -1.1529447 -1.9778464 -0.0903145

H 0.9746421 -1.7550075 -0.0423144

C -2.3913094 -1.3237368 -0.0857579

H -3.4383358 0.5404204 -0.0281460

H -1.1111331 -3.0601018 -0.1326512

C 2.3336100 0.3744879 0.0469178

C 2.9751567 0.1117308 1.2478600

C 2.9556208 0.1753961 -1.1766434

C 4.2796278 -0.3705316 1.2174334

H 2.4593222 0.2902689 2.1854642

C 4.2597730 -0.3078052 -1.1924350

H 2.4256600 0.4030966 -2.0955693

C 4.9217830 -0.5813525 0.0009329

H 4.7948189 -0.5776387 2.1500496

H 4.7598088 -0.4657239 -2.1427637

H 5.9404456 -0.9554503 -0.0172413

Cl -3.8522017 -2.2835124 -0.1504680

N -1.2324116 2.1660557 0.0741509

N -2.5479920 2.7368523 0.0710144

N -2.7758785 3.8768277 0.1106597

**TS^S1^**

E (CAM-B3LYP-SMD/6-31G(D)) = -1161.2616142910 au

Total Enthalpy (CAM-B3LYP-SMD/6-31G(D)) = 121.636 kcal/mol

Total Entropy (CAM-B3LYP-SMD/6-31G(D)) = 125.281 cal/mol.K

E (CAM-B3LYP-SMD/DEF2-TZVP//CAM-B3LYP-SMD/6-31G(D)) = -1161.5612052267 au

O 1.0687313 0.9343492 0.0484098

C -0.0163726 0.1440075 0.0063872

C -1.2824734 0.8368089 0.0183586

C 0.0278831 -1.2421923 -0.0445687

C -2.4718851 0.0474906 -0.0216207

C -1.1474643 -1.9755302 -0.0828820

H 0.9814175 -1.7549774 -0.0538967

C -2.3840139 -1.3182769 -0.0707659

H -3.4324373 0.5428485 -0.0122725

H -1.1080656 -3.0578879 -0.1217136

C 2.3440267 0.3726376 0.0325528

C 2.9723330 0.1000303 1.2384287

C 2.9783063 0.1802372 -1.1856524

C 4.2757669 -0.3853956 1.2182924

H 2.4473393 0.2734700 2.1719114

C 4.2814780 -0.3060359 -1.1911640

H 2.4582858 0.4153030 -2.1083725

C 4.9301782 -0.5895069 0.0071259

H 4.7806628 -0.6002103 2.1547841

H 4.7909651 -0.4589045 -2.1372892

H 5.9480079 -0.9661669 -0.0028767

Cl -3.8469110 -2.2772149 -0.1197229

N -1.2123082 2.1656114 0.0662466

N -2.6598381 2.7563878 0.0713592

N -2.8639412 3.8829204 0.1093377

**MECP S_1_/T_1_**

E (CAM-B3LYP-SMD/DEF2-TZVP) = -1161.5446618495 au

O 1.0344967 0.9033651 0.0484278

C -0.0233723 0.1303769 0.0052588

C -1.3121907 0.8570674 0.0194376

C 0.0266699 -1.2668587 -0.0487392

C -2.5032663 0.0381367 -0.0238332

C -1.1360587 -1.9804859 -0.0876151

H 0.9827962 -1.7737306 -0.0582753

C -2.3942527 -1.3071113 -0.0742510

H -3.4627509 0.5339078 -0.0140576

H -1.1098102 -3.0631302 -0.1282487

C 2.3303489 0.3591022 0.0331840

C 2.9548932 0.1074322 1.2430134

C 2.9566021 0.1772468 -1.1882878

C 4.2638540 -0.3633344 1.2204934

H 2.4296072 0.2796868 2.1763779

C 4.2653247 -0.2944890 -1.1910207

H 2.4330257 0.4033681 -2.1111241

C 4.9168911 -0.5658414 0.0084137

H 4.7721333 -0.5697022 2.1569972

H 4.7748515 -0.4469481 -2.1371375

H 5.9383904 -0.9324395 -0.0014985

Cl -3.8305885 -2.2958180 -0.1267887

N -1.2319710 2.1495253 0.0700887

N -2.5416325 2.7333562 0.0780152

N -2.6743199 3.9071201 0.1215667

**T_1_**

E (CAM-B3LYP-SMD/6-31G(D)) = -1161.2882206993 au

Total Enthalpy (CAM-B3LYP-SMD/6-31G(D)) = 121.046 kcal/mol

Total Entropy (CAM-B3LYP-SMD/6-31G(D)) = 127.235 cal/mol.K

E (CAM-B3LYP-SMD/DEF2-TZVP//CAM-B3LYP-SMD/6-31G(D)) = -1161.5860591220 au

O -1.0622211 0.9272257 -0.0066415

C 0.0318619 0.1445627 -0.0040836

C 1.2910079 0.8412565 -0.0021572

C -0.0010933 -1.2442091 -0.0040284

C 2.4880986 0.0660726 -0.0003706

C 1.1803329 -1.9695621 -0.0021722

H -0.9504559 -1.7647873 -0.0056655

C 2.4144962 -1.2997979 -0.0003460

H 3.4416296 0.5768566 0.0010516

H 1.1509653 -3.0531652 -0.0023119

C -2.3307289 0.3526891 -0.0022529

C -2.9656316 0.1159659 -1.2124856

C -2.9558429 0.1117069 1.2122949

C -4.2644145 -0.3818273 -1.2009963

H -2.4488937 0.3268662 -2.1428967

C -4.2546151 -0.3861145 1.2095476

H -2.4316671 0.3193662 2.1392730

C -4.9090031 -0.6337528 0.0064514

H -4.7737961 -0.5684125 -2.1411395

H -4.7563963 -0.5760182 2.1531057

H -5.9233622 -1.0198212 0.0098925

Cl 3.8847469 -2.2466318 0.0019652

N 1.2215667 2.1787195 -0.0020343

N 2.5291028 2.7705308 0.0000536

N 2.6635930 3.9383362 0.0003777

**TS^T1^**

E (CAM-B3LYP-SMD/6-31G(D)) = -1161.2874439316 au

Total Enthalpy (CAM-B3LYP-SMD/6-31G(D)) = 120.094 kcal/mol

Total Entropy (CAM-B3LYP-SMD/6-31G(D)) = 126.560 cal/mol.K

E (CAM-B3LYP-SMD/DEF2-TZVP//CAM-B3LYP-SMD/6-31G(D)) = -1161.5854328472 au

O 1.0675299 0.9357157 0.0001156

C -0.0201623 0.1413530 0.0000287

C -1.2862232 0.8328998 0.0000770

C 0.0242650 -1.2453918 -0.0000994

C -2.4767818 0.0436879 -0.0000050

C -1.1516846 -1.9823062 -0.0001770

H 0.9782269 -1.7576259 -0.0001387

C -2.3896905 -1.3220792 -0.0001277

H -3.4361253 0.5423236 0.0000321

H -1.1131014 -3.0655102 -0.0002756

C 2.3406108 0.3724872 0.0000687

C 2.9727778 0.1380507 1.2123564

C 2.9728222 0.1383764 -1.2122585

C 4.2753921 -0.3497579 1.2052816

H 2.4506495 0.3428231 2.1411378

C 4.2754361 -0.3494339 -1.2052674

H 2.4507278 0.3433986 -2.1410037

C 4.9268512 -0.5944652 -0.0000138

H 4.7823346 -0.5343513 2.1471505

H 4.7824127 -0.5337754 -2.1471673

H 5.9441752 -0.9726839 -0.0000460

Cl -3.8534308 -2.2803157 -0.0002271

N -1.2185734 2.1651094 0.0001979

N -2.6604801 2.7694883 0.0002276

N -2.7986287 3.9149081 0.0003742
