# Supplementary material for: An unexpected synthesis of azepinone derivatives through a metal-free photochemical cascade reaction
Source: Nat Commun. 2023 Feb 14;14:831. doi: 10.1038/s41467-023-36190-z (PMC9929248; doi:10.1038/s41467-023-36190-z)
Supplement: Supplementary file 4 — Dataset 2 [file 41467_2023_36190_MOESM4_ESM.docx]

**Supplementary Table 2 (Calculated by Gaussian). Total potential (E), enthalpy (H) and Gibbs free energies (G) of all structures optimized at the M06-2X-SMD/6-31G(d) level of theory along with the total potential energies calculated by M06-2X-SMD/def2-TZVP//M06-2X-SMD/6-31G(d) and Cartesian coordinates for all of the calculated structures.**

**3a**

E (M06-2X-SMD/6-31G(D)) = -1161.39004941 au

H (M06-2X-SMD/6-31G(D)) = -1161.194254 au

G (M06-2X-SMD/6-31G(D)) = -1161.253663 au

E (M06-2X-SMD/DEF2-TZVP//M06-2X-SMD/6-31G(D)) = -1161.69261252 au

O 1.12127200 0.83908600 -0.55889400

C -0.02037800 0.11285300 -0.37200900

C -1.19170800 0.83574600 -0.09755100

C -0.07457200 -1.27006400 -0.50799400

C -2.40531400 0.16266100 0.04239800

C -1.28128100 -1.94774800 -0.36259200

H 0.83413400 -1.81949200 -0.73112400

C -2.43184500 -1.21969900 -0.08905400

H -3.31919100 0.70882700 0.25804400

H -1.32260500 -3.02582000 -0.46955500

C 2.33398700 0.28948900 -0.18952500

C 2.51280500 -0.30438800 1.05855400

C 3.38955500 0.41840300 -1.08517700

C 3.77350600 -0.78315200 1.40255000

H 1.67594200 -0.38556400 1.74581100

C 4.64803800 -0.05734900 -0.72370400

H 3.21447600 0.89043400 -2.04657900

C 4.84321400 -0.66233200 0.51592300

H 3.91986500 -1.24661100 2.37345700

H 5.47581400 0.04238500 -1.41911500

H 5.82406000 -1.03576500 0.79230900

Cl -3.95587600 -2.05664400 0.09470900

N -1.04440200 2.23824000 0.01994500

N -2.08110100 2.87578000 0.23547200

N -2.96004400 3.55388800 0.43128800

**N_2_**

E (M06-2X-SMD/6-31G(D)) = -109.482008174 au

H (M06-2X-SMD/6-31G(D)) = -109.472900 au

G (M06-2X-SMD/6-31G(D)) = -109.494642 au

E (M06-2X-SMD/DEF2-TZVP//M06-2X-SMD/6-31G(D)) = -109.530028995 au

N 0.00000000 0.00000000 0.54918000

N 0.00000000 0.00000000 -0.54918000

**A^T^**

E (M06-2X-SMD/6-31G(D)) = -1051.9011203 au

H (M06-2X-SMD/6-31G(D)) = -1051.719514 au

G (M06-2X-SMD/6-31G(D)) = -1051.774342 au

E (M06-2X-SMD/DEF2-TZVP//M06-2X-SMD/6-31G(D)) = -1052.15656228 au

O 0.96435600 1.24545900 -0.00589200

C -0.23802600 0.62450800 -0.00830100

C -1.36823200 1.50739500 0.03798700

C -0.42528200 -0.75002000 -0.05760900

C -2.67724400 0.94327600 0.03238900

C -1.71449800 -1.27832300 -0.06128700

H 0.42860000 -1.41708300 -0.09404000

C -2.82302100 -0.42356200 -0.01624800

H -3.53170300 1.61027300 0.06694800

H -1.85822600 -2.35251000 -0.10052500

C 2.11242700 0.46099800 0.00127700

C 2.62455400 0.02117000 1.21615100

C 2.74137400 0.18268800 -1.20549700

C 3.80455900 -0.71872100 1.21663700

H 2.10367100 0.26314700 2.13756000

C 3.92230400 -0.55656500 -1.19043600

H 2.30933500 0.54769900 -2.13195300

C 4.45307600 -1.00799900 0.01671400

H 4.21807100 -1.06640200 2.15813700

H 4.42724200 -0.77807900 -2.12551400

H 5.37357500 -1.58304400 0.02296100

Cl -4.42776000 -1.11679200 -0.02254000

N -1.19792000 2.82410300 0.08658400

**B^T^**

E (M06-2X-SMD/6-31G(D)) = -1051.89972506 au

H (M06-2X-SMD/6-31G(D)) = -1051.718710 au

G (M06-2X-SMD/6-31G(D)) = -1051.769697 au

E (M06-2X-SMD/DEF2-TZVP//M06-2X-SMD/6-31G(D)) = -1052.15519165 au

O 0.92678200 1.53179700 0.28705300

C -0.30659600 0.96753200 0.22057700

C -0.46240000 -0.44994600 0.12456200

C -1.40365100 1.81667300 0.20449400

C -1.78241600 -0.95511600 -0.05510900

C -2.68235300 1.29357900 0.05834000

H -1.24556200 2.88656200 0.28942600

C -2.85149800 -0.09320300 -0.08310500

H -1.90692400 -2.02923100 -0.14347800

H -3.54419000 1.95149300 0.04000700

C 2.00277500 0.69135900 0.12264800

C 1.82734700 -0.71164800 0.60596400

C 3.15714000 1.18334100 -0.40103500

C 2.99592500 -1.57386100 0.23597800

H 1.77094000 -0.67526900 1.72014400

C 4.26260700 0.32399800 -0.59321700

H 3.20311300 2.22929800 -0.68919900

C 4.13827900 -1.05831200 -0.29253900

H 2.89153100 -2.63394300 0.44602900

H 5.18139500 0.71380500 -1.01470100

H 4.97148100 -1.72050200 -0.51013000

Cl -4.46878900 -0.72551800 -0.28906500

N 0.55177500 -1.32131100 0.22774700

**B**

E (M06-2X-SMD/6-31G(D)) = -1051.90951209 au

H (M06-2X-SMD/6-31G(D)) = -1051.726076 au

G (M06-2X-SMD/6-31G(D)) = -1051.775511 au

E (M06-2X-SMD/DEF2-TZVP//M06-2X-SMD/6-31G(D)) = -1052.1662205 au

O 0.91785800 1.49908800 0.04662500

C -0.32285600 0.94128600 0.26181600

C -0.42181500 -0.45663500 -0.00314200

C -1.41915700 1.78820700 0.40657700

C -1.74657800 -0.93761000 -0.24711400

C -2.69033600 1.26346100 0.28522300

H -1.26007900 2.84534800 0.59592000

C -2.81585100 -0.09826300 -0.06444200

H -1.87941200 -1.98144500 -0.50986700

H -3.57033700 1.88143900 0.41505600

C 1.95254600 0.69921700 0.03880100

C 1.77449000 -0.63216600 0.66772900

C 3.14234200 1.13726700 -0.58261500

C 2.98586400 -1.49538700 0.52190200

H 1.52631700 -0.47331500 1.73986900

C 4.20237700 0.27893900 -0.60138900

H 3.17780300 2.12041500 -1.03912000

C 4.12024600 -1.05730900 -0.05258600

H 2.89437000 -2.51054500 0.89718300

H 5.12542700 0.58612400 -1.08384800

H 4.98298000 -1.70809600 -0.14664300

Cl -4.43631900 -0.73951800 -0.27703300

N 0.64426400 -1.25240900 -0.04522100

**C**

E (M06-2X-SMD/6-31G(D)) = -1051.93854774 au

H (M06-2X-SMD/6-31G(D)) = -1051.754787 au

G (M06-2X-SMD/6-31G(D)) = -1051.803214 au

E (M06-2X-SMD/DEF2-TZVP//M06-2X-SMD/6-31G(D)) = -1052.19305999 au

O 0.89623600 1.66736900 0.23420900

C -0.37809700 1.17656000 0.19028900

C -0.37505300 -0.17018500 -0.17715900

C -1.54377600 1.87071700 0.44396100

C -1.55490400 -0.87255900 -0.32921000

C -2.74622300 1.16809000 0.30293400

H -1.53207900 2.91509600 0.73433600

C -2.73675500 -0.16971600 -0.07667100

H -1.56120800 -1.91619900 -0.62311200

H -3.69005400 1.66928800 0.48758600

C 1.74745300 0.57806300 -0.05429100

C 1.75041100 -0.59732600 0.89353200

C 2.96370900 0.89790300 -0.80371900

C 2.97228100 -1.40989000 0.91909800

H 1.15279100 -0.51724400 1.79802100

C 3.98527500 0.02266400 -0.79317800

H 3.00177300 1.84412400 -1.33371400

C 3.98045600 -1.15769800 0.06124600

H 3.05620900 -2.18964100 1.67016200

H 4.88007700 0.23231900 -1.37119100

H 4.86994100 -1.78030900 0.07180200

Cl -4.26198400 -1.01548700 -0.24031100

N 0.95967500 -0.62183400 -0.38332200

**D**

E (M06-2X-SMD/6-31G(D)) = -1051.95315983 au

H (M06-2X-SMD/6-31G(D)) = -1051.768805 au

G (M06-2X-SMD/6-31G(D)) = -1051.818546 au

E (M06-2X-SMD/DEF2-TZVP//M06-2X-SMD/6-31G(D)) = -1052.21128551 au

O 0.80379100 1.93092100 0.17586900

C -0.44596000 1.36864000 0.09169100

C -0.32374500 -0.01026500 -0.07483500

C -1.66094900 2.00508000 0.17838700

C -1.43865800 -0.82431000 -0.15310000

C -2.80522600 1.19737400 0.09146200

H -1.73465700 3.07778200 0.31579900

C -2.67698500 -0.17668600 -0.06452300

H -1.36390700 -1.89803500 -0.28268400

H -3.79022600 1.64607300 0.15044000

C 1.72378300 0.92714700 0.01711000

C 1.55489100 -1.48090900 0.50628400

C 3.04173900 1.15859400 -0.07040100

C 2.82473600 -1.87785100 0.42589900

H 0.81482800 -2.02766000 1.08527300

C 4.01903700 0.13938500 -0.44255600

H 3.36486000 2.18780800 0.03349300

C 3.91826200 -1.19080000 -0.27417900

H 3.07482700 -2.79361000 0.95539000

H 4.92138100 0.52254800 -0.91505400

H 4.73589200 -1.81850200 -0.61851400

Cl -4.12537400 -1.15578000 -0.16061000

N 1.04178300 -0.30112800 -0.11259500

**G**

E (M06-2X-SMD/6-31G(D)) = -1947.12964809 au

H (M06-2X-SMD/6-31G(D)) = -1946.785669 au

G (M06-2X-SMD/6-31G(D)) = -1946.860733 au

E (M06-2X-SMD/DEF2-TZVP//M06-2X-SMD/6-31G(D)) = -1947.67078417 au

O 1.72154300 -1.51818100 -1.56881700

C 0.39453600 -1.73645800 -1.27951400

C 0.16677100 -1.37223200 0.03842900

C -0.59289000 -2.24224700 -2.10330100

C -1.07492600 -1.50673100 0.63999800

C -1.85252900 -2.36572400 -1.52687600

H -0.40086400 -2.51828000 -3.13322300

C -2.07131200 -2.00675600 -0.18851200

H -1.26219300 -1.22994900 1.67097300

H -2.67923500 -2.74661700 -2.11587600

C 2.26177800 -0.96507000 -0.50394000

C 1.69337000 -0.50435300 1.83234000

C 3.66068100 -0.51324900 -0.42038600

C 2.85958200 -0.76794000 2.43823500

H 0.86068500 -0.01246400 2.32053100

C 4.36552100 -1.41175600 0.57335400

H 4.10785000 -0.54632000 -1.41305700

C 3.99910900 -1.47203800 1.86174000

H 2.92958200 -0.46930000 3.48014600

H 5.19529600 -2.01064700 0.21114000

H 4.58890400 -2.07253700 2.54969100

Cl -3.67718900 -2.18597700 0.46400500

N 1.40523700 -0.91194800 0.50942800

C -3.16441900 1.43857600 0.66383100

C -2.18854300 1.84627100 1.58036500

C -0.87553500 2.07565600 1.17653400

C -0.52077100 1.88793600 -0.15742200

C -1.47416200 1.46701800 -1.08182200

C -2.78536200 1.24602000 -0.66890600

H -2.46527100 1.99490200 2.62192700

H -0.11950400 2.39685800 1.88715200

H -1.18245100 1.29900600 -2.11488600

H -3.52799700 0.91210500 -1.39011800

C -4.58903900 1.23338500 1.10922500

H -4.63834900 0.60106900 2.00149600

H -5.05622900 2.19186700 1.36298700

H -5.18777900 0.76510500 0.32378500

S 1.15211300 2.23400500 -0.70413500

O 1.98663400 2.10693300 0.51408900

O 1.12676000 3.58694000 -1.28201400

O 1.43894500 1.16635800 -1.69841800

H 3.61706800 0.52811800 -0.06957300

**(H_2_O)_4_**

E (M06-2X-SMD/6-31G(D)) = -305.573718565 au

H (M06-2X-SMD/6-31G(D)) = -305.464048 au

G (M06-2X-SMD/6-31G(D)) = -305.506909 au

E (M06-2X-SMD/DEF2-TZVP//M06-2X-SMD/6-31G(D)) = -305.767229042 au

O -1.90469100 0.01222000 -0.01279000

H -2.36856700 0.07797500 0.83504300

O -0.01247300 -1.90434700 0.01280400

H -0.07719900 -2.36744600 -0.83553300

H -1.28403700 0.78547500 -0.02362300

H -0.78571900 -1.28366800 0.02330500

O 1.90473600 -0.01250200 -0.01275900

H 1.28374500 -0.78552200 -0.02315900

H 2.36873700 -0.07801300 0.83501000

O 0.01230900 1.90437200 0.01277800

H 0.78560900 1.28375000 0.02301800

H 0.07838400 2.36950700 -0.83432900

**(H_2_O)_3_**

E (M06-2X-SMD/6-31G(D)) = -229.165221352 au

H (M06-2X-SMD/6-31G(D)) = -229.084042 au

G (M06-2X-SMD/6-31G(D)) = -229.123880 au

E (M06-2X-SMD/DEF2-TZVP//M06-2X-SMD/6-31G(D)) = -229.314812382 au

O 1.07885700 -1.39086100 0.01132800

H 1.27317000 -0.44478700 -0.11180900

H 0.11181300 -1.37747400 0.07670300

O 0.75651000 1.44900000 -0.10464100

H -0.15550700 1.10320500 -0.05567900

H 0.95816000 1.72005300 0.80326600

O -1.56934800 -0.14412200 0.01297000

H -2.22102900 -0.10511500 0.73067400

H -2.09475900 -0.20801600 -0.80041000

**H_2_O**

E (M06-2X-SMD/6-31G(D)) = -76.3801705457 au

H (M06-2X-SMD/6-31G(D)) = -76.355097 au

G (M06-2X-SMD/6-31G(D)) = -76.376541 au

E (M06-2X-SMD/DEF2-TZVP//M06-2X-SMD/6-31G(D)) = -76.4328325464 au

O 0.00000000 0.00000000 0.11969300

H 0.00000000 0.76094200 -0.47877300

H 0.00000000 -0.76094200 -0.47877300

**TsOH**

E (M06-2X-SMD/6-31G(D)) = -895.133145429 au

H (M06-2X-SMD/6-31G(D)) = -894.977791 au

G (M06-2X-SMD/6-31G(D)) = -895.028469 au

E (M06-2X-SMD/DEF2-TZVP//M06-2X-SMD/6-31G(D)) = -895.421639531 au

C 2.65036500 0.00048700 0.01955000

C 1.93634000 -1.20405400 -0.01422000

C 0.54889800 -1.21241800 -0.06287600

C -0.12364600 0.00754600 -0.07394200

C 0.55356600 1.22238100 -0.04320100

C 1.94306900 1.20688000 0.00533500

H 2.47758900 -2.14606400 -0.00449600

H -0.00472000 -2.14537000 -0.09626000

H 0.00424000 2.15809500 -0.06087900

H 2.48784800 2.14627800 0.03076200

C 4.15463200 -0.01013100 0.04223400

H 4.53313300 -0.81563400 0.67783700

H 4.54990500 -0.17438800 -0.96673100

H 4.55295000 0.93991900 0.40676300

S -1.88414300 0.00995500 -0.12023900

O -2.37806900 -1.21544200 -0.72509900

O -2.36873000 1.28742300 -0.61220900

O -2.16641800 -0.07009600 1.47309000

H -3.12826500 -0.20134300 1.61329100

**TS_A_^T^**

E (M06-2X-SMD/6-31G(D)) = -1051.87523047 au

H (M06-2X-SMD/6-31G(D)) = -1051.695373 au

G (M06-2X-SMD/6-31G(D)) = -1051.746357 au

E (M06-2X-SMD/DEF2-TZVP//M06-2X-SMD/6-31G(D)) = -1052.13016121 au

O 0.91758900 1.51254700 0.34954900

C -0.30271200 0.92513000 0.26612400

C -0.45698300 -0.48045100 0.02373700

C -1.40891300 1.75928000 0.36943000

C -1.78801600 -0.96270500 -0.17622500

C -2.69017800 1.25405600 0.19748900

H -1.25079800 2.81418200 0.56698000

C -2.86017200 -0.10866000 -0.08800000

H -1.91751100 -2.02129600 -0.37504300

H -3.55164100 1.90861700 0.27070500

C 2.01646600 0.69824800 0.17223500

C 2.03797600 -0.55176800 0.84970200

C 2.99200900 1.08216000 -0.72245500

C 3.20803600 -1.35139500 0.66633700

H 1.51357100 -0.62445600 1.80203700

C 4.09108000 0.24214600 -0.92574000

H 2.88844100 2.02320900 -1.25289900

C 4.18670500 -0.97363100 -0.22975600

H 3.30175800 -2.27088700 1.23483200

H 4.86915400 0.53509000 -1.62251000

H 5.05518700 -1.60793400 -0.37842100

Cl -4.47965300 -0.72649400 -0.32325500

N 0.53620500 -1.38585100 0.00514100

**TS_B_**

E (M06-2X-SMD/6-31G(D)) = -1051.90831643 au

H (M06-2X-SMD/6-31G(D)) = -1051.725726 au

G (M06-2X-SMD/6-31G(D)) = -1051.773805 au

E (M06-2X-SMD/DEF2-TZVP//M06-2X-SMD/6-31G(D)) = -1052.16475212 au

O 0.90723300 1.54744900 0.06564600

C -0.33415400 0.99521100 0.26229200

C -0.39732800 -0.38128000 -0.06250500

C -1.44945100 1.79495600 0.47572500

C -1.68793600 -0.91358100 -0.30212700

C -2.70351200 1.22258800 0.33718800

H -1.33147000 2.84542100 0.72095700

C -2.79066800 -0.11857100 -0.07069900

H -1.78681600 -1.95175700 -0.59941200

H -3.60265700 1.80306900 0.50641600

C 1.90985000 0.67952400 0.02392100

C 1.76398400 -0.57930200 0.76047900

C 3.06154200 1.03150700 -0.73672600

C 2.99850600 -1.42219300 0.72346600

H 1.37370300 -0.43636200 1.78191800

C 4.12402500 0.18836600 -0.71499200

H 3.05420300 1.95568400 -1.30439500

C 4.08969800 -1.05815700 0.03120000

H 2.96573200 -2.35915400 1.27163100

H 5.01503600 0.43012100 -1.28605300

H 4.96736400 -1.69587000 0.00902300

Cl -4.38751800 -0.81461000 -0.28332100

N 0.73821800 -1.10811100 -0.16744600

**TS_C_**

E (M06-2X-SMD/6-31G(D)) = -1051.92672245 au

H (M06-2X-SMD/6-31G(D)) = -1051.744593 au

G (M06-2X-SMD/6-31G(D)) = -1051.792593 au

E (M06-2X-SMD/DEF2-TZVP//M06-2X-SMD/6-31G(D)) = -1052.18228261 au

O 0.87796300 1.75213000 0.25543800

C -0.39251000 1.24169500 0.17316900

C -0.34663200 -0.10486500 -0.17911400

C -1.57762500 1.90774000 0.40458200

C -1.49702700 -0.85463100 -0.31514900

C -2.75479400 1.16330500 0.26559400

H -1.60093400 2.95564300 0.68052800

C -2.70123000 -0.18246100 -0.08372000

H -1.46806200 -1.90397400 -0.58604000

H -3.71578100 1.63795300 0.43079900

C 1.73003600 0.71663400 -0.10375900

C 1.68301400 -0.81801000 0.91327500

C 2.99624400 0.98154600 -0.63736700

C 2.90420800 -1.49194000 0.89080700

H 1.06533100 -0.75406900 1.80506700

C 3.92060000 -0.03694800 -0.78752900

H 3.25088100 2.01454600 -0.85046200

C 3.86484700 -1.25436100 -0.08334800

H 3.14900000 -2.12209500 1.74231400

H 4.83463700 0.18566300 -1.33104700

H 4.73676800 -1.90038600 -0.12784100

Cl -4.19779300 -1.07554400 -0.24148700

N 1.01602300 -0.49318300 -0.34945700

**TS_I_**

E (M06-2X-SMD/6-31G(D)) = -2023.50436135 au

H (M06-2X-SMD/6-31G(D)) = -2023.137330 au

G (M06-2X-SMD/6-31G(D)) = -2023.216024 au

E (M06-2X-SMD/DEF2-TZVP//M06-2X-SMD/6-31G(D)) = -2024.08595663 au

O 1.63881800 -0.76900300 1.31862100

C 0.79572500 0.27192900 0.98547600

C 1.14451900 0.87451800 -0.21935500

C -0.28180700 0.69775000 1.73626900

C 0.40682800 1.94553000 -0.70678500

C -1.03979000 1.76767300 1.25758500

H -0.53149400 0.20370300 2.66941800

C -0.68273100 2.36579200 0.05448700

H 0.66539900 2.43480300 -1.63918700

H -1.89951200 2.12461200 1.81330500

C 2.82390100 -0.83586100 -0.17244000

C 3.23875600 1.26479700 -1.39341600

C 4.16469000 -0.75168300 0.49723400

C 4.15558900 1.93375100 -0.68803100

H 3.09591500 1.42092100 -2.45821600

C 4.37178900 0.50499900 1.29744900

H 4.28544800 -1.65160900 1.10237500

C 4.39326200 1.72241600 0.74166700

H 4.75869100 2.67254100 -1.21116800

H 4.55679300 0.39066700 2.36125300

H 4.62934900 2.59336300 1.34773300

Cl -1.65061100 3.69394600 -0.54448300

N 2.30937500 0.33733200 -0.79747300

H 4.89837400 -0.80841000 -0.32124800

O 2.53308500 -1.98962100 -0.66200300

H 1.59237200 -2.00555400 -1.11295600

C -4.10000500 0.37971300 0.12212100

C -3.29370200 0.40682900 -1.01776600

C -2.24528500 -0.49625800 -1.17901400

C -2.00862200 -1.44045400 -0.18703100

C -2.80645600 -1.49968200 0.95530100

C -3.84362300 -0.58789500 1.10298900

H -3.48166300 1.15037600 -1.78794500

H -1.61078100 -0.46352200 -2.05850100

H -2.60763400 -2.24091900 1.72417700

H -4.46598400 -0.62288500 1.99361900

C -5.19620300 1.39344300 0.31569200

H -6.06026700 0.95274800 0.82124700

H -4.84349600 2.22760200 0.93421600

H -5.52616900 1.80819500 -0.64047600

S -0.70437000 -2.63040100 -0.38410000

O 0.17163800 -2.05451500 -1.46134900

O -1.29454000 -3.92100100 -0.71885000

O 0.03402600 -2.65604500 0.92603100

H 1.06412700 -1.64098200 1.21228900

**MECP1**

E (M06-2X-SMD/6-31G(D)) = -1051.89870123 au

O 0.92881900 1.52675700 0.17281210

C -0.31085450 0.96224690 0.19810340

C -0.45719430 -0.45426160 0.07476240

C -1.40741870 1.81232930 0.23756660

C -1.78164280 -0.95370880 -0.10072180

C -2.68849710 1.29021300 0.11856280

H -1.24851240 2.88109180 0.33801420

C -2.85134760 -0.09351070 -0.06702580

H -1.90776090 -2.02415080 -0.22229570

H -3.55358870 1.94255000 0.14373200

C 1.99822540 0.69569820 0.07716270

C 1.81478340 -0.69657270 0.58953930

C 3.17557250 1.17970080 -0.42915030

C 2.99172360 -1.56473560 0.27116720

H 1.72511760 -0.62392070 1.69888450

C 4.27106810 0.31514580 -0.57084610

H 3.23186660 2.21735440 -0.74386530

C 4.14454930 -1.06552400 -0.24190370

H 2.87655840 -2.62113190 0.49300880

H 5.20096340 0.68967120 -0.98295520

H 4.98164470 -1.73050360 -0.43147400

Cl -4.47136090 -0.72827780 -0.25158800

N 0.56399690 -1.31488330 0.13990080

**I**

E (M06-2X-SMD/6-31G(D)) = -2023.52999177 au

H (M06-2X-SMD/6-31G(D)) = -2023.158539 au

G (M06-2X-SMD/6-31G(D)) = -2023.237620 au

E (M06-2X-SMD/DEF2-TZVP//M06-2X-SMD/6-31G(D)) = -2024.10906098 au

O 1.60684200 -0.89340800 1.14138200

C 0.81390500 0.21132100 0.93338100

C 1.20494900 0.86073500 -0.23597800

C -0.22004600 0.64655700 1.72731700

C 0.55708500 2.01391100 -0.64678500

C -0.89487600 1.80762800 1.32006300

H -0.50049500 0.11893000 2.63286500

C -0.49669500 2.45801900 0.16132300

H 0.84970600 2.54826100 -1.54364300

H -1.71948800 2.19434100 1.90809500

C 2.65920400 -0.92611300 0.11897100

C 3.24227300 0.84380200 -1.58013300

C 4.01432000 -0.69516600 0.77676100

C 4.16451300 1.63049400 -1.01215000

H 3.15451800 0.74036500 -2.65775000

C 4.22045800 0.71155900 1.27116600

H 4.11642600 -1.42678000 1.58155100

C 4.32415900 1.75914600 0.44124000

H 4.82576400 2.20220000 -1.66017500

H 4.34553800 0.86228500 2.33986800

H 4.56532500 2.74358100 0.83551000

Cl -1.36812000 3.89533700 -0.33575100

N 2.25906400 0.14948100 -0.80377900

H 4.76274400 -0.93173100 0.01096600

O 2.67203900 -2.15992500 -0.46185800

H 1.84277300 -2.25995100 -0.97460600

H 0.51234300 -2.22706300 1.10680900

C -3.90932800 0.68831100 -0.05024900

C -3.90673600 -0.33719700 0.90687900

C -3.00958600 -1.39177500 0.82411500

C -2.09268700 -1.40678500 -0.22776200

C -2.07141500 -0.40954100 -1.19519400

C -2.99027700 0.63224700 -1.09933300

H -4.61989000 -0.30465000 1.72635000

H -3.01373900 -2.18260600 1.56864000

H -1.34785200 -0.44402400 -2.00294400

H -2.98120600 1.42098600 -1.84660300

C -4.86735000 1.84128200 0.07731000

H -4.85234500 2.46952200 -0.81668000

H -5.89042000 1.48723700 0.23815100

H -4.60022000 2.46849600 0.93561500

S -0.96556200 -2.75746400 -0.37139300

O -0.26850100 -2.84388700 1.05986700

O 0.05406400 -2.38525600 -1.35455600

O -1.69033200 -3.99973600 -0.53123100

**TS_D_**

E (M06-2X-SMD/6-31G(D)) = -1947.09060439 au

H (M06-2X-SMD/6-31G(D)) = -1946.754208 au

G (M06-2X-SMD/6-31G(D)) = -1946.831999 au

E (M06-2X-SMD/DEF2-TZVP//M06-2X-SMD/6-31G(D)) = -1947.63398994 au

O -2.33203800 0.07022600 1.97988900

C -3.43373500 -0.47466300 1.35759100

C -3.45286300 -0.03549500 0.04145600

C -4.38897700 -1.31231300 1.89117600

C -4.45393600 -0.41684300 -0.83806800

C -5.40507600 -1.70997000 1.02192500

H -4.35436200 -1.64251100 2.92261500

C -5.42054800 -1.26654100 -0.30352300

H -4.49019200 -0.07770400 -1.86672700

H -6.19100900 -2.36737200 1.37555100

C -1.67565400 0.81287300 1.08287100

C -1.80320600 1.22653600 -1.35354900

C -0.45678700 1.45097500 1.38143200

C -1.00670000 2.28600400 -1.49853000

H -2.11066300 0.59922300 -2.18241600

C -0.28127900 2.82087800 0.81803000

H -0.21296900 1.35896600 2.43740800

C -0.52461500 3.18577100 -0.44716200

H -0.69007100 2.49916200 -2.51594300

H 0.10598400 3.56296100 1.51106500

H -0.31389100 4.20760600 -0.74900200

Cl -6.71618700 -1.80010200 -1.34497600

N -2.32162800 0.77462100 -0.10237900

C 6.03316000 -1.98987900 -0.08094700

C 5.46014400 -1.61498600 -1.29809300

C 4.42337700 -0.68568500 -1.34745100

C 3.95759200 -0.12776300 -0.16314700

C 4.51448400 -0.48094500 1.06495900

C 5.54705300 -1.40865600 1.09774500

H 5.83221500 -2.05263900 -2.22055100

H 3.98547100 -0.38659400 -2.29441400

H 4.14763800 -0.02216800 1.97805100

H 5.99033800 -1.68635100 2.05091000

C 7.15564900 -2.99192900 -0.02339800

H 6.87966000 -3.85220300 0.59560100

H 7.41305100 -3.35724600 -1.02068000

H 8.05304400 -2.54753100 0.42010400

S 2.61259100 1.03507000 -0.20341500

O 2.55845100 1.59179300 -1.55417800

O 2.79645400 1.97209300 0.90729400

O 1.39184200 0.12578400 0.07698700

H 0.53471000 0.69927500 0.69448300

**TS_H_**

E (M06-2X-SMD/6-31G(D)) = -2023.50613156 au

H (M06-2X-SMD/6-31G(D)) = -2023.137589 au

G (M06-2X-SMD/6-31G(D)) = -2023.217878 au

E (M06-2X-SMD/DEF2-TZVP//M06-2X-SMD/6-31G(D)) = -2024.09040771 au

O -2.27537300 -1.53135500 1.58313400

C -3.21762400 -0.80709300 0.87666400

C -2.56841600 0.17804000 0.13924000

C -4.58543000 -0.96252700 0.88163600

C -3.27092600 1.08197400 -0.63841100

C -5.31343000 -0.06744600 0.09260300

H -5.07314300 -1.73368900 1.46651300

C -4.65757800 0.92162600 -0.63995900

H -2.78188800 1.86580500 -1.20513300

H -6.39397300 -0.14023700 0.05053100

C -1.05943500 -1.14249000 1.12505100

C -0.18112300 0.95400500 0.10936600

C 0.14071200 -1.35296700 1.99275700

C 0.66803800 1.41974500 1.03348500

H -0.16586200 1.27938600 -0.92492600

C 0.36249900 -0.17425300 2.90889100

H -0.00520300 -2.28491000 2.54099900

C 0.65539900 1.04972300 2.44877000

H 1.39958100 2.15298100 0.70254400

H 0.33889500 -0.35930800 3.97849300

H 0.90944500 1.83618000 3.15585000

Cl -5.61341400 2.01762300 -1.60865800

N -1.20683300 0.03011200 0.42440400

C 4.20830400 2.19924200 0.13400000

C 4.17083900 1.09396800 0.98493000

C 3.61456400 -0.11626100 0.56816000

C 3.08974000 -0.21975700 -0.71297700

C 3.11601900 0.87114800 -1.58296800

C 3.67002700 2.07021700 -1.15545700

H 4.58285800 1.17732500 1.98739900

H 3.58662800 -0.97578600 1.23070000

H 2.70667500 0.77904300 -2.58518400

H 3.69389300 2.92300100 -1.82993200

C 4.81374600 3.50602700 0.57499300

H 5.13292500 3.46268200 1.61937400

H 5.68685000 3.75703100 -0.03701000

H 4.09697700 4.32721200 0.46880800

S 2.36018100 -1.75397100 -1.25794400

O 2.44481100 -2.68297100 -0.12024300

O 3.06012000 -2.14795800 -2.48173700

O 0.91237600 -1.38666900 -1.53907400

H 1.00596800 -1.49119400 1.33099300

O -0.76244600 -2.43065200 -0.06378300

H -0.00589100 -2.05332400 -0.72288800

H -1.57321400 -2.54968900 -0.59471600

**H**

E (M06-2X-SMD/6-31G(D)) = -2023.52408880 au

H (M06-2X-SMD/6-31G(D)) = -2023.152019 au

G (M06-2X-SMD/6-31G(D)) = -2023.235830 au

E (M06-2X-SMD/DEF2-TZVP//M06-2X-SMD/6-31G(D)) = -2024.11194631 au

O -2.27819600 -1.48082600 1.76647500

C -3.15998800 -0.72422800 1.03116800

C -2.45439400 0.30026900 0.41859300

C -4.52599500 -0.88580300 0.89823500

C -3.07629400 1.25254200 -0.37592700

C -5.16731600 0.05035900 0.09518200

H -5.06227600 -1.69302200 1.38265600

C -4.44854600 1.08760800 -0.51841100

H -2.53655000 2.06285900 -0.85199400

H -6.23779500 -0.01903500 -0.06213000

C -1.07758500 -0.98330600 1.55811900

C -0.02931000 0.96155400 0.50868300

C 0.15876300 -1.56184700 2.11277900

C 0.97324200 1.20242900 1.36539300

H -0.09997300 1.41295000 -0.47203700

C 0.73730700 -0.56360600 3.09207000

H -0.07760400 -2.51727900 2.58163300

C 1.12743100 0.66077400 2.70989300

H 1.72276400 1.91192400 1.02330600

H 0.84269600 -0.87624200 4.12614400

H 1.61412300 1.30949500 3.43398300

Cl -5.32151500 2.23136700 -1.50266900

N -1.12241600 0.11773800 0.81953200

C 4.53356500 1.71459900 0.22528100

C 4.38522400 0.45127500 0.80126200

C 3.55388800 -0.50932000 0.22478600

C 2.85328600 -0.20307800 -0.93502800

C 2.98515600 1.05324100 -1.52673000

C 3.81953500 2.00135200 -0.94709100

H 4.92697200 0.21392300 1.71363500

H 3.43452900 -1.48826500 0.67875600

H 2.42549900 1.28873000 -2.42784400

H 3.92190000 2.98184300 -1.40637100

C 5.45276100 2.74366000 0.82978000

H 5.71137700 2.48802200 1.86076800

H 6.38580000 2.81243800 0.25888400

H 4.99462300 3.73749700 0.82403700

S 1.76843000 -1.40857900 -1.68956500

O 1.62832800 -2.48858200 -0.67391100

O 2.42028300 -1.84034400 -2.93333700

O 0.48937400 -0.67681900 -1.91174100

H 0.83975600 -1.73670100 1.26282800

O -1.18415900 -2.47563800 -0.59721900

H -0.89909700 -1.77799000 -1.21960900

H -0.32836800 -2.92446800 -0.47531900

**F**

E (M06-2X-SMD/6-31G(D)) = -1128.36968693 au

H (M06-2X-SMD/6-31G(D)) = -1128.156253 au

G (M06-2X-SMD/6-31G(D)) = -1128.209968 au

E (M06-2X-SMD/DEF2-TZVP//M06-2X-SMD/6-31G(D)) = -1128.66582156 au

O 1.01567100 -1.71916800 0.31919100

C -0.27077300 -1.26984200 0.27478900

C -0.33285600 -0.04219600 -0.39401500

C -1.38444400 -1.86947200 0.81420200

C -1.52824600 0.64238300 -0.51896900

C -2.61144500 -1.19623100 0.68592300

H -1.31624600 -2.81847200 1.33429000

C -2.65819700 0.02805800 0.03780500

H -1.59163300 1.60551300 -1.01283200

H -3.51517200 -1.62848100 1.09997000

C 1.84662400 -0.84157600 -0.45962200

C 1.38404800 1.60919200 -0.99318800

C 3.06680100 -0.41431000 0.33865400

C 1.98231100 2.30679200 -0.01874200

H 1.14249700 2.06282800 -1.95127000

C 2.74256700 0.50400400 1.48551300

H 3.56674100 -1.32425300 0.67841900

C 2.29457800 1.75296400 1.30280600

H 2.22577000 3.34894600 -0.21390300

H 2.93694500 0.14797400 2.49340500

H 2.16251400 2.41192100 2.15817100

Cl -4.19686900 0.86035700 -0.09652400

N 0.95269600 0.26544300 -0.82030700

H 3.73613200 0.09142000 -0.36766600

O 2.30990200 -1.51828200 -1.57418700

H 1.53996500 -1.74055600 -2.12649600

**TS_F_**

E (M06-2X-SMD/6-31G(D)) = -1128.32782770 au

H (M06-2X-SMD/6-31G(D)) = -1128.115942 au

G (M06-2X-SMD/6-31G(D)) = -1128.168515 au

E (M06-2X-SMD/DEF2-TZVP//M06-2X-SMD/6-31G(D)) = -1128.62669298 au

O -0.92939700 -1.60362500 -0.74869900

C 0.23918000 -1.10688200 -0.61965700

C 0.41333100 0.01217100 0.26880100

C 1.43413300 -1.55917200 -1.24094300

C 1.62655100 0.60905400 0.55954000

C 2.65951800 -0.97447400 -0.95619300

H 1.37327100 -2.38700900 -1.94000300

C 2.75831900 0.09350400 -0.06107700

H 1.68715600 1.45141600 1.24215500

H 3.55922400 -1.35338800 -1.43222800

C -1.72844400 -0.50108200 0.95125600

C -1.27018700 1.75660500 0.12646100

C -3.20508500 -0.31851700 0.83522100

C -2.28424100 2.00320800 -0.71261200

H -0.58272900 2.54573500 0.41250100

C -3.63731700 -0.05012800 -0.58165700

H -3.68379300 -1.20858300 1.24298700

C -3.23397200 1.03338800 -1.24954100

H -2.36604000 3.03061300 -1.05798800

H -4.30416500 -0.77009900 -1.04527400

H -3.61575000 1.22588700 -2.24887500

Cl 4.32891100 0.79963100 0.28232300

N -0.87515200 0.50048800 0.68127900

H -3.46532700 0.53484900 1.47985500

O -1.36004800 -1.47449100 1.75620400

H -0.38242600 -1.52767100 1.80079000

**E**

E (M06-2X-SMD/6-31G(D)) = -1357.50848147 au

H (M06-2X-SMD/6-31G(D)) = -1357.215225 au

G (M06-2X-SMD/6-31G(D)) = -1357.285591 au

E (M06-2X-SMD/DEF2-TZVP//M06-2X-SMD/6-31G(D)) = -1357.95839745 au

O 0.16885200 -2.02013400 -0.95192300

C -1.05081700 -1.45373700 -0.66725000

C -0.85304300 -0.41215800 0.22404800

C -2.29807100 -1.82708800 -1.13361500

C -1.90793200 0.34010800 0.72009100

C -3.36809300 -1.08194800 -0.65343100

H -2.43228900 -2.64514500 -1.83115400

C -3.16079700 -0.02810800 0.25028500

H -1.76070800 1.16801000 1.40199600

H -4.37617600 -1.31180500 -0.97918100

C 1.08115400 -1.31670300 -0.31129800

C 1.22060700 0.50864500 1.31034200

C 2.52315000 -1.59226800 -0.39177000

C 2.30235400 0.14806500 2.01316500

H 0.77941700 1.49632700 1.34233200

C 2.99722500 -1.99898600 0.98630900

H 2.68781200 -2.37291100 -1.13478100

C 2.92130400 -1.17313800 2.03925500

H 2.71712500 0.91234800 2.66415500

H 3.42917500 -2.98888400 1.10063900

H 3.35523100 -1.48284700 2.98693700

Cl -4.55213300 0.86852600 0.80700800

N 0.52803500 -0.38531500 0.45742200

O 0.93966600 0.42020200 -2.26124700

H 3.00447000 -0.63815200 -0.71969000

H 1.86252400 0.71241500 -1.98197100

O 2.31409500 3.04356400 -0.02040800

H 2.58739800 3.83494200 -0.50668600

H 0.38551100 1.09718100 -1.82668300

O 3.24804200 1.13043800 -1.22649300

H 3.87204600 1.43189600 -1.90162300

H 2.79529700 2.19565000 -0.54875400

O -0.14841900 2.41689200 -0.54326700

H 0.79684500 2.74474100 -0.37778200

H -0.63380600 3.17469700 -0.89893600

**A^OSS^**

E (M06-2X-SMD/6-31G(D)) = -1051.88425362 au

H (M06-2X-SMD/6-31G(D)) = -1051.702696 au

G (M06-2X-SMD/6-31G(D)) = -1051.756699 au

E (M06-2X-SMD/DEF2-TZVP//M06-2X-SMD/6-31G(D)) = -1052.1409324 au

O 0.96359100 1.24027400 -0.00147000

C -0.23217000 0.61918700 -0.00521900

C -1.37089100 1.52926700 0.03038300

C -0.42376200 -0.75108000 -0.04319700

C -2.69520400 0.94460200 0.02456800

C -1.71443000 -1.27793400 -0.04682300

H 0.42913000 -1.42000200 -0.07055800

C -2.82931500 -0.41335100 -0.01260200

H -3.54956100 1.61112100 0.05038300

H -1.86281000 -2.35126400 -0.07705200

C 2.11528500 0.45803500 0.00200700

C 2.64079600 0.03697500 1.21721900

C 2.72985700 0.16607000 -1.20857800

C 3.82297800 -0.69951400 1.21399300

H 2.12924700 0.29016900 2.14076500

C 3.91273600 -0.57002200 -1.19653700

H 2.28623400 0.51744200 -2.13481300

C 4.45840900 -1.00318200 0.01070000

H 4.24815800 -1.03317300 2.15531500

H 4.40767700 -0.80289900 -2.13414300

H 5.38059800 -1.57548300 0.01425800

Cl -4.42723100 -1.12114700 -0.01880100

N -1.20003100 2.80958200 0.06739100

**A^CSS^**

E (M06-2X-SMD/6-31G(D)) = -1051.852248au

H (M06-2X-SMD/6-31G(D)) = -1051.669800au

G (M06-2X-SMD/6-31G(D)) = -1051.723949au

E (M06-2X-SMD/DEF2-TZVP//M06-2X-SMD/6-31G(D)) = -1052.121071au

O 0.95832000 1.23381400 0.03872300

C -0.22762100 0.65735500 0.01342000

C -1.38228600 1.57443300 0.04103400

C -0.40177900 -0.73657100 -0.03681500

C -2.69639600 0.93424000 0.00838800

C -1.68207000 -1.24761800 -0.06265600

H 0.44566500 -1.41151800 -0.05563800

C -2.83247600 -0.41764700 -0.04035800

H -3.55201000 1.60213800 0.02702500

H -1.81547700 -2.32520600 -0.10207700

C 2.11329000 0.44548900 0.02468400

C 2.66075100 0.04387200 1.23506800

C 2.69767600 0.14119900 -1.19669500

C 3.84169800 -0.69491700 1.21477400

H 2.16985900 0.31111800 2.16547900

C 3.87878000 -0.59778000 -1.19940300

H 2.23528900 0.48220000 -2.11744600

C 4.44948000 -1.01567700 0.00183200

H 4.28655000 -1.01696100 2.15072900

H 4.35267200 -0.84402600 -2.14412200

H 5.37021300 -1.58983700 -0.00711700

Cl -4.40555900 -1.17883500 -0.07601800

N -1.24527900 2.85440800 0.09437700

**TS_A_^OSS^**

E (M06-2X-SMD/6-31G(D)) = -1051.86549766 au

H (M06-2X-SMD/6-31G(D)) = -1051.685641 au

G (M06-2X-SMD/6-31G(D)) = -1051.735823 au

E (M06-2X-SMD/DEF2-TZVP//M06-2X-SMD/6-31G(D)) = -1052.12127956 au

O 0.90172200 1.48793400 0.16440300

C -0.30521100 0.90001900 0.27505900

C -0.45893700 -0.51536500 -0.02093700

C -1.40475900 1.71840200 0.47249200

C -1.81059300 -0.95011900 -0.31034900

C -2.68335900 1.22018000 0.33171400

H -1.23990700 2.76097700 0.71163000

C -2.85868300 -0.11639300 -0.08083500

H -1.95260200 -1.97618800 -0.61940900

H -3.54375200 1.85296400 0.49651000

C 2.00675300 0.68889100 0.09506700

C 2.07600200 -0.45753800 0.90116200

C 2.99886900 1.02220700 -0.81574200

C 3.23584900 -1.26121900 0.78805000

H 1.47336600 -0.50283900 1.79964100

C 4.10324500 0.19934400 -0.92505400

H 2.88143600 1.90538000 -1.43001300

C 4.21328800 -0.94916000 -0.12408700

H 3.33593600 -2.12585700 1.43080200

H 4.88322200 0.43972100 -1.63528100

H 5.08870200 -1.57942000 -0.21358700

Cl -4.48117900 -0.69557900 -0.31450200

N 0.48072500 -1.40698100 -0.00403100

**TS^water^_D_**

E (M06-2X-SMD/6-31G(D)) = -1357.50543676 au

H (M06-2X-SMD/6-31G(D)) = -1357.214938 au

G (M06-2X-SMD/6-31G(D)) = -1357.284603 au

E (M06-2X-SMD/DEF2-TZVP//M06-2X-SMD/6-31G(D)) = -1357.95384618 au

O 0.16914000 -2.05588700 -0.84580300

C -1.05259500 -1.48245400 -0.59137400

C -0.86780500 -0.43631200 0.29879100

C -2.28975300 -1.84827000 -1.08318700

C -1.92760100 0.32905100 0.76072600

C -3.36826200 -1.09172700 -0.63387500

H -2.41387000 -2.66990800 -1.77847200

C -3.17402900 -0.03313100 0.26318300

H -1.79423800 1.15900900 1.44366500

H -4.36976600 -1.31871400 -0.98099700

C 1.08200100 -1.35666000 -0.18940800

C 1.17651500 0.57183300 1.33814100

C 2.50106000 -1.59061900 -0.31439800

C 2.31724400 0.34051600 1.99522500

H 0.66151300 1.52415500 1.34597200

C 3.14405100 -1.78912100 1.02829300

H 2.66495400 -2.43021100 -0.98939800

C 3.07150500 -0.91075000 2.03781700

H 2.69897000 1.17580500 2.57612600

H 3.71860800 -2.70229400 1.16476400

H 3.62050300 -1.11184400 2.95413700

Cl -4.57077600 0.87964700 0.78228500

N 0.50722900 -0.40364900 0.55075300

O 0.64278600 0.39575600 -2.27927800

H 2.91692300 -0.53026300 -0.83921300

H 1.60978500 0.56072700 -2.12971500

O 2.51782000 2.94290200 -0.27469900

H 2.81761800 3.62326500 -0.89561500

H 0.22741800 1.12324400 -1.77800700

O 3.14459200 0.74364200 -1.40097100

H 3.91743300 0.74775000 -1.98340300

H 2.84850700 2.05873700 -0.68452600

O -0.09820600 2.52361500 -0.52889800

H 0.86912600 2.76359600 -0.45127400

H -0.52392600 3.28412900 -0.95058700

**5a**

E (M06-2X-SMD/6-31G(D)) = -1128.38454167 au

H (M06-2X-SMD/6-31G(D)) = -1128.170965 au

G (M06-2X-SMD/6-31G(D)) = -1128.227836 au

E (M06-2X-SMD/DEF2-TZVP//M06-2X-SMD/6-31G(D)) = -1128.68448218 au

O -0.47565700 2.30666500 -0.43569600

C 0.61001100 1.52013300 -0.26452500

C 0.38842000 0.17639300 0.06208700

C 1.91962100 1.98345900 -0.39244500

C 1.45724800 -0.68893100 0.25502300

C 2.99427600 1.12237900 -0.19794400

H 2.10070500 3.02445000 -0.64551000

C 2.75462400 -0.20742600 0.12522000

H 1.26389200 -1.72795600 0.50238400

H 4.01077000 1.48754400 -0.29832300

C -1.55351800 -0.84595500 -0.92524900

C -1.54375500 -0.11352600 1.46703600

C -2.91478000 -1.46590500 -0.67940800

C -2.83355000 0.16400700 1.73948600

H -0.81665900 -0.14910600 2.27347300

C -3.92206500 -0.38709200 -0.39151700

H -3.17543000 -2.03760500 -1.56985000

C -3.88930500 0.31238200 0.75237900

H -3.07460600 0.34094900 2.78389900

H -4.69129200 -0.19345600 -1.13369500

H -4.68052200 1.02565500 0.97256600

Cl 4.10394800 -1.29347900 0.37668200

N -0.95669000 -0.29164200 0.20415500

H -2.83930600 -2.14501800 0.18027100

O -0.99731500 -0.83392300 -2.00505900

H -0.18741300 3.20374000 -0.67272500

**TS_A′_**

E (M06-2X-SMD/6-31G(D)) = -1051.85702356 au

H (M06-2X-SMD/6-31G(D)) = -1051.676199 au

G (M06-2X-SMD/6-31G(D)) = -1051.727736 au

E (M06-2X-SMD/DEF2-TZVP//M06-2X-SMD/6-31G(D)) = -1052.11368549 au

C -0.25462500 0.86475500 0.12831800

C -1.30862200 1.65051300 0.53139300

C -2.93071400 -0.06817000 -0.05151500

C -1.93487000 -0.81929100 -0.65832700

C -0.54706500 -0.51853400 -0.30686100

H -1.10656300 2.65406500 0.89018000

H -2.13181300 -1.71018800 -1.23808100

N -0.00549100 -1.64826700 -0.13418300

O 0.95393300 1.42235200 -0.02521900

C 2.07947000 0.61651100 0.01753100

C 2.21226200 -0.38848000 0.96991400

C 3.08997800 0.90636900 -0.89514700

C 3.38962800 -1.13767300 0.98735400

H 1.42532700 -0.55454600 1.69756700

C 4.25729300 0.15421900 -0.85998800

H 2.94534900 1.70581600 -1.61451600

C 4.40674200 -0.87126300 0.07719000

H 3.50557700 -1.92730000 1.72278100

H 5.05210100 0.36608400 -1.56807300

H 5.32044300 -1.45660600 0.09800900

C -2.62598100 1.15824700 0.52734900

H -3.41953500 1.77131200 0.94205800

Cl -4.59911100 -0.58663600 -0.15248000

**B′**

E (M06-2X-SMD/6-31G(D)) = -1051.88450322 au

H (M06-2X-SMD/6-31G(D)) = -1051.701446 au

G (M06-2X-SMD/6-31G(D)) = -1051.754552 au

E (M06-2X-SMD/DEF2-TZVP//M06-2X-SMD/6-31G(D)) = -1052.14079801 au

O 1.08943900 1.29353300 -0.30297400

C -0.10354500 0.71633200 -0.05129700

C -1.32261100 1.42950300 -0.35640400

C -0.38172500 -0.48313500 0.52231400

C -2.64516500 0.87206500 -0.66181900

C -1.74566400 -0.96555500 0.67352300

H 0.42376900 -1.09745900 0.91222000

C -2.81809500 -0.36408900 0.10022800

H -1.88135900 -1.88714000 1.23033400

C 2.22244200 0.49348300 -0.17257800

C 3.08627000 0.75140200 0.88258000

C 2.48633500 -0.49283800 -1.11682400

C 4.25543700 0.00001900 0.99057900

H 2.84214800 1.53181300 1.59600600

C 3.65494900 -1.23906900 -0.99337500

H 1.78743300 -0.66377000 -1.93010100

C 4.53947100 -0.99421900 0.05723800

H 4.94256200 0.19441900 1.80808200

H 3.87650200 -2.01002600 -1.72468900

H 5.45073500 -1.57720200 0.14515600

Cl -4.38983200 -1.12763300 0.11817500

N -2.25093200 2.13946700 0.10713500

H -3.14221900 1.01119300 -1.61711800

**TS_B′_**

E (M06-2X-SMD/6-31G(D)) = -1051.86562305 au

H (M06-2X-SMD/6-31G(D)) = -1051.684751 au

G (M06-2X-SMD/6-31G(D)) = -1051.737289 au

E (M06-2X-SMD/DEF2-TZVP//M06-2X-SMD/6-31G(D)) = -1052.12333395 au

O -1.21256000 1.61783600 -0.10490200

C 0.12182900 1.37339600 -0.26922100

C 1.08071900 1.64975600 0.65804000

C 0.69132000 0.76061800 -1.40485900

C 2.41815100 0.47186900 1.07011100

C 1.90319800 0.08712400 -1.34826500

H 0.12208300 0.76152800 -2.33204700

C 2.56237900 -0.19918700 -0.12990500

H 2.26489400 -0.40073300 -2.24660800

C -2.04777000 0.52020200 0.00684400

C -3.39925200 0.76059200 -0.22908000

C -1.59335300 -0.74492600 0.36979200

C -4.30781000 -0.28426100 -0.10138200

H -3.71695100 1.76001800 -0.50859200

C -2.51749900 -1.78207300 0.48912800

H -0.53904900 -0.91974100 0.56192400

C -3.87187600 -1.56035000 0.25517800

H -5.36165900 -0.09882400 -0.28569500

H -2.16778100 -2.77023900 0.77239900

H -4.58328600 -2.37425200 0.35075300

Cl 3.48862400 -1.68990500 -0.06814100

N 2.21973600 1.92968900 1.06484300

H 2.59725300 0.00356100 2.03330800

**C′**

E (M06-2X-SMD/6-31G(D)) = -1051.87651112 au

H (M06-2X-SMD/6-31G(D)) = -1051.693735 au

G (M06-2X-SMD/6-31G(D)) = -1051.746819 au

E (M06-2X-SMD/DEF2-TZVP//M06-2X-SMD/6-31G(D)) = -1052.13506702 au

O -1.23733500 -1.71704900 0.07642500

C 0.11615800 -1.57037000 0.23108700

C 1.04461900 -1.85972900 -0.65795400

C 0.72678500 -1.01316100 1.44281300

C 2.61085100 -0.43159700 -1.06946600

C 1.76807800 -0.15563300 1.32197700

H 0.34472400 -1.28060800 2.42616700

C 2.40786900 0.26932400 0.07578700

H 2.18482100 0.28488700 2.22414900

C -1.98517400 -0.56036000 -0.03064800

C -3.35140200 -0.69738600 0.20789600

C -1.43772200 0.67022200 -0.38408800

C -4.17799600 0.41396700 0.09039400

H -3.74358600 -1.67200200 0.48046000

C -2.28035400 1.77620000 -0.49180400

H -0.37440000 0.76534600 -0.58316400

C -3.64685900 1.65677300 -0.25607100

H -5.24270800 0.30765600 0.27511600

H -1.85616200 2.73729600 -0.76649600

H -4.29430400 2.52342100 -0.34215000

Cl 3.15135700 1.85766000 0.16090700

N 2.19223800 -1.78077500 -1.14930500

H 3.19242800 -0.03389600 -1.89529400

**J**

E (M06-2X-SMD/6-31G(D)) = -1281.10099405 au

H (M06-2X-SMD/6-31G(D)) = -1280.833784 au

G (M06-2X-SMD/6-31G(D)) = -1280.902199 au

E (M06-2X-SMD/DEF2-TZVP//M06-2X-SMD/6-31G(D)) = -1281.50085509 au

O -0.91655700 -2.11087500 0.47806100

C 0.33448400 -1.51720900 0.43351000

C 0.43841800 -0.31285100 -0.31496100

C 1.42338300 -2.25559800 0.87803200

C 1.76501100 0.05882000 -0.68624900

C 2.70282800 -1.81299100 0.59002700

H 1.25611300 -3.17474700 1.43102600

C 2.83605300 -0.66591700 -0.21156700

H 1.90818300 0.93977300 -1.30404400

H 3.57682500 -2.34861400 0.93921000

C -1.95917800 -1.38917900 0.18177900

C -1.78818800 0.08976200 0.18450600

C -3.16738800 -2.04185300 -0.12421100

C -2.99621600 0.80975900 -0.31373100

H -1.60631900 0.39906800 1.23721400

C -4.23155500 -1.26330100 -0.48762000

H -3.21836000 -3.12458400 -0.09989700

C -4.14706500 0.17321400 -0.59952000

H -2.89130900 1.88522800 -0.40983700

H -5.16973700 -1.74671400 -0.74485400

H -5.01904800 0.71828300 -0.94351300

Cl 4.45384100 -0.13174100 -0.62287700

N -0.63419800 0.40446000 -0.66252200

O -1.41434900 2.92345000 1.41964100

H -0.49907400 2.68937600 1.66838800

H -1.26597400 3.31750900 0.54385900

O 1.37451400 2.54629600 1.28094600

H 1.65604900 1.62393400 1.18353500

H 1.11553900 2.80932600 0.37272900

O -0.06242000 3.00474400 -1.03393000

H 0.11051700 3.23685400 -1.95820300

H -0.34233900 2.03883200 -1.02675200

**TS_J_**

E (M06-2X-SMD/6-31G(D)) = -1281.09673268 au

H (M06-2X-SMD/6-31G(D)) = -1280.833224 au

G (M06-2X-SMD/6-31G(D)) = -1280.900246 au

E (M06-2X-SMD/DEF2-TZVP//M06-2X-SMD/6-31G(D)) = -1281.49712178 au

O -1.02395100 -1.82455700 0.29609100

C 0.28263400 -1.31902200 0.13932400

C 0.46511000 -0.09320500 -0.55545100

C 1.30636300 -2.12173300 0.58706600

C 1.82210700 0.23795300 -0.81315200

C 2.63434700 -1.75054100 0.36157900

H 1.06493300 -3.04400500 1.10703200

C 2.84808600 -0.57664500 -0.35507600

H 2.03864900 1.14639700 -1.36656500

H 3.45898700 -2.35814400 0.71204900

C -2.03458700 -1.07977000 -0.05500300

C -1.81119700 0.33601400 -0.37172700

C -3.32571600 -1.62569600 0.00093500

C -2.98204100 1.04901200 -0.89526900

H -1.83427200 0.66647000 0.74175400

C -4.38242000 -0.83590900 -0.38981000

H -3.45889300 -2.65845400 0.30293400

C -4.22564400 0.50794000 -0.84281000

H -2.80579400 2.05923900 -1.25499900

H -5.38184800 -1.26135000 -0.36629500

H -5.09726400 1.06546600 -1.16541300

Cl 4.50241500 -0.08879900 -0.69025300

N -0.54476400 0.69894400 -0.95803100

O -1.01551300 0.63183000 2.40377700

H -0.16765700 1.11348100 2.21799300

H -1.48053300 1.19618100 3.03955000

O 1.21839600 2.01801600 1.77821900

H 1.77987200 1.36274200 1.33347300

H 0.87205700 2.57138200 1.03174500

O 0.09294000 3.23353000 -0.35701100

H 0.70963200 3.58852800 -1.01431700

H -0.18280900 2.32809000 -0.71066200

**K**

E (M06-2X-SMD/6-31G(D)) = -1052.01343935 au

H (M06-2X-SMD/6-31G(D)) = -1051.828561 au

G (M06-2X-SMD/6-31G(D)) = -1051.878268 au

E (M06-2X-SMD/DEF2-TZVP//M06-2X-SMD/6-31G(D)) = -1052.27182731 au

O -0.94603800 1.47952500 0.32747300

C 0.31417400 0.94802800 0.17097900

C 0.52232800 -0.43988400 0.22239800

C 1.37438300 1.81798700 -0.00106900

C 1.81739800 -0.93451700 0.09757800

C 2.67817300 1.32932900 -0.11181600

H 1.17422200 2.88376300 -0.03939100

C 2.87442600 -0.04133300 -0.06062800

H 1.99372000 -2.00481000 0.13268600

H 3.51467500 2.00703000 -0.23655000

C -2.01433400 0.63345000 0.10379100

C -1.85078800 -0.75747200 0.15360200

C -3.25485700 1.19724200 -0.13525400

C -2.96169900 -1.57282400 -0.04249300

C -4.36736300 0.37271500 -0.31818100

H -3.33672200 2.27908000 -0.16764000

C -4.21693900 -1.00852300 -0.27198900

H -2.83435000 -2.65128000 -0.00675200

H -5.34118600 0.81633600 -0.49662600

H -5.07407800 -1.65850600 -0.41570000

Cl 4.49778900 -0.68359200 -0.20057300

N -0.57717200 -1.27027500 0.41870000

H -0.43960300 -2.26001200 0.24753400

**L**

E (M06-2X-SMD/6-31G(D)) = -517.174896986 au

H (M06-2X-SMD/6-31G(D)) = -516.988426 au

G (M06-2X-SMD/6-31G(D)) = -517.032424 au

E (M06-2X-SMD/DEF2-TZVP//M06-2X-SMD/6-31G(D)) = -517.363628078 au

C -0.69186200 0.50682600 0.10579500

C -1.07076300 -0.90808500 0.04954600

C -1.68874900 1.53090800 0.00831500

C -2.46887800 -1.24533500 -0.08553800

C -2.98813000 1.15870000 -0.13553400

H -1.40228100 2.57810800 0.04649500

C -3.37203100 -0.23662900 -0.18183500

H -2.76383900 -2.28955000 -0.11667700

H -3.76727200 1.91025900 -0.21577800

C 0.67815100 0.51764400 0.22284400

C 1.08787000 -0.91400300 0.35314500

C 1.68497900 1.53456900 0.16269100

C 2.43589400 -1.23388600 -0.19957500

H 1.25250200 -1.03747800 1.45217700

C 2.96422300 1.15429500 -0.09802600

H 1.41448000 2.58419800 0.23070500

C 3.32970100 -0.23479100 -0.33365500

H 2.69598600 -2.27457500 -0.36887300

H 3.73983000 1.90870500 -0.19369200

H 4.34725000 -0.45027200 -0.64508100

N -0.07028100 -1.74868900 0.12810200

H -4.42712000 -0.46985300 -0.29503200

**TS_L_**

E (M06-2X-SMD/6-31G(D)) = -517.096510774 au

H (M06-2X-SMD/6-31G(D)) = -516.912531 au

G (M06-2X-SMD/6-31G(D)) = -516.956379 au

E (M06-2X-SMD/DEF2-TZVP//M06-2X-SMD/6-31G(D)) = -517.288243631 au

C 0.81374800 0.49016300 -0.29073200

C 0.98600200 -0.82498000 0.18073900

C 1.84305700 1.39033900 -0.44702500

C 2.27322800 -1.27625100 0.47394800

C 3.14323500 0.92699400 -0.17478100

H 1.66582900 2.41549000 -0.76185000

C 3.33319000 -0.37572700 0.28528500

H 2.44638700 -2.27540300 0.86242400

H 3.99378700 1.58786700 -0.30350400

C -0.66509400 0.50833700 -0.30443800

C -1.15440900 -0.81288000 -0.71969800

C -1.48791600 1.44751800 0.32885100

C -2.58197700 -1.05547000 -0.45127400

H -0.85265700 -1.13190500 -1.72594500

C -2.80796400 1.12829000 0.55478900

H -1.06601400 2.38377300 0.68006100

C -3.35614100 -0.13690800 0.17650500

H -3.00671400 -1.99599100 -0.79080200

H -3.44886400 1.84170400 1.06546800

H -4.40317100 -0.33650900 0.37764800

N -0.24355800 -1.46252100 0.34404000

H 4.34256600 -0.70792700 0.51519700

**M**

E (M06-2X-SMD/6-31G(D)) = -517.126196165 au

H (M06-2X-SMD/6-31G(D)) = -516.939660 au

G (M06-2X-SMD/6-31G(D)) = -516.983497 au

E (M06-2X-SMD/DEF2-TZVP//M06-2X-SMD/6-31G(D)) = -517.314278355 au

C -0.86715900 0.60685900 0.16595500

C -1.01669200 -0.66348800 -0.37720800

C -1.94135900 1.38272000 0.54254500

C -2.23215300 -1.28239300 -0.55925700

C -3.20171800 0.78117500 0.35776200

H -1.84992900 2.38838800 0.94067500

C -3.33987600 -0.50381600 -0.16921000

H -2.34912400 -2.27187800 -0.98881500

H -4.09566500 1.33489500 0.62851700

C 0.62136800 0.45069200 0.03415700

C 1.05805800 -0.86071200 0.69261900

C 1.63464800 1.37689500 -0.45339800

C 2.50961200 -1.09816600 0.70244700

H 0.47480800 -1.24491800 1.52638800

C 2.92515300 0.99101300 -0.51248100

H 1.32021800 2.37095700 -0.75595900

C 3.36106900 -0.26953300 0.06517500

H 2.88055200 -1.94189100 1.27673900

H 3.67832400 1.66445000 -0.90997000

H 4.42602000 -0.48332600 0.07578900

N 0.39814900 -0.90999700 -0.63378300

H -4.33795300 -0.91417900 -0.29151600

**N**

E (M06-2X-SMD/6-31G(D)) = -746.365233246 au

H (M06-2X-SMD/6-31G(D)) = -746.094909 au

G (M06-2X-SMD/6-31G(D)) = -746.157912 au

E (M06-2X-SMD/DEF2-TZVP//M06-2X-SMD/6-31G(D)) = -746.694179497 au

C -1.50487900 0.71776200 0.12581300

C -1.12461000 -0.57982600 -0.40884700

C -2.87178100 1.01020000 0.41669700

C -2.13945000 -1.57772400 -0.60796900

C -3.80561700 0.04442200 0.18999100

H -3.15006800 1.98389600 0.80952800

C -3.42945400 -1.24951700 -0.32171900

H -1.86493600 -2.55760600 -0.98703700

H -4.85329800 0.23478800 0.39909500

C -0.32998600 1.44528000 0.21665000

C 0.75942200 0.51827800 -0.16832200

C -0.02638400 2.81104400 0.49601700

C 1.96223900 1.14704200 -0.77145300

H 1.22580900 0.17786200 0.80652700

C 1.19481400 3.28681300 0.12133300

H -0.78273600 3.47102000 0.90967200

C 2.17837700 2.46111400 -0.54997000

H 2.69920500 0.50710300 -1.24817100

H 1.43302400 4.33398000 0.28362400

H 3.09812100 2.92744000 -0.88856200

N 0.16962500 -0.70828100 -0.64376600

H -4.21354000 -1.98530600 -0.47681800

O 2.68127000 -1.07371100 1.56750600

H 2.08944400 -1.83652800 1.71876400

H 3.01166300 -1.27214400 0.67288500

O 0.89751300 -3.17548600 1.01385300

H 0.39932500 -2.44696700 0.60132100

H 1.55571500 -3.36928600 0.32240300

O 2.48917300 -2.17117600 -1.04960200

H 2.83994300 -2.31102900 -1.94175300

H 1.60517300 -1.74559600 -1.15848800

**O**

E (M06-2X-SMD/6-31G(D)) = -517.270062372 au

H (M06-2X-SMD/6-31G(D)) = -517.081696 au

G (M06-2X-SMD/6-31G(D)) = -517.125411 au

E (M06-2X-SMD/DEF2-TZVP//M06-2X-SMD/6-31G(D)) = -517.459978846 au

C -0.72491200 0.50494500 0.00025600

C -1.12677800 -0.85254600 0.00022500

C -1.69653900 1.51107600 0.00005000

C -2.47460000 -1.22114800 -0.00009000

C -3.03710700 1.15111200 -0.00000800

H -1.40403100 2.55753700 0.00046100

C -3.41743200 -0.20252000 -0.00011600

H -2.76922500 -2.26603900 0.00000600

H -3.80276900 1.92052500 -0.00021200

C 0.72503300 0.50486400 -0.00019900

C 1.12671100 -0.85278400 -0.00015600

C 1.69653500 1.51100800 -0.00004400

C 2.47460900 -1.22114300 0.00004500

C 3.03716600 1.15113600 -0.00006500

H 1.40386800 2.55742600 0.00042600

C 3.41740600 -0.20246600 -0.00000500

H 2.76944800 -2.26598100 0.00027100

H 3.80276800 1.92060300 -0.00009400

H 4.47276000 -0.45860800 -0.00005600

N -0.00004400 -1.64816300 -0.00009900

H -4.47282600 -0.45847900 -0.00030900

H -0.00024300 -2.65905200 0.00083100

**(H_2_O)OH^-^**

E (M06-2X-SMD/6-31G(D)) = -228.642827915 au

H (M06-2X-SMD/6-31G(D)) = -228.576256 au

G (M06-2X-SMD/6-31G(D)) = -228.611456 au

E (M06-2X-SMD/DEF2-TZVP//M06-2X-SMD/6-31G(D)) = -228.805500736 au

O -1.53235500 -0.59899200 0.01806700

H -1.05522800 0.29542500 -0.02584100

O 1.34095100 -0.79953900 -0.08501700

H 1.21653000 -1.02301200 0.84824400

H -0.75753700 -1.15001300 -0.17845600

O 0.12788900 1.40556400 -0.10126600

H 0.19597000 1.64686200 0.83483000

H 0.90839000 0.17447700 -0.13304800

**D′**

E (M06-2X-SMD/6-31G(D)) = -1035.92442608 au

H (M06-2X-SMD/6-31G(D)) = -1035.727924 au

G (M06-2X-SMD/6-31G(D)) = -1035.777373 au

E (M06-2X-SMD/DEF2-TZVP//M06-2X-SMD/6-31G(D)) = -1036.17332105 au

O 0.80609700 1.86149600 0.19224500

C -0.43303600 1.27783100 0.12568200

C -0.36676900 -0.07214900 -0.20663800

C -1.62436500 1.93637900 0.37076300

C -1.52842200 -0.82001500 -0.29971600

C -2.79737000 1.18607100 0.27215100

H -1.64780700 2.98910000 0.62909500

C -2.73704800 -0.16551800 -0.05705000

H -1.50680900 -1.87524500 -0.55307100

H -3.75851000 1.65533600 0.45275800

C 1.73990300 0.90268800 -0.12871600

C 1.61799000 -1.43140800 0.62558800

C 3.04812300 1.18437900 -0.23256900

C 2.91899100 -1.76552200 0.66103900

H 0.90986300 -1.88274400 1.31666700

C 4.04423100 0.16419900 -0.47576000

H 3.36946200 2.21812400 -0.14209900

C 3.98154600 -1.14280300 -0.10954900

H 3.22471900 -2.55953900 1.34037300

H 4.97285200 0.51001000 -0.92502500

H 4.86327300 -1.75256400 -0.29317800

Cl -4.22839200 -1.07712200 -0.17323100

C 1.08460700 -0.43810400 -0.38152100

H 1.31655700 -0.77954100 -1.40077100

**D′′**

E (M06-2X-SMD/6-31G(D)) = -1016.05884613 au

H (M06-2X-SMD/6-31G(D)) = -1015.850126 au

G (M06-2X-SMD/6-31G(D)) = -1015.900744 au

E (M06-2X-SMD/DEF2-TZVP//M06-2X-SMD/6-31G(D)) = -1016.29712455 au

C -0.49735000 1.48788600 0.10202200

C -0.31699000 0.10521100 -0.03258500

C -1.77434100 2.01588400 0.13770100

C -1.39065200 -0.77211500 -0.12400400

C -2.87962500 1.16123200 0.04305200

H -1.92916400 3.08516800 0.24919500

C -2.66463300 -0.20568200 -0.07925800

H -1.25258200 -1.84171300 -0.24045000

H -3.88964200 1.55430800 0.06888300

C 1.80882200 0.98018800 0.01959400

C 1.48642400 -1.40634500 0.59376500

C 3.13071600 1.05417400 -0.17728400

C 2.71774200 -1.91520300 0.50074900

H 0.72990600 -1.88090300 1.21626800

C 4.01350500 -0.05552600 -0.55084600

H 3.57542500 2.04446000 -0.12592700

C 3.82869000 -1.35766900 -0.28013800

H 2.91281300 -2.82360000 1.06420000

H 4.91085400 0.22958800 -1.09662100

H 4.58163500 -2.06701900 -0.61604300

Cl -4.05037300 -1.27383100 -0.19008800

N 1.04352500 -0.22534300 -0.05215900

C 0.85459500 2.14297100 0.23840900

H 1.02797900 2.93025600 -0.50116700

H 0.98300500 2.59193800 1.23120000

**D′′′**

E (M06-2X-SMD/6-31G(D)) = -1000.02660543 au

H (M06-2X-SMD/6-31G(D)) = -999.805827 au

G (M06-2X-SMD/6-31G(D)) = -999.856124 au

E (M06-2X-SMD/DEF2-TZVP//M06-2X-SMD/6-31G(D)) = -1000.25616225 au

C -0.49214600 1.39776300 0.14791600

C -0.36330100 0.04025000 -0.15718500

C -1.75188300 1.95542800 0.33917000

C -1.48075400 -0.77914600 -0.27013100

C -2.88441100 1.15083000 0.22407200

H -1.86336300 3.00966500 0.57691600

C -2.73128300 -0.20036100 -0.07682800

H -1.38842200 -1.83602100 -0.50277700

H -3.87664200 1.56579800 0.36655700

C 1.82318800 0.95692800 -0.12883200

C 1.57500900 -1.32249800 0.74805000

C 3.14556900 1.07232800 -0.34123800

C 2.83891600 -1.78190500 0.74341700

H 0.87250100 -1.65020700 1.51203900

C 4.03629800 -0.04219100 -0.59999100

H 3.59758800 2.06314800 -0.31479100

C 3.89682000 -1.31035200 -0.13184000

H 3.11575500 -2.55155800 1.46195100

H 4.95770100 0.19375600 -1.12922400

H 4.71479600 -2.00332700 -0.31859200

Cl -4.16177900 -1.20470100 -0.21722400

C 1.08561600 -0.35897200 -0.30559000

H 1.29861200 -0.78113200 -1.29950000

C 0.85774600 2.06992000 0.23515800

H 0.94457700 2.91565200 -0.45769400

H 1.04484400 2.46600900 1.24104600

**J**

E (M06-2X-SMD/6-31G(D)) = -1052.41135577 au

H (M06-2X-SMD/6-31G(D)) = -1052.212968 au

G (M06-2X-SMD/6-31G(D)) = -1052.262319 au

E (M06-2X-SMD/DEF2-TZVP//M06-2X-SMD/6-31G(D)) = -1052.66385287 au

O -0.86075700 1.85482600 -0.10038400

C 0.41885300 1.34533000 -0.01963700

C 0.35227200 -0.02664300 -0.19906800

C 1.60186600 2.02518900 0.20212600

C 1.48031600 -0.83333800 -0.16010500

C 2.74544600 1.23691900 0.23463600

H 1.63601200 3.09898800 0.34219700

C 2.67260000 -0.15467900 0.05768100

H 1.43863800 -1.90887600 -0.28608300

H 3.71131800 1.70009400 0.40232100

C -1.67377500 0.84100100 -0.32353700

C -1.58483800 -1.59411300 -0.53506300

C -3.13212900 0.98133500 -0.50056900

C -2.77034800 -1.93447300 -0.00653400

H -0.93820300 -2.28841600 -1.05768800

C -3.79062700 0.23357300 0.64007100

H -3.39296300 2.03912500 -0.51210800

C -3.63772700 -1.08804600 0.80176900

H -3.06220500 -2.97261900 -0.13653500

H -4.40898600 0.80655000 1.32311900

H -4.20106500 -1.59229500 1.58246900

Cl 4.14750500 -1.07580800 0.12349900

N -1.01410500 -0.30790700 -0.37685300

H -3.39679300 0.53059200 -1.46673900

**J′**

E (M06-2X-SMD/6-31G(D)) = -1036.33013841 au

H (M06-2X-SMD/6-31G(D)) = -1036.121464 au

G (M06-2X-SMD/6-31G(D)) = -1036.171552 au

E (M06-2X-SMD/DEF2-TZVP//M06-2X-SMD/6-31G(D)) = -1036.57508416 au

O -1.04412700 1.70165600 -0.21015200

C 0.27932100 1.22023700 -0.08978500

C 0.33747100 -0.10877200 -0.47263700

C 1.34587300 1.96459600 0.36014200

C 1.54425100 -0.78293800 -0.40412300

C 2.56209700 1.28515900 0.43237400

H 1.25079700 3.00573400 0.64567500

C 2.64318500 -0.05801700 0.06021400

H 1.63651400 -1.82528900 -0.68860000

H 3.44834700 1.80121600 0.78427200

C -1.80467200 0.73567700 -0.60993900

C -1.65255900 -1.80215600 -0.50847800

C -3.25696100 0.84926700 -0.61172000

C -2.57677800 -1.94248400 0.45105100

H -1.27184700 -2.67421100 -1.02991200

C -3.44962300 0.37583400 0.82741300

H -3.60795400 1.87021500 -0.75325300

C -3.15712500 -0.86598900 1.24978700

H -2.88001800 -2.95272000 0.71432700

H -3.78044700 1.13187200 1.53218600

H -3.38911000 -1.11228500 2.28348800

Cl 4.18070500 -0.86842600 0.18258600

H -3.69319100 0.15132300 -1.33107100

C -1.04447500 -0.49587700 -0.93935000

H -1.05209000 -0.49307300 -2.04954900

**J′′**

E (M06-2X-SMD/6-31G(D)) = -1016.51347462 au

H (M06-2X-SMD/6-31G(D)) = -1016.291265 au

G (M06-2X-SMD/6-31G(D)) = -1016.341183 au

E (M06-2X-SMD/DEF2-TZVP//M06-2X-SMD/6-31G(D)) = -1016.74653698 au

C 0.44957700 1.47653300 0.01953100

C 0.35441800 0.10373600 -0.18570500

C 1.69497200 2.04152200 0.24785500

C 1.43321700 -0.76177600 -0.16705800

C 2.81481300 1.21080600 0.25796500

H 1.80441600 3.10788800 0.41482500

C 2.67091800 -0.16184800 0.05326600

H 1.33900600 -1.83266000 -0.30700700

H 3.80206800 1.62595600 0.42865900

C -1.77186100 0.86077800 -0.33518800

C -1.48330800 -1.54900600 -0.54001600

C -3.23040900 0.80466300 -0.57377400

C -2.62313400 -2.00082000 0.01693600

H -0.78427500 -2.19844100 -1.05252800

C -3.80529700 0.07704000 0.62101000

H -3.62966700 1.81339100 -0.67203800

C -3.54401200 -1.22430200 0.82527000

H -2.80761200 -3.06691300 -0.08047400

H -4.44506500 0.63080600 1.30075700

H -4.04913900 -1.75271000 1.62938600

Cl 4.09049800 -1.17335400 0.07925400

H -3.42125500 0.23968400 -1.49653500

C -0.93071300 2.05794900 -0.04889700

H -1.25402200 2.53858600 0.88394700

H -1.06524100 2.79623900 -0.85020600

N -1.02896600 -0.22378000 -0.38475100

**J′′′**

E (M06-2X-SMD/6-31G(D)) = -1000.43320227 au

H (M06-2X-SMD/6-31G(D)) = -1000.200288 au

G (M06-2X-SMD/6-31G(D)) = - 1000.250510au

E (M06-2X-SMD/DEF2-TZVP//M06-2X-SMD/6-31G(D)) = -1000.65684605 au

C 0.20167700 1.36655400 -0.07257300

C 0.25669400 0.02962300 -0.47229500

C 1.35184200 2.00379300 0.37635500

C 1.43845700 -0.69609100 -0.43393000

C 2.55061800 1.29426300 0.42891500

H 1.32466800 3.04243300 0.69176300

C 2.57665100 -0.03841500 0.02616100

H 1.48535300 -1.73533300 -0.74369300

H 3.45791900 1.77010700 0.78498000

C -2.02654700 0.67530000 -0.43754700

C -1.49828200 -1.81513800 -0.57009400

C -3.45130800 0.61185200 -0.59647700

C -2.32866700 -2.09434300 0.46362500

H -1.03087400 -2.62575300 -1.12301100

C -2.97964700 0.27700800 0.84789900

H -4.01797400 1.53740800 -0.61591400

C -2.81102200 -1.03256900 1.27728800

H -2.48729900 -3.11806200 0.78464200

H -3.14893200 1.05541000 1.58138100

H -3.01256000 -1.25118800 2.32476800

Cl 4.08390200 -0.92092500 0.11054200

H -3.87316500 -0.23673500 -1.12997700

C -1.11870400 -0.42708100 -0.94272500

H -1.14740700 -0.37601900 -2.04434400

C -1.19210100 1.92947400 -0.21659100

H -1.52024700 2.51635900 0.64533600

H -1.27377700 2.57172000 -1.10320200

**MECP 2**

E (M06-2X-SMD /6-31G(D)) = -1051.8885573300 au

O 0.86454256 1.54301356 0.02570922

C -0.36294306 0.96409836 0.14048154

C -0.48678385 -0.45997713 0.16139166

C -1.47324882 1.79641301 0.13186345

C -1.80452988 -0.99539563 0.05814978

C -2.74689979 1.24492923 0.09598804

H -1.33091431 2.87201832 0.13442520

C -2.88852946 -0.15204859 0.03836891

H -1.91449678 -2.07408867 0.03479471

H -3.62341132 1.88262095 0.09407114

C 1.94463059 0.71785395 -0.00563219

C 1.79171570 -0.60194761 0.67767847

C 3.09481778 1.14423627 -0.61219127

C 2.98682303 -1.47799028 0.47009113

H 1.69115012 -0.39385521 1.76810166

C 4.20339507 0.28470765 -0.66910528

H 3.12266587 2.13034874 -1.06497498

C 4.11580530 -1.03473090 -0.14013850

H 2.90736099 -2.48961790 0.85631678

H 5.11285431 0.61333562 -1.15894924

H 4.96685481 -1.70053309 -0.24757635

Cl -4.50110034 -0.82442591 -0.04922864

N 0.55111747 -1.29234573 0.30359477
